# Benefits and harms of interventions to improve anxiety, depression, and other mental health outcomes for autistic people: A systematic review and network meta-analysis of randomised controlled trials

**DOI:** 10.1177/13623613221117931

**Published:** 2022-08-11

**Authors:** Audrey Linden, Lawrence Best, Freya Elise, Danielle Roberts, Aoife Branagan, Yong Boon Ernest Tay, Laura Crane, James Cusack, Brian Davidson, Ian Davidson, Caroline Hearst, William Mandy, Dheeraj Rai, Edward Smith, Kurinchi Gurusamy

**Affiliations:** 1 University College London, UK; 2 Autistica, UK; 3 Cheshire and Wirral Partnership NHS Foundation Trust, UK; 4 AutAngel Community Interest Company, UK; 5 University of Bristol, UK

**Keywords:** adolescents, adults, anxiety, autism spectrum disorders, depression, interventions – pharmacologic, interventions – psychosocial/behavioural, school-age children

## Abstract

Mental health difficulties are prevalent in autistic people with ~14%–50% having experienced depression and ~40%–80% having experienced anxiety disorders. Identifying interventions that improve autistic people’s mental health is a top priority. However, at present, there is no high-quality network meta-analysis of benefits and harms of different interventions. We conducted a systematic review and network meta-analysis of randomised controlled trials, searching MEDLINE, EMBASE, other databases, and trial registers until 17 October 2020. We included randomised controlled trials reporting anxiety or depression in a suitable format. We calculated effect estimates and 95% credible intervals using Bayesian network meta-analysis. Our search identified 13,794 reports, of which 71 randomised controlled trials (3630 participants) were eligible for inclusion. All trials had high risk of bias. The follow-up period ranged from 1 to 24 months. Evidence indicates uncertainty about the effects of different interventions, with more high-quality evidence needed. Available evidence suggests that some forms of cognitive behavioural therapy may decrease anxiety and depression scores in autistic children and adults; mindfulness therapy may decrease anxiety and depression scores in autistic adults with previous mental health conditions; and behavioural interventions may provide some benefit for depression in autistic children. We recommend that autistic people are given access to mental health interventions available to non-autistic people, following principles of person-centred care.

**PROSPERO registration ID:** CRD42019136093

**Lay Abstract:**

Nearly three out of four autistic people experience mental health problems such as stress, anxiety or depression. The research already done does not guide us on how best to prevent or treat mental health problems for autistic people. Our aim was to look at the benefits and harms of different interventions on mental health outcomes in autistic people. We searched all the published randomised controlled trials (RCTs) about interventions for mental health conditions in autistic people until 17 October 2020. We also searched for RCTs that were not published in peer-reviewed journals. These were obtained from registers of clinical trials online. We then combined the information from all these trials using advanced statistical methods to analyse how good the interventions are. Seventy-one studies (3630 participants) provided information for this research. The studies reported how participants were responding to the intervention for only a short period of time. The trials did not report which interventions worked for people with intellectual disability. In people without intellectual disability, some forms of cognitive behavioural therapy and mindfulness therapy may be helpful. However, further research is necessary. Many trials used medications to target core features of autism rather than targeting mental health conditions, but these medications did not help autistic people. Until we have more evidence, treatment of mental health conditions in autistic people should follow the evidence available for non-autistic people. We plan to widely disseminate the findings to healthcare professionals through medical journals and conferences and contact other groups representing autistic people.

## Introduction

Mental health problems are common in autistic people. For example, approximately 14% to 50% of autistic people have a current or previous history of depression (Hudson et al., 2019; Lever & Geurts, 2016; Rai et al., 2018) and 40% to 80% have a current or previous history of anxiety disorders (Kent & Simonoff, 2017). Identifying interventions that improve the mental health of autistic people is the number one research priority of the autism community (James Lind Alliance Priority Setting Partnerships, 2016). Here, we present the results of a systematic review and network meta-analysis (NMA) focused on interventions to improve anxiety or depression in autistic people (i.e. the most common mental health conditions that have been researched in relation to this group), as well as interventions to improve other mental health outcomes (e.g. quality of life) in autistic people (where reported by authors of interventions on anxiety and depression).

There are many different types of interventions for anxiety and depression among autistic people. These interventions include drugs such as antidepressants; psychological therapies such as cognitive behavioural therapy (CBT), counselling, and mindfulness-based therapy; behavioural interventions (e.g. interventions based on applied behaviour analysis (ABA)); other therapies such as music therapy; and waitlist (i.e. no additional intervention until the outcome is measured) (Choque Olsson et al., 2017; Chugani et al., 2016; Dean et al., 2017; Enticott et al., 2014; Hurwitz et al., 2012; McNally Keehn et al., 2013; Murphy et al., 2017; Politte et al., 2018; Spek et al., 2013; Xu et al., 2018). It is not always possible to conclude that the interventions that work for non-autistic people work in the same way for autistic people (see, for example, Babb et al., 2021; Tchanturia et al., 2016). However, while each autistic individual is different and may respond to interventions differently, it is important to understand how likely it is that an intervention will work, or how likely it is to cause harm, so that informed decision about which intervention to start can be made.

It is important that, whenever possible, information about the relative effects of different interventions is obtained from randomised controlled trials (RCTs), which ensure similar types of participants receive the compared interventions. RCTs overcome the problem of outcome differences due to differences in the type of people who received them. Therefore, research that includes evidence from RCTs including only autistic people (or reporting outcomes separately in autistic people) is important to address the significant uncertainty about the relative benefits and harms of different interventions designed to improve mental health (such as anxiety and depression) in autistic people.

Interventions for autistic people tend to fall within the medical model of disability (generally aimed at changing the autistic person themselves) or the social model of disability (aimed at making adaptations to an autistic person’s environment). Here, we focus on providing a comprehensive overview of existing RCTs, irrespective of the type of intervention being researched. We note that some of these interventions may not be feasible or acceptable for all autistic people (Bradley et al., 2015; Hoekstra et al., 2018). However, our review focuses on the existing research and does not include or exclude studies based on those taking a particular approach to mental health treatment.

Previous meta-analyses looking at interventions for anxiety in autistic people have focused on children and adolescents, and have examined specific interventions such as CBT (Kreslins et al., 2015; Perihan et al., 2020; Sukhodolsky et al., 2013; Ung et al., 2015), school-based interventions (Perihan et al., 2022), or use of specific medications (D’Alò et al., 2021; Deb et al., 2021). From this evidence, there is some indication that CBT may be effective compared to no intervention for autistic youth without intellectual disability (ID), although there is significant heterogeneity in findings. School-based interventions for anxiety show some promise for improving anxiety, although further evidence is needed. Finally, evidence for the use of anti-anxiety medications in autistic people is inconsistent (D’Alò et al., 2021; Deb et al., 2021).

While there are multiple meta-analyses examining the prevalence of anxiety and depression in autistic people, no meta-analysis looking at interventions for depression has been published in over a decade (Menezes et al., 2020). A recent systematic review of the evidence on treatment for depression in autistic people concluded that there is some suggestion that mindfulness-based therapy may be beneficial; however, the strength of evidence is poor and studies have inconsistent findings (Menezes et al., 2020). A meta-analysis looking at evidence for the effectiveness of antidepressants in autistic people, which focused on outcomes other than depression, found that evidence for the use of antidepressants is also contradictory (Deb et al., 2021).

Previous meta-analyses looking at other mental health outcomes in autistic people (i.e. aside from anxiety and depression) have predominantly focused on the prevalence of mental health conditions or risk factors for different mental health conditions (e.g. Lai et al., 2019). Few previous meta-analyses have included the impact of interventions on other mental health outcomes in autistic people. One exception is a meta-analysis looking at the effectiveness of antipsychotics for autistic people, which recorded no benefit of these medications on self-harm (D’Alò et al., 2021). There are no meta-analyses looking at the impact of interventions on quality of life in autistic people.

Standard meta-analyses conducted on the mental health of autistic people (as described above) have only compared each treatment to a waitlist control (or no additional intervention) group. Here, we use a technique called NMA to examine this topic. NMA allows for a combination of direct and indirect evidence and the ranking of different interventions for different outcomes (Salanti, 2012; Salanti et al., 2011). It also usually results in more precise estimates of treatment benefit or harm than examining direct or indirect evidence in isolation (Caldwell et al., 2015; Cooper et al., 2011). When people need to decide between more than two competing interventions, NMA provides information for comparisons between pairs of interventions that have never been evaluated within individual RCTs, thereby reducing the need for RCTs on the topic (Chaimani et al., 2019). Although previous NMAs have been conducted on mental health conditions (see Cortese et al., 2019 for a review), few have been conducted with autistic populations (for exceptions, see Fallah et al., 2019; Siafis et al., 2022), and no existing systematic reviews on anxiety or depression in autism have attempted NMA.

Here, our objectives were to compare relative benefits and harms of different interventions to improve mental health of autistic people via a systematic review and NMA of RCTs, as well as to identify research gaps.

## Methods

The protocol was registered in PROSPERO prior to project commencement (registration number: CRD42019136093). We conducted and reported the systematic review according to Preferred Reporting Items for Systematic Reviews and Meta-Analyses (PRISMA) statement (2021) and its extension for NMA (Hutton et al., 2015; Page et al., 2021).

### Criteria for considering studies for this review

We included all RCTs regardless of publication status, year of publication, language of publication, and the setting, if they reported anxiety or depression in a suitable format for analysis. We included all interventions where anxiety or depression was assessed, irrespective of whether these were primary outcomes of the RCT. As mental health is a priority research area for autistic people, it was important to include any intervention that may have had an impact on mental health. We included any study focused on autistic people (e.g. of all ages, levels of intellectual ability). Separate meta-analyses were planned for children and adolescents without ID, children and adolescents with ID, adults with ID, and adults without ID.

#### Types of interventions

We included any of the following interventions for comparison with one another (either alone or in combination):

- Drugs such as selective serotonin reuptake inhibitors (SSRIs), serotonin and norepinephrine reuptake inhibitors (SNRIs), antipsychotics, antioxidants, other medications such as oxytocin, anti-diuretic hormone (ADH).
- Psychological therapies such as CBT, mindfulness-based therapy, counselling.
- Behavioural therapies such as social skills training, ABA.
- Miscellaneous interventions such as music therapy, parent psychoeducation, dietary supplements.
- Wait-list (i.e. no additional intervention or placebo intervention until measurement of the outcomes).

### Outcomes

We included the following outcome measures, based on previous reviews (e.g. Hurwitz et al., 2012) and input from clinicians.

#### Primary outcomes

1. Anxiety or depression using any validated measure;
2. Overall health-related quality of life (HRQoL) using any validated measure;
3. Serious adverse events.

#### Secondary outcomes

4. Mental health–related quality of life;
5. Presence of self-harm or number of attempts;
6. Suicidal thoughts or attempted suicide;
7. Non-serious adverse events;
8. Any adverse events;
9. Psychotic symptoms;
10. Post-traumatic stress disorder (PTSD);
11. Employment status;
12. Meaningful life activities;
13. All-cause mortality.

All outcomes were collected until the latest time point post-intervention when outcomes were reported.

### Search methods for identification of studies

We searched MEDLINE, EMBASE, Cochrane library, PsycINFO, CINAHL Plus, Science Citation Index, and trial registers until 17 October 2020, and reference lists of included trials and related systematic reviews. We did not apply language restrictions. We searched for all possible comparisons formed by the interventions of interest. To identify further ongoing or completed trials, we also searched ClinicalTrials.gov and the World Health Organization International Clinical Trials Registry Platform (apps.who.int/trialsearch/), which searches various trial registers, including ISRCTN and ClinicalTrials.gov. For the complete search strategy, see Supplemental Appendix 1.

We searched the references of the identified trials and the existing systematic reviews on autism and mental health interventions to identify additional trials for inclusion. We also contacted the study authors to identify further trials and obtain aggregate data from unpublished studies. We acknowledge that the searches were last performed in October 2020. However, by including the searches of clinical trial registers and thorough search of conference abstracts, we aimed to minimise the number of studies that would be eligible for inclusion beyond October 2020.

### Data collection

Two review authors from the review author team independently identified trials for inclusion by screening the titles and abstracts and short-listed reports (after translation if required). We resolved any discrepancies through discussion and arbitration. Two review authors independently extracted data related to the participants, interventions, and outcomes using a pre-piloted data extraction form. We used the Cochrane risk-of-bias tool for randomised trials (RoB 2.0) (Sterne et al., 2019) for assessment of risk of bias.

### Data synthesis

We conducted NMA on all outcomes with multiple intervention comparisons. We obtained a network plot to understand the network geometry and ensure that trials were connected by interventions using Stata/SE15 (Chaimani & Salanti, 2012). We conducted a Bayesian NMA using the Markov chain Monte Carlo method in OpenBUGS 3.2.3 as per guidance from the National Institute for Health and Care Excellence (NICE) Decision Support Unit (DSU) documents (Dias, Welton, Sutton, & Ades, 2011a) using study-level data and appropriate likelihood and link functions. We used ‘wait-list, treatment-as-usual, or placebo’ as the reference group (‘no additional intervention’). We calculated effect estimates with 95% credible intervals (CrI) (Severini, 1993). We performed the meta-analysis using a fixed-effect model and random-effects model for the NMA and reported the more conservative model. We performed an intention-to-treat analysis whenever possible (Newell, 1992); otherwise, we used the data available to us. We conducted best-worst-case and worst-best-case scenario analyses as sensitivity analyses for binary outcomes whenever possible. For continuous outcomes, although we planned to impute the mean and/or standard deviation from median and p values according to guidance in the Cochrane Handbook if the data appeared to be normally distributed, we were unable to make judgements on the distribution of data (Higgins et al., 2011); therefore, we did not impute these data.

### Assessment and investigation of heterogeneity and inconsistency

We assessed clinical and methodological heterogeneity by carefully examining the characteristics and design of included trials. We avoided two major sources of clinical heterogeneity by performing separate meta-analyses based on age and ID. We investigated heterogeneity through subgroup analyses and meta-regression using methods and codes described in the NICE DSU documents (Dias, Sutton, et al., 2011). We assessed statistical heterogeneity by comparing results of the fixed-effect model meta-analysis and the random-effects model meta-analysis and calculating the between-study standard deviation (tau) (Turner et al., 2012) and NMA-specific I^2^ (Jackson et al., 2014).

We evaluated the plausibility of transitivity assumption (the assumption that any participant that meets the inclusion criteria is, in principle, equally likely to be randomised to any of the above eligible interventions) by looking at the inclusion and exclusion criteria in the studies. We assessed inconsistency (statistical evidence of the violation of transitivity assumption) by fitting both an inconsistency model (Dias, Welton, et al., 2011) and a consistency model (agreement between direct and indirect estimates for the same treatment comparison), when direct and indirect evidence was available. In addition, we used design-by-treatment full interaction model and inconsistency factor (IF) plots to assess inconsistency (Chaimani & Salanti, 2012; Dias, Welton, Sutton, & Ades, 2011b). Where there were no closed loops (direct comparisons involving comparison of three or more interventions with each other), it is not possible to assess inconsistency.

#### Sensitivity analysis

We performed best-worst-case scenario and worst-best-case scenario sensitivity analyses to assess the impact of missing data.

#### Assessment of reporting biases

For the NMA, we planned to perform a comparison-adjusted funnel plot. However, there was no meaningful way in which to rank these studies (i.e. there was no specific change in the risk of bias in the studies, sample size, or the control group used over time), we judged this reporting bias by the completeness of the search (Chaimani & Salanti, 2012) (i.e. identify completed but unpublished trials from the trial registry for which we are unable to obtain data from the study authors). Therefore, we assessed reporting bias by the completeness of searches and absence of reporting of results. In addition, in our supplementary tables, we have summarised information on the number of studies in which mental health outcomes were not measured or were not reported in an analysable format for each included comparison (to provide an indication of reporting biases), and the overall number of studies in which mental health outcomes were not measured or reported to provide an indication of the opportunity lost in adequately measuring or reporting the outcomes that are most important to autistic people.

### Community involvement statement

Our research team includes both autistic and non-autistic researchers and lay members, who had input into all stages of the project, including development of the grant submission, study design and the drafting/dissemination of the study. We additionally held focus groups with autistic people to establish prioritisation of outcomes through group discussion, which included autistic lay members and autistic researchers.

## Results

### Searches and characteristics of included studies

**Figure 1.**
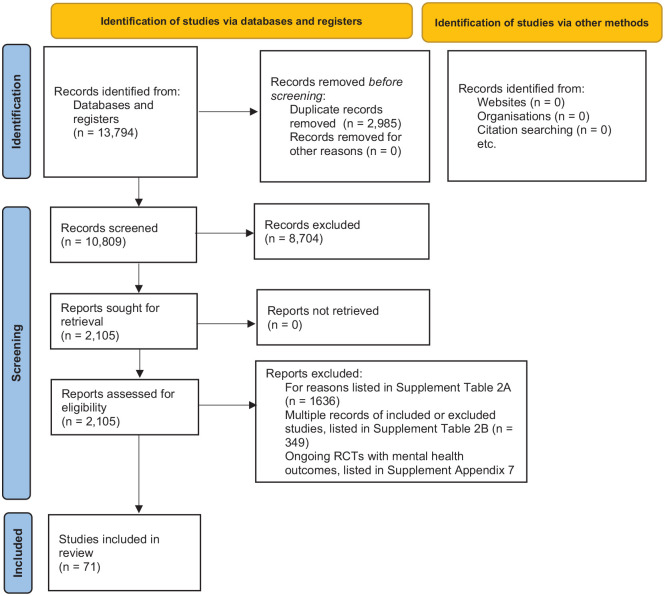
Reference flow.

We identified 13,794 records through electronic searches. The reference flow is shown in Figure 1. We included a total of 71 trials (3630 participants) in this review. Reasons for exclusion of remaining records included studies not reporting results for autistic people separately, and not measuring or reporting mental health outcomes. Full details of excluded studies are available in Supplemental Table 2A and Supplemental Table 2B. There were several deviations from the protocol, the reasons for which are described in detail in the Supplement: ‘Deviations from Protocol’. However, overall, none of these deviations would have resulted in different conclusions from those stated here.

In the included studies, 387 participants were excluded after randomisation, leaving a total of 3243 participants included in one or more mental health outcomes. Sample sizes in the trials varied from 11 to 223 participants. Only six trials had sample sizes of 100 or more participants (Cortesi et al., 2012; Dean et al., 2017; Reddihough et al., 2019; Squassante et al., 2018; Wood et al., 2020; Yamasue et al., 2020). The follow-up period in the trials ranged from 1 month to 24 months. Only one trial had a follow-up longer than 12 months (Bischof et al., 2018).

The characteristics of included studies are summarised in Supplemental Table 1. Overall, most trials included only people without ID or included only a small number of participants with ID. No trials included only participants with ID or reported mental health outcomes in the subset of people with ID. Risk of bias is summarised in Supplemental Table 4. All trials had some concerns about bias or were at high risk of bias in at least one domain. Furthermore, all trials had some concerns about bias or were at high risk of bias overall.

### Effect estimates

A summary of the number of trials and participants for the outcomes reported by at least one trial is available in Table 1. Estimates of effect size are reported in Table 2. No trials reported suicidal thoughts or attempted suicide, psychotic symptoms, PTSD, employment status, meaningful life activities, or all-cause mortality (although we could infer that there were no deaths in several trials as they reported mental health outcomes for all randomised participants).

**Table 1.** Summary of outcomes.

Outcome	Number of studies	Total number of participants	Number of trials included in network meta-analysis (NMA)
Children: Proportion of patients with anxiety	4	78	NMA was not performed for this outcome measure
Children: Anxiety scores	44	1966	42
Children: Anxiety change scores	12	804	12
Adults: Anxiety scores	13	526	13
Adults: Anxiety change scores	2	121	2
Children: Depression scores	7	231	7
Adults: Depression scores	10	448	10
Adults: Depression change scores	1	40	NMA was not performed for this outcome measure
Children: Quality of life	2	87	2
Adults: Quality of life	1	48	NMA was not performed for this outcome measure
Adults: Change in Quality of life	2	95	NMA was not performed for this outcome measure
Serious adverse events	9	328	NMA was not performed for this outcome measure
Adults: Mental health–related quality of life	1	48	NMA was not performed for this outcome measure
Self-harm	1	41	NMA was not performed for this outcome measure
Non-serious adverse events – number of people	7	183	4
Non-serious adverse events – number of events	1	12	NMA was not performed for this outcome measure
Proportion of people with adverse events	9	337	5
Number of adverse events	8	568	8
Proportion of people who died	1	20	NMA was not performed for this outcome measure

NMA: network meta-analysis.

We have listed study names here. Full references for these studies can be found in Supplemental Appendix 9.

**Table 2.** Summary of findings (certainty of evidence).

Interventions	Anticipated absolute effect (95% CrI)		Certainty of evidence
	Various interventions	Difference	
Anxiety (scores) (children) Total studies: 42 Total participants: 1831			
No additional intervention	Reference		
CBT: Adapted-Group	SMD 1.44 lower (2.47 lower to 0.48 lower) Network estimate	Same as previous column	Very low certainty evidence^a,c,d^
CBT: Adapted-Family-based	SMD 1.09 lower (1.88 lower to 0.35 lower) Network estimate	Same as previous column	Very low certainty evidence^a,c,d^
CBT: Adapted-Individual	SMD 0.75 lower (1.74 lower to 0.23 higher) Network estimate	Same as previous column	Very low certainty evidence^a,c,d,e^
Medication: Anti-diuretic hormone	SMD 1.04 higher (0.95 lower to 3.02 higher) Network estimate	Same as previous column	Very low certainty evidence^a,c,d,e^
Skills training	SMD 0.53 lower (1.92 lower to 0.85 higher) Network estimate	Same as previous column	Very low certainty evidence^a,c,d,e^
CBT: Non-adapted-Family-based	SMD 1.35 lower (2.77 lower to 0.02 higher) Network estimate	Same as previous column	Very low certainty evidence^a,c,d,e^
CBT: Non-adapted-Self-directed	SMD 0.44 lower (2.38 lower to 1.47 higher) Network estimate	Same as previous column	Very low certainty evidence^a,c,d,e^
Medication: Oxytocin	SMD 1.06 lower (3.02 lower to 0.91 higher) Network estimate	Same as previous column	Very low certainty evidence^a,c,d,e^
Medication: Serotonin-Norepinephrine Reuptake Inhibitors	SMD 0.05 higher (1.05 lower to 1.16 higher) Network estimate	Same as previous column	Very low certainty evidence^a,c,d,e^
Skills Training-Group	SMD 0.10 lower (1.55 lower to 1.31 higher) Network estimate	Same as previous column	Very low certainty evidence^a,c,d,e^
CBT: Non-adapted-Individual	SMD 0.64 lower (2.39 lower to 1.13 higher) Network estimate	Same as previous column	Very low certainty evidence^a,c,d,e^
Group activity	SMD 1.46 lower (3.70 lower to 0.63 higher) Network estimate	Same as previous column	Very low certainty evidence^a,c,d,e^
Medication: Selective Serotonin Reuptake Inhibitors	SMD 0.19 lower (1.52 lower to 1.14 higher) Network estimate	Same as previous column	Very low certainty evidence^a,c,d,e^
Skills Training-Group-PEERS	SMD 0.23 higher (1.11 lower to 1.59 higher) Network estimate	Same as previous column	Very low certainty evidence^a,c,d,e^
Skills Training-MASSI	SMD 0.32 lower (2.30 lower to 1.63 higher) Network estimate	Same as previous column	Very low certainty evidence^a,c,d,e^
Book reading	SMD 0.84 lower (3.10 lower to 1.49 higher) Network estimate	Same as previous column	Very low certainty evidence^a,c,d,e^
CBT: Adapted-Parent-mediated	SMD 0.77 lower (2.76 lower to 1.20 higher) Network estimate	Same as previous column	Very low certainty evidence^a,c,d,e^
CBT: Family-based-Exposure-focussed	SMD 2.24 lower (4.30 lower to 0.18 lower) Network estimate	Same as previous column	Very low certainty evidence^a,c,e^
CBT: Non-adapted-Group	SMD 2.80 higher (0.19 higher to 5.43 higher) Network estimate	Same as previous column	Low certainty evidence^a,c,e^
Counselling	SMD 0.67 lower (3.44 lower to 2.06 higher) Network estimate	Same as previous column	Very low certainty evidence^a,c,d,e^
Distraction	SMD 0.34 lower (2.31 lower to 1.58 higher) Network estimate	Same as previous column	Very low certainty evidence^a,c,d,e^
Medication: *N*-acetyl cysteine	SMD 0.30 lower (2.16 lower to 1.58 higher) Network estimate	Same as previous column	Very low certainty evidence^a,c,d,e^
Parent psychoeducation	SMD 0.85 lower (2.89 lower to 1.19 higher) Network estimate	Same as previous column	Very low certainty evidence^a,c,d,e^
Skills Training-Group-SENSE	SMD 0.60 lower (2.44 lower to 1.26 higher) Network estimate	Same as previous column	Very low certainty evidence^a,c,d,e^
Skills Training-Self-directed	SMD 0.16 lower (2.91 lower to 2.65 higher) Network estimate	Same as previous column	Very low certainty evidence^a,c,d,e^
Skills Training-Video	SMD 0.17 higher (1.72 lower to 2.09 higher) Network estimate	Same as previous column	Very low certainty evidence^a,c,d,e^
Skills Training-Video plus Distraction	SMD 0.11 lower (2.06 lower to 1.80 higher) Network estimate	Same as previous column	Very low certainty evidence^a,c,d,e^
Anxiety (scores) (children): change Total studies: 12 Total participants: 804			
No additional intervention	Reference		
CBT: Adapted-Group	SMD 1.80 lower (4.15 lower to 0.37 higher) Network estimate	Same as previous column	Very low certainty evidence^a,c,d,e^
CBT: Adapted-Family-based	SMD 0.77 lower (3.95 lower to 2.43 higher) Network estimate	Same as previous column	Very low certainty evidence^a,c,d,e^
CBT: Adapted-Individual	SMD 0.95 lower (3.88 lower to 1.99 higher) Network estimate	Same as previous column	Very low certainty evidence^a,c,d,e^
Medication: Anti-diuretic hormone	SMD 1.04 lower (4.24 lower to 2.15 higher) Network estimate	Same as previous column	Very low certainty evidence^a,c,d,e^
CBT: Non-adapted-Family-based	SMD 1.77 lower (4.95 lower to 1.42 higher) Network estimate	Same as previous column	Very low certainty evidence^a,c,d,e^
CBT: Non-adapted-Individual	SMD 0.34 lower (2.56 lower to 1.91 higher) Network estimate	Same as previous column	Very low certainty evidence^a,c,d,e^
Dietary supplement	SMD 0.01 lower (2.24 lower to 2.21 higher) Network estimate	Same as previous column	Very low certainty evidence^a,c,d,e^
Medication: Selective Serotonin Reuptake Inhibitors	SMD 0.03 lower (2.24 lower to 2.19 higher) Network estimate	Same as previous column	Very low certainty evidence^a,c,d,e^
CBT: Non-adapted-Individual plus Medication: Melatonin	SMD 1.70 lower (4.66 lower to 1.28 higher) Network estimate	Same as previous column	Very low certainty evidence^a,c,d,e^
Medication: Melatonin	SMD 0.33 lower (3.26 lower to 2.63 higher) Network estimate	Same as previous column	Very low certainty evidence^a,c,d,e^
Medication: Noradrenergic and Specific Serotonergic Antidepressant	SMD 0.34 lower (3.51 lower to 2.86 higher) Network estimate	Same as previous column	Very low certainty evidence^a,c,d,e^
Anxiety (scores) (adults) Total studies: 13 Total participants: 526			
No additional intervention	Reference		
CBT: Adapted-Group	SMD 0.37 higher (1.09 lower to 1.87 higher) Network estimate	Same as previous column	Very low certainty evidence^a,c,e^
Medication: Anti-diuretic hormone	SMD 0.55 higher (1.08 lower to 2.16 higher) Network estimate	Same as previous column	Very low certainty evidence^a,c,e^
Skills Training	SMD 0.50 lower (1.98 lower to 0.97 higher) Network estimate	Same as previous column	Very low certainty evidence^a,c,e^
CBT: Non-adapted-Self-directed	SMD 0.05 lower (1.60 lower to 1.42 higher) Network estimate	Same as previous column	Very low certainty evidence^a,c,e^
Medication: Oxytocin	SMD 0.16 higher (0.89 lower to 1.18 higher) Network estimate	Same as previous column	Very low certainty evidence^a,c,e^
Mindfulness	SMD 0.41 lower (1.27 lower to 0.48 higher) Network estimate	Same as previous column	Very low certainty evidence^a,c,e^
Skills Training-Group	SMD 0.04 lower (1.55 lower to 1.47 higher) Network estimate	Same as previous column	Very low certainty evidence^a,c,e^
CBT: Adapted-Self-directed	SMD 0.72 lower (2.21 lower to 0.75 higher) Network estimate	Same as previous column	Very low certainty evidence^a,c,e^
Medication: Atypical Anti-psychotic	SMD 0.61 lower (2.15 lower to 0.92 higher) Network estimate	Same as previous column	Very low certainty evidence^a,c,e^
Medication: 3,4-Methylenedioxymethamphetamine	SMD 0.80 lower (2.65 lower to 1.05 higher) Network estimate	Same as previous column	Very low certainty evidence^a,c,e^
Skills Training-Individual	SMD 0.63 lower (2.15 lower to 0.89 higher) Network estimate	Same as previous column	Very low certainty evidence^a,c,e^
Anxiety (scores) (adults): change Total studies: 2 Total participants: 121			
No additional intervention	Reference		
Medication: Anti-diuretic hormone	SMD 0.21 lower (1.12 lower to 0.73 higher) Network estimate	Same as previous column	Very low certainty evidence^a,b,c,e^
Medication: Oxytocin	SMD 0.12 higher (0.27 lower to 0.51 higher) Network estimate	Same as previous column	Very low certainty evidence^a,b,c,e^
Depression (scores) (children) Total studies: 7 Total participants: 231			
No additional intervention	Reference		
CBT: Adapted-Group	SMD 0.31 higher (2.27 lower to 3.00 higher) Network estimate	Same as previous column	Very low certainty evidence^a,b,c,e^
Skills training	SMD 0.45 lower (4.06 lower to 3.17 higher) Network estimate	Same as previous column	Very low certainty evidence^a,b,c,e^
Applied behaviour analysis	SMD 1.01 lower (4.71 lower to 2.70 higher) Network estimate	Same as previous column	Very low certainty evidence^a,b,c,e^
Skills Training-Group-PEERS	SMD 0.37 lower (2.95 lower to 2.23 higher) Network estimate	Same as previous column	Very low certainty evidence^a,b,c,e^
Individual CBT	SMD 0.32 lower (4.01 lower to 3.40 higher) Network estimate	Same as previous column	Very low certainty evidence^a,b,c,e^
Depression (scores) (adults) Total studies: 10 Total participants: 448			
No additional intervention	Reference		
CBT: Adapted-Group	SMD 0.04 higher (2.70 lower to 2.83 higher) Network estimate	Same as previous column	Very low certainty evidence^a,c,e^
Skills Training	SMD 0.48 lower (3.28 lower to 2.30 higher) Network estimate	Same as previous column	Very low certainty evidence^a,c,e^
CBT: Non-adapted-Self-directed	SMD 0.50 lower (3.13 lower to 2.09 higher) Network estimate	Same as previous column	Very low certainty evidence^a,c,e^
Medication: Oxytocin	SMD 0.03 higher (2.72 lower to 2.80 higher) Network estimate	Same as previous column	Very low certainty evidence^a,c,e^
Mindfulness	SMD 0.52 lower (2.12 lower to 1.11 higher) Network estimate	Same as previous column	Very low certainty evidence^a,c,e^
Group activity	SMD 0.47 higher (3.42 lower to 4.41 higher) Network estimate	Same as previous column	Very low certainty evidence^a,c,e^
CBT: Adapted-Self-directed	SMD 0.72 lower (3.48 lower to 2.04 higher) Network estimate	Same as previous column	Very low certainty evidence^a,c,e^
Medication: Atypical Anti-psychotic	SMD 0.53 lower (3.31 lower to 2.24 higher) Network estimate	Same as previous column	Very low certainty evidence^a,c,e^
Skills Training-Individual	SMD 0.76 lower (3.55 lower to 2.02 higher) Network estimate	Same as previous column	Very low certainty evidence^a,c,e^
Depression (scores) (adults): change Total studies: 1 Total participants: 40			
No additional intervention	Reference		
Medication: Oxytocin	SMD 0.25 lower (0.38 lower to 0.87 higher) Direct estimate	Same as previous column	Very low certainty evidence^a,b,c,e^

CBT: cognitive behavioural therapy; SMD: standardised mean difference.

a Downgraded one level for risk of bias.

b Downgraded one level for imprecision due to small sample size.

c Downgraded one level for reporting bias.

d Downgraded one level for heterogeneity.

e Downgraded one level for imprecision due to poor overlap of confidence intervals.

**Table 3.** Summary of abbreviations used for interventions.

Abbreviation	Full name
ABA	Applied behaviour analysis
CBT	Cognitive behavioural therapy
CBT Adapted	CBT that was adapted for autistic people
MDMA	3,4-Methylenedioxymethamphetamine (ecstasy)
NAC	*N*-acetyl cysteine
NaSSAs	Noradrenergic and specific serotonergic antidepressants
SNRI	Serotonin and norepinephrine reuptake inhibitors (antidepressants)
SSRI	Selective serotonin reuptake inhibitors (antidepressants)
PEERS skills training	Social skills training based on the Program for the Education and Enrichment of Relational Skills
MASSI skills training	Skills training based on Multimodal Anxiety and Social Skill Intervention

**Figure 2.**
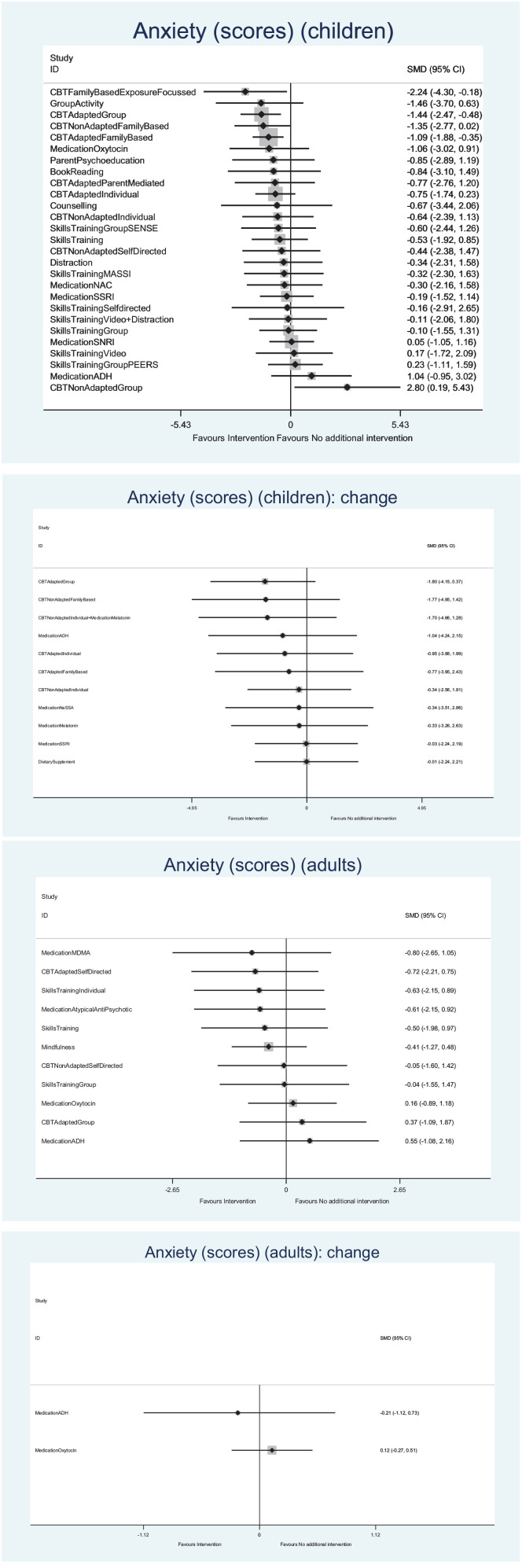
Forest plots (anxiety). The abbreviations for the interventions shown in the forest plot are available in Supplemental Appendix 2.

**Figure 3.**
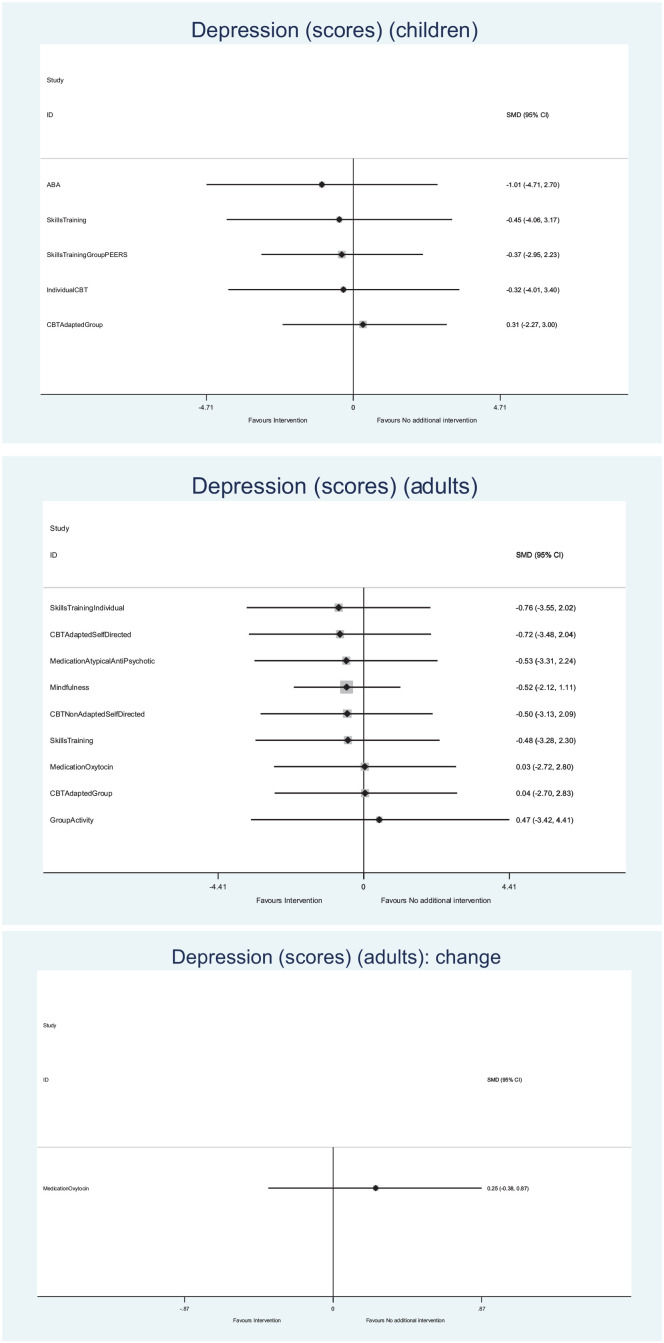
Forest plots (depression). The abbreviations for the interventions shown in the forest plot are available in Supplemental Appendix 2.

Many findings were examined, a summary of which are presented below^1^ (Table 2; Figures 2 and 3). Where specific interventions are mentioned, this is because these interventions were examined in the studies that we were able to include for analysis. Where ‘final scores’ are mentioned, this refers to the scores on a measure (e.g. of the severity of anxiety or depression) at the final time point assessed in the studies. For interventions in children, the mean age was 10 years (median 10; range 2–17 years). Note that we report outcomes as they were assessed within the original studies.

#### Anxiety in children

##### Proportion of participants with a diagnosis of anxiety

Only direct comparisons were performed because of the nature of the comparisons. The first comparison compared parent psychoeducation with no additional intervention. The proportion of participants with anxiety was lower in participants whose parents received psychoeducation (0.15; 95% CrI: 0.02 to 0.87; one trial; 24 participants; control group proportion: 76.9%; very low certainty evidence). The second comparison looked at counselling versus MASSI skills training (Multimodal Anxiety and Social Skill Intervention). There was no evidence of difference in the proportion of participants with anxiety after these two interventions (0.32; 95% CrI: 0.05 to 1.61; one trial; 32 participants; control group proportion: 80%; very low certainty evidence). Overall, from these data, we conclude that there is no strong evidence for the effectiveness of a particular intervention in terms of reducing the proportion of participants with a diagnosis of anxiety.

##### Anxiety – final scores for severity of anxiety

A random-effects model was used as it had better model fit than a fixed-effect model. The between-study variance (variability in studies over and above that expected due to random sampling error) was 0.75 (95% CrI: 0.31 to 1.87). There was no evidence of inconsistency according to the inconsistency model fit, treatment-by-design (95% CrI: 0.00 to 15.93), and IF plot. The effect estimates are shown in Table 2. There were several comparisons in which participants had lower or higher scores in one intervention than another intervention (very low certainty evidence). Overall, from these data, we conclude that many CBT interventions yielded lower anxiety scores in those receiving CBT interventions compared to other interventions.

##### Anxiety – change scores

The results presented are the differences in the change from baseline between two interventions rather than the change from baseline in a specific group. A random-effects model was used as it was more conservative, although the model fit statistics was similar in the fixed-effects and random-effects model. The between-study variance was 1.26 (95% CrI: 0.22 to 11.66).

The effect estimates are shown in Table 2. Overall, from these data, we conclude that there was no evidence of differences in the change in anxiety scores between any of the comparisons in the NMA (very low certainty evidence), although in the direct comparisons, some forms of CBT resulted in lower anxiety scores than no additional intervention (very low certainty evidence). Ranking the effectiveness of interventions, to enable us to recommend one treatment over another, was deemed inappropriate because of the uncertainty in evidence.

#### Anxiety in adults

##### Proportion of participants with anxiety

None of the trials reported the proportion of adult participants with a diagnosis of anxiety.

##### Anxiety – final scores

A random-effects model was used as it was more conservative, although the model fit statistics were similar in the fixed-effect and random-effects models. The between-study variance was 0.10 (95% CrI: 0.00 to 4.15). The effect estimates are shown in Table 2. There was no evidence of differences in the final anxiety scores in any of the comparisons in the NMA (very low certainty evidence), although in the direct comparisons, some forms of CBT resulted in lower anxiety scores than no additional intervention (low certainty evidence). Overall, ranking the effectiveness of interventions in terms of their benefit or harm was deemed inappropriate because of the uncertainty in evidence. However, there was some indication that some forms of CBT may provide some benefit for anxiety in autistic adults when compared to offering no intervention.

##### Anxiety – change scores

As there was only one study for each comparison, only the fixed-effect model was applicable. As indicated in Table 2 (effect size estimates), there was no evidence of differences in the change in anxiety scores in any of the comparisons in the direct comparisons or NMA (very low certainty evidence). Therefore, in terms of change score for anxiety, we cannot recommend any specific intervention over another.

#### Depression in children

##### Proportion of participants with depression

None of the trials reported the proportion of participants with a diagnosis of depression.

##### Depression – final scores

A random-effects model was used as it was more conservative, although the model fit statistics were similar in the fixed-effect and random-effects models. The between-study variance was 0.87 (95% CrI: 0.01 to 17.93). The effect estimates are shown in Table 2. There was no evidence of differences in final depression scores in any of the comparisons in the NMA (very low certainty evidence), although in the direct comparisons, behavioural interventions resulted in lower depression scores than no additional intervention (very low certainty evidence). Overall, this means that ranking the effectiveness of interventions for depression in terms of their benefits or harm to enable us to recommend one treatment over another is inappropriate, because of the uncertainty in evidence. However, there is some indication that behavioural interventions may provide some benefit for depression in autistic children when compared to offering no intervention.

##### Depression – change scores

None of the trials reported change scores for depression in children.

#### Depression in adults

##### Proportion of participants with depression

None of the trials reported proportion of participants with a diagnosis of depression.

##### Depression – final scores

A random-effects model was used as it was more conservative, although the model fit statistics were similar in the fixed-effect and random-effects models. The between-study variance was 0.29 (95% CrI: 0.00 to 14.29). The effect estimates are shown in Table 2. There was no evidence of differences in final depression scores in any of the comparisons in the NMA (very low certainty evidence). However, in the direct comparisons, self-directed adapted CBT and individual skills training resulted in lower depression scores than no additional intervention (low certainty evidence). This means that ranking interventions in terms of their benefits or harm are inappropriate because of the uncertainty in evidence. However, there is some indication that self-directed CBT adapted for autistic people, or individual skills training, may provide some benefit for depression in autistic adults, when compared to offering no intervention.

##### Depression – change scores

Only one trial reported change in depression scores, and this compared oxytocin with no additional intervention. There was no evidence of difference in the change in depression scores (standardised mean difference (SMD) 0.25; 95% CrI: –0.38 to 0.87; one trial; 40 participants; very low certainty evidence).

### Other mental health–related outcomes

In addition to our main outcomes of depression and anxiety, we also decided to report findings from a commonly reported secondary outcome, quality of life, as this was highlighted as being of particular importance to autistic people during our focus group work. We differentiate between overall quality of life scores and mental health–related quality of life as these were reported separately in the RCTs. We also report the outcome of self-harm, the only other mental health–related outcome reported in the studies.

### Quality of life

#### Quality of life in children

Two trials reported quality of life (Hospital & Health, 2007; National Library of Medicine, 2013b). As shown in Supplemental Table 7, there was no evidence of differences in any of the comparisons in the direct comparisons or NMA (very low certainty evidence). We therefore conclude that there is no evidence that these interventions improve quality of life in autistic children.

#### Quality of life in adults

##### Final scores

Only one trial (A. Russell et al., 2019) reported quality of life scores at final follow-up, and this compared self-directed CBT with no additional intervention. Scores were higher (indicative of better quality of life) in the self-directed adapted CBT group (SMD: 0.87; 95% CrI: 0.26 to 1.48; one trial; 48 participants; very low certainty evidence).

##### Change in quality of life

Two trials reported change in quality of life. Only direct comparisons were performed because of the nature of the comparisons. The first trial reported oxytocin versus no additional intervention. There was no evidence of differences in change in quality of life between these two groups (SMD: 0.12; 95% CrI: –0.51 to 0.74; 1 trial; 40 participants; very low certainty evidence). The second trial reported group activity versus CBT adapted for autistic people. There was no evidence of differences in quality of life between these two groups (SMD: –0.39; 95% CrI: –0.92 to 0.14; one trial; 55 participants; very low certainty evidence). Overall, we cannot recommend any particular intervention to improve quality of life in autistic adults.

#### Mental health–related quality of life in adults

One study looked at mental health–related quality of life in autistic adults. This study compared self-directed CBT with no additional intervention and showed no difference in mental health–related quality of life scores (SMD: 4.34; 95% CrI: –2.14 to 10.74; one trial; 48 participants; very low certainty evidence).

### Self-harm

Only one study reported self-harm as an outcome, and this looked at opioid receptor antagonist versus no additional intervention. There was no evidence of a difference in the proportion of participants who self-harmed between participants in the two intervention groups (0.48; 95% CrI: 0.12 to 1.71; one trial; 41 participants; control group proportion: 61.1%; very low certainty evidence).

### Adverse events

Here, we report the adverse events (harms) that resulted from different interventions. In terms of adverse events, we report these using the classifications used by the study authors, in terms of serious, non-serious, or all adverse events (i.e. no differentiation reported between those which may be serious or non-serious). We retained these categories as we recognise that clinicians may wish to differentiate between the severity of adverse events when considering the risks and tolerability of a given intervention.

#### Serious adverse events

Among the trials that reported serious adverse events, in seven trials (227 participants), there were no serious adverse events in both arms (Danforth et al., 2018; National Library of Medicine, 2011, 2013a, 2013b; A. Russell et al., 2019; Storch et al., 2013, 2015), and in one trial (46 participants), there were zero-events in one of the arms, which prevented the calculation of effect estimates (Potter et al., 2019). In the remaining trial (Dean et al., 2017), there was no evidence of difference in the proportion of participants who developed serious adverse events between *N*-acetyl cysteine and no additional intervention (1.05; 95% CrI: 0.03 to 41.06; one trial; 98 participants; control group proportion 2.0%; very low certainty evidence). Since each participant in this trial developed only one serious adverse event, we did not calculate the effect estimates for the number of serious adverse events. Overall, this means that no interventions showed strong evidence of serious harm to participants.

#### Non-serious adverse events

Among the trials that reported non-serious adverse events, in three trials (108 participants), there were no non-serious adverse events in both arms (National Library of Medicine, 2013a; Storch et al., 2013, 2015). In two trials (42 participants), where participants received MDMA or NaSSA (noradrenergic and specific serotonergic antidepressants), all participants developed non-serious adverse events (Danforth et al., 2018; National Library of Medicine, 2011). The proportion of participants in the remaining two trials who developed non-serious adverse events (in the no additional intervention group) was 39.4%. As there was only one study for each comparison, only the fixed-effect model was applicable. The effect estimates are shown in Supplemental Table 7. There was no evidence of differences in non-serious adverse events in any of the direct comparisons or NMA (very low certainty evidence).

The number of non-serious adverse events in the no additional intervention group was 2.5 events per participant in the only trials that reported the number of non-serious adverse events per participant (Danforth et al., 2018). The number of non-serious adverse events was higher in the MDMA group than no additional intervention (2.30; 95% CrI: 1.21 to 4.86; one trial; 12 participants; control group event rate: 2.5 events per participant; very low certainty evidence). Overall, this means that ranking interventions in terms of their likelihood of causing non-serious adverse events were inappropriate because of the uncertainty of evidence. In one trial that compared MDMA with no additional intervention, there were more adverse events in participants who received MDMA.

#### Any adverse events

##### Proportion of people who developed any adverse events

Nine trials (337 participants) reported the proportion of participants who developed any adverse events (Campbell et al., 1993; Danforth et al., 2018; National Library of Medicine, 2012, 2013a, 2013b; Reddihough et al., 2019; A. Russell et al., 2019; Storch et al., 2013, 2015). A total of 10 interventions were compared in these trials. Among the trials that reported the proportion of participants who developed any adverse events, in three trials, there were no adverse events in both arms (National Library of Medicine, 2013a; Storch et al., 2013, 2015), and in one trial, all the participants in the intervention group (MDMA) developed adverse events (Danforth et al., 2018). We did not calculate the effect estimates in these trials. In the remaining five trials (262 participants), the proportion of participants who developed any adverse events in the no additional intervention group was 50.0%. As there was only one study for each comparison, only the fixed-effect model was applicable.

The effect estimates are shown in Supplemental Table 7. There was no evidence of differences in the proportion of people who developed any adverse events in any of the comparisons in the direct comparisons or NMA (very low certainty evidence). Overall, this means that ranking interventions in terms of their likelihood of participants experiencing adverse events were inappropriate because of the uncertainty in evidence.

##### Number of adverse events

The mean number of events in the no additional intervention group was 1.8 events per participant in the trials that reported the number of adverse events per participant. A random-effects model was used as it was more conservative, although the model fit statistics were similar in the fixed-effects and random-effects models. The between-study variance was 6.31 (95% CrI: 0.02 to 23.79).

The effect estimates are available in Supplemental Table 7. As shown in the direct comparisons and NMA, several medications increased the number of ‘any’ adverse events (very low certainty evidence). Overall, this means that these medications were likely to increase the number of adverse events experienced by participants.

### Assessment of reporting biases

We performed a thorough search of literature including searching trial registers. Therefore, we identified most published studies registered in the clinical trial registers. There was no meaningful way in which to order these studies (i.e. there was no specific change in the risk of bias in the studies, sample size, or the control group used over time). This meant that we were unable to perform the comparison-adjusted funnel plot, which would have enabled us to assess publication bias. Important mental health outcomes were not reported in many trials: some of these measures were subscales of other measures reported by authors but were not reported. A total of 608 other trials that assessed these interventions (mentioned above) in autistic people did not report mental health outcomes. A detailed breakdown of interventions used in these studies can be found in Supplemental Table 11. Even in those trials where mental health outcomes were reported, only a small proportion reported adverse events adequately, indicating reporting biases.

### Exploratory analysis

We performed an exploratory metaregression to determine whether an intervention’s effect on anxiety and depression could be explained by its effect on core features of autism. This was because trialists often targeted interventions towards reducing core features of autism. There was no evidence that an intervention’s effect on core features of autism could predict its effect on anxiety or depression scores (Supplemental Appendix 6).

## Discussion

### Summary

The aim of this review was to compare relative benefits and harms of different interventions to improve the mental health of autistic people, via a systematic review and NMA of RCTs, and to identify research gaps. Our focus was on anxiety and depression, as well as broader mental health outcomes. We included RCTs irrespective of the interventions being investigated. This is the first NMA on the impact of different interventions on mental health conditions in autistic people.

To summarise our main findings, few trials specifically studied mental health conditions in autistic people, and those that existed were at high risk of bias. The risk of bias assessment highlighted low study quality, small sample sizes resulting in insufficient statistical power, a lack of blinding of participants and researchers, few RCTs comparing different interventions, and potential conflicts of interest based on the source of funding (e.g. industry-funded studies of medications, or studies funded by organisations with an emphasis on ‘curing’ autism). It is worth noting that some indices (e.g. blinding of participants) may be less appropriate to assess some interventions (e.g. blinding those involved in delivering the intervention as well as study participants as to whether they received CBT or not is not feasible), suggesting an overestimate of the seriousness of the situation. Yet, conflicts of interest, for example, are often underreported in autism research, and as such, our risk of bias assessment may be an underestimate of the situation (Bottema-Beutel et al., 2021b). Overall, the certainty of evidence that interventions can improve the mental health of autistic people was very low.

In addition to the aforementioned issues with risk of bias, our review also highlights other sources of bias around the representativeness of the samples included. For example, our review showed a common issue in autism research, namely that most trials only included autistic people without ID or included only a small number of autistic participants with ID (G. Russell et al., 2019). No trials included only autistic participants with ID, or reported mental health outcomes in a subset of autistic people with ID. This was not the only sampling issue identified. For example, in terms of mental health, of the 71 studies included in the review, 31 included autistic people with mental health diagnoses, 38 did not assess whether participants had any mental health conditions, and 2 excluded people with mental health conditions. Therefore, the findings of this review should be interpreted with caution, as many studies did not assess the benefits or harms of the interventions for autistic people with mental health conditions.

In terms of harms, adverse effects were examined in our review. Adverse events are likely to occur in a proportion of people receiving any intervention. However, in line with a recent review of autism intervention research, harms are often not reported adequately by researchers (Bottema-Beutel et al., 2021a). Failure to warn people of the potential for adverse events related to psychological therapy interventions may increase the risk of adverse events such as increased self-harm or suicidal ideation (Britton et al., 2021; Dawson & Fletcher-Watson, 2022; Papaioannou et al., 2021). It is essential that any intervention studies report adverse events so that both the benefits and risks of interventions can be appraised, and people being offered a given intervention can give truly informed consent. An issue related to adverse events is the acceptability of interventions (psychological interventions or medications) for autistic people: these should also be assessed and reported in the studies.

Although this was not a focus of our analysis, it is also important to note issues associated with the measurement of mental health outcomes in our review. Existing literature demonstrates that there is a lack of robust and reproducible evidence to support the idea that the outcome measures used in trials in this review reliably assess the effectiveness of interventions (Cassidy et al., 2018; Wigham & McConachie, 2014). Linked to this, there is paucity of research on the smallest change in the intervention outcome of importance to an individual autistic person (otherwise known as minimally important differences; MID) (Chatham et al., 2018). There is, therefore, difficulty in understanding the implications of decreased mental health scores for autistic people and whether these constitute a clinically significant change.

This issue may be particularly problematic in autism research. We included any RCTs in autistic people where anxiety or depression was measured. Our review includes a high number of studies that are based on an individualistic model, which suggests that autistic people themselves need to change. This is a result of the evidence base and nature of the literature at present; many interventions included in our review were targeted at reducing core autistic features rather than targeting mental health conditions. Yet we showed that an intervention’s effect on core features of autism does not predict its effect on the mental health of autistic people. Although, historically, studies have been conducted on autistic people with the aim of fundamentally changing them (i.e. making them less autistic), intervention studies that focus on changing core features of autism have become increasingly critiqued (Bradley et al., 2015; Hoekstra et al., 2018). Recent work highlights some of the difficulties that autistic people can experience in differentiating aspects of their experience that relate to autism and aspects that relate to mental health (Crane et al., 2019). Differentiating these elements of autistic people’s experiences and providing interventions that do not aim to change the core of autistic people, but focus on separate and co-occurring mental health conditions, are key to ensuring that their mental health needs are met (Crane et al., 2019).

Overall, the aforementioned methodological issues demonstrate that the evidence base for mental health interventions in autistic people is poor. However, we can give some tentative recommendations as to interventions that may be useful starting points for further study. In line with existing literature, we found that some forms of CBT may improve health-related quality of life in some autistic children and may decrease anxiety and depression scores in some autistic children and adults, but further research is necessary (Perihan et al., 2020; Tseng et al., 2020; Vasa et al., 2014; Weston et al., 2016). At present, due to limitations associated with the quality of evidence, we are unable to agree with researchers who concluded that CBT is an effective intervention for all autistic people (Lang et al., 2010; Ung et al., 2015).

Furthermore, in line with existing literature, our review found that mindfulness therapy may decrease anxiety and depression scores in some autistic adults with previous mental health conditions (Cachia et al., 2016; Hartley et al., 2019; Menezes et al., 2020). However, as per a previous review on this topic, our review found there is low certainty of evidence. Lack of reporting around potential harms and meditation-related side effects is problematic in light of the fact that a large proportion of participants in mindfulness-based interventions can experience negative side effects (Britton et al., 2021), even if they benefit from the intervention overall (Cachia et al., 2016; Hartley et al., 2019). Existing evidence regarding the effectiveness of behavioural interventions for depression in autistic people is limited (Menezes et al., 2020; White et al., 2018). Our review provides some indication that behavioural interventions may provide some benefit for depression in autistic children when compared to offering no intervention.

In terms of pharmacological interventions, this review found that some medications (ADH, MDMA, SNRI) were associated with increased adverse events, although there is no evidence these medications improve mental health. This is in line with existing meta-analyses that suggest that the evidence for the use of antidepressants or anti-anxiety medications in autistic people is inconsistent (D’Alò et al., 2021; Deb et al., 2021), and antipsychotics are not effective for anxiety or depression (D’Alò et al., 2021).

Notably, there were considerable variations in the way that interventions were administered across studies. This included variations in who delivered the intervention (e.g. CBT could be self-directed, parent-delivered, family-based, or specialist-delivered), whether it was delivered as an individual therapy or group therapy, and in terms of the frequency and duration of the intervention. We considered major variations as separate interventions and calculated the relative effects of these variations. However, different variations were better for different outcomes, for example, group CBT adapted for autistic people may be effective for anxiety in children, but there is no evidence it is effective for depression in children. This introduces uncertainty in how the intervention should be delivered, especially as anxiety and depression commonly co-occur (Hranov, 2007; Melton et al., 2016). Therefore, on the basis of currently available evidence, in contrast to existing work (e.g. Chancel et al., 2020), we cannot recommend a specific modality of CBT over another. As noted previously, and in line with existing literature (for exceptions, see Selles et al., 2015; White et al., 2015), most trials did not assess the effects of interventions over a long time period. Therefore, we cannot assess whether effects of interventions such as CBT would persist over time, or whether further booster sessions may be required in future.

### Clinical recommendations

Overall, the reviewed evidence indicates considerable uncertainty about the effects of different interventions for mental health conditions in autistic people. Our results suggest that some forms of CBT and mindfulness therapy may be useful to treat mental health conditions in some autistic people. In contrast, the routine use of interventions to manage core features of autism with a view to improve mental health conditions of autistic people should be avoided. Indeed, in line with existing literature regarding problems with the use of existing outcome measures validated with non-autistic people (Cassidy et al., 2018; Wigham & McConachie, 2014), we recommend that mental health interventions should focus specifically on autistic mental health rather than on outcomes that have not been validated in this population, or outcomes that aim to reduce core features of autism. We did not, however, systematically review whether the effects of interventions in autistic people are different from those in non-autistic people. We also do not have strong evidence from this systematic review to suggest that specific interventions are likely to work for autistic people with mental health conditions (as the studies included in the review tended to focus on autistic people without a diagnosed mental health condition).

It is imperative that future research seeks to overcome the methodological limitations highlighted in this review, to facilitate the development of a sound evidence base upon which to make clinical recommendations. Until such evidence is available, we recommend that autistic people are given full access to mental health interventions that are available to non-autistic people. Refusal to offer interventions until further evidence has been obtained would mean that autistic people lack access to mental health support, and the risks of this need to be balanced against potential harms from trying interventions (Hallett & Crompton, 2018). We have not investigated person-centred care in this review. However, based on the wider ethical principles (Santana et al., 2018), we recommend that clinicians follow the key principles of person-centred care when supporting the mental health needs of autistic people. This includes discussing the pros and cons of trying any intervention, monitoring risk of harm (both short and long term) relating to any intervention offered and taking into account the acceptability of a given intervention to each individual (Smith & Williams, 2016).

## Supplemental Material

sj-docx-1-aut-10.1177_13623613221117931 – Supplemental material for Benefits and harms of interventions to improve anxiety, depression, and other mental health outcomes for autistic people: A systematic review and network meta-analysis of randomised controlled trials Click here for additional data file. Supplemental material, sj-docx-1-aut-10.1177_13623613221117931 for Benefits and harms of interventions to improve anxiety, depression, and other mental health outcomes for autistic people: A systematic review and network meta-analysis of randomised controlled trials by Audrey Linden, Lawrence Best, Freya Elise, Danielle Roberts, Aoife Branagan, Yong Boon Ernest Tay, Laura Crane, James Cusack, Brian Davidson, Ian Davidson, Caroline Hearst, William Mandy, Dheeraj Rai, Edward Smith and Kurinchi Gurusamy in Autism

## Notes

1. For a more detailed examination of findings, network plots (where applicable) are available in Supplemental Figure 2. Data and unprocessed data analysis are available in Supplemental Appendix 3 and Supplemental Appendix 4. Model fit (when network meta-analysis (NMA) was performed) is available in Supplemental Table 6. Effect estimates (when NMA was performed) is available in Supplemental Table 7. The certainty of evidence for anxiety and depression are available from the ‘Summary of Findings’ table (Table 2). Abbreviations used for interventions are available in Table 3. Forest plots for anxiety and depression are available in Figures 2 and 3. Detailed results including forest plots for all analysed outcomes are available in the Supplement.
