# Supplementary material for: Benefits and harms of interventions to improve anxiety, depression, and other mental health outcomes for autistic people: A systematic review and network meta-analysis of randomised controlled trials
Source: Autism. 2022 Aug 11;27(1):7–30. doi: 10.1177/13623613221117931 (PMC9806485; doi:10.1177/13623613221117931)
Supplement: sj-docx-1-aut-10.1177_13623613221117931 – Supplemental material for Benefits and harms of interventions to improve anxiety, depression, and other mental health outcomes for autistic people: A systematic review and network meta-analysis of randomised controlled trials [file sj-docx-1-aut-10.1177_13623613221117931.docx]

# Interventions to improve mental health in people with Autism Spectrum: a systematic review and network meta-analysis

Table of Contents

[Interventions to improve mental health in people with Autism Spectrum: a systematic review and network meta-analysis 1](#_Toc80959594)

[Abstract 5](#_Toc80959595)

[Background 5](#_Toc80959596)

[Objectives 5](#_Toc80959597)

[Methods 5](#_Toc80959598)

[Key findings 5](#_Toc80959599)

[Outputs, impact, dissemination 5](#_Toc80959600)

[Conclusions 5](#_Toc80959601)

[Future plans 5](#_Toc80959602)

[Plain language summary 7](#_Toc80959603)

[Aims and objectives 7](#_Toc80959604)

[Background 7](#_Toc80959605)

[Methods 7](#_Toc80959606)

[Key findings 7](#_Toc80959607)

[Dissemination, outputs and impact 7](#_Toc80959608)

[Patient and public involvement 7](#_Toc80959609)

[Conclusions and future plans 7](#_Toc80959610)

[Background and rationale 8](#_Toc80959611)

[Autism Spectrum Disorder 8](#_Toc80959612)

[Socio-economic impact of autism 8](#_Toc80959613)

[Importance of mental health in autistic people 8](#_Toc80959614)

[Review of existing evidence 9](#_Toc80959615)

[Why is this research important? 9](#_Toc80959616)

[Aims and objectives 11](#_Toc80959617)

[Primary objective 11](#_Toc80959618)

[Secondary objectives 11](#_Toc80959619)

[Methods 12](#_Toc80959620)

[Criteria for considering studies for this review 12](#_Toc80959621)

[Outcomes 13](#_Toc80959622)

[Search methods for identification of studies 13](#_Toc80959623)

[Data collection 14](#_Toc80959624)

[Data synthesis 14](#_Toc80959625)

[Dealing with missing data 15](#_Toc80959626)

[Assessment and investigation of heterogeneity and inconsistency 15](#_Toc80959627)

[Assessment of reporting biases 16](#_Toc80959628)

[Reporting 17](#_Toc80959629)

[Recommendations for clinical practice and future research 17](#_Toc80959630)

[Results 18](#_Toc80959631)

[Results of search 18](#_Toc80959632)

[Characteristics of included studies 18](#_Toc80959633)

[Risk of bias in the trials 18](#_Toc80959634)

[Effect estimates 18](#_Toc80959635)

[Subgroup analysis 24](#_Toc80959636)

[Sensitivity analysis 26](#_Toc80959637)

[Assessment of reporting biases 26](#_Toc80959638)

[Exploratory analysis 27](#_Toc80959639)

[Discussion 28](#_Toc80959640)

[Funding 34](#_Toc80959641)

[Conflicts of interest 34](#_Toc80959642)

[Acknowledgements 34](#_Toc80959643)

[Contribution of authors 34](#_Toc80959644)

[Deviations from protocol 35](#_Toc80959645)

[Tables 36](#_Toc80959646)

[Table 1: Characteristics of included studies (ordered by comparisons) 36](#_Toc80959647)

[Table 2A: Reasons for exclusion of studies 44](#_Toc80959648)

[Table 2B: Additional reports of included and excluded studies 51](#_Toc80959649)

[Table 3: List of randomised controlled trials in which mental health outcomes were not measured at all and list of randomised controlled trials in which mental health outcomes were measured but not reported at all or not reported in an analysable format 53](#_Toc80959650)

[Table 4 Risk of bias (ordered by comparisons) 58](#_Toc80959651)

[Table 5 Summary of outcomes, included studies and included interventions 65](#_Toc80959652)

[Table 6 Fit statistics for fixed-effect, random-effects, and inconsistency model 68](#_Toc80959653)

[Table 7 Effect estimates 71](#_Toc80959654)

[Table 8 Summary of findings (Certainty of evidence) 71](#_Toc80959655)

[Table 9 Anxiety scales used 102](#_Toc80959656)

[Table 10 Depression scales used 116](#_Toc80959657)

[Table 11 Assessment of reporting bias 119](#_Toc80959658)

[Figures 122](#_Toc80959659)

[Figure 1 Reference flow 122](#_Toc80959660)

[Figure 2 Network plots 122](#_Toc80959661)

[Figure 3 Inconsistency factor plot 130](#_Toc80959662)

[Figure 4 Forest plots 130](#_Toc80959663)

[Appendices 143](#_Toc80959664)

[Appendix 1 Search strategy 143](#_Toc80959665)

[Appendix 2 Abbreviations 148](#_Toc80959666)

[Appendix 3 Data 150](#_Toc80959667)

[Appendix 4 Data analysis (unprocessed) 150](#_Toc80959668)

[Appendix 5 Sensitivity analysis 150](#_Toc80959669)

[Appendix 6 Results of metaregression 151](#_Toc80959670)

[Appendix 7 List of ongoing randomised controlled trials with mental health outcomes 152](#_Toc80959671)

[Appendix 8 References for excluded studies 155](#_Toc80959672)

[Appendix 9 References for included studies 262](#_Toc80959673)

[References 267](#_Toc80959674)

## Abstract

### Background

There is currently no high-quality network meta-analysis of effectiveness of different interventions for improving mental health in autistic people.

### Objectives

Our objectives were to compare the relative benefits and harms of different interventions to improve mental health by a systematic review of randomised controlled trials (RCTs) and network meta-analysis and to identify research gaps.

### Methods

We followed the PRISMA 2009 guidance. We searched MEDLINE, EMBASE, Cochrane library, PsycINFO, CINAHL Plus, Science Citation Index, and trial registers until 17th October 2020, and reference lists of included trials and related systematic reviews. We assessed the risk of bias in the trials using Cochrane risk-of-bias tool for randomized trials (RoB 2). We performed the network meta-analysis using Bayesian methods and calculated the effect estimates and the 95% credible intervals. We used the fixed-effect or random-effects model and reported the more conservative model.

PROSPERO registration ID: CRD42019136093

### Key findings

We included a total of 71 randomised clinical trials (3630 participants) in the review. All the trials had some concerns about bias or were at high risk of bias. The follow-up period in the trials ranged from 1 month to 24 months. During this short follow-up period, some forms of cognitive behavioural therapy improved health-related quality of life in autistic children and decreased anxiety and depression scores in autistic children and adults; mindfulness therapy decreased anxiety and depression scores in adults with previous mental health problems; and many medications increased adverse events. The implications of the decreased anxiety and depression scores are unclear as there is uncertainty about the measurement properties of the outcome measures used in the trials.

### Outputs, impact, dissemination

We have submitted this NIHR report and presented the work in Autistica conference. We plan to submit the work to journals, present in national and international conferences, and communicate the findings to psychiatrists and psychologists with the help of the research team. We will also contact other groups representing autistic people and their carers to help with dissemination of information.

### Conclusions

The evidence indicates considerable uncertainty about the effects of the different interventions in decreasing mental health problems in autistic people. Some forms of cognitive behavioural therapy and mindfulness therapy may be useful to prevent or treat mental ill-health in autistic people. Use of medications routinely to manage core features of autism should be avoided. There is a need for better understanding of how mental ill-health should be measured in autistic people and when the intervention can be considered effective.

### Future plans

We want to highlight to healthcare professionals and UK parliament that use of medications routinely to manage core features of autism should be avoided. We also want to develop and evaluate variations in cognitive behavioural therapy and mindfulness therapy. We also want to perform research related to better interpretation of the findings of this project.

## Plain language summary

### Aims and objectives

Our aim was to look at the benefits and harms of different interventions on mental health outcomes in autistic people.

### Background

Nearly three out of four autistic people experience mental health problems such as stress, anxiety or depression. The research already done does not guide us on how best to prevent or treat mental health problems for autistic people.

### Methods

We searched all the published and unpublished randomised controlled trials about interventions for mental health problems in autistic people until 17^th^ October 2020. We then combined the information from all these trials using advanced statistical methods to analyse how good the interventions are.

### Key findings

Seventy-one studies (3630 participants) provided information for this research. The studies reported how participants were responding to the intervention for only a short period of time. The trials did not report which interventions worked for people with intellectual disability. In people without intellectual disability, some forms of cognitive behavioural therapy and mindfulness therapy may be helpful. However, further research is necessary. Many trials used medications to target core features of autism rather than targeting mental health conditions, but these medications did not help autistic people.

### Dissemination, outputs and impact

We plan to widely disseminate the findings to healthcare professionals through medical journals and conferences and contact other groups representing autistic people.

### Patient and public involvement

This project was led by a researcher with autism and history of poor mental health. He was supported by other members of the autistic community and charitable sector and by healthcare and education professionals with and without special interests in autism.

### Conclusions and future plans

Medications to target core features of autism should be avoided. We plan to conduct further research on how best to deliver cognitive behavioural therapy and find its impact on autistic people.

## Background and rationale

### Autism Spectrum Disorder

Autism is diagnosed on the basis of persistent difficulties in social communication and interaction, as well as the presence of restricted and repetitive patterns of behaviour, interests, or activities[1]. The prevalence of autism varies according to the different definitions used and is approximately 0.1% to 2% in different countries (1% in the UK and other western countries)[2-4]. Approximately 25% to 30% of autistic people have intellectual disability (intelligent quotient (IQ) ≤70), and another 25% of autistic people have borderline intellectual ability (IQ 71-85)[5, 6].

Although autism is not a mental health condition, approximately 14% to 50% of autistic people have a current or previous history of depression[7-9] and 40% to 80% have a current or previous history of anxiety disorders[10].There is some uncertainty about the reason for this high incidence of mental health conditions in autistic people. However, in general, autistic people have a greater prevalence of negative life events such as unemployment, which are linked to lower quality of life than non-autistic people [11-13] and may be related to the prevalence of mental health problems. Anxiety may be associated with sensory hyper-responsivity (which is very common in autism), although it is not clear whether sensory hyper-responsivity leads to anxiety or whether anxiety leads to sensory hyper-responsivity or whether they both co-exist due to factors such as intolerance of uncertainty[14, 15] or hitherto unrecognised risk factors[16, 17]. Symptoms of depression may be mediated by being a victim of bullying which may be linked to atypical social skills in autistic people[18]. Autistic people may also have post-traumatic stress disorder (PTSD) with similar or higher prevalence than general population (median prevalence: 2.8%)[19], perhaps linked to being a victim of abuse (including bullying) and assault[19]. The prevalence of psychotic disorders such as schizophrenia and mood disorders such as bipolar disorder is also higher in young autistic people than in young people without an autism diagnosis[20]. Overall, about 80% of young autistic adults have experienced one or more mental health problems[11].

### Socio-economic impact of autism

In a study performed in 2013, the annual costs of supporting people with autism were estimated to be £3 billion in children and £25 billion in adults in UK[21]. The lifetime costs for autistic people without intellectual disability (ID) were £0.9 million per individual and were £1.5 million per individual for autistic people with additional ID[22]. This socio-economic impact may stem from childhood experiences, with the school years (for many with autism) being characterised by lack of understanding of autism by others, social isolation, being a victim of bullying, and failure to meet learning support needs[8, 23]. This may lead to increasing social isolation, unemployment, and poor mental health in adulthood[8, 23-26].

### Importance of mental health in autistic people

Autistic people without ID are four times more likely to have suicidal thoughts than people in the general population[27]: about two-thirds of autistic people have suicidal thoughts and a third of autistic people have made suicidal plans or attempts[27]. Autistic people are seven times more likely (nine times for autism without ID) to die of suicide compared to general population[28]. Overall, autistic people have about 2.5 times higher all-cause premature mortality than general population[22] and their average lifespan is decreased by about 16 years[28], which may be related to poor mental health

Autistic people are also less likely to be employed than non-autistic people[29]: only 16% of autistic adults with a diagnosis are in full-time work and only 32% are in any kind of paid work. Fewer autistic people are employed than people with physical disability[30]. Among autistic people, those with poor mental health are less likely to be employed than those without poor mental health[29]. Unemployment and underemployment are likely to worsen the mental health of autistic people[23], resulting in a vicious cycle of unemployment and poor mental health and leading to suicidal thoughts, plans, attempts, and premature deaths in autistic people.

### Review of existing evidence

#### Interventions used to address mental health conditions in autistic people

Some of the interventions used in the RCTs that measured mental health outcomes in autistic people include drugs such as sympatholytics, antidepressants, hypnotic anxiolytics, and antipsychotics; psychological therapies such as cognitive behavioural therapy, counselling, and mindfulness-based therapy; behavioural interventions such as applied behavioural analysis, and social skills training; and other therapies such as music therapy, and waitlist (i.e. no additional intervention until the outcome is measured)[31-40]. Although many of these trials have focussed on autistic features as primary outcomes, a significant proportion of these trials also reported on mental health outcomes.

#### Existing systematic reviews

Prior to this review, there were no high-quality systematic reviews that compared the effectiveness of different interventions for improving mental health in autistic people. At the time of the start of the project, while there were several Cochrane systematic reviews and other systematic reviews including autistic people as participants[40-55]: mental health outcomes were reported in only four reviews[40, 43, 54, 55]. Only one of these systematic reviews included more than one intervention comparison[54] and this limited the language to English articles and included evidence from non-randomised studies during the interpretation of results[54]. Only eight randomised controlled trials (RCTs) were included in this review[54].

Network meta-analysis (NMA) allows for a combination of direct and indirect evidence and the ranking of different interventions for different outcomes[56, 57]. It also usually results in more precise estimates than direct or indirect evidence in isolation[58, 59]. When people need to decide between more than two competing interventions, network meta-analysis provides an analysis option and provides information for comparisons between pairs of interventions that have never been evaluated within individual RCTs[60]. However, none of the existing systematic reviews have attempted NMA. This is the first research project to perform a NMA on interventions to reduce mental health problems in autistic people.

### Why is this research important?

Improving the mental health of autistic people through interventions informed by the best evidence could lead to improvements in their overall health-related quality of life (HRQoL) and has the potential to decrease pre-mature mortality[61] and unemployment[62]. Better mental health is also likely to reduce the significant costs of health and care services for autistic people[62]. Identifying interventions that improve the mental health of autistic people is the top research priority, as identified in the James-Lind Alliance priority setting partnership (JLA-PSP) that involved autistic people, their families, and the professionals involved in their care[63].

It is important to note that interventions that improve mental health in non-autistic people or individual autistic people may not be effective for all autistic people[64]. Therefore, one cannot extrapolate the effectiveness of interventions in non-autistic people to autistic people. While each autistic individual is different and may respond to interventions differently (as do non-autistic individuals), it is important to understand how likely it is that an intervention will work, so that informed decision about which intervention to start can be made. It is also important that, whenever possible, this information about the relative effects of different interventions is obtained from randomised controlled trials (RCT), which ensure similar type of participants receive the compared interventions: this would overcome the problem of outcome differences due to differences in the type of people who received them. Therefore, research which includes evidence from RCTs including only autistic people (or reporting outcomes separately in autistic people) is important to address the significant uncertainty about the relative benefits and harms of different interventions designed to improve mental health in autistic people.

## Aims and objectives

### Primary objective

An initial search of the literature showed that there was insufficient literature to conduct a network meta-analysis on autistic adults, and people with ID. Therefore, our primary objective was to compare the relative benefits and harms of different interventions to improve mental health in autistic children and adolescents without ID by a systematic review and network meta-analysis.

### Secondary objectives

Our secondary objectives were to conduct a systematic review and meta-analyses on the benefits and harms of interventions to improve mental health in autistic adults; and to identify research gaps, particularly in autistic adults without ID and autistic people of any age with ID.

## Methods

The protocol was registered in PROSPERO database prior to project commencement. The PROSPERO registration ID is CRD42019136093. We conducted and report the systematic review according to Preferred Reporting Items for Systematic Reviews and Meta-Analyses (PRISMA) statement (2009) and its extension for NMA[65, 66].

### Criteria for considering studies for this review

#### Type of studies

We included all RCTs regardless of the publication status, year of publication, and language of publication.

#### Setting

Community, primary care, secondary care

#### Types of participants

Autistic people regardless of their age, symptom severity, and intelligence. Separate meta-analyses were planned for children and adolescents without ID, children and adolescents with ID, adults with ID, and adults without ID. We stratified the analysis based on ID, because some interventions, for example CBT, require an ability to understand language and concepts that may not be possible for some people with ID.

#### Types of interventions

We included any of the following interventions for comparison with one another either alone or in combination.

- Drugs
  - Sympatholytics
  - Antidepressants (e.g., selective serotonin reuptake inhibitors (SSRIs), serotonin and norepinephrine reuptake inhibitors (SNRIs), Noradrenergic and specific serotonergic antidepressants (NaSSAs), monoamine oxidase inhibitors (MAOIs), tricyclic antidepressants (TCAs) and atypical antidepressants).
  - Hypnotic anxiolytics
  - Antipsychotics
  - Antioxidants
  - Other medications such as oxytocin, anti-diuretic hormone
- Psychological therapies
  - Cognitive behavioural therapy
  - Mindfulness-based therapy
  - Counselling
- Behavioural therapies
  - Social skills training
  - Early intensive behavioural intervention (including 'Applied Behavioural Analysis')
- Miscellaneous interventions
  - Music therapy
  - Equine therapy
  - Wait-list (i.e., no additional intervention or placebo intervention until measurement of the outcomes).

We considered other interventions, such as parent education or dietary supplements, where mental health in autistic people was assessed and included them.

### Outcomes

We assessed the benefits and harms of the different interventions by assessing their effect on the following outcomes. The list of outcomes and the relative importance of the outcomes was revised following advisory group meetings involving autistic people, carers, and autistic and non-autistic healthcare and education professionals. The major revisions were accepting anxiety and depression however measured (not just as proportion of those with a diagnosis) as primary measure, addition of self-harm, any adverse events, and meaningful life activities as secondary measures, and removing the overall improvement in core features of autism as a secondary measure. This was because of the patient and public engagement activity, in which participants emphasised the need to include self-harm and meaningful life activities as important to them and did not want core features of autism as an outcome measure which devalued them.

#### Primary outcomes

1. Anxiety or depression (however measured using validated measures or as proportion)
2. Overall health-related quality of life (HRQoL) using any validated measure (this will allow us to assess the effect intervention on overall health-related quality of life)
3. Serious adverse events (as defined by authors)

#### Secondary outcomes

1. Mental health-related quality of life
2. Self-harm
3. Suicidal thoughts or attempted suicide
4. Non-serious adverse events (as defined by authors)
5. Any adverse events (as defined by authors)
6. Psychotic symptoms using any validated measure
7. Post-traumatic stress disorder using any validated measure
8. Employment status
9. Meaningful life activities
10. All-cause mortality (same justification for inclusion as employment status).

All outcomes were collected until maximal follow-up. For the characteristics of included studies, we have presented only data from studies which reported mental health outcomes in an analysable format (or where we could obtain such data by contacting the study authors). However, we have summarised information on the number of studies in whom mental health outcomes were not measured or were not reported in an analysable format for each included comparison (to provide an indication of reporting biases), and the overall number of studies in whom mental health outcomes were not measured or reported to provide an indication of the opportunity lost in measuring the outcomes that are most important to autistic people.

### Search methods for identification of studies

#### Electronic searches

We searched the Cochrane Central Register of Controlled Trials (CENTRAL) in the Cochrane Library, MEDLINE (OvidSP), Embase (OvidSP), PsycINFO (OvidSP), CINAHL Plus (EBSCO), and Science Citation Index Expanded (Web of Knowledge) from inception to 17^th^ October 2020 for RCTs without applying any language restrictions. We searched for all possible comparisons formed by the interventions of interest. To identify further ongoing or completed trials, we also searched ClinicalTrials.gov and the World Health Organization International Clinical Trials Registry Platform (apps.who.int/trialsearch/), which searches various trial registers, including ISRCTN and ClinicalTrials.gov. For the complete search strategy, please see Appendix 1.

#### Other resources

We searched the references of the identified trials and the existing systematic reviews on autism and mental health interventions to identify additional trials for inclusion. We also contacted the study authors to identify further trials and obtain aggregate data from unpublished studies.

### Data collection

#### Selection of studies

Two review authors from the review author team independently identified trials for inclusion by screening the titles and abstracts and sought full-text articles for any references identified by at least one of the review authors for potential inclusion. We selected trials for inclusion based on the full-text articles (after translation if required). We documented the process to enable completion of the PRISMA flow-chart. We resolved any discrepancies through discussion and arbitration.

#### Obtain data

Two review authors independently extracted the following data (after translation, if required) using a pre-piloted data extraction form.

1. Outcome definition/scale and data (in appropriate format for analysis)
2. Data on potential effect modifiers
3. Participant characteristics such as age, sex, intelligence, presence of anxiety or depression at baseline
4. Details of the intervention and control (including dose, frequency, and duration)
5. Length of follow-up
6. Information to assess risk of bias
7. Other data
   1. Year and language of publication
   2. Country in which the participants were recruited
   3. Year(s) in which the trial was conducted
   4. Inclusion and exclusion criteria

#### Assessment of risk of bias in included studies

We used the Cochrane risk-of-bias tool for randomized trials (RoB 2.0)[67] for assessment of risk of bias.

### Data synthesis

#### Methods for indirect and mixed comparisons

We conducted NMA on all outcomes with multiple intervention comparisons. We obtained a network plot to understand the network geometry and ensure that the trials are connected by interventions using Stata/SE 15[68]. For comparisons not connected to the network, we performed direct pairwise meta-analysis only. We conducted a Bayesian network meta-analysis using the Markov chain Monte Carlo method in OpenBUGS 3.2.3 as per guidance from the National Institute for Health and Care Excellence (NICE) Decision Support Unit (DSU) documents[69] using study-level data and appropriate likelihood and link functions. We used 'wait-list, treatment-as-usual, or placebo' as the reference group ('no additional intervention'). The codes we used for analysis accounted for the correlation between the effect sizes from studies with more than two groups.

For binary outcomes (proportion of people with anxiety or depression, serious adverse events and non-serious adverse events), we calculated the odds ratio (OR) with 95% credible interval (CrI)[70]. For continuous outcomes (overall health-related quality of life, anxiety, depression, psychotic symptoms, post-traumatic stress disorder, core features of autism), we calculated or planned to calculate the standardised mean difference (SMD) with 95% Crl when possible. For count outcomes (number of serious adverse events and non-serious adverse events), we calculated the rate ratio (RaR) with 95% Crl. For time-to-event data (employment status and all-cause mortality at maximal follow-up), we planned to calculate hazard ratio (HR) with 95% Crl.

We performed the meta-analysis using a fixed-effect model and random-effects model and reported the more conservative model (treatment effect is smaller compared to the other model).

We used a hierarchical Bayesian model using ‘vague’ or ‘flat’ priors and three different initial values (to ensure convergence of values), employing codes provided by NICE DSU[69], and using technical details similar to those of the lead-author’s previous network meta-analyses[71-73]. If we did not obtain convergence, we increased the number of simulations for the 'burn-in'. If we still did not obtain convergence, we planned to use alternate priors and initial values[74]. We estimated the probability that each intervention ranks at one of the possible positions using the NICE DSU codes[69]. We did not present the surface under the cumulative ranking curve (SUCRA) (cumulative probability) and rankogram[56, 68] because of the uncertainty in the ranking for the outcomes.

#### Direct comparison

Whenever interventions have been compared directly, we performed the direct comparisons using the same codes and the same technical details.

### Dealing with missing data

We performed an intention-to-treat analysis whenever possible[75]; otherwise, we used the data available to us. We conducted best-worst case and worst-best case scenario analyses as sensitivity analyses for binary outcomes whenever possible.

For continuous outcomes, although we planned to impute the mean and/or standard deviation from median and P values according to guidance in the Cochrane Handbook if the data appear to be normally distributed, we were unable to make judgments on the distribution of data; therefore, we did not impute these data[76].

### Assessment and investigation of heterogeneity and inconsistency

#### Heterogeneity

We assessed clinical and methodological heterogeneity by carefully examining the characteristics and design of included trials. We avoided two major sources of clinical heterogeneity by performing separate meta-analyses based on age and ID. Other potential sources of clinical heterogeneity included age of participants within children and adolescents (preschool children versus adolescents versus other children), pre-existing anxiety or depression (previous mental health problems), variations in the interventions, different definitions used for outcomes, different scales used for assessment of outcomes, and period of follow-up (short-term follow-up ≤ 1 year versus medium to long-term follow-up (> 1 year)).

#### Dealing with diverse range of outcome measures

We encountered four types of diverse range of outcome measures of mental health. A brief description is as follows.

- Different aspects of mental health were reported – for example, anxiety versus psychosis: we analysed the data separately for each type of mental disorder.
- An aspect of mental health was reported as mean and standard deviation using different scales: we converted the available data to standardised mean difference and the standard error and used the codes for combining ‘intervention differences’.
- An aspect of mental health was self-reported in some trials and informant (carer)-reported in some trials: we considered this as a source of heterogeneity and addressed this by metaregression and subgroup analysis when meaningful, i.e., multiple studies were included in the network meta-analysis for different intervention comparisons, whilst modelling different ways of reporting (e.g. self- versus informant-report).
- An aspect of mental health was reported as continuous data in some studies and as binary data in others (i.e., whether or not a mental health outcome is present). Although we planned to convert the binary outcomes into standardised mean difference and then combine the standardised mean difference as above, we did not perform this as the mental health outcomes were reported as continuous outcomes in most studies. Therefore, we analysed the continuous data separately from binary data.

We investigated heterogeneity through subgroup analyses and meta-regression using methods and codes described in the NICE DSU documents[77]. If the 95% credible intervals of the interaction term do not overlap zero, we considered this as presence of subgroup differences.

We assessed statistical heterogeneity by comparing the results of the fixed-effect model meta-analysis and the random-effects model meta-analysis and calculating the between-study standard deviation (tau^2^)[78] and NMA-specific I^2^[79]. If we identified substantial, clinical, methodological, or statistical heterogeneity, we explored and addressed the heterogeneity in subgroup analysis as mentioned above.

#### Inconsistency

We evaluated the plausibility of transitivity assumption (the assumption that any participant that meets the inclusion criteria is, in principle, equally likely to be randomised to any of the above eligible interventions[56]) by looking at the inclusion and exclusion criteria in the studies. This also requires that information on potential effect-modifiers such as the presence of anxiety or depression are similar across comparisons. If there was any concern about the transitivity assumption, we planned to perform separate meta-analysis for people with and without anxiety or depression.

We assessed inconsistency (statistical evidence of the violation of transitivity assumption) by fitting both an inconsistency model and a consistency model, when direct and indirect evidence was available. We used inconsistency models employed in the NICE DSU manual, as we used a common between-study standard deviation[80]. In addition, we used design-by-treatment full interaction model and inconsistency factor (IF) plots to assess inconsistency[68, 81]. If there was evidence of inconsistency, we planned to identify areas in the network where substantial inconsistency might be present in terms of clinical and methodological diversities between trials and, when appropriate, limit network meta-analysis to a more compatible subset of trials.

#### Sensitivity analysis

In addition to the best-worst case scenario and worst-best case scenario sensitivity analyses mentioned above, we planned to perform a sensitivity analysis excluding the trials in which mean or standard deviation or both were imputed and use different imputed standard deviations.

### Assessment of reporting biases

For the NMA, we planned to perform a comparison-adjusted funnel plot. However, there was no meaningful way in which to rank these studies (i.e. there was no specific change in the risk of bias in the studies, sample size, or the control group used over time), we judged this reporting bias by the completeness of the search[68] (i.e., identify completed but unpublished trials from the trial registry for which we are unable to obtain data from the study authors). Therefore, we have assessed the reporting bias by the completeness of the search and absence of reporting of results.

### Reporting

We presented the effect estimates with 95% CrI for each pairwise comparison calculated from the direct comparisons and network meta-analysis. We planned to present the rankograms and SUCRA but did not do this because of the considerable uncertainty in the ranks[57, 81].

We presented 'Summary of findings' tables for each of the primary and secondary outcomes using methods described by GRADE Working Group[82] as guidance.

### Recommendations for clinical practice and future research

We gathered information on the relative importance of the outcomes by discussions between autistic people and researchers. We also plan to discuss the findings of the research with policy makers.

We have also provided recommendations for future research in the population, intervention, control, outcomes, period of follow-up, and study design based on the uncertainties that we identified from the existing research by presenting the results of the systematic review to an advisory group meeting (the author team) involving patients and carers, clinicians, methodologists, statisticians, academic researchers in this field, and people with experience in NIHR funding panels.

## Results

### Results of search

We identified 13794 records through electronic searches of the Cochrane Central Register of Controlled Trials (CENTRAL) in the Cochrane Library (Wiley) (n = 1949), MEDLINE Ovid (n = 3354), Embase Ovid (n = 2004), PsycInfo (n = 2091), CINAHL Plus (n = 1015), Science Citation Index Expanded and Conference Proceedings Citation Index-Science (n = 1354), ClinicalTrials.gov (n = 289), and WHO Trials register (n = 1738). After removing duplicates, there were 10809 records. We excluded 8704 clearly irrelevant records through reading titles and abstracts. We retrieved a total of 2105 full text records for further assessment in detail. We included a total of 71 trials (Table 1). We excluded 1636 records for the reasons stated in the 'Table 2A'. Additional reports of included and excluded studies (349 records) are listed in Table 2B. Forty-nine records were records of ongoing trials with mental health outcomes measured. RCTs in which mental health outcomes were not measured at all and list of randomised controlled trials in which mental health outcomes were measured but not reported at all or not reported in an analysable format are listed in Table 3. The reference flow is shown in Figure 1.

### Characteristics of included studies

The characteristics of included studies are summarised in Table 1. The abbreviations used in Table 1 and other tables are available in Appendix 2. We included a total of 71 trials (3630 participants) in this review. Of these, 387 participants were excluded after randomisation for various reasons, leaving a total of 3243 participants included in one or more mental health outcomes. The sample sizes in the trials varied from 11 participants to 223 participants. Only six trials had sample sizes of 100 or more participants [33, 83-87]. The follow-up period in the trials ranged from 1 month to 24 months. Only one trial had a follow-up of more than 12 months [88].

Overall, most trials included only people without intellectual disability or included only a small number of participants with intellectual disability. None of the trials included only participants with intellectual disability or reported the mental health outcomes in the subset of people with intellectual disability.

### Risk of bias in the trials

The risk of bias in the trials is summarised in Table 4. All the trials some concerns about bias or were at high risk of bias in at least one of the domains and were considered to have some concerns about bias or were at high risk of bias overall.

### Effect estimates

A summary of the outcomes and trials and interventions included for the outcome are available in Table 5. The network plots where applicable are available in Figure 2. The inconsistency factor plot for anxiety scores in children (the only outcome when there was closed loop) is available in Figure 3. The data and unprocessed data analysis are available in appendices 3 and 4. The model fit when network meta-analysis was performed is available in Table 6. The effect estimates when network meta-analysis was performed is available in Table 7. The certainty of evidence is available from the 'Summary of Findings' table (Table 8). The forest plots are available in Figure 4.

#### Anxiety

A total of 69 different anxiety scales were used in the trials. The anxiety scales used in the trials and their interpretations are shown in Table 9.

##### Children

###### Proportion of participants with anxiety

Four trials (78 participants) reported the proportion of participants with anxiety [35, 36, 88, 89]. A total of six interventions (counselling, family-based adapted CBT, individual adapted CBT, MASSI skills training, parent psychoeducation, and no additional intervention) were compared in these trials. In two trials, all participants in the no additional intervention group had anxiety [35, 89]. Therefore, they could not be included in the calculations of odds ratios as they are equivalent to zero event trials. In the remaining two trials, the interventions were not connected to the network [36, 88]. Therefore, only direct comparisons were performed.

The proportion of participants with anxiety was lower in participants in whom parents received psychoeducation than in those whose parents received no additional intervention (0.15; 95% CrI 0.02 to 0.87; 1 trial; 24 participants; control group proportion: 76.9%; very low certainty evidence). There was no evidence of difference in proportion of participants with anxiety between counselling and MASSI skills training (0.32; 95% CrI 0.05 to 1.61; 1 trial; 32 participants; control group proportion: 80%; very low certainty evidence).

###### Anxiety scores

Final scores

Forty-four trials (1966 participants) reported anxiety final scores [33, 36, 37, 84, 86, 88, 90-121]. A total of 32 interventions (ABA, ABA plus diuretic, anti-diuretic hormone analogue, book reading, counselling, distraction, family-based adapted CBT, family-based exposure-focussed CBT, family-based non-adapted CBT, group activity, group adapted CBT, group non-adapted CBT, group skills training, individual adapted CBT, individual non-adapted CBT, MASSI skills training, N-acetyl cysteine, oxytocin, parent psychoeducation, parent-mediated adapted CBT, PEERS group skills training, self-directed non-adapted CBT, self-directed skills training, SENSE group skills training, sensory integration therapy, sensory integration therapy plus Thai traditional massage, skills training, SNRI, SSRI, video skills training, video skills training plus distraction, and no additional intervention) were compared in these trials. Two trials were not included in the network meta-analysis as the interventions were unconnected [92, 106]. The remaining 42 trials (1831 participants) were included in the network meta-analysis. Random-effects model was used as it had better model fit than fixed-effect model. The between study variance was 0.75 (95% CrI 0.31 to 1.87). There was no evidence of inconsistency according to the inconsistency model fit, treatment-by-design (95% CrI 0.00 to 15.93), and inconsistency factor plot.

The effect estimates are shown in Table 7. As indicated in table 7, there were several comparisons in which participants had lower or higher scores in one intervention than another intervention (very low certainty evidence). Overall, many CBT interventions had lower anxiety scores compared to no additional interventions and other interventions such as anti-diuretic hormone analogues.

In the comparisons not connected to the network, there were no evidence of differences between ABA plus diuretic versus ABA (SMD -0.34; 95% CrI -0.87 to 0.19; 1 trial; 55 participants; very low certainty evidence) or between sensory integration therapy plus Thai traditional massage versus sensory integration therapy (SMD -0.20; 95% CrI -0.71 to 0.31; 1 trial; 60 participants; very low certainty evidence).

Change scores

Twelve trials (804 participants) reported anxiety change scores [83, 84, 86, 90, 115, 118, 122-127]. A total of 12 interventions (anti-diuretic hormone analogue, dietary supplement, family-based adapted CBT, family-based non-adapted CBT, group adapted CBT, individual adapted CBT, individual non-adapted CBT, individual non-adapted CBT plus melatonin, melatonin, NaSSA, SSRI, and no additional intervention) were compared in these trials. All the trials were connected to the network. Random-effects model was used as it was more conservative although the model fit statistics was similar in the fixed-effects and random-effects model. The between study variance was 1.26 (95% CrI 0.22 to 11.66). Since there were no closed loops, inconsistency was not checked.

The effect estimates are shown in Table 7. As indicated in table 7, there was no evidence of differences in the change in anxiety scores in any of the comparisons in the network meta-analysis (very low certainty evidence), although in the direct comparisons, some CBTs resulted in lower anxiety scores than no additional intervention (very low certainty evidence).

##### Adults

###### Proportion of participants with anxiety

None of the trials reported the proportion of adult participants with anxiety.

###### Anxiety scores

Final scores

Thirteen trials (526 participants) reported final anxiety scores in adults [38, 87, 128-138]. A total of 12 interventions (anti-diuretic hormone analogue, atypical antipsychotic, group adapted CBT, group skills training, individual skills training, MDMA, mindfulness, oxytocin, self-directed adapted CBT, self-directed non-adapted CBT, skills training, and no additional intervention) were compared in these trials. All the trials were connected to the network. Random-effects model was used as it was more conservative although the model fit statistics was similar in the fixed-effects and random-effects model. The between study variance was 0.10 (95% CrI 0.00 to 4.15). Since there were no closed loops, inconsistency was not checked.

The effect estimates are shown in Table 7. As indicated in table 7, there was no evidence of differences in the final anxiety scores in any of the comparisons in the network meta-analysis (very low certainty evidence), although in the direct comparisons, some CBTs resulted in lower anxiety scores than no additional intervention (low certainty evidence).

Change scores

Two trials (121 participants) reported change in anxiety scores in adults [87, 131]. A total of 3 interventions (anti-diuretic hormone analogue, oxytocin, and no additional intervention) were compared in these trials. Both the trials were connected to the network. As there was only one study for each comparison, only the fixed-effect model was applicable. Since there were no closed loops, inconsistency was not checked.

The effect estimates are shown in Table 7. As indicated in table 7, there was no evidence of differences in the change in anxiety scores in any of the comparisons in the direct comparisons or network meta-analysis (very low certainty evidence).

#### Depression

A total of 16 different depressions scales were used in the trials. The depression scales used in the trials and their interpretations are shown in Table 10.

##### Children

###### Proportion of participants with depression

None of the trials reported proportion of participants with depression.

###### Scores

Final scores

Seven trials (231 participants) reported finals scores for depression [102, 104, 105, 139-142]. A total of 6 interventions (ABA, group adapted CBT, individual CBT, PEERS group skills training, skills training, and no additional intervention) were compared in these trials. All the trials were connected to the network. Random-effects model was used as it was more conservative although the model fit statistics was similar in the fixed-effects and random-effects model. The between study variance was 0.87 (95% CrI 0.01 to 17.93). Since there were no closed loops, inconsistency was not checked.

The effect estimates are shown in Table 7. As indicated in table 7, there was no evidence of differences in the final depression scores in any of the comparisons in the network meta-analysis (very low certainty evidence), although in the direct comparisons, ABA resulted in lower depression scores than no additional intervention (very low certainty evidence).

Change scores

None of the trials reported change scores for depression in children.

##### Adults

###### Proportion of participants with depression

None of the trials reported proportion of participants with depression.

###### Scores

Final scores

Ten trials (448 participants) reported final depressions scores [38, 87, 128, 129, 134, 136-138, 143, 144]. A total of 10 interventions (atypical antipsychotic, group activity, group adapted CBT, individual skills training, mindfulness, oxytocin, self-directed adapted CBT, self-directed non-adapted CBT, skills training, and no additional intervention) were compared in these trials. All the trials were connected to the network. Random-effects model was used as it was more conservative although the model fit statistics was similar in the fixed-effects and random-effects model. The between study variance was 0.29 (95% CrI 0.00 to 14.29). Since there were no closed loops, inconsistency was not checked.

The effect estimates are shown in Table 7. As indicated in table 7, there was no evidence of differences in the final depression scores in any of the comparisons in the network meta-analysis (very low certainty evidence), although in the direct comparisons, self-directed adapted CBT and individual skills training resulted in lower depression scores than no additional intervention (low certainty evidence).

Change scores

One trial (40 participants) reported change in depression scores in adults [135]. There was no evidence of difference in the change in depressions scores between oxytocin and no additional intervention (SMD 0.25; 95% CrI -0.38 to 0.87; 1 trial; 40 participants; very low certainty evidence).

##### Proportion of participants with anxiety or depression

None of the trials reported proportion of participants with anxiety or depression.

#### Quality of life

##### Children

Two trials (87 participants) reported in quality of life [91, 145]. In both the trials, the scale used to measure quality of life was Pediatric Quality of Life Inventory. A total of 3 interventions (anti-diuretic hormone analogue, SNRI, and no additional intervention) were compared in these trials. Both trials were connected to the network. As shown in Table 7, there was no evidence of differences in any of the comparisons in the direct comparisons or network meta-analysis (very low certainty evidence).

###### Change in quality of life

None of the trials reported change in quality of life.

##### Adults

One trial (48 participants) reported quality of life [134]. The scale used to measure the quality of life was EQ-5D. The quality of life was better in self-directed adapted CBT than no additional intervention (SMD 0.87; 95% CrI 0.26 to 1.48; 1 trial; 48 participants; very low certainty evidence).

###### Change in quality of life

Two trials (95 participants) reported change in quality of life [135, 143]. The scales used to measure quality of life were Quality of Life Inventory [143] and WHO-QOL [135]. A total of 4 interventions (group activity, group adapted CBT, oxytocin, and no additional intervention) were compared in these trials. The interventions were not connected to the network; so only direct comparisons were performed. There was no evidence of differences in change in quality of life between oxytocin versus no additional intervention (SMD 0.12; 95% CrI -0.51 to 0.74; 1 trial; 40 participants; very low certainty evidence) or between group activity versus group adapted CBT (SMD -0.39; 95% CrI -0.92 to 0.14; 1 trial; 55 participants; very low certainty evidence).

#### Serious adverse events

Nine trials (328 participants) reported serious adverse events [33, 97, 107, 118, 123, 124, 132, 134, 145]. A total of 10 interventions were compared in these trials. In seven trials (227 participants), there no serious adverse events in both arms [97, 107, 123, 124, 132, 134, 145]; in one trial (46 participants), there was zero-event in one of the arms [118]. We did not calculate the effect estimates for these trials because of zero events. In the remaining trial [33], there was no evidence of difference in the proportion of participants who developed serious adverse events between N-acetyl cysteine and no additional intervention (1.05; 95% CrI 0.03 to 41.06; 1 trial; 98 participants; control group proportion 2.0%; very low certainty evidence). Since each participant in this trial developed only one serious adverse event, we did not calculate the effect estimates for the number of serious adverse events.

#### Mental health-related quality of life

##### Children

None of the trials reported mental health-related quality of life in children.

##### Adults

One trial (48 participants) reported mental health-related quality of life [134]. There was no evidence of difference in mental health-related quality of life between self-directed adapted CBT and no additional intervention (MD 4.34; 95% CrI -2.14 to 10.74; 1 trial; 48 participants; very low certainty evidence).

#### Self harm

##### Proportion of patients with self-harm

One trial (41 participants) reported the proportion of participants who self-harmed [146]. There was no evidence of difference in the proportion of participants who self-harmed between opioid receptor antagonist and no additional intervention (0.48; 95% CrI 0.12 to 1.71; 1 trial; 41 participants; control group proportion: 61.1%; very low certainty evidence).

##### Proportion of patients with suicidal thoughts or attempted suicide

None of the trials reported the proportion of patients with suicidal thoughts or attempted suicide.

#### Non-serious adverse events

Seven trials (183 participants) reported non-serious adverse events (number of people) [97, 107, 123, 124, 132, 134, 145]. A total of 8 interventions (anti-diuretic hormone analogue, family-based adapted CBT, family-based non-adapted CBT, individual adapted CBT, MDMA, NaSSA, self-directed adapted CBT, and no additional intervention) were compared in these trials. Three trials (108 participants) were not connected to the network because they had zero-events in both arms [97, 107, 124]; In two trials (42 participants), all participants who received MDMA or NaSSA developed non-serious adverse events [123, 132]. The proportion of participants who developed non-serious adverse events in the no additional intervention group in the remaining two trials was 39.4%. As there was only one study for each comparison, only the fixed-effect model was applicable. Since there were no closed loops, inconsistency was not checked.

The effect estimates are shown in Table 7. As indicated in the table 7, there was no evidence of differences in There was no evidence of differences in the non-serious adverse events in any of the direct comparisons or network meta-analysis (very low certainty evidence).

One trial (12 participants) reported non-serious adverse events (number of events) [132]. Each participant in the no additional intervention group was 2.5 events per participant. The number of adverse events was higher in MDMA than no additional intervention (2.30; 95% CrI 1.21 to 4.86; 1 trial; 12 participants; control group event rate: 2.5 events per participant; very low certainty evidence).

#### Any adverse events

##### Proportion of people who developed any adverse events

Nine trials (337 participants) reported the proportion of participants who developed any adverse events [86, 96, 97, 107, 124, 132, 134, 145, 146]. A total of 10 interventions (anti-diuretic hormone analogue, family-based adapted CBT, family-based non-adapted CBT, individual adapted CBT, MDMA, opioid receptor antagonist, oxytocin, self-directed adapted CBT, SSRI, and no additional intervention) were compared in these trials. Three trials were not connected to the network because they had zero-events in both arms [97, 107, 124]; one trial was not connected to the network because all the participants in the intervention group (MDMA) developed adverse events [132] (which is equivalent to zero-event trials while calculating odds ratios). We did not calculate the effect estimates in these trials because of zero-events. The remaining five trials (262 participants) were included in the network meta-analysis. The proportion of participants who developed any adverse events in the no additional intervention group was 50.0% in the studies included in network meta-analysis. As there was only one study for each comparison, only the fixed-effect model was applicable. Since there were no closed loops, inconsistency was not checked.

The effect estimates are shown in Table 7. As indicated in table 7, there was no evidence of differences in the change in anxiety scores in any of the comparisons in the direct comparisons or network meta-analysis (very low certainty evidence).

##### Number of adverse events

Eight trials (568 participants) reported the number of any adverse events [33, 37, 86, 87, 118, 127, 132, 146]. A total of 8 interventions (anti-diuretic hormone analogue, MDMA, N-acetyl cysteine, opioid receptor antagonist, oxytocin, SNRI, SSRI, and no additional intervention) were compared in these trials. The number of events in the no additional intervention group was 1.8 events per participant. All the trials were connected to the network. Random-effects model was used as it was more conservative although the model fit statistics was similar in the fixed-effects and random-effects model. The between study variance was 6.31 (95% CrI 0.02 to 23.79). Since there were no closed loops, inconsistency was not checked.

The effect estimates are available in Table 7. As shown in the direct comparisons and network meta-analysis several medications increased the number of 'any' adverse events (very low certainty evidence).

#### Psychotic symptoms

None of the trials reported the psychotic symptoms.

#### Post-traumatic stress disorder

None of the trials reported post-traumatic stress disorder.

#### Employment status

None of the trials reported the employment status of participants.

#### Meaningful life activities

None of the trials reported the proportion of people having meaningful life activities.

#### Proportion of people who died

One trial (20 participants) reported proportion of people who died [145]. There were no deaths in any of the participants in this trial. In 17 other trials (777 participants) without post-randomisation drop-outs, we can infer that there were no deaths [35, 36, 85, 92, 94, 100, 107-109, 112, 115, 124, 127, 132, 135, 136, 139].

### Subgroup analysis

The only outcomes with sufficient data for subgroup analysis were the anxiety and depression scores. Of the planned subgroup analysis and metaregression, the only subgroup analyses and metaregression performed related to history of previous mental health problems and the person reporting the outcome (self-reported, parent-reported, clinician-reported, or teacher reported). This was only possible for anxiety and depression scores.

The remaining planned subgroup analyses and metaregression were not performed for the following reasons.

- Different definitions/scales used for outcomes: the trialists used different scales; therefore, we could only perform the analysis using standardised mean difference. It would have been meaningless to analyse each scale separately because of the paucity of data related to each different scale.
- Period of follow-up: Only one of the trials had a follow-up of more than 12 months [88].

#### Previous mental health problems

The effect estimates in a subset of people with previous mental health problems for anxiety and depression scores is provided in Table 7. A formal subgroup analysis was not possible as many studies did not report whether participants had previous mental health problems or did not report the data separately for participants with and without previous mental health problems.

##### Anxiety scores

###### Final scores (Children)

Twenty trials (813 participants) reported anxiety scores in children with previous mental health problems [35, 36, 84, 89, 90, 95, 97, 100, 101, 103, 107, 110, 112, 114, 116, 117, 120, 121, 124, 147]. A total of 16 interventions (counselling, distraction, family-based adapted CBT, family-based exposure-focussed CBT, family-based non-adapted CBT, group activity, group adapted CBT, individual adapted CBT, individual non-adapted CBT, MASSI skills training, parent-mediated adapted CBT, self-directed non-adapted CBT, self-directed skills training, video skills training, video skills training plus distraction, and no additional intervention) were compared in these trials. All the trials were connected to the network. Random-effects model was used as it was more conservative although the model fit statistics was similar in the fixed-effects and random-effects model. The between study variance was 1.46 (95% CrI 0.45 to 5.95). Since there were no closed loops, inconsistency was not checked.

The effect estimates are available in Table 7. As indicated in table 7, some forms of CBT resulted in lower anxiety scores in direct comparisons and network meta-analysis.

###### Change scores (children)

Five trials (305 participants) reported change in anxiety scores in children with mental health problems [84, 90, 122-124]. A total of 7 interventions (family-based adapted CBT, family-based non-adapted CBT, group adapted CBT, individual adapted CBT, individual non-adapted CBT, NaSSA, and no additional intervention) were compared in these trials. All the trials were connected to the network. As there was only one study for each comparison, only the fixed-effect model was applicable. Since there were no closed loops, inconsistency was not checked.

The effect estimates are available in Table 7. As indicated in table 7, some forms of CBT resulted in greater reduction in anxiety scores in direct comparisons and network meta-analysis.

###### Final scores (adults)

Four trials (149 participants) reported final scores in adults with mentall health problems [38, 129, 132, 134]. A total of 5 interventions (group adapted CBT, MDMA, mindfulness, self-directed adapted CBT, and no additional intervention) were compared in these trials. All the trials were connected to the network. As there was only one study for each comparison, only the fixed-effect model was applicable. Since there were no closed loops, inconsistency was not checked.

The effect estimates are available in Table 7. As indicated in table 7, mindfulness and some forms of CBT resulted in lower anxiety scores in direct comparisons and network meta-analysis.

###### Change scores (adults)

None of the trials reported change in anxiety scores in adults with previous history of mental health problems.

##### Depression scores

###### Final scores (children)

One trial (13 participants) reported final depression scores in children with previous mental health problems. There was no evidence of difference in the final depression scores between group adapted CBT and no additional intervention (SMD 0.65; 95% CrI -0.50 to 1.80; 1 trial; 13 participants; very low certainty evidence).

###### Change scores (children)

None of the trials reported change in depression scores in children with previous history of mental health problems.

###### Final scores (adults)

Three trials (137 participants) reported depression scores in adults with previous history of mental health problems [38, 129, 134]. A total of 4 interventions (group adapted CBT, mindfulness, self-directed adapted CBT, and no additional intervention) were compared in these trials. All the trials were connected to the network. As there was only one study for each comparison, only the fixed-effect model was applicable. Since there were no closed loops, inconsistency was not checked.

The effect estimates are available in Table 7. As indicated in table 7, mindfulness and some forms of CBT resulted in lower depression scores in direct comparisons and network meta-analysis.

###### Change scores (adults)

None of the trials reported change in depression scores in adults with previous history of mental health problems.

#### Effect of person who reports the measures on the effect estimates

The subgroup analysis based on who reported the outcome measures did not reveal any subgroup differences.

##### Anxiety

- Final scores (children): The interaction factor was -0.12 (95% CrI -1.07 to 0.81).
- Change scores (children): The interaction factor was 0.88 (95% CrI -0.53 to 2.49).
- Final scores (adults): The interaction factor was 0.29 (95% CrI -3.10 to 3.72).
- Change scores (adults): Both trials under this outcome used clinician-reported outcome measures.

##### Depression

- Final scores (children): Convergence could not be obtained, likely to be due to sparse data
- Change scores (children): None of the trials reported this outcome
- Final scores (adults): The interaction factor was 0.39 (95% CrI -9.42 to 10.22).
- Change scores (adults): Both trials under this outcome used self-reported outcome measures.

### Sensitivity analysis

#### 'Best-worst' and 'worst-best' scenario analyses

We performed the 'best-worst' and 'worst-best' scenario analyses for the sensitivity analysis related to missing outcome data for the only outcomes with binomial distribution. This sensitivity analysis revealed that the results for proportion of participants with any adverse events changed depending upon the scenario used for analysis (Appendix 5). Therefore, this outcome is susceptible to attrition bias. The remaining outcomes did not reveal any alterations in results and robust to attrition bias.

#### Imputation of standard deviation

We did not perform any imputation of standard deviation.

### Assessment of reporting biases

We performed a thorough search of literature including search of the trial registers. Therefore, we identified most of the published or studies registered in the clinical trials register. Since there was no meaningful way in which to order these studies (i.e., there was no specific change in the risk of bias in the studies, sample size, or the control group used over time), we were unable to perform the comparison-adjusted funnel plot. Important mental health outcomes were not reported in many trials: some of these measures are subscales of other measures reported by authors but were not reported. We performed a thorough search of literature including the trial registers, which also revealed that 608 other trials which assessed these interventions in autistic people did not report mental health outcomes. A detailed breakdown of the interventions used in these studies can be found in Table 11.

### Exploratory analysis

We performed an exploratory analysis to find if the differences in the effects of the interventions on anxiety and depression could be explained by differences in their effects on core features of autism (often stated as ‘overall autistic symptoms’ by study authors and measured using a variety of scales such as the Autism Diagnostic Interview-Revised (ADI-R) or Autism Diagnostic Observation Schedule (ADOS)). We performed a metaregression in order to identify this. There was no evidence that differences in core features of autism could predict differences in anxiety or depression scores. The results of metaregression are available in Appendix 6 for anxiety final scores (children), anxiety change scores (children), anxiety final scores (adults), and depression final scores (children). This exploratory analysis was not possible for the remaining outcomes because of paucity of data.

## Discussion

#### Summary of main results

We performed a systematic review and network meta-analysis of the interventions to improve mental health in autistic people. We included a total of 71 trials (3630 participants) in this review. Of these, 387 participants were excluded after randomisation for various reasons, leaving a total of 3243 participants included in one or more mental health outcomes. The follow-up period in the trials ranged from 1 month to 24 months. Only one trial had a follow-up of more than 12 months [88].

During this follow-up period, some forms of CBT may improve health-related quality of life in some autistic children, and decrease anxiety and depression scores in some autistic children and adults; mindfulness therapy may decrease anxiety and depression scores in some adults with previous mental health problems. Many interventions were targeted at reducing core autistic features. Attempting to reduce autistic features may result in autistic people increasing use of camouflaging to attempt to hide autistic features, which is associated with increased mental health problems including anxiety, depression, and suicidality[148-151]. Medications were associated with increased adverse events, although there is no evidence that these medications, which are targeted at core features of autism, improve mental health.

As one of our patient representatives noted, there is anecdotal evidence that CBT is aversive for some autistic people. Therefore, interventions that improve mental health in many autistic people on average may not be effective for some autistic people.

It is important for the results of this review to be interpreted with caution. Of the several systematic reviews about the measurement properties of outcome measures used in autistic people[152-158], only two systematic reviews related to measurement properties of mental health outcomes[154, 158]. Both these systematic reviews highlighted the lack of robust and reproducible evidence to support that the outcome measures used in trials reliably assess the effectiveness of interventions. As a result, the studies we included used a variety of mental-health related outcome measures that have not been validated in autistic people.

There is paucity of research on the minimally important differences (MID) in autism. We were able to identify only one study related to MID in autism and this did not relate to a mental health outcome[159]. A recent systematic review of MIDs did not reveal any study related to MID in autism[160]. We also searched the PROMID (<https://promid.mcmaster.ca/>) and did not identify any study related to MID in autism. Other researchers have also highlighted the paucity of validated tools to assess mood in autistic people[161]. Therefore, there is major difficulty in understanding the implications of these decreased scores to autistic people.

#### Overall completeness and applicability of evidence

Of the 71 studies included in the review, 31 studies included autistic people with mental health diagnoses, 38 did not assess whether participants had any mental health problems, and two excluded people with mental health problems. Therefore, the findings of this review should be interpreted with caution, as many studies did not assess the benefits or harms of the interventions for autistic people with mental health problems.

Our review highlights a common issue in autism research, namely that most trials included people without intellectual disability or included only a small number of participants with intellectual disability[162]. None of the trials included only participants with intellectual disability or reported the mental health outcomes in the subset of people with intellectual disability. Therefore, the findings of this review are applicable only in people without intellectual disability.

There were considerable variations in the way that the intervention was administered across studies. This included variations in who delivered the intervention (for example, CBT could be self-directed, parent-delivered, family-based, or specialist-delivered), whether it was delivered as an individual therapy or group therapy, frequency of the intervention, and the duration of the intervention. We considered major variations as separate interventions and calculated the relative effects of these variations. However, different variations were better for different outcomes, for example, adapted group CBT seems to be effective for anxiety in children, but there is no evidence that it is effective for depression in children. This introduces uncertainty in how the intervention should be delivered. Therefore, we cannot recommend that the intervention such as CBT is delivered using a specific modality.

As noted previously, most trials had short follow up periods, and did not assess the effects of interventions over a longer time period. Therefore, we cannot assess whether the effects of interventions such as CBT would persist over time, or whether further booster sessions may be required in future. Since there is uncertainty over how much benefit is experienced from the changes in mental health scores, and whether the effects of interventions persist over time, it is not possible to assess cost effectiveness of different interventions at this time.

#### Certainty of the evidence

The overall certainty of evidence was very low for all outcomes and comparisons. One of the main reasons for this was the risk of bias in the trials: there were some concerns or high risk of bias in all the trials. There was evidence of statistical heterogeneity in some outcomes, which is another reason for decreasing the certainty of evidence. Another major reason for the decreased certainty of evidence was imprecision. The sample sizes were small in most trials, and many were insufficiently powered for studying the mental health outcomes reported. Only six trials had sample sizes of 100 or more participants [33, 83-87]. This introduces imprecision. We used clinical outcomes; therefore, there is no issue of indirectness due to outcomes. There was no suggestion that the potential effect modifiers were systematically different across comparisons (i.e., there was no concern about the transitivity assumption) for most outcomes. However, we were unable to perform a formal analysis to assess this for most outcomes because of sparse data. Therefore, one cannot rule out inconsistency ('incoherence' according to GRADE terminology). There was no meaningful way to order these studies (i.e., there was no specific change in the risk of bias in the studies, sample size, or the control group used over time); we have completed a thorough search for studies on effectiveness. However, many trials which assessed these interventions in autistic people did not report mental health outcomes (Table 11). This may suggest reporting bias for these outcomes.

#### Potential biases in the review process

We selected a range of databases to search without using any language restrictions and conducted the network meta‐analysis according to NICE DSU guidance. In addition, we have analysed using the fixed‐effects model and random‐effects model and assessed and reported inconsistency whenever possible. These are the strengths of the review process.

There were several deviations from the protocol. Firstly, we conducted a focus group for identifying the relative importance of the outcomes prior to the data extraction rather than after completing the review. This meant that the relative importance was based on the importance to autistic people rather than availability of the information; therefore, this is more robust than our original plan in terms of finding out how important the outcome was for autistic people. Secondly, we accepted anxiety and depression however measured (not just as proportion) as primary measure. We noted that majority of the trialists reported this outcome domain as continuous measures using validated scales. As these scales measure the same outcome domain, we have included the scales in the same outcome domain as a primary outcome measure. Therefore, we do not consider this has resulted in bias in our review. Thirdly, we have added self-harm and meaningful life activities as secondary measures and removed the overall improvement in core autistic features as a secondary measure. This was because of the focus group activity in which participants emphasised the need to include self-harm and meaningful life activities as these outcomes were important to them and considered core features of autism to be an outcome measure which devalued them. As the main purpose of the review was to identify the benefits (and harms) of intervention to autistic people, the above changes in the outcomes do not lead to bias. Another deviation from protocol was that we added any adverse events as one of the outcomes. This was because many trialists did not report serious and non-serious adverse events separately. This has not led to any change in conclusions. Lastly, we performed an exploratory metaregression to find if the differences in anxiety and depression could be explained by core features of autism. The metagression results suggested that there is no evidence that the effects of interventions on mental health can be explained by the effect of interventions on overall core features of autism. While this is an important finding in terms of how future trials should be designed, this was not a planned analysis.

#### Agreements and disagreements with other studies or reviews

This is the first network meta-analysis on the impact of different interventions in reducing mental health problems in autistic people. We identified several systematic reviews related to interventions for autism. Despite the differences in methodology (in study selection and analysis), we agree with several authors who suggested that CBT may be beneficial but further research is necessary[163-166] or that a consistent measurement scale is necessary to compare the interventions[167].

At present, we are unable to agree with researchers who concluded that CBT is an effective intervention for all autistic people[168, 169] or that group CBT should be introduced into service for youth and adolescents[170]. This is because there was insufficient evidence to conclude that CBT is an effective intervention for all autistic people, and because of the uncertainty in the way CBT should be delivered and the uncertainty in the implications of decreased anxiety or depression scores.

#### Future research

We have developed the following directions for future research based on the findings of the reviews, list of ongoing studies (Appendix 7), literature reviews, and creative thinking.

##### Study design

Randomised controlled trial (further details of the design can be found below).

##### Participants

###### People with intellectual disability

While there are reasonable number of studies for children and adults without intellectual disability, none of the trials included solely people with intellectual disability; even when trials included people with intellectual disability, the outcomes were not reported. We acknowledge the difficulties in such research: for example, there are no recommended tools to measure mental health outcomes in people with intellectual disability[171-174]. The last search date in these systematic reviews was September 2016 and the systematic reviews did not specifically look at validation studies of measurement tools in the autistic population. Therefore, a programme of research which involves systematic reviews of the measurement properties of the instruments in autistic people with ID and agreement of how to measure whether interventions work is necessary prior to trials comparing interventions.

###### People without intellectual disability

Despite the many trials that we identified, the trials had some concerns about bias or were at high risk of bias. Therefore, there is great uncertainty about the effects of the interventions. So, people without intellectual disability should also be studied in future trials at low risk of bias.

##### Intervention

As indicated earlier, CBT-based approaches appear to be the most promising intervention; mindfulness therapy also appears promising in a subset of autistic people with previous mental health problems. However, there is great uncertainty in the content, who delivers it, and for how long it should be delivered. Purely from a logistical and resource use point of view, self-directed CBT appears to be attractive, as this relies on less input from an external person when delivering this. However, adherence and motivation could be potential issues, as there is less professional support available. Compliance to CBT predicts the outcome[175]. It should be noted that CBT can be tailored to an individual[176]. It is worth exploring whether tailoring of CBT can be effectively done by answers to a series of questions asked by an individual or artificial intelligence and an effective self-directed personalised CBT focusing on factors that underpin mental health problems in autistic people, such as sensory difficulties[177] can be developed. Such an intervention is likely to be less resource intensive and can change over time (without incurring considerable costs) as circumstances change (the change of CBT over time might increase the compliance). Further research involving interviews and focus group meetings are probably necessary to develop acceptable and feasible ways of delivering CBT for long periods of time.

It should be noted that the interventions were provided in additional to the usual supportive care that autistic people received in the trials. Therefore, CBT should be considered in addition to the usual supportive care and not as replacement for usual supportive care.

##### Control

Usual supportive care

##### Outcome measures in effectiveness trials in autistic people (with or without ID)

Many trials were targeted at reducing core features of autism, with mental health assessed as a secondary outcome. Overall autistic symptom scores are not a measure of mental health and should not be used to assess whether an intervention works to improve mental health problems, nor to show whether a treatment is appropriate (i.e., people should not be excluded from interventions on the basis of having higher levels of autistic traits). Our focus group also identified opposition to the overall autistic symptom scores as outcome measures in future trials.

We did not find any project related to the core outcome measures in effectiveness trials in autism. We are aware of an ongoing project to identify core outcome measures in effectiveness trials in autism (personal communication from Head of Research, Autistica). This is conducted by an organisation whose main source of income is pharmaceutical industry[178]. Once the project is completed, engagement with autistic people about the relative importance of the outcome measures identified is necessary to find out whether the outcome measures identified in the project are unbiased. Once it is clear that the core outcome measures resulting from the project is unbiased, these outcomes can be included in the trials comparing interventions. We are also aware of a toolbox of core outcome measures that are co-produced or co-modified from existing measures[179]. The methods used in this project do not appear to follow the methods suggested by the Core Outcome Measures in Effectiveness Trials Initiative. Nevertheless, this project has the potential to identify the outcome measures to be used in future trials.

As mentioned earlier, the two systematic reviews related to measurement properties of mental health outcomes [154, 158] highlighted the lack of robust and reproducible evidence to support that the outcome measures used in trials reliably measure the effectiveness of the interventions. The studies we included used a variety of mental-health related outcome measures that have not been validated in autistic people. As also mentioned earlier, the minimal important differences for these outcome measures are also lacking. Therefore, further research is necessary to find the reliability of these measures and the minimal important differences, i.e., it is important to know when to say that an intervention can make a difference to the outcome(s) important to autistic people, their carers, and health care, social care, and educational professionals involved in providing the care for autistic people.

##### Further details of the study design to decrease the risk of bias

Randomisation can be performed using standard methods, for example, web-based central randomisation; an intention-to-treat analysis can be performed; and a protocol should be published prior to recruitment. However, blinding of providers and participants may not be possible for interventions such as CBT or mindfulness therapy. Since the mental health outcomes relate to mood and behaviour, they are subjective outcomes. There is uncertainty about the impact of lack of blinding on subjective outcomes: some meta-epidemiological studies suggest that lack of blinding leads to bias[180, 181], while others suggest that there is no evidence of bias due to lack of blinding[182]. While further research related to this aspect is awaited, we must accept that there may be an element of overestimation of the effect of an intervention and should factor this into the interpretation of findings.

###### Sample size

Since we do not know the outcome measure used and the MID for the outcome measure, we are unable to estimate the sample size for the trial.

###### Follow-up

Only one of the trials [88] had follow-up longer than 12 months. Even this trial had a follow-up of 24 months only. However, autism is a life-long condition and circumstances for autistic people may change for autistic people just as they may change for non-autistic people. While short-term follow-up may be sufficient if the purpose of the study is to assess the impact of an intervention on a short-term acute mental health problem, long-term follow-up after interventions are necessary to assess whether the impact of an intervention targeted at preventing or improving mental health outcomes is long-lasting.

Linking education, social care and electronic health records[183, 184], education, health and care plans[185], as well as mortality records such as those maintained by the Office for National Statistics will allow long-term follow of outcomes and resource utilisation without incurring considerable trial costs. However, the use of national electronic health record data brings its own challenges such as data quality and validation, completeness of data capture, and heterogeneity among systems for international trials[186]. Besides, national electronic health record data does not include anxiety scores, depression scores, or health-related quality of life. Potential solutions include self-reported measures, but there is no current evidence on the validity of these approaches and the biases in these approaches. Therefore, nesting methodological research projects within autism trials can determine the optimal trade-off between the most valid and most efficient study designs in trials involving autistic people.

#### Authors' conclusions

##### Implications for practice

The evidence indicates considerable uncertainty about the effects of the different interventions in decreasing mental health problems in autistic people. Some forms of cognitive behavioural therapy may decrease the anxiety and depression scores in some adults and children with or without mental health problems. Mindfulness therapy may decrease the anxiety and depression scores in some adults and children with mental health problems. In line with NICE guidelines, we recommend against the use of medications routinely to manage core features of autism because of the lack of evidence about their effectiveness and potential adverse events. There is insufficient evidence regarding the effectiveness of medications to treat mental health problems in autistic people, and as such, guidance for the use of medications for mental health problems in non-autistic people should continue to be used until further research has been conducted on this issue.

##### Implications for research

Further well-designed randomised clinical trials are necessary. However, before conducting such randomised controlled trials, one needs to understand how outcomes should be measured and when the intervention can be considered effective. Changes in core features of autism do not correlate with changes in mental health; therefore, core features of autism should not be used to assess whether an intervention works to improve mental health problems nor to show whether a treatment is appropriate based on the levels of their autistic traits (i.e., people should not be excluded from interventions on the basis of having higher levels of autistic traits). Methodological research within trials may help with conducting trials in the optimal way.

## Funding

Blinded for review

## Conflicts of interest

Blinded for review

## Acknowledgements

Blinded for review

## Contribution of authors

Blinded for review

## Deviations from protocol

The major deviations from the protocol were as follows.

1. We changed our use of terminology around autism in line with the preferences of autistic people[187].
2. We conducted the focus group for identifying the relative importance of the outcomes prior to the data extraction rather than after completing the review. This meant that the relative importance was based on the importance to autistic people rather than availability of the information; therefore, this is more robust than our original plan in terms of finding out how important the outcome was for autistic people.
3. We accepted anxiety and depression however measured (not just as proportion) as primary measure. We noted that majority of the trialists reported this outcome domain as continuous measures using validated scales. As these scales measure the same outcome domain, we have included the scales in the same outcome domain as a primary outcome measure.
4. We have added self-harm and meaningful life activities as secondary measures and removed the overall improvement in core features of autism as a secondary measure. This was because of the patient and public engagement activity in which participants emphasised the need to include self-harm and meaningful life activities as important to them and did not want core features of autism as an outcome measure which devalued them.
5. We also added any adverse events as one of the outcomes as many trialists did not report serious and non-serious adverse events separately.
6. As there was no meaningful way in which to rank the included studies for a comparison-adjusted funnel plot, we used the completeness of the search and absence of reporting to assess reporting bias.
7. We performed an exploratory metaregression to find if the differences in anxiety and depression could be explained by overall autistic symptom scores.

## Tables

### Table 1: Characteristics of included studies (ordered by comparisons)

| Study name | Intervention 1 | Intervention 2 | Intervention 1: number of participants | Intervention 2: number of participants | Intellectual disability | Age group | Pre-existing mental health issues | Period of recruitment | Follow-up in months | Source of funding |
| --- | --- | --- | --- | --- | --- | --- | --- | --- | --- | --- |
|
| Chalfant 2007[90] | CBTAdaptedGroup | NoAdditionalIntervention | 28 | 19 | Normal or high IQ | Primary or secondary school children | Previous anxiety | Not stated | 6.0 | Not clear |
| Kilburn 2020[120] | CBTAdaptedGroup | NoAdditionalIntervention | 19 | 19 | Normal or high IQ | Primary or secondary school children | Previous anxiety | 2016-2018 | 3.0 | Industry-funded |
| Langdon 2016[129] | CBTAdaptedGroup | NoAdditionalIntervention | 23 | 25 | Normal or high IQ | Any adults | Previous anxiety | Not stated | 5.5 | Non-industry funded |
| Mackay 2017[141] | CBTAdaptedGroup | NoAdditionalIntervention | 16 | 12 | Normal or high IQ | Primary or secondary school children | Included people with and without previous mental health issues | Not stated | 6.0 | Non-industry funded |
| Santomauro 2016[140] | CBTAdaptedGroup | NoAdditionalIntervention | 8 | 5 | Normal or high IQ | Secondary school children or adolescents | Previous depression | July 2013 - June 2014 | 5.1 | Non-industry funded |
| Weiss 2018[115] | CBTAdaptedGroup | NoAdditionalIntervention | 35 | 33 | Normal or high IQ | Primary or secondary school children | Not stated | Jan 2013 - April 2016 | 2.5 | Industry-funded |
| Fujii 2013[89] | CBTAdaptedFamilyBased | NoAdditionalIntervention | 7 | 5 | Normal or high IQ | Primary school children (around 5 to 11 years) | Previous anxiety |  | 7.4 | Not stated |
| Luxford 2017[112] | CBTAdaptedFamilyBased | NoAdditionalIntervention | 18 | 17 | Normal or high IQ | Secondary school children or adolescents | Previous anxiety | Not stated | 3.0 | Non-industry funded |
| Mcconachie 2014[103] | CBTAdaptedFamilyBased | NoAdditionalIntervention | 17 | 14 | Normal or high IQ | Primary or secondary school children | Previous anxiety | Not stated | 3.0 | Non-industry funded |
| Storch 2013a[124] | CBTAdaptedFamilyBased | NoAdditionalIntervention | 24 | 21 | Normal or high IQ | Primary school children (around 5 to 11 years) | Previous anxiety | March 2010-January 2012 | 3.0 | Industry-funded |
| Wood 2009[93] | CBTAdaptedFamilyBased | NoAdditionalIntervention | 14 | 22 | Normal or high IQ | Primary school children (around 5 to 11 years) | Not stated | September 2004-August 2007 | 4.0 | Industry-funded |
| Wood 2015[108] | CBTAdaptedFamilyBased | NoAdditionalIntervention | 19 | 14 | Normal or high IQ | Primary or secondary school children | Not stated |  | 5.0 | Non-industry funded |
| Andrews 2013[188] | CBTAdaptedFamilyBased | CBTAdaptedGroup | 29 | 30 | Normal or high IQ | Primary school children (around 5 to 11 years) | Not stated | Not stated | 3.0 | Non-industry funded |
| Maskey 2019b[117] | CBTAdaptedIndividual | NoAdditionalIntervention | 14 | 13 | Not stated | Primary or secondary school children | Previous anxiety | 2015-2016 | 6.0 | Non-industry funded |
| Mcnally Keehn 2013[35] | CBTAdaptedIndividual | NoAdditionalIntervention | 12 | 10 | Normal or high IQ | Primary or secondary school children | Previous anxiety | Not stated | 3.7 | Non-industry funded |
| Storch 2015[107] | CBTAdaptedIndividual | NoAdditionalIntervention | 16 | 15 | Normal or high IQ | Primary or secondary school children | Previous anxiety |  | 3.7 | Industry-funded |
| Wood 2020[84] | CBTAdaptedIndividual | NoAdditionalIntervention | 66 | 18 | Normal or high IQ | Primary or secondary school children | Previous anxiety | April 2014 - Jan 2017 | 3.7 | Non-industry funded |
| Nct 2013e[145] | MedicationADH | NoAdditionalIntervention | 17 | 13 | All IQ | Primary school children (around 5 to 11 years) | Not stated | Dec 2013 - May 2017 | 1.0 | Non-industry funded |
| Parker 2019[127] | MedicationADH | NoAdditionalIntervention | 17 | 13 | All IQ | Primary school children (around 5 to 11 years) | Not stated | 2013-2017 | 0.9 | Non-industry funded |
| Squassante 2018[85] | MedicationADH | NoAdditionalIntervention | 141 | 72 | Normal or high IQ | Any adults | Not stated |  | 2.8 | Industry-funded |
| Umbricht 2017[131] | MedicationADH | NoAdditionalIntervention | 9 | 9 | Normal or high IQ | Any adults | Not stated |  | 1.0 | Industry-funded |
| Corbett 2017[111] | SkillsTraining | NoAdditionalIntervention | 17 | 13 | Normal or high IQ | Primary or secondary school children | Not stated | Not stated | 3.0 | Non-industry funded |
| Kuehnel 2014[102] | SkillsTraining | NoAdditionalIntervention | 19 | 23 | Not stated | Secondary school children or adolescents | Not stated | Not stated | 1.4 | Not stated |
| Mcvey 2016a[130] | SkillsTraining | NoAdditionalIntervention | 24 | 23 | Normal or high IQ | Any adults | Not stated | Not stated | Not stated | Non-industry funded |
| Morgan 2014[144] | SkillsTraining | NoAdditionalIntervention | 12 | 12 | Normal or high IQ | Any adults | Not stated | August 2012 to January 2013 | 5.8 | Not clear |
| Drahota 2009[122] | CBTNonAdaptedFamilyBased | NoAdditionalIntervention | 17 | 23 | Normal or high IQ | Primary school children (around 5 to 11 years) | Previous anxiety | Not stated | 3.0 | Non-industry funded |
| Nct 2013b[97] | CBTNonAdaptedFamilyBased | NoAdditionalIntervention | 14 | 18 | Normal or high IQ | Primary school children (around 5 to 11 years) | Previous anxiety |  | 6.0 | Non-industry funded |
| Reaven 2012[147] | CBTNonAdaptedFamilyBased | NoAdditionalIntervention | 20 | 23 | Normal or high IQ | Primary or secondary school children | Previous anxiety | not stated | 2.8 | Industry-funded |
| Conaughton 2017[110] | CBTNonAdaptedSelfDirected | NoAdditionalIntervention | 18 | 18 | Normal or high IQ | Primary or secondary school children | Previous anxiety | Not stated | 3.0 | Not stated |
| Gaigg 2020[137] | CBTNonAdaptedSelfDirected | NoAdditionalIntervention | 9 | 16 | Normal or high IQ | Any adults | Not stated | Jun 2016 - April 2017 | 6.0 | Non-industry funded |
| Bernaerts 2020[135] | MedicationOxytocin | NoAdditionalIntervention | 22 | 18 | Normal or high IQ | Any adults | Not stated | 2015-16 | 12.0 | Non-industry funded |
| Nct 2012b[96] | MedicationOxytocin | NoAdditionalIntervention | 16 | 18 | All IQ | Primary school children (around 5 to 11 years) | Not stated | Jun 2012 - April 2016 | 1.0 | Non-industry funded |
| Yamasue 2020[87] | MedicationOxytocin | NoAdditionalIntervention | 51 | 52 | Normal or high IQ | Any adults | Not stated | 2015-2016 | 1.4 | Non-industry funded |
| Eslamzadeh 2018[113] | MedicationSNRI | NoAdditionalIntervention | 22 | 22 | All IQ | Primary or secondary school children | No previous mental health issues |  | 2.0 | Non-industry funded |
| Hospital 2007[91] | MedicationSNRI | NoAdditionalIntervention | 27 | 30 | All IQ | Primary or secondary school children | Not stated | July 2007 - Oct 2015 | 1.9 | Non-industry funded |
| Politte 2018[37] | MedicationSNRI | NoAdditionalIntervention | 30 | 32 | All IQ | Primary or secondary school children | No previous mental health issues | not stated | 1.8 | Industry-funded |
| Gaigg 2020[137] | Mindfulness | NoAdditionalIntervention | 14 | 16 | Normal or high IQ | Any adults | Not stated | Jun 2016 - April 2017 | 6.0 | Non-industry funded |
| Pagni 2020[138] | Mindfulness | NoAdditionalIntervention | 15 | 13 | Normal or high IQ | Any adults | Included people with and without previous mental health issues | Not stated | 1.8 | Non-industry funded |
| Spek 2013[38] | Mindfulness | NoAdditionalIntervention | 20 | 21 | Normal or high IQ | Any adults | Previous anxiety or depression |  | 2.1 | Not stated |
| Gaigg 2020[137] | Mindfulness | CBTNonAdaptedSelfDirected | 14 | 9 | Normal or high IQ | Any adults | Not stated | Jun 2016 - April 2017 | 6.0 | Non-industry funded |
| Oswald 2018[133] | SkillsTrainingGroup | NoAdditionalIntervention | 25 | 15 | Normal or high IQ | Any adults | Included people with and without previous mental health issues | not stated | 2.9 | Non-industry funded |
| Reitzel 2013[98] | SkillsTrainingGroup | NoAdditionalIntervention | 8 | 7 | Not stated | Primary school children (around 5 to 11 years) | Not stated | not stated | Not stated | Non-industry funded |
| Wright 2016[109] | SkillsTrainingGroup | NoAdditionalIntervention | 23 | 27 | Not stated | Primary or secondary school children | Not stated |  | 3.7 | Non-industry funded |
| Solomon 2008[139] | ABA | NoAdditionalIntervention | 10 | 9 | Normal or high IQ | Primary or secondary school children | Not stated |  | 3.2 | Not clear |
| Cortesi 2012[83] | CBTNonAdaptedIndividual | NoAdditionalIntervention | 33 | 32 | Not stated | Primary school children (around 5 to 11 years) | Not stated | 2007-2010 | 2.8 | Non-industry funded |
| Wood 2020[84] | CBTNonAdaptedIndividual | NoAdditionalIntervention | 59 | 18 | Normal or high IQ | Primary or secondary school children | Previous anxiety | April 2014 - Jan 2017 | 3.7 | Non-industry funded |
| Wood 2020[84] | CBTNonAdaptedIndividual | CBTAdaptedIndividual | 59 | 66 | Normal or high IQ | Primary or secondary school children | Previous anxiety | April 2014 - Jan 2017 | 3.7 | Non-industry funded |
| Liu 2019[126] | DietarySupplement | NoAdditionalIntervention | 36 | 35 | Not stated | Primary or secondary school children | Not stated | 2016 | 0.9 | Industry-funded |
| Mehrazad-Saber 2018[125] | DietarySupplement | NoAdditionalIntervention | 21 | 22 | Not stated | Primary or secondary school children | Not stated | Not stated | 2.0 | Not stated |
| Hesselmark 2014[143] | GroupActivity | CBTAdaptedGroup | 28 | 27 | Normal or high IQ | Any adults | Not stated | Aug 2005 - Sept 2011 | 8.3 | Non-industry funded |
| Sung 2011[95] | GroupActivity | CBTAdaptedGroup | 29 | 28 | Normal or high IQ | Primary or secondary school children | Previous anxiety | February 2007-August 2008 | 6.0 | Non-industry funded |
| Potter 2019[118] | MedicationSSRI | NoAdditionalIntervention | 26 | 21 | Not stated | Toddlers or preschool children (< 4 to 5 years) | Not stated | 2015-2018 | 6.0 | Non-industry funded |
| Reddihough 2019[86] | MedicationSSRI | NoAdditionalIntervention | 53 | 53 | All IQ | Primary or secondary school children | Not stated | 2010-2017 | 3.7 | Non-industry funded |
| Schohl 2014[104] | SkillsTrainingGroupPEERS | NoAdditionalIntervention | 29 | 29 | Normal or high IQ | Secondary school children or adolescents | Not stated | not stated | 3.2 | Non-industry funded |
| Yoo 2014[105] | SkillsTrainingGroupPEERS | NoAdditionalIntervention | 23 | 24 | Normal or high IQ | Secondary school children or adolescents | Not stated |  | 3.2 | Industry-funded |
| White 2013[100] | SkillsTrainingMASSI | NoAdditionalIntervention | 15 | 15 | Normal or high IQ | Secondary school children or adolescents | Previous anxiety | Oct 2008 - Oct 2010 | 3.4 | Non-industry funded |
| Du 2015[106] | ABA+MedicationDiuretic | ABA | 29 | 26 | Not stated | Toddlers or preschool children (< 4 to 5 years) | Not stated | Not stated | 3.0 | Non-industry funded |
| Tachibana 2013[99] | BookReading | NoAdditionalIntervention | 6 | 4 | Normal or high IQ | Primary school children (around 5 to 11 years) | Not stated |  | 2.0 | Not clear |
| Cook 2019[116] | CBTAdaptedParentMediated | NoAdditionalIntervention | 12 | 14 | Normal or high IQ | Toddlers or preschool children (< 4 to 5 years) | Previous anxiety | Not stated | 3.0 | Not stated |
| Russell 2019[134] | CBTAdaptedSelfDirected | NoAdditionalIntervention | 28 | 20 | Normal or high IQ | Any adults | Previous depression | 2016-2017 | 6.5 | Non-industry funded |
| Storch 2020[121] | CBTFamilyBasedExposureFocussed | NoAdditionalIntervention | 11 | 18 | Normal or high IQ | Primary or secondary school children | Previous anxiety | 2012-2015 | 2.8 | Non-industry funded |
| Scarpa 2011[94] | CBTNonAdaptedGroup | NoAdditionalIntervention | 5 | 6 | Not stated | Primary school children (around 5 to 11 years) | Not stated | not stated | 2.3 | Not stated |
| Cortesi 2012[83] | CBTNonAdaptedIndividual+MedicationMelatonin | NoAdditionalIntervention | 35 | 32 | Not stated | Primary school children (around 5 to 11 years) | Not stated | 2007-2010 | 2.8 | Non-industry funded |
| Cortesi 2012[83] | CBTNonAdaptedIndividual+MedicationMelatonin | CBTNonAdaptedIndividual | 35 | 33 | Not stated | Primary school children (around 5 to 11 years) | Not stated | 2007-2010 | 2.8 | Non-industry funded |
| Murphy 2017[36] | Counselling | SkillsTrainingMASSI | 19 | 17 | Normal or high IQ | Secondary school children or adolescents | Previous anxiety | April 2011 - April 2013 | 6.0 | Non-industry funded |
| Isong 2014[101] | Distraction | NoAdditionalIntervention | 15 | 19 | Not stated | Primary or secondary school children | Previous anxiety | Dec 2010 - July 2012 | 6.0 | Not clear |
| Balci 2020[142] | IndividualCBT | NoAdditionalIntervention | 11 | 13 | Normal or high IQ | Primary or secondary school children | Previous other mental health issues | June 2017-2019 | 1.6 | Not stated |
| Mcdougle 1998[128] | MedicationAtypicalAntiPsychotic | NoAdditionalIntervention | 14 | 16 | All IQ | Any adults | Not stated | Not stated | 2.8 | Non-industry funded |
| Danforth 2018[132] | MedicationMDMA | NoAdditionalIntervention | 8 | 4 | Normal or high IQ | Any adults | Previous anxiety | Feb 2014 - April 2017 | 6.0 | Industry-funded |
| Cortesi 2012[83] | MedicationMelatonin | NoAdditionalIntervention | 34 | 32 | Not stated | Primary school children (around 5 to 11 years) | Not stated | 2007-2010 | 2.8 | Non-industry funded |
| Cortesi 2012[83] | MedicationMelatonin | CBTNonAdaptedIndividual | 34 | 33 | Not stated | Primary school children (around 5 to 11 years) | Not stated | 2007-2010 | 2.8 | Non-industry funded |
| Cortesi 2012[83] | MedicationMelatonin | CBTNonAdaptedIndividual+MedicationMelatonin | 34 | 35 | Not stated | Primary school children (around 5 to 11 years) | Not stated | 2007-2010 | 2.8 | Non-industry funded |
| Dean 2017[33] | MedicationNAC | NoAdditionalIntervention | 48 | 50 | All IQ | Primary school children (around 5 to 11 years) |  | 2011-2013 | 6.0 | Non-industry funded |
| Nct 2011c[123] | MedicationNaSSA | NoAdditionalIntervention | 20 | 10 | All IQ | Primary or secondary school children | Previous anxiety |  | 2.5 | Industry-funded |
| Campbell 1993[146] | OpioidReceptorAntagonist | NoAdditionalIntervention | 23 | 18 | All IQ | Children of any age | Not stated | Not stated | 0.9 | Industry-funded |
| Bischof 2018[88] | ParentPsychoeducation | NoAdditionalIntervention | 11 | 13 | Not stated | Toddlers or preschool children (< 4 to 5 years) | Not stated | 2011-2012 | 24.0 | Not stated |
| Piravej 2009[92] | SensoryIntegrationTherapy+ThaiTraditionalMassage | SensoryIntegrationTherapy | 30 | 30 | Not stated | Children of any age | Not stated | not stated | 2.0 | Non-industry funded |
| Ioannou 2020[119] | SkillsTrainingGroupSENSE | NoAdditionalIntervention | 44 | 33 | Normal or high IQ | Primary or secondary school children | Not stated | Not stated | Not stated | Non-industry funded |
| Capriola-Hall 2020[136] | SkillsTrainingIndividual | NoAdditionalIntervention | 16 | 16 | Normal or high IQ | Young adults (around 18 to 25 years) | Not stated | Not stated | 3.7 | Non-industry funded |
| Pryor 2018[114] | SkillsTrainingSelfdirected | CBTNonAdaptedSelfDirected | 12 | 12 | Normal or high IQ | Primary or secondary school children | Previous anxiety | not stated | 2.8 | Not stated |
| Isong 2014[101] | SkillsTrainingVideo | NoAdditionalIntervention | 17 | 19 | Not stated | Primary or secondary school children | Previous anxiety | Dec 2010 - July 2012 | 6.0 | Not clear |
| Isong 2014[101] | SkillsTrainingVideo | Distraction | 17 | 15 | Not stated | Primary or secondary school children | Previous anxiety | Dec 2010 - July 2012 | 6.0 | Not clear |
| Isong 2014[101] | SkillsTrainingVideo+Distraction | NoAdditionalIntervention | 18 | 19 | Not stated | Primary or secondary school children | Previous anxiety | Dec 2010 - July 2012 | 6.0 | Not clear |
| Isong 2014[101] | SkillsTrainingVideo+Distraction | Distraction | 18 | 15 | Not stated | Primary or secondary school children | Previous anxiety | Dec 2010 - July 2012 | 6.0 | Not clear |
| Isong 2014[101] | SkillsTrainingVideo+Distraction | SkillsTrainingVideo | 18 | 17 | Not stated | Primary or secondary school children | Previous anxiety | Dec 2010 - July 2012 | 6.0 | Not clear |

### Table 2A: Reasons for exclusion of studies

| Reason for exclusion | Number of studies | Study names |
| --- | --- | --- |
| Not in autistic people | 64 | Actrn 2014B; Actrn 2016G; Actrn 2017E; ACTRN 2020; Anonymous 1980; Bradshaw 2018; Carter Alena 2005; Center 2009; Center 2012; Chictr-Trc 2012; Ctri 2017E; Da Paz 2018; Dickson 2020; Dille 2010; Dubicka 2008; Dykens 2014; Erickson 2011; Estes 2014; Euctr-009475-35-Nl 2009; Feinberg 2014; Fox 2018; Goodday 2014; Gulsrud 2014; Hajiabolhasani-Nargani 2016; Hemdi 2017; Iadarola 2018B; Ichikawa 2013; Ingersoll 2015; Irct201404222394n 2014; Irct2016080829268n 2017; Irct2016111130830n 2017; Irct2016112330230n 2016; Isrctn 2011C; Isrctn 2016F; Johnson 2014; Jprn 2016A; Karp 2015; Kuhlthau 2019; Kuravackel 2018; Locke 2012; Maughan 2017; Nct 2009L; Nct 2011L; Nct 2015j; Nct 2015k; Nct 2016c; Nct 2018g; Nct01712464 2012; Nct03757585 2018; Ogba 2020; Rbr-44vc9n 2020; Reed 2018; Respirerx 2002; Riahi 2012A; Roux 2013; Sofronoff 2002; Sutoko 2019; Suzuki 2012; Suzuki 2014; Unis 2002; University 2010; Whittingham 2020; Yoder 2020; Zuidersma 2019 |
| Not a RCT | 184 | Actrn 2011E; Actrn 2018C; Actrn 2018D; Alliance 2006; Anagnostou 2010B; Anagnostou 2013C; Anninos 2016; Anonymous 2002; Anonymous 2009; Anonymous1; Anonymous2; Anonymous5; Anonymous8; Awad 1996; Becker 2017; Beckloff 1998; Bharathi 2019; Blackman 2020; Bonnot 2015B; Buitelaar 1996; Caihong 2016; Campillo 2014; Capano 2018; Cell 2017; Center 2005; Chaitanya Hospital 2014; Chan 2014; Chictr 2018I; Chictr 2018J; Chictr 2018K; Chictr 2018L; Chictr 2018M; Chictr-Ccc 2013; Chictr-Onrc 2012; Chictr-Oon 2014; Chictr-Oon 2017; Chictr-Tnrc 2011; Chlebowski 2020; Chu 2012; Clifford 2013; Corbett 2011; Corti 2018; Council 2009B; Ctri 2018C; Ctri 2018D; Curemark 2010; Curemark 2015B; Davlantis 2015; Dekker 2020; D'elia 2014; Demb 1996; Demurie 2010; Derguy 2018; Development 2006B; Dinasty 2018; Einfeld 2018; Ernst 1993; Euctr-001220-31-Outside-Eu/Eea 2015; Ghaziuddin 1991; Goeb 2009A; Goeb 2009B; Goodman 2018; Grandgeorge 2012; Hadjikhani 2018; Hallett 2020; Handen 2017; Hegarty 2017; Hepburn 2016; Higashida 2019; Holtzer 2015; Honomichl 2002; Hospital 2014B; Hospital 2017A; Howard 2005; Howlin 2018; Ichikawa 2018; Irct201109037462n 2011; Irct20130904014562n 2018; Irct201405273930n 2014; Irct2014102919732n 2015; Irct20171231038156n 2018; Isrctn 2018C; Johnson 2015; Jprn-Umin 2010C; Jprn-Umin 2014D; Jprn-Umin 2015C; Khamooshi 2015; Kim 2020; King 2002; Klebanoff 2019; Knabe 1990; Koehne 2016; Koshes 1997; Laboratories 2012; Laugeson 2014; Leadbitter 2020; Lerner 2011; Lopata 2015A; Lopata 2015B; Mackay 2007; Malone 2006; Malow 2020; Mcconachie 2005; Mccracken 2005A; Medicine 2002; Medicine 2004B; Medicine 2005A; Medicine 2005B; Medicine 2007B; Melamed 2013; Miller 2012; Molnar 2017; Myers 2010; Nct 2005M; Nct 2008O; Nct 2013Q; Nct 2014\; Nct 2014]; Nct 2015e; Nct 2015f; Nct 2016a; Nct 2016b; Nct 2016f; Nct 2017f; Nct02574559 2015; Nct03225651 2017; Nct03786744 2018; Nct04007224 2019; Nct04427150 2020; Nebraska 2009; Nguyen 2016; Nigam 2010; Ntr 2015; Ntr 2016C; Ntr 2017C; Ouellette 2019; Pactr 2018; Pangalila Ratulangi 1973; Pardo 2013; Peters-Scheffer 2010; Peters-Scheffer 2013; Postorino 2019; Potenza 1999; Professional 2018; Propper 2018; Ratcliffe 2014; Reaven 2009; Reinblatt 2006; Research Units on Pediatric Psychopharmacology Autism 2005; Roberts-Collins 2018; Rodgers 2017; Sadek 2018; Saklayen 2011; Salt 2002A; Sasayama 2009; Scahill 2001B; Scahill 2009; Scherzo 2011; Seaside Therapeutics 2011; Selles 2015; Selvapandiyan 2019; Shaffer 2018; Shahani 2012; Shea 2006; Shenzhen Beike Bio-Technology Co 2009; Shih 2016; Silva 2014; Sokhadze 2012; Sokhadze 2018; Storch 2012; Szabo 1994; Tang 2020; Tufan 2009; University 2004B; Van Steensel 2014; Vitiello 2005A; Vitiello 2005B; Vuijk 2017; Warreyn 2005; White 2015; Wichers 2019; Wijnhoven 2015; Wijnhoven 2020; Zamani 2017 |
| No mental health outcomes | 1394 | Aaronson 2018; Actrn 2008A; Actrn 2009A; Actrn 2009B; Actrn 2009C; Actrn 2010A; Actrn 2010B; Actrn 2010C; Actrn 2011A; Actrn 2011B; Actrn 2011C; Actrn 2011D; Actrn 2012A; Actrn 2012B; Actrn 2013A; Actrn 2013B; Actrn 2014A; Actrn 2015A; Actrn 2015B; Actrn 2015C; Actrn 2015D; Actrn 2016A; Actrn 2016B; Actrn 2016C; Actrn 2016D; Actrn 2016E; Actrn 2016F; Actrn 2017A; Actrn 2017B; Actrn 2017C; ACTRN 2017D; Actrn 2018A; Actrn 2018B; Actrn 2019A; Actrn 2019B; Actrn 2019C; Adams 2009; Adams 2011; Adams 2012; Adams 2018; Adibsereshki 2015; Aghababaei 2020; Ajram 2015B; Akabogu 2019; Akhondzadeh 2008; Alaerts 2020A; Albaum 2020; Aldred 2004; Alolaby 2020; Alquraini 2019; Althaus 2015; Alvares 2019; Aman 2005; Aman 2009B; Aman 2015; Aman 2017; Aman 2018; Amatachaya 2015; Ameis 2020; Amminger 2007B; Anagnostou 2006A; Anagnostou 2010A; Anagnostou 2011; Anagnostou 2012; Anagnostou 2013A; Anagnostou 2013B; Anagnostou 2014; Anagnostou 2016A; Anagnostou 2016B; Anderson 1989; Anderson 2009; Anonymous 2005A; Anonymous 2005B; Anonymous 2011; Anonymous 2016A; Anonymous 2016B; Anonymous10; Anonymous3; Anonymous6; Anonymous7; Antonini 2017; Aoki 2014; Arabi 2019; Arkansas 2012; Arnold 2003; Arnold 2006; Arnold 2012; Arnold 2018; Arnold 2019; As 2015; Asadabadi 2013; Auyeung 2015; Ayatollahi 2020; Bader 2006; Bagaiolo 2017; Ballester 2015; Ballester 2019; Bangabandhu 2019; Barthelemy 1989; Bauminger-Zviely 2020; Bearss 2015; Beaumont 2008A; Beaumont 2018B; Begeer 2011; Begeer 2015; Behmanesh 2019; Bekhet 2017A; Belsito 2001; Bent 2014; Benton 2011; Bernaerts 2017; Bernard-Opitz 2004; Bertoglio 2010; Bettison 1996; Beversdorf 2011; Beversdorf 2014; Beversdorf 2015; Bieleninik 2017; Black 2012; Bolte 2016; Bonnot 2015A; Bordini 2020; Borgi 2016; Borowiak 2020; Bouvard 1995; Bowrin 2020; Boyd 2018; Bradshaw 2019; Brainsway 2013; Brian 2017; Brito 2020; Brookma-Frazee 2020; Brunero 2009; Buchsbaum 2001; Buitelaar 1992; Byford 2015; Campbell 1978; Campbell 1982; Campbell 1990; Carey 2016; Cariveau 2019; Carminati 2016; Carolina 2011; Carr 2016; Carrick 2018; Carter 2011; Castorina 2011; Center 2002; Center 2010; Center 2011; Center 2014; Center 2016; Center 2017; Center 2020A; Center 2020B; Center for Autisme 2006; Central Hospital 2015; Cermak 2015; Chan 2013; Chen 2020; Cheng 2018B; Chester 2019; Chez 2018; Chi 2012; Chi 2017; Chictr 2018A; Chictr 2018B; Chictr 2018C; Chictr 2018D; Chictr 2018E; Chictr 2018F; Chictr 2018G; Chictr 2018H; ChiCTR 2019A; ChiCTR 2019B; ChiCTR 2019C; ChiCTR 2019D; ChiCTR 2019E; ChiCTR 2019F; ChiCTR 2020A; ChiCTR 2020B; ChiCTR 2020C; ChiCTR 2020D; ChiCTR 2020E; ChiCTR 2020F; ChiCTR 2020G; ChiCTR 2020H; ChiCTR 2020I; ChiCTR 2020J; ChiCTR 2020K; ChiCTR 2020L; ChiCTR 2020M; Chictr-Iir 2016; Chictr-Inr 2017; Chictr-Ior 2016; Chictr-Ior 2017A; Chictr-Ior 2017B; Chictr-Ipr 2016; Chictr-Ipr 2017A; Chictr-Ipr 2017B; Chictr-Ipr 2017C; Chictr-Trc 2009; Chictr-Trc 2014; Children 1999; Children's Hospital Medical Center 2013A; Children's Hospital Medical Center 2013B; Choque Olsson 2017; Chugani 2004; Chugani 2016; Cibrian 2020; Clinical 2014A; Coggins 1988; Conner 2013; Corbett 2008; Corbett 2016A; Corbett 2019; Coronado Biosciences 2014; Corporation 2002A; Corporation 2002B; Costescu 2017; Council 2009A; Crawford 2017; Crowell 2020; CTRI 2015; Ctri 2016; Ctri 2017A; Ctri 2017B; Ctri 2017C; CTRI 2017D; Ctri 2018A; Ctri 2018B; Ctri 2019A; Ctri 2019B; CTRI 2019C; CTRI 2019D; CTRI 2019E; CTRI 2019F; CTRI 2020A; CTRI 2020B; CTRI 2020C; CTRI 2020D; CTRI 2020E; Curemark 2009; Curemark 2015A; Da Paz 2016; Dadds 2014; Daly 2012; Danfors 2005; Dawalt 2018; Dawson 2010; de Jong 2019; Dean 2020; Deckers 2016; Dekker 2014; Del Valle Rubido 2015; Derosier 2011; Desarkar 2020; Development 2003; Development 2006A; Development 2019; Divan 2019; Doernberg 2020; Domes 2013; Drks 2012; DRKS 2015A; DRKS 2015B; DRKS 2016A; DRKS 2016B; DRKS 2018; DRKS 2019A; DRKS 2019B; Drks 2019C; DRKS 2020; Eack 2018; Edelson 1999; Enticott 2014; Erickson 2014; Esse Wilson 2018; EU/EEA 2017; Euctr 2006; Euctr 2007; EUCTR 2013; Euctr 2018A; EUCTR 2018B; Euctr 2018C; Euctr-000106-11-Fr 2016; Euctr-000586-45-Be 2014; Euctr-000955-25-Fr 2015; Euctr-001230-17-Fr 2015; Euctr-001320-31-Outside-Eu/Eea 2015; Euctr-001560-35-Nl 2016; Euctr-001689-97-Fr 2009; Euctr-003259-39-Es 2014; Euctr-003712-36-Fr 2008; Euctr-003750-89-De 2012; Euctr-004419-38-De 2018; Euctr-004420-30-Es 2018; Euctr-006126-25-Fr 2007; Euctr-006444-21-Es 2010; Euctr-010393-38-Fr 2009; Euctr-012102-39-It 2009; Euctr-018740-13-Nl 2010; Euctr-022511-18-De 2010; Eugene Arnold 2019; Factor 2019; Falissard 2019; Fang 2018; Feldman 1999; Felzer-Kim 2020; Field 1997; Findling 2014; Fisher 2005; Fisher 2020; Floreo 2018; Frankel 2010A; Frazier 2017; Freitag 2016; Fridenson-Hayo 2017A; Frolli 2020; Frye 2018; Fung 2014; Furukawa 2018; Gabis 2019; Gabriels 2015; Gabriels 2018; Gantman 2012; Gao 2020; Garcia-Villamisar 2011; Garcia-Villamisar 2017; Gengoux 2019; Geretsegger 2016; Germone 2019; Gev 2017; Ghaleiha 2013; Ghaleiha 2014; Ghalichi 2016; Ghanizadeh 2013; Ghodsi 2019; Giarelli 2005; Ginn 2017; Glod 2013; Golan 2010; Gonzalez-Domenech 2020; Goods 2013; Gordon 1992; Gordon 1993; Gordon 2011; Gordon 2015; Gordon 2016; Grahame 2015; Gringras 2014; Gringras 2017; Guastella 2010; Guastella 2015; Gulsrud 2007; Haakonsen Smith 2018; Hadwin 1996; Hagerman 2018; Hagner 2012; Hajizadeh-Zaker 2018; Halas 2016; Handen 2009; Handen 2011; Hannant 2019; Hardan 2012; Hardan 2015; Hardan 2016; Hardan 2019; Harfterkamp 2014; Health 2006B; Health 2010; Health 2012A; Health 2013; Hellings 2001; Hellings 2005; Hendouei 2020; Hendren 2016; Herscu 2020; Ho 2020; Ho Yan 2018; Hochhauser 2018; Hodgetts 2011; Hollander 2003; Hollander 2005; Hollander 2006B; Hollander 2006C; Hollander 2007; Hollander 2010; Hollander 2012; Hollander 2013B; Hollander 2014D; Hollander 2018; Hollander 2019; Hollway 2018; Holopainen 2018; Hopkins 2011; Hospital 2009; Hospital 2011; Hospital 2012; Hospital 2013; Hospital 2014A; Hospital 2014C; Hospital 2015B; Hospital 2018; Hospital 2020A; Hospital 2020B; Howlin 2007; Iadarola 2018A; Ibanez 2018B; Inc 2016; Ingersoll 2012A; Ingersoll 2016; Institute 2018; IRCT 2017; IRCT 2018A; Irct 2018B; Irct 2019A; IRCT 2019B; IRCT 2019C; IRCT 2019D; Irct 2019E; IRCT 2019F; IRCT 2019G; IRCT 2019H; IRCT 2019I; IRCT 2019J; IRCT 2019K; IRCT 2019L; IRCT 2020A; IRCT 2020B; IRCT 2020C; IRCT 2020D; IRCT 2020E; IRCT 2020F; IRCT 2020G; IRCT 2020H; IRCT 2020I; IRCT 2020J; IRCT 2020K; IRCT 2020L; IRCT 2020M; Irct1138901151556n 2010; Irct138711161556n 2009; Irct138808202698n 2010; Irct138901141556n 2010; Irct138904204264n 2010; Irct20090117001556n 2017; Irct20090117001556n 2018A; Irct20090117001556n 2018B; Irct20090117001556n 2018C; Irct20090117001556n 2018D; Irct201012031556n 2010; Irct201101105280n 2011; Irct201106101556n 2011; Irct201106103930n 2011; Irct201107281556n 2011; Irct201108155280n 2013; Irct201110233930n 2011; Irct201110281556n 2011; Irct201202281556n 2012; Irct201204037202n 2012; Irct201204081556n 2012; Irct201204246834n 2012; Irct201205259854n 2012; Irct2012091010806n 2013; Irct2012111011421n 2013; Irct201212079854n 2012; Irct201302201556n 2013; Irct201307303930n 2014; Irct20131013014994n 2018; Irct2013110915339n 2016; Irct201402043930n 2014; Irct20140212016564n 2018; Irct201404212017n 2014; Irct2014102519665n 2014; Irct20150519022323n 2017; Irct2015080223454n 2015; Irct201512081556n 2015; Irct2015122625699n 2016; Irct201512315280n 2016; Irct201602041556n 2016; Irct2016022826802n 2016; Irct2016061711689n 2016A; Irct2016061711689n 2016B; Irct2016061828511n 2016; Irct2016071728966n 2016; Irct2016082215339n 2016; Irct2016100330113n 2017; Irct20161210031330n 2018; Irct201701131556n 2017; Irct2017013132326n 2017; Irct201702171556n 2017; Irct2017030532884n 2017; Irct2017041333406n 2017; Irct201710083551n 2017; Irct20180416039330n 2018; Irct20180503039517n 2018; Irct20180626040242n 2018; Irct20181014041341n 2019; Irct20181211041929n 2019; Irct20190129042536n 2019; Irvine 2019; Isrctn 2003B; ISRCTN 2004; Isrctn 2005B; Isrctn 2006; Isrctn 2010A; Isrctn 2011D; Isrctn 2012B; Isrctn 2013B; Isrctn 2016B; Isrctn 2016C; Isrctn 2016D; Isrctn 2016E; Isrctn 2017B; Isrctn 2017C; Isrctn 2017D; ISRCTN 2017E; Isrctn 2018B; Isrctn 2018D; Isrctn 2019A; ISRCTN 2019B; Isrctn 2019C; Isrctn 2019D; ISRCTN 2020A; ISRCTN 2020B; ISRCTN 2020C; ISRCTN 2020D; Jamison 2017; Janssen-Ortho Inc 1999; Jarusiewicz 2002; Javadfar 2020; Joanne Kurtzberg 2016; Joanne Kurtzberg 2020; Jocelyn 1998; Johnson 2007; Johnson 2013; Johnson 2019; Jones 2020; Jonsson 2019; Jprn 2011; Jprn 2012; Jprn 2016B; Jprn 2018; JPRN-JMA-II 2014; Jprn-Jma-Iia 2010; JPRN-jRCT 2019A; JPRN-jRCT 2019B; JPRN-jRCT 2019C; JPRN-jRCT 2020A; JPRN-jRCT 2020B; Jprn-Umin 2009B; JPRN-UMIN 2009C; Jprn-Umin 2010B; JPRN-UMIN 2011A; Jprn-Umin 2011B; JPRN-UMIN 2011C; JPRN-UMIN 2011D; Jprn-Umin 2012C; Jprn-Umin 2012D; JPRN-UMIN 2012E; Jprn-Umin 2013; JPRN-UMIN 2014B; Jprn-Umin 2014C; JPRN-UMIN 2015A; JPRN-UMIN 2015B; JPRN-UMIN 2016A; JPRN-UMIN 2016B; JPRN-UMIN 2016C; JPRN-UMIN 2016D; Jprn-Umin 2016G; Jprn-Umin 2016H; Jprn-Umin 2017B; JPRN-UMIN 2017C; Jprn-Umin 2018B; Jprn-Umin 2018C; JPRN-UMIN 2018D; JPRN-UMIN 2018E; Jprn-Umin 2018F; Jprn-Umin 2019A; JPRN-UMIN 2019B; JPRN-UMIN 2019C; JPRN-UMIN 2019D; JPRN-UMIN 2020A; JPRN-UMIN 2020B; Jun 2020; K.K 2012; Kaale 2012; Kaluzhny 2020; Kamps 2015; Kanat 2015; Kanat 2017; Kang 2019; Karst 2015A; Kasari 2006; Kasari 2010; Kasari 2012; Kasari 2014; Kasari 2015; Kasari 2016; Kashefimehr 2018; Kaufmann 2013; KCT 2018; Keim 2018B; Keim 2020; Kelly 2017; Kent 2013; Kent 2020; Kenworthy 2014; Kerley 2018; Kern 2013; Kerns 2016A; Khalaj 2018; Khodabakhshi 2015; Kim 2008; King 2009A; Kitzerow 2020; Klaiman 2013; Knivsberg 2002; Ko 2018; Koch 2019; Kolevzon 2014; Kong A; Kong B; Kong C; Kong 2005; Kong 2020A; Koning 2013; Kouijzer 2013; Kretzmann 2015; Kuhlthau 2020; Kumazaki 2017; Kumazaki 2019; Kumazaki 2020; Laboratories 2009; Lagasse 2014; Lamberti 2016; Landa 2011; Lanning 2014; Laugeson 2015; Le Couteur 2012; Leaf 2017; Lecavalier 2017A; Lemonnier 2012; Lerner 2012; Leuven 2015; Leuven 2018; Leventhal 1993; Levine 2016; Levy 2003; Lewis 2009; Li 2016; Li 2017; Li 2019A; Lilly 2006; Lim 2010; Lim 2011; Lim 2019; Lindsay 2006; Ling 2012; Liu 2018; Liu 2019; Liu 2020; Lo 2020; Locke 2019; Loebel 2016; Lomas 2020; Lopata 2010; Lopata 2016; Lopata 2019; Lopata 2020B; Ltd 2020; Luby 2006; Luckhardt 2018B; Ludlow 2020; Lundqvist 2009; Lyon 2014; Mah 2016; Mahoney 2016; Maisel 2019; Mankad 2015; Manohar 2019; Maras 2018; Marcus 2009; Marcus 2011A; Marino 2019; Marino 2020B; Marshall 2016; Martsenkovska 2015; Martsenkovsky 2016; Mastrominico 2018; Matthews 2018; Maximo 2017; Mccabe 2017; Mccracken 2002; Mccracken 2014; Mcdougle 1996A; Mcdougle 1996B; Mcdougle 2005A; Medicine 2004A; Medicine 2007A; Mehling 2018; Mengoni 2017; Mills 2020; Minjarez 2013; Minnesota 2014A; Minnesota 2014B; Minshawi 2016; Miral 2008; Miyajima 2016; Mohammadi 2013; Mohammadzaheri 2014; Momtazmanesh 2020; Moody 2019; Moradi 2020; Moreno 2014; Morgan 2018; Moseley 2019; Mousavinejad 2018; Munasinghe 2010; Munesue 2016; Murdock 2014; Naber 2013; Nagaraj 2006; Nair 2015; National Healthcare Group 2011; Navarro 2009; Navarro 2015; Naviaux 2017; Nct 1999A; Nct 1999B; Nct 2001B; Nct 2002A; Nct 2003B; Nct 2003C; Nct 2004B; Nct 2004C; Nct 2005D; Nct 2005E; Nct 2005F; Nct 2005G; Nct 2005H; Nct 2005I; Nct 2005J; Nct 2005K; Nct 2005L; Nct 2006B; Nct 2006C; Nct 2006D; Nct 2006E; Nct 2006F; Nct 2006G; Nct 2006H; Nct 2006I; Nct 2007A; Nct 2007B; Nct 2007C; Nct 2007D; Nct 2007E; Nct 2007F; Nct 2007G; Nct 2007H; Nct 2007I; Nct 2007J; Nct 2008F; Nct 2008G; Nct 2008H; Nct 2008I; Nct 2008J; Nct 2008K; Nct 2008L; Nct 2008M; Nct 2008N; Nct 2009D; Nct 2009E; Nct 2009F; Nct 2009G; Nct 2009H; Nct 2009I; Nct 2009J; Nct 2009K; Nct 2009a; Nct 2009b; Nct 2010F; Nct 2010G; Nct 2010H; Nct 2010I; Nct 2010J; Nct 2010K; Nct 2010L; Nct 2010M; Nct 2010N; Nct 2010O; Nct 2010P; Nct 2010Q; Nct 2010R; Nct 2010S; Nct 2010T; Nct 2011C; Nct 2011D; Nct 2011E; Nct 2011F; Nct 2011G; Nct 2011H; Nct 2011I; Nct 2011J; Nct 2011K; Nct 2011a; Nct 2011b; Nct 2012A; Nct 2012B; Nct 2012C; Nct 2012D; Nct 2012E; Nct 2012F; Nct 2012G; Nct 2012H; Nct 2012I; Nct 2012J; Nct 2012N; Nct 2012O; Nct 2012a; Nct 2012c; Nct 2013E; Nct 2013F; Nct 2013G; Nct 2013H; Nct 2013I; Nct 2013J; Nct 2013K; Nct 2013L; Nct 2013M; Nct 2013N; Nct 2013O; Nct 2013P; Nct 2013T; Nct 2013a; Nct 2013c; Nct 2013d; Nct 2013f; Nct 2013g; Nct 2014F; Nct 2014G; Nct 2014H; Nct 2014I; Nct 2014J; Nct 2014K; Nct 2014L; Nct 2014M; Nct 2014N; Nct 2014O; Nct 2014P; Nct 2014Q; Nct 2014R; Nct 2014S; Nct 2014T; Nct 2014U; Nct 2014V; Nct 2014W; Nct 2014X; Nct 2014Y; Nct 2014Z; Nct 2014[; Nct 2015E; Nct 2015F; Nct 2015G; Nct 2015H; Nct 2015I; Nct 2015J; Nct 2015K; Nct 2015L; Nct 2015M; Nct 2015N; Nct 2015O; Nct 2015P; Nct 2015Q; Nct 2015R; Nct 2015S; Nct 2015T; Nct 2015U; Nct 2015V; Nct 2015W; Nct 2015X; Nct 2015Y; Nct 2015Z; Nct 2015[; Nct 2015\; Nct 2015]; Nct 2015^; Nct 2015_; Nct 2015`; Nct 2015a; Nct 2015b; Nct 2015c; Nct 2015d; Nct 2015i; Nct 2015a; Nct 2015b; Nct 2015c; Nct 2015d; Nct 2016C; Nct 2016D; Nct 2016E; Nct 2016F; Nct 2016G; Nct 2016H; Nct 2016I; Nct 2016J; Nct 2016K; Nct 2016L; Nct 2016M; Nct 2016N; Nct 2016O; Nct 2016P; Nct 2016Q; Nct 2016R; Nct 2016S; Nct 2016T; Nct 2016U; Nct 2016V; Nct 2016W; Nct 2016X; Nct 2016Y; Nct 2016Z; Nct 2016[; Nct 2016\; Nct 2016]; Nct 2016^; Nct 2016_; Nct 2016`; Nct 2016a; Nct 2016b; Nct 2016c; Nct 2017C; Nct 2017D; Nct 2017E; Nct 2017F; Nct 2017G; Nct 2017H; Nct 2017I; Nct 2017J; Nct 2017K; Nct 2017L; Nct 2017M; Nct 2017N; Nct 2017O; Nct 2017P; Nct 2017Q; Nct 2017R; Nct 2017S; Nct 2017T; Nct 2017U; Nct 2017V; Nct 2017W; Nct 2017X; Nct 2017Y; Nct 2017Z; Nct 2017[; Nct 2017\; Nct 2017]; Nct 2017^; Nct 2017_; Nct 2017`; Nct 2017a; Nct 2017b; Nct 2017c; Nct 2017d; Nct 2017e; Nct 2017a; Nct 2017b; Nct 2017c; Nct 2017d; Nct 2017e; Nct 2017f; Nct 2018B; Nct 2018C; Nct 2018D; Nct 2018E; Nct 2018F; Nct 2018G; Nct 2018H; Nct 2018I; Nct 2018J; Nct 2018K; Nct 2018L; Nct 2018M; Nct 2018N; Nct 2018O; Nct 2018P; Nct 2018Q; Nct 2018R; Nct 2018S; Nct 2018T; Nct 2018U; Nct 2018V; Nct 2018W; Nct 2018X; Nct 2018Y; Nct 2018Z; Nct 2018[; Nct 2018\; Nct 2018]; Nct 2018^; Nct 2018_; Nct 2018`; Nct 2018a; Nct 2018b; Nct 2018c; Nct 2018d; Nct 2018h; Nct 2018a; Nct 2018b; Nct 2019A; Nct 2019B; Nct 2019C; Nct 2019D; Nct 2019E; Nct 2019F; Nct 2019G; Nct 2019H; Nct 2019I; Nct 2019J; Nct 2019K; Nct 2019O; Nct 2019P; Nct 2019Q; Nct 2019R; Nct 2019S; Nct 2019T; Nct 2020A; Nct 2020B; Nct 2020C; Nct 2020D; Nct 2020E; Nct 2020F; Nct 2020G; Nct 2020H; Nct 2020I; Nct 2020J; Nct01013545 2009; Nct01187940 2010; Nct01233414 2010; Nct01734941 2012; Nct01972074 2013; Nct01993251 2013; Nct02147236 2014; Nct02153203 2014; Nct02940574 2016; Nct03310775 2017; Nct03424811 2018; Nct03553875 2018; Nct03778827 2018; Nct03899831 2019; Nct03924973 2019; Nct03957993 2019; Nct03963479 2019; Nct03984487 2019; Nct03984513 2019; Nct03994757 2018; Nct04005547 2018; Nct04016701 2019; Nct04024111 2019; Nct04031755 2019; Nct04042337 2019; Nct04053036 2019; Nct04060017 2019; Nct04060030 2019; Nct04078061 2019; Nct04089579 2019; Nct04114942 2019; Nct04119492 2018; Nct04146428 2019; Nct04166591 2019; Nct04174365 2019; Nct04182633 2019; Nct04209452 2019; Nct04218331 2019; Nct04220086 2019; Nct04233502 2020; Nct04237870 2020; Nct04242355 2020; Nct04244721 2020; Nct04258254 2020; Nct04270708 2020; Nct04276571 2020; Nct04278898 2020; Nct04283045 2020; Nct04293783 2020; Nct04298164 2020; Nct04299464 2020; Nct04312932 2020; Nct04319640 2020; Nct04327648 2020; Nct04440852 2020; Nct04442061 2020; Nct04446442 2019; Nct04452045 2020; Nct04460677 2020; Nct04467073 2020; Nct04501588 2020; Nct04509401 2020; Nct04533607 2020; Nct04551313 2020; Nct04557488 2020; Nct04562688 2020; Nct04574206 2020; Nefdt 2010; Ni 2017; Ni 2019; Niederhofer 2002A; Niederhofer 2002B; Niederhofer 2003A; Niederhofer 2003B; Niederhofer 2004; Nikoo 2015; Nikvarz 2017; no MH outcomes; Noone 2014; Noterdaeme 2013; Novakovic 2019; Novotny 2004A; Nowell 2019; Ntr 2010A; Ntr 2010B; Ntr 2010C; Ntr 2011; Ntr 2013; Ntr 2014A; Ntr 2014B; Ntr 2014C; Ntr 2016A; Ntr 2016B; Ntr 2016a; Ntr 2016b; Ntr 2016c; Ntr 2016d; Ntr 2017A; Ntr 2017B; Olincy 2016; Olivar-Parra 2011; Organization ; Organization 2014; Oriel 2011; Ortiz-Sanchez 2018; Owen 2009; Owens 2008; Ozyurt 2017; Ozyurt 2020; Padmanabha 2019; Pajareya 2011; Pan 2019; Papadopoulos 2019A; Papadopoulos 2019B; Parellada 2017; Park 2020; Parker 2017; Parsons 2019; Pashazadeh Azari 2019; Pearson 2013; Pearson 2020; Pedroza Garcia 2017; Pelphrey 2014; Peters 2014; Peterson 2019; Petrovska 2019; Pfeiffer 2011; Phung 2017; Phung 2019; Picciotto 2018; Pineda 2008; Pittsburgh 2002; Posey 2005; Poslawsky 2015; Powell 2016; Pretzsch 2019A; Prillinger 2020; Pritchard 1987; Procyshyn 2020; Qian 2014; Quintana 2017A; Rabeyron 2020; Rabin 2018; Rahman 2018; Randell 2019A; Ranson 2014; Rbr-6c36pg 2018; Rbr-75bsvk 2020; Rbr-7nq8m7 2011; Rbr-8bfjgk 2018; Rbr-8cq 2015; Rbr-8ttw3f 2018; Rbr-93wgtg 2020; Rbr-9737g8 2020; Reaven 2018; Remington 2001; Research 2020; Riahi 2012B; Rice 2015; Rickards 2007; Rimland 1978; Robb 2011; Roberts 2001; Roberts 2011; Rodgers 2015; Roeyers 1996; Rogers 2019; Rohmann 1985; Rollins 2018; Rosenblau 2019; Rossigno 2009; Ruble 2012; Ruble 2013; Russell 2013; Russell 2019B; Russo-Ponsaran 2016; Ryan 2010; Saad 2015; Sallows 2005; Sampanthavivat 2012; Sanders 2020; Sandler 1999; Santocchi 2016; Scahill 2016; Schroder 2019; Schwartzberg 2013; Sciberras 2015; Scudder 2019; Sharda 2018A; Sharp 2014; Shayestehfar 2013; Shea 2004; Sikich 2013; Sikich 2014; Siller 2014; Silva 2011A; Silva 2011B; Silva 2015; Silver 2001; Silverman 2014; Simpson 2013; Singh 2014; Singh 2018; Singh 2020; Sivakumar 2019; Smith 2014; Smith 2016; Smith 2019; Smith-Sponholz 2015; So 2018; Sofronoff 2005; Sofronoff 2007; Sokhadze 2011; Solomon 2014; Soorya 2015; Souza-Santos 2018; Sponheim 1991; Sprengers 2020; Squibb 2011; Srinivasan 2015; Stivaros 2018; Storch 2014; Strydom 2020B; Sugie 2003; Sukhodolsky 2019; Sun 2018; Tajik-Parvinchi 2019; Tanaka 2010; Tchintcharauli 2018; Tctr 2016; Tctr20191215004 2019; Tctr20200420002 2020; Tctr20200522003 2020; Tctr20200610001 2020; Teitelbaum 2012; Teixeira-Machado 2019; Tellegen 2014; The University of Texas Health Science Center 2019; Thiemann-Bourque 2018; Thomeer 2012; Thomeer 2015; Thomeer 2019; Thompson 2014; Tonge 2006; Tonge 2014; Toscano 2019; Touzet 2017; Tse 2019A; Tse 2019B; Tse 2020; Tsheringla 2018; Tumuluru 2017A; Turner-Brown 2016; Uccheddu 2019; Umbricht 2014A; Umbricht 2014B; University 2004A; University 2008; University 2009; University 2011A; University 2011B; University 2012; University 2015A; University 2015B; University 2016A; University 2016B; University 2016C; University 2018A; University 2018B; University 2019A; University 2020A; University Hospital 2010; University of California 2005A; University of California 2008; University of California 2010; University of California 2012; University of California 2015; University of Colorado 2012; University of North Carolina 2006; University of North Carolina 2017; Urbano 2015; Valeri 2019; van der Meer 2013; Van Hecke 2015; Van Steenburgh 2017; Varley 2019; Varni 2012 A; Varni 2012 B; Vasconcelos 2014; Vause 2017; Vause 2020; Veenstra-Vanderweele 2017; Verbaten 1996; Vernon 2019; Visser 2013; Visser 2017; Vivanti 2019; Voss 2019A; Wagner 2019; Wang 2007; Wang 2019; Wasserman 2006; Waugh 2015; Webb 2019; Wehman 2019; Weitlauf 2020; Welterlin 2010; Whitehouse 2017; Whiteley 2010; Whittingham 2009A; Whittingham 2009B; Widjaja 2012; Wijker 2019B; Wijker 2020; Williams 2002; Williams 2012; Williams 2020; Wilson 2020; Wink 2016; Wink 2018; Wink 2019B; Winter 2019; Wirojanan 2009; Wolters 2016; Wong 2010A; Wong 2010B; Wong 2013; Wong 2014; Wong 2020; Wongpakaran 2017; Woo 2013; Woo 2015; Woodard 2007; Wright 2011; Wuang 2010; Xiao 2014; Xu 2017; Yamada 2012; Yang 2014; Yatawara 2016; Yoder 1988; Young 2012; Young 2015; Young 2019; Yuan 2013; Yun 2017; Zamzow 2014; Zamzow 2017; Zhang 2009; Zhang 2012; Zhao 2015; Zhao 2018; Zheng 2020; Zingarelli 1992B; Zody 2018 |

Note: we have listed study name here. Full references for these studies can be found in Appendix 8. Please note that some exclusions were based on abstracts, when only the conference abstracts were available. Five references were excluded on the basis of abstracts as full texts were not available (Anonymous 1980; Collard 1969; Decocq 1996; Rollins 2018; Zhao 2015).

### Table 2B: Additional reports of included and excluded studies

| Number of studies | Study name |
| --- | --- |
| 349 | Actrn 2008B; Actrn 2014C; Actrn 2017F; ACTRN 2019D; ACTRN 2019E; ACTRN 2019F; ACTRN 2019G; ACTRN 2019H; Afsharnejad 2019; Ajram 2015A; Alaerts 2019; Alaerts 2020B; Albaum 2019; Althaus 2016; Aman 2009A; Aman 2010; Ameis 2017A; Ameis 2017B; Ameis 2018; Amminger 2007A; Anagnostou 2006B; Anagnostou 2006C; Anagnostou 2013D; Anagnostou 2018; Anonymous 2017; Anonymous4; Anonymous9; Arnold 2010; Baghdadli 2010; Beaumont 2008B; Beaumont 2018A; Bekhet 2017B; Bettison 1997; Bolognani 2019; Brookma-Frazee 2019; Brookman-Frazee 2018; Cheng 2018A; Chez 2016; Choque Olsson 2016; Chugani 2009; Cidav 2017; Clinical 2014B; Cook 2019; Corbett 2016B; Da Paz 2017A; Da Paz 2017B; Dawalt 2017; Desarkar 2017; Doble 2017; Domes 2014; Drahota 2011; DRKS 2015C; Drks 2019D; Drks 2019E; Enticott 2015; EUCTR 2018D; Euctr 2018E; Euctr 2019; Euctr-024202-34-De 2013; Florida 2009; Florida 2010; Florida 2014; Foundation 2004; Frank 2020; Frankel 2010B; Freitag 2013; Freitag 2015; Fridenson-Hayo 2017B; Fujii 2013; Geretsegger 2012; Grahame 2014; Grob 2014; Guastella 2012; Harfterkamp 2013; Hawkins 2019; Health 1997; Health 1999; Health 2001; Health 2006A; Health 2012B; Hollander 2006A; Hollander 2013A; Hollander 2014A; Hollander 2014B; Hollander 2014C; Hollander 2020; Hollway 2019; Hospital 2006; Hospital 2010; Hospital 2015A; Hospital 2017B; Hugo W. Moser Research Institute at Kennedy Krieger 2012; Iadarola 2017; Ibanez 2018A; Ingersoll 2012B; IRCT 2019M; IRCT 2019N; Irct 2019O; Irct 2019P; Irct 2019Q; Irct138711091556n 2009; Ireri 2019; ISRCTN 2003A; ISRCTN 2005A; Isrctn 2008A; Isrctn 2008B; Isrctn 2010B; Isrctn 2010C; Isrctn 2011A; Isrctn 2011B; Isrctn 2012A; Isrctn 2012C; Isrctn 2013A; Isrctn 2015; Isrctn 2016A; Isrctn 2016G; Isrctn 2017A; ISRCTN 2018A; ISRCTN 2018E; Isrctn 2018F; Isrctn 2019E; Isrctn 2019F; JapicCTI 2012; Jonsson 2018; Jprn 2014; Jprn-Japiccti ; JPRN-JapicCTI 2012; JPRN-UMIN 2009A; JPRN-UMIN 2010A; JPRN-UMIN 2012A; JPRN-UMIN 2012B; Jprn-Umin 2012F; JPRN-UMIN 2014A; Jprn-Umin 2014E; Jprn-Umin 2015D; Jprn-Umin 2016E; Jprn-Umin 2016F; JPRN-UMIN 2017A; JPRN-UMIN 2018A; Jprn-Umin 2018G; JPRN-UMIN 2018H; Kaluzhny 2019; Karst 2014; Karst 2015B; Keim 2018A; Keim 2018C; Kendall 2014; Kerns 2016B; Kim 2009; King 2009B; King 2013; Kong 2020B; Kouijzer 2010; Kroeger 2007; Lang 2010; Langdon 2013; Leboyer 1992; Lecavalier 2017B; Lee 2019; Li 2019B; Locke 2018; Lopata 2018; Lopata 2020A; Luckhardt 2018A; Maddox 2017; Marcus 2011B; Marino 2020A; Martsenkovsky 2015; Maskey 2019; Matthews 2020; Mcconachie 2011; Mccracken 2005B; Mcdougle 2005B; Mcnally Keehn 2011; Mcvey 2016; Mohammadzaheri 2015; Nct 2000A; Nct 2000B; Nct 2001A; Nct 2002B; Nct 2003A; Nct 2003D; Nct 2004A; Nct 2004D; Nct 2004E; Nct 2005A; Nct 2005B; Nct 2005C; Nct 2005N; Nct 2005O; Nct 2005P; Nct 2006A; Nct 2006J; Nct 2006K; Nct 2006L; Nct 2006M; Nct 2008A; Nct 2008B; Nct 2008C; Nct 2008D; Nct 2008E; Nct 2008P; Nct 2008Q; Nct 2009A; Nct 2009B; Nct 2009C; Nct 2009M; Nct 2009N; Nct 2009O; Nct 2010A; Nct 2010B; Nct 2010C; Nct 2010D; Nct 2010E; Nct 2010U; Nct 2010V; Nct 2010W; Nct 2010X; Nct 2010Y; Nct 2011A; Nct 2011B; Nct 2012K; Nct 2012L; Nct 2012M; Nct 2013A; Nct 2013B; Nct 2013C; Nct 2013D; Nct 2013R; Nct 2013S; Nct 2014A; Nct 2014B; Nct 2014C; Nct 2014D; Nct 2014E; Nct 2014^; Nct 2014_; Nct 2014`; Nct 2015A; Nct 2015B; Nct 2015C; Nct 2015D; Nct 2015g; Nct 2015h; Nct 2016A; Nct 2016B; Nct 2016d; Nct 2016e; Nct 2017A; Nct 2017B; Nct 2018A; Nct 2018e; Nct 2018f; Nct 2019L; Nct 2019M; Nct 2019N; Nct00080145 2004; Nct01784276 2013; Nct02275715 2014; Nct02995408 2016; Nct03570372 2018; Nct03620097 2018; Nct03785327 2018; Nct03947086 2019; Nct04145076 2019; Nct04204226 2019; Nct04246398 2020; Nct04259671 2020; Nct04295512 2020; Nct04339153 2020; Nct04472780 2020; Nct04517799 2020; Nct04520685 2020; Nct04545606 2020; Nefdt 2007; Novotny 2004B; Ntr294 2005; Pandina 2007; Papadopoulos 2015; Parellada 2015; Paris 2020; Parsons 2020; Pedersen 2014; Pellecchia 2016; Petty 2017; Phung 2018; Preckel 2016; Pretzsch 2019B; Pretzsch 2019C; Pretzsch 2019D; Quintana 2017B; Quintana 2017C; Randell 2019B; Reddihough 2019; Rickards 2009; Roche 2020; Rodgers 2013; Rodgers Jonathan 2014; Russell 2017; Russell 2019A; Russell 2020; Rutgers 2019; Salt 2002B; Scahill 2001A; Scahill 2011; Scahill 2015; Schiltz 2017; Schiltz 2018A; Schiltz 2018B; Servier 2018; Sharda 2018B; Siller 2018; Simmons 2020; So 2019; So 2020; Sofronoff 2004; Storch 2013; Strydom 2020A; Studies 2014A; Studies 2014B; Tctr20161103002 2016; The University of Texas Health Science Center 2016; Thiemann-Bourque 2019; Tumuluru 2017B; Turner-Brown 2019; University 2019B; University 2020B; University 2020C; University of California 2005B; University of California 2005C; University of California 2020; University of Colorado 2020; Urbano 2013; Vasilevska Petrovska 2019; Visser 2015; Voss 2019B; Waugh 2017; Wehman 2014; White 2013; White 2014A; White 2014B; Wijker 2019A; Wink 2019A; Wood 2009; Wood 2014; Wright 2014; Yamasue 2018; Zamzow 2016; Zingarelli 1992A |

Note: we have listed study name here. Full references for these studies can be found in Appendix 8.

### Table 3: List of randomised controlled trials in which mental health outcomes were not measured at all and list of randomised controlled trials in which mental health outcomes were measured but not reported at all or not reported in an analysable format

| Reason | Number of studies | Study name |
| --- | --- | --- |
| Mental health outcomes not measured | 674 | Actrn 2009B; Actrn 2009C; Actrn 2010B; Actrn 2010C; Actrn 2011A; Actrn 2011B; Actrn 2011C; Actrn 2012A; Actrn 2015A; Actrn 2015B; Actrn 2016A; Actrn 2016B; Actrn 2016D; Actrn 2016F; Actrn 2018A; Anagnostou 2010A; Anagnostou 2011; Anagnostou 2016B; Anonymous10; Anonymous3; Anonymous6; Anonymous7; Arkansas 2012; As 2015; Bangabandhu 2019; Bowrin 2020; Brainsway 2013; Carolina 2011; Carr 2016; Center 2002; Center 2011; Center 2014; Center 2017; Center for Autisme 2006; Chi 2012; Chi 2017; Chictr 2018A; Chictr 2018B; Chictr 2018C; Chictr 2018D; Chictr 2018E; Chictr 2018F; Chictr 2018G; Chictr 2018H; ChiCTR 2019A; ChiCTR 2019B; ChiCTR 2019C; ChiCTR 2019D; ChiCTR 2019E; ChiCTR 2019F; ChiCTR 2020A; ChiCTR 2020B; ChiCTR 2020C; ChiCTR 2020D; ChiCTR 2020E; ChiCTR 2020F; ChiCTR 2020G; ChiCTR 2020H; ChiCTR 2020I; ChiCTR 2020J; ChiCTR 2020K; ChiCTR 2020L; ChiCTR 2020M; Chictr-Iir 2016; Chictr-Inr 2017; Chictr-Ior 2016; Chictr-Ior 2017A; Chictr-Ior 2017B; Chictr-Ipr 2016; Chictr-Ipr 2017A; Chictr-Ipr 2017B; Chictr-Ipr 2017C; Chictr-Trc 2009; Chictr-Trc 2014; Children 1999; Children's Hospital Medical Center 2013A; Children's Hospital Medical Center 2013B; Chugani 2004; Coronado Biosciences 2014; Corporation 2002A; Corporation 2002B; Council 2009A; CTRI 2015; Ctri 2017C; Ctri 2019A; CTRI 2019C; Curemark 2009; Curemark 2015A; Development 2003; Development 2006A; Drks 2012; DRKS 2015A; DRKS 2015B; DRKS 2016A; DRKS 2016B; DRKS 2018; DRKS 2019A; DRKS 2019B; Drks 2019C; DRKS 2020; EU/EEA 2017; Euctr 2006; Euctr 2007; EUCTR 2013; Euctr 2018A; EUCTR 2018B; Euctr 2018C; Euctr-000106-11-Fr 2016; Euctr-000586-45-Be 2014; Euctr-000955-25-Fr 2015; Euctr-001230-17-Fr 2015; Euctr-001320-31-Outside-Eu/Eea 2015; Euctr-001560-35-Nl 2016; Euctr-001689-97-Fr 2009; Euctr-003259-39-Es 2014; Euctr-003712-36-Fr 2008; Euctr-003750-89-De 2012; Euctr-004419-38-De 2018; Euctr-004420-30-Es 2018; Euctr-006126-25-Fr 2007; Euctr-006444-21-Es 2010; Euctr-010393-38-Fr 2009; Euctr-012102-39-It 2009; Euctr-018740-13-Nl 2010; Euctr-022511-18-De 2010; Factor 2019; Health 2012A; Hospital 2012; Hospital 2013; Hospital 2014A; Hospital 2018; IRCT 2017; IRCT 2018A; Irct 2018B; Irct 2019A; IRCT 2019B; IRCT 2019C; IRCT 2019D; Irct 2019E; IRCT 2019F; IRCT 2019G; IRCT 2019H; IRCT 2019I; IRCT 2019J; IRCT 2019K; IRCT 2019L; IRCT 2020A; IRCT 2020B; IRCT 2020C; IRCT 2020E; IRCT 2020G; IRCT 2020I; IRCT 2020J; IRCT 2020L; IRCT 2020M; Irct1138901151556n 2010; Irct138711161556n 2009; Irct138808202698n 2010; Irct138901141556n 2010; Irct138904204264n 2010; Irct20090117001556n 2017; Irct20090117001556n 2018A; Irct20090117001556n 2018B; Irct20090117001556n 2018C; Irct20090117001556n 2018D; Irct201012031556n 2010; Irct201101105280n 2011; Irct201106101556n 2011; Irct201106103930n 2011; Irct201107281556n 2011; Irct201108155280n 2013; Irct201110233930n 2011; Irct201110281556n 2011; Irct201202281556n 2012; Irct201204037202n 2012; Irct201204081556n 2012; Irct201204246834n 2012; Irct201205259854n 2012; Irct2012091010806n 2013; Irct2012111011421n 2013; Irct201212079854n 2012; Irct201302201556n 2013; Irct201307303930n 2014; Irct20131013014994n 2018; Irct2013110915339n 2016; Irct201402043930n 2014; Irct20140212016564n 2018; Irct201404212017n 2014; Irct2014102519665n 2014; Irct20150519022323n 2017; Irct2015080223454n 2015; Irct201512081556n 2015; Irct2015122625699n 2016; Irct201512315280n 2016; Irct201602041556n 2016; Irct2016022826802n 2016; Irct2016061711689n 2016A; Irct2016061711689n 2016B; Irct2016061828511n 2016; Irct2016071728966n 2016; Irct2016082215339n 2016; Irct2016100330113n 2017; Irct20161210031330n 2018; Irct201701131556n 2017; Irct2017013132326n 2017; Irct201702171556n 2017; Irct2017030532884n 2017; Irct2017041333406n 2017; Irct201710083551n 2017; Irct20180416039330n 2018; Irct20180503039517n 2018; Irct20180626040242n 2018; Irct20181014041341n 2019; Irct20181211041929n 2019; Irct20190129042536n 2019; Isrctn 2012B; Isrctn 2016E; Isrctn 2018D; ISRCTN 2020A; ISRCTN 2020C; ISRCTN 2020D; Jamison 2017; Jarusiewicz 2002; Javadfar 2020; Joanne Kurtzberg 2016; Jocelyn 1998; Johnson 2007; Johnson 2013; Johnson 2019; Jones 2020; Jonsson 2019; Jprn 2011; Jprn 2012; Jprn 2018; JPRN-JMA-II 2014; Jprn-Jma-Iia 2010; JPRN-jRCT 2019A; JPRN-jRCT 2019B; JPRN-jRCT 2020B; Jprn-Umin 2009B; Jprn-Umin 2010B; JPRN-UMIN 2011A; Jprn-Umin 2012C; JPRN-UMIN 2014B; JPRN-UMIN 2015A; JPRN-UMIN 2016A; JPRN-UMIN 2016B; Jprn-Umin 2016H; Jprn-Umin 2017B; JPRN-UMIN 2017C; Jprn-Umin 2018B; Jprn-Umin 2018C; JPRN-UMIN 2018D; JPRN-UMIN 2018E; Jprn-Umin 2019A; JPRN-UMIN 2019B; JPRN-UMIN 2019C; JPRN-UMIN 2020A; K.K 2012; Kaale 2012; Kamps 2015; Kanat 2015; Kang 2019; Karst 2015A; Kasari 2006; Kasari 2010; Kasari 2012; Kasari 2014; Kasari 2015; Kasari 2016; Kashefimehr 2018; Keim 2018B; Kelly 2017; Kent 2013; Kent 2020; Kenworthy 2014; Kerley 2018; Kern 2013; Khalaj 2018; Kim 2008; King 2009A; Klaiman 2013; Ko 2018; Koch 2019; Kolevzon 2014; Kong A; Kong B; Kong C; Kong 2005; Koning 2013; Kouijzer 2013; Kretzmann 2015; Kuhlthau 2020; Kumazaki 2017; Kumazaki 2019; Kumazaki 2020; Laboratories 2009; Lagasse 2014; Lamberti 2016; Landa 2011; Laugeson 2015; Leaf 2017; Lemonnier 2012; Lerner 2012; Leuven 2015; Leuven 2018; Leventhal 1993; Levy 2003; Li 2016; Li 2017; Li 2019A; Lilly 2006; Lim 2010; Lim 2011; Lim 2019; Lindsay 2006; Ling 2012; Liu 2018; Liu 2019; Liu 2020; Lo 2020; Locke 2019; Loebel 2016; Lomas 2020; Lopata 2010; Lopata 2016; Lopata 2019; Lopata 2020B; Ltd 2020; Luckhardt 2018B; Ludlow 2020; Lundqvist 2009; Lyon 2014; Mah 2016; Mahoney 2016; Mankad 2015; Maras 2018; Marcus 2011A; Marino 2019; Marshall 2016; Martsenkovska 2015; Martsenkovsky 2016; Mastrominico 2018; Maximo 2017; Mccabe 2017; Mccracken 2002; Mccracken 2014; Mcdougle 2005A; Medicine 2004A; Medicine 2007A; Mehling 2018; Minjarez 2013; Minshawi 2016; Miral 2008; Miyajima 2016; Mohammadi 2013; Mohammadzaheri 2014; Moody 2019; Moreno 2014; Morgan 2018; Mousavinejad 2018; Munasinghe 2010; Munesue 2016; Murdock 2014; Naber 2013; Nagaraj 2006; Nair 2015; National Healthcare Group 2011; Navarro 2009; Navarro 2015; Naviaux 2017; Nct 1999A; Nct 1999B; Nct 2001B; Nct 2002A; Nct 2003B; Nct 2005D; Nct 2005E; Nct 2005F; Nct 2005G; Nct 2005H; Nct 2005I; Nct 2005J; Nct 2005K; Nct 2005L; Nct 2006B; Nct 2006C; Nct 2006D; Nct 2006E; Nct 2006F; Nct 2006G; Nct 2006H; Nct 2006I; Nct 2007A; Nct 2007B; Nct 2007C; Nct 2007D; Nct 2007E; Nct 2007F; Nct 2007G; Nct 2007H; Nct 2007I; Nct 2007J; Nct 2008F; Nct 2008G; Nct 2008H; Nct 2008I; Nct 2008J; Nct 2008K; Nct 2008L; Nct 2008M; Nct 2009D; Nct 2009E; Nct 2009F; Nct 2009G; Nct 2009H; Nct 2009I; Nct 2009J; Nct 2009K; Nct 2010F; Nct 2010G; Nct 2010H; Nct 2010I; Nct 2010J; Nct 2010K; Nct 2010L; Nct 2010M; Nct 2010N; Nct 2010O; Nct 2010P; Nct 2010Q; Nct 2011C; Nct 2011D; Nct 2011E; Nct 2011F; Nct 2011G; Nct 2011H; Nct 2011I; Nct 2011J; Nct 2011K; Nct 2012A; Nct 2012B; Nct 2012C; Nct 2012D; Nct 2012E; Nct 2012F; Nct 2012G; Nct 2012H; Nct 2012I; Nct 2012O; Nct 2013E; Nct 2013F; Nct 2013G; Nct 2013H; Nct 2013I; Nct 2013J; Nct 2013K; Nct 2013L; Nct 2013M; Nct 2013N; Nct 2013T; Nct 2014F; Nct 2014G; Nct 2014H; Nct 2014I; Nct 2014J; Nct 2014K; Nct 2014L; Nct 2014M; Nct 2014N; Nct 2014O; Nct 2014P; Nct 2014Q; Nct 2014R; Nct 2014S; Nct 2014T; Nct 2014U; Nct 2014V; Nct 2014W; Nct 2014X; Nct 2014Y; Nct 2015E; Nct 2015F; Nct 2015G; Nct 2015H; Nct 2015I; Nct 2015J; Nct 2015K; Nct 2015L; Nct 2015M; Nct 2015N; Nct 2015O; Nct 2015P; Nct 2015Q; Nct 2015R; Nct 2015S; Nct 2015T; Nct 2015U; Nct 2015V; Nct 2015W; Nct 2015X; Nct 2015Y; Nct 2015Z; Nct 2015[; Nct 2015\; Nct 2015i; Nct 2016E; Nct 2016F; Nct 2016G; Nct 2016H; Nct 2016I; Nct 2016J; Nct 2016K; Nct 2016L; Nct 2016M; Nct 2016N; Nct 2016O; Nct 2016P; Nct 2016Q; Nct 2016R; Nct 2016S; Nct 2016T; Nct 2016U; Nct 2016V; Nct 2016W; Nct 2016X; Nct 2016Y; Nct 2016Z; Nct 2017C; Nct 2017D; Nct 2017E; Nct 2017F; Nct 2017G; Nct 2017H; Nct 2017I; Nct 2017J; Nct 2017K; Nct 2017L; Nct 2017M; Nct 2017N; Nct 2017O; Nct 2017P; Nct 2017Q; Nct 2017R; Nct 2017S; Nct 2017T; Nct 2017U; Nct 2018B; Nct 2018C; Nct 2018D; Nct 2018E; Nct 2018F; Nct 2018G; Nct 2018H; Nct 2018I; Nct 2018J; Nct 2018Z; Nct 2018^; Nct 2018h; Nct 2019B; Nct 2019G; Nct 2020G; Nct01013545 2009; Nct01233414 2010; Nct01734941 2012; Nct01972074 2013; Nct01993251 2013; Nct02147236 2014; Nct02153203 2014; Nct02940574 2016; Nct03424811 2018; Nct03957993 2019; Nct04031755 2019; Nct04053036 2019; Nct04209452 2019; Nct04298164 2020; Nct04440852 2020; Nct04467073 2020; Nikoo 2015; Noterdaeme 2013; Olivar-Parra 2011; Papadopoulos 2019A; Parellada 2017; Parker 2017; Pelphrey 2014; Phung 2017; Pittsburgh 2002; Poslawsky 2015; Quintana 2017A; Rabin 2018; Ranson 2014; Reaven 2018; Remington 2001; Rickards 2007; Rimland 1978; Roberts 2011; Rodgers 2015; Roeyers 1996; Rogers 2019; Ruble 2012; Russell 2013; Russo-Ponsaran 2016; Saad 2015; Santocchi 2016; Scahill 2016; Schwartzberg 2013; Shea 2004; Sikich 2014; Siller 2014; Silva 2011A; Silva 2011B; Simpson 2013; Singh 2014; Singh 2018; Smith 2014; Smith 2016; Smith-Sponholz 2015; Sofronoff 2005; Tctr20200420002 2020; Tctr20200610001 2020; Tellegen 2014; The University of Texas Health Science Center 2019; Tonge 2006; Tonge 2014; Turner-Brown 2016; Umbricht 2014A; University 2004A; University 2009; University 2011A; University 2011B; University 2016A; University 2016B; University 2016C; University 2018A; University 2018B; University 2019A; University Hospital 2010; University of California 2005A; University of California 2008; University of California 2010; University of California 2012; University of North Carolina 2006; University of North Carolina 2017; Van Hecke 2015; Vasconcelos 2014; Vause 2017; Veenstra-Vanderweele 2017; Visser 2013; Wasserman 2006; Waugh 2015; Wehman 2019; Whitehouse 2017; Whittingham 2009A; Whittingham 2009B; Williams 2002; Williams 2012; Wink 2016; Wink 2018; Wirojanan 2009; Wolters 2016; Wong 2010A; Wong 2010B; Wong 2013; Wong 2014; Wongpakaran 2017; Woo 2013; Woo 2015; Wuang 2010; Yatawara 2016; Yoder 1988; Young 2012; Young 2015; Zamzow 2014; Zhao 2018 |
| Mental health outcomes not reported or not analysable | 491 | Aaronson 2018; Actrn 2011D; Actrn 2013B; Actrn 2015D; Actrn 2017C; Adams 2009; Adams 2011; Adams 2012; Adams 2018; Adibsereshki 2015; Aghababaei 2020; Ajram 2015B; Akabogu 2019; Akhondzadeh 2008; Alaerts 2020A; Albaum 2020; Aldred 2004; Alolaby 2020; Alquraini 2019; Althaus 2015; Alvares 2019; Aman 2005; Aman 2009B; Aman 2015; Aman 2017; Aman 2018; Amatachaya 2015; Ameis 2020; Amminger 2007B; Anagnostou 2006A; Anagnostou 2012; Anagnostou 2013A; Anagnostou 2013B; Anagnostou 2014; Anagnostou 2016A; Anderson 1989; Anderson 2009; Anonymous 2005A; Anonymous 2005B; Anonymous 2011; Anonymous 2016A; Anonymous 2016B; Antonini 2017; Aoki 2014; Arabi 2019; Arnold 2003; Arnold 2006; Arnold 2012; Arnold 2018; Arnold 2019; Asadabadi 2013; Auyeung 2015; Ayatollahi 2020; Bader 2006; Bagaiolo 2017; Ballester 2015; Ballester 2019; Barthelemy 1989; Bauminger-Zviely 2020; Bearss 2015; Beaumont 2008A; Beaumont 2018B; Begeer 2011; Begeer 2015; Behmanesh 2019; Bekhet 2017A; Belsito 2001; Bent 2014; Benton 2011; Bernaerts 2017; Bernard-Opitz 2004; Bertoglio 2010; Bettison 1996; Beversdorf 2011; Beversdorf 2014; Beversdorf 2015; Bieleninik 2017; Black 2012; Bolte 2016; Bonnot 2015A; Bordini 2020; Borgi 2016; Borowiak 2020; Bouvard 1995; Boyd 2018; Bradshaw 2019; Brian 2017; Brito 2020; Brookma-Frazee 2020; Brunero 2009; Buchsbaum 2001; Buitelaar 1992; Byford 2015; Campbell 1978; Campbell 1982; Campbell 1990; Carey 2016; Cariveau 2019; Carminati 2016; Carrick 2018; Carter 2011; Castorina 2011; Cermak 2015; Chan 2013; Chen 2020; Cheng 2018B; Chester 2019; Chez 2018; Choque Olsson 2017; Chugani 2016; Cibrian 2020; Coggins 1988; Conner 2013; Corbett 2008; Corbett 2016A; Corbett 2019; Costescu 2017; Crawford 2017; Crowell 2020; Da Paz 2016; Dadds 2014; Daly 2012; Danfors 2005; Dawalt 2018; Dawson 2010; de Jong 2019; Dean 2020; Deckers 2016; Dekker 2014; Del Valle Rubido 2015; Derosier 2011; Desarkar 2020; Divan 2019; Doernberg 2020; Domes 2013; Eack 2018; Edelson 1999; Enticott 2014; Erickson 2014; Esse Wilson 2018; Eugene Arnold 2019; Falissard 2019; Fang 2018; Feldman 1999; Felzer-Kim 2020; Field 1997; Findling 2014; Fisher 2005; Fisher 2020; Frankel 2010A; Frazier 2017; Freitag 2016; Fridenson-Hayo 2017A; Frolli 2020; Frye 2018; Fung 2014; Furukawa 2018; Gabis 2019; Gabriels 2015; Gabriels 2018; Gantman 2012; Gao 2020; Garcia-Villamisar 2011; Garcia-Villamisar 2017; Gengoux 2019; Geretsegger 2016; Germone 2019; Gev 2017; Ghaleiha 2013; Ghaleiha 2014; Ghalichi 2016; Ghanizadeh 2013; Ghodsi 2019; Giarelli 2005; Ginn 2017; Glod 2013; Golan 2010; Gonzalez-Domenech 2020; Goods 2013; Gordon 1992; Gordon 1993; Gordon 2011; Gordon 2015; Gordon 2016; Grahame 2015; Gringras 2014; Gringras 2017; Guastella 2010; Guastella 2015; Gulsrud 2007; Haakonsen Smith 2018; Hadwin 1996; Hagerman 2018; Hagner 2012; Hajizadeh-Zaker 2018; Halas 2016; Handen 2009; Handen 2011; Hannant 2019; Hardan 2012; Hardan 2015; Hardan 2016; Hardan 2019; Harfterkamp 2014; Hellings 2001; Hellings 2005; Hendouei 2020; Hendren 2016; Herscu 2020; Ho 2020; Ho Yan 2018; Hochhauser 2018; Hodgetts 2011; Hollander 2003; Hollander 2005; Hollander 2006B; Hollander 2006C; Hollander 2007; Hollander 2010; Hollander 2012; Hollander 2013B; Hollander 2014D; Hollander 2018; Hollander 2019; Hollway 2018; Holopainen 2018; Hopkins 2011; Howlin 2007; Iadarola 2018A; Ibanez 2018B; Inc 2016; Ingersoll 2012A; Ingersoll 2016; Institute 2018; Irvine 2019; Isrctn 2003B; ISRCTN 2004; Isrctn 2005B; Isrctn 2006; Isrctn 2010A; Isrctn 2013B; Janssen-Ortho Inc 1999; JPRN-jRCT 2019C; JPRN-UMIN 2009C; Jprn-Umin 2011B; JPRN-UMIN 2011C; JPRN-UMIN 2011D; Jprn-Umin 2012D; JPRN-UMIN 2012E; Jprn-Umin 2014C; JPRN-UMIN 2015B; JPRN-UMIN 2016C; JPRN-UMIN 2016D; Jprn-Umin 2018F; JPRN-UMIN 2019D; JPRN-UMIN 2020B; Jun 2020; Kanat 2017; Kaufmann 2013; Khodabakhshi 2015; Knivsberg 2002; Lanning 2014; Lecavalier 2017A; Lewis 2009; Luby 2006; Maisel 2019; Manohar 2019; Marcus 2009; Marino 2020B; Matthews 2018; Mcdougle 1996A; Mcdougle 1996B; Mills 2020; Momtazmanesh 2020; Moradi 2020; Moseley 2019; Nct 2003C; Nct 2004B; Nct 2004C; Nct 2008N; Nct 2009a; Nct 2009b; Nct 2010R; Nct 2010S; Nct 2011a; Nct 2011b; Nct 2012a; Nct 2012c; Nct 2013a; Nct 2013c; Nct 2013d; Nct 2013f; Nct 2013g; Nct 2014Z; Nct 2014[; Nct 2015]; Nct 2015a; Nct 2015b; Nct 2015c; Nct 2015d; Nct 2016C; Nct 2016D; Nct 2016a; Nct 2016b; Nct 2016c; Nct 2017a; Nct 2017b; Nct 2017c; Nct 2017d; Nct 2017e; Nct 2017f; Nct 2018Y; Nct 2018\; Nct 2018b; Nct 2018b; Nct 2019A; Nct 2019C; Nct 2019H; Nct 2019I; Nct 2019K; Nct03310775 2017; Nefdt 2010; Ni 2017; Ni 2019; Niederhofer 2002A; Niederhofer 2002B; Niederhofer 2003A; Niederhofer 2003B; Niederhofer 2004; Nikvarz 2017; no MH outcomes; Noone 2014; Novakovic 2019; Novotny 2004A; Nowell 2019; Ntr 2010A; Ntr 2010B; Ntr 2010C; Ntr 2011; Ntr 2013; Ntr 2014A; Ntr 2014B; Ntr 2014C; Ntr 2016A; Ntr 2016B; Ntr 2016a; Ntr 2016b; Ntr 2016c; Ntr 2016d; Ntr 2017A; Ntr 2017B; Olincy 2016; Organization ; Organization 2014; Oriel 2011; Ortiz-Sanchez 2018; Owen 2009; Owens 2008; Ozyurt 2017; Ozyurt 2020; Padmanabha 2019; Pajareya 2011; Pan 2019; Park 2020; Parsons 2019; Pashazadeh Azari 2019; Pearson 2013; Pearson 2020; Pedroza Garcia 2017; Peters 2014; Peterson 2019; Petrovska 2019; Pfeiffer 2011; Phung 2019; Picciotto 2018; Pineda 2008; Posey 2005; Powell 2016; Pretzsch 2019A; Prillinger 2020; Pritchard 1987; Procyshyn 2020; Qian 2014; Rabeyron 2020; Riahi 2012B; Rice 2015; Robb 2011; Roberts 2001; Rohmann 1985; Rollins 2018; Rosenblau 2019; Rossigno 2009; Ruble 2013; Russell 2019B; Ryan 2010; Sallows 2005; Sampanthavivat 2012; Sanders 2020; Sandler 1999; Schroder 2019; Sciberras 2015; Scudder 2019; Sharda 2018A; Sharp 2014; Shayestehfar 2013; Sikich 2013; Silva 2015; Silver 2001; Singh 2020; Sivakumar 2019; So 2018; Sofronoff 2007; Sokhadze 2011; Solomon 2014; Soorya 2015; Souza-Santos 2018; Sponheim 1991; Sprengers 2020; Squibb 2011; Srinivasan 2015; Stivaros 2018; Storch 2014; Strydom 2020B; Sugie 2003; Tajik-Parvinchi 2019; Tchintcharauli 2018; Tctr 2016; Teitelbaum 2012; Teixeira-Machado 2019; Thiemann-Bourque 2018; Thomeer 2012; Thomeer 2015; Thomeer 2019; Thompson 2014; Toscano 2019; Touzet 2017; Tse 2019A; Tse 2019B; Tse 2020; Tsheringla 2018; Tumuluru 2017A; Uccheddu 2019; Umbricht 2014B; University 2008; University 2012; University 2015A; University 2015B; University of California 2015; University of Colorado 2012; Urbano 2015; Valeri 2019; van der Meer 2013; Van Steenburgh 2017; Varni 2012 A; Varni 2012 B; Vause 2020; Verbaten 1996; Vernon 2019; Visser 2017; Vivanti 2019; Voss 2019A; Wagner 2019; Wang 2007; Wang 2019; Webb 2019; Weitlauf 2020; Welterlin 2010; Whiteley 2010; Widjaja 2012; Wijker 2019B; Wijker 2020; Williams 2020; Wilson 2020; Wink 2019B; Wong 2020; Woodard 2007; Wright 2011; Xiao 2014; Xu 2017; Yamada 2012; Yang 2014; Young 2019; Yuan 2013; Yun 2017; Zamzow 2017; Zhang 2009; Zhang 2012; Zhao 2015; Zheng 2020; Zingarelli 1992B; Zody 2018 |
| Ongoing studies with no mental health outcomes | 209 | Actrn 2008A; Actrn 2009A; Actrn 2010A; Actrn 2012B; Actrn 2013A; Actrn 2014A; Actrn 2015C; Actrn 2016C; Actrn 2016E; Actrn 2017A; Actrn 2017B; ACTRN 2017D; Actrn 2018B; Actrn 2019A; Actrn 2019B; Actrn 2019C; Center 2010; Center 2016; Center 2020A; Center 2020B; Central Hospital 2015; Clinical 2014A; Ctri 2016; Ctri 2017A; Ctri 2017B; CTRI 2017D; Ctri 2018A; Ctri 2018B; Ctri 2019B; CTRI 2019D; CTRI 2019E; CTRI 2019F; CTRI 2020A; CTRI 2020B; CTRI 2020C; CTRI 2020D; CTRI 2020E; Development 2019; Hospital 2020A; Hospital 2020B; IRCT 2020D; IRCT 2020F; IRCT 2020H; IRCT 2020K; Isrctn 2016B; Isrctn 2016C; Isrctn 2016D; Isrctn 2017B; Isrctn 2017C; Isrctn 2017D; ISRCTN 2017E; Isrctn 2018B; Isrctn 2019A; ISRCTN 2019B; Isrctn 2019C; Isrctn 2019D; ISRCTN 2020B; Joanne Kurtzberg 2020; JPRN-jRCT 2020A; Kaluzhny 2020; KCT 2018; Keim 2020; Kerns 2016A; Kitzerow 2020; Kong 2020A; Le Couteur 2012; Levine 2016; Mengoni 2017; Minnesota 2014B; Nct 2010T; Nct 2012J; Nct 2013O; Nct 2013P; Nct 2015^; Nct 2015_; Nct 2015`; Nct 2015a; Nct 2015b; Nct 2015c; Nct 2015d; Nct 2016[; Nct 2016\; Nct 2016]; Nct 2016^; Nct 2016_; Nct 2016`; Nct 2017V; Nct 2017W; Nct 2017X; Nct 2017Y; Nct 2017Z; Nct 2017[; Nct 2017\; Nct 2017]; Nct 2017^; Nct 2017_; Nct 2017`; Nct 2017a; Nct 2017b; Nct 2017c; Nct 2017d; Nct 2017e; Nct 2018K; Nct 2018L; Nct 2018M; Nct 2018N; Nct 2018O; Nct 2018P; Nct 2018Q; Nct 2018R; Nct 2018S; Nct 2018T; Nct 2018U; Nct 2018V; Nct 2018W; Nct 2018X; Nct 2018]; Nct 2018_; Nct 2018`; Nct 2018a; Nct 2018c; Nct 2018d; Nct 2018a; Nct 2019D; Nct 2019F; Nct 2019J; Nct 2019O; Nct 2019P; Nct 2019Q; Nct 2019R; Nct 2019S; Nct 2019T; Nct 2020A; Nct 2020B; Nct 2020C; Nct 2020D; Nct 2020E; Nct 2020F; Nct 2020H; Nct 2020I; Nct 2020J; Nct01187940 2010; Nct03553875 2018; Nct03778827 2018; Nct03899831 2019; Nct03924973 2019; Nct03963479 2019; Nct03984487 2019; Nct03984513 2019; Nct03994757 2018; Nct04005547 2018; Nct04024111 2019; Nct04042337 2019; Nct04060017 2019; Nct04060030 2019; Nct04078061 2019; Nct04089579 2019; Nct04114942 2019; Nct04119492 2018; Nct04146428 2019; Nct04166591 2019; Nct04174365 2019; Nct04182633 2019; Nct04218331 2019; Nct04220086 2019; Nct04237870 2020; Nct04242355 2020; Nct04244721 2020; Nct04258254 2020; Nct04270708 2020; Nct04276571 2020; Nct04278898 2020; Nct04283045 2020; Nct04293783 2020; Nct04299464 2020; Nct04312932 2020; Nct04319640 2020; Nct04327648 2020; Nct04442061 2020; Nct04446442 2019; Nct04452045 2020; Nct04460677 2020; Nct04501588 2020; Nct04509401 2020; Nct04533607 2020; Nct04551313 2020; Nct04557488 2020; Nct04562688 2020; Nct04574206 2020; Papadopoulos 2019B; Rahman 2018; Randell 2019A; Rbr-6c36pg 2018; Rbr-75bsvk 2020; Rbr-7nq8m7 2011; Rbr-8bfjgk 2018; Rbr-8cq 2015; Rbr-8ttw3f 2018; Rbr-93wgtg 2020; Rbr-9737g8 2020; Silverman 2014; Smith 2019; Sukhodolsky 2019; Tanaka 2010; Tctr20191215004 2019; Tctr20200522003 2020; University 2020A; Varley 2019; Winter 2019 |
| Withdrawn | 20 | Floreo 2018; Health 2006B; Health 2010; Health 2013; Hospital 2009; Hospital 2011; Hospital 2014C; Hospital 2015B; Isrctn 2011D; Jprn 2016B; Jprn-Umin 2013; Jprn-Umin 2016G; Minnesota 2014A; Nct 2012N; Nct 2018[; Nct 2019E; Nct04016701 2019; Nct04233502 2020; Research 2020; Sun 2018 |

Note: we have listed study names here. Full references for these studies can be found in Appendix 8.

### Table 4 Risk of bias (ordered by comparisons)

| Study name | Intervention 1 | Intervention 2 | Intervention 1: number of participants | Intervention 2: number of participants | Bias arising from the randomisation process | Risk of bias due to deviations from the intended interventions | Risk of bias due to missing outcome data | Risk of bias in measurement of the outcome | Risk of bias in selection of the reported result |
| --- | --- | --- | --- | --- | --- | --- | --- | --- | --- |
|
| Chalfant 2007[90] | CBTAdaptedGroup | NoAdditionalIntervention | 28 | 19 | Some concerns | Some concerns | Low risk | High risk | Some concerns |
| Kilburn 2020[120] | CBTAdaptedGroup | NoAdditionalIntervention | 19 | 19 | Low risk | High risk | High risk | High risk | Some concerns |
| Langdon 2016[129] | CBTAdaptedGroup | NoAdditionalIntervention | 23 | 25 | Low risk | High risk | High risk | Low risk | High risk |
| Mackay 2017[141] | CBTAdaptedGroup | NoAdditionalIntervention | 16 | 12 | Low risk | Low risk | High risk | High risk | Some concerns |
| Santomauro 2016[140] | CBTAdaptedGroup | NoAdditionalIntervention | 8 | 5 | Some concerns | Some concerns | High risk | High risk | Some concerns |
| Weiss 2018[115] | CBTAdaptedGroup | NoAdditionalIntervention | 35 | 33 | Low risk | Low risk | Low risk | High risk | Low risk |
| Fujii 2013[89] | CBTAdaptedFamilyBased | NoAdditionalIntervention | 7 | 5 | Some concerns | Some concerns | High risk | High risk | Some concerns |
| Luxford 2017[112] | CBTAdaptedFamilyBased | NoAdditionalIntervention | 18 | 17 | Some concerns | Low risk | Low risk | High risk | Some concerns |
| Mcconachie 2014[103] | CBTAdaptedFamilyBased | NoAdditionalIntervention | 17 | 14 | Low risk | Low risk | High risk | High risk | Some concerns |
| Storch 2013a[124] | CBTAdaptedFamilyBased | NoAdditionalIntervention | 24 | 21 | High risk | Low risk | Low risk | Low risk | Low risk |
| Wood 2009[93] | CBTAdaptedFamilyBased | NoAdditionalIntervention | 14 | 22 | Some concerns | Some concerns | Low risk | Some concerns | Low risk |
| Wood 2015[108] | CBTAdaptedFamilyBased | NoAdditionalIntervention | 19 | 14 | Low risk | Low risk | Low risk | Some concerns | Some concerns |
| Andrews 2013[188] | CBTAdaptedFamilyBased | CBTAdaptedGroup | 29 | 30 | Low risk | Low risk | Low risk | High risk | Some concerns |
| Maskey 2019b[117] | CBTAdaptedIndividual | NoAdditionalIntervention | 14 | 13 | Low risk | High risk | High risk | High risk | Some concerns |
| Mcnally Keehn 2013[35] | CBTAdaptedIndividual | NoAdditionalIntervention | 12 | 10 | Some concerns | Low risk | Low risk | High risk | Some concerns |
| Storch 2015[107] | CBTAdaptedIndividual | NoAdditionalIntervention | 16 | 15 | Some concerns | Low risk | Low risk | Low risk | Low risk |
| Wood 2020[84] | CBTAdaptedIndividual | NoAdditionalIntervention | 66 | 18 | Low risk | High risk | High risk | High risk | Some concerns |
| Nct 2013e[145] | MedicationADH | NoAdditionalIntervention | 17 | 13 | Low risk | Low risk | Low risk | High risk | Some concerns |
| Parker 2019[127] | MedicationADH | NoAdditionalIntervention | 17 | 13 | Low risk | Low risk | Low risk | High risk | Some concerns |
| Squassante 2018[85] | MedicationADH | NoAdditionalIntervention | 141 | 72 | Low risk | High risk | High risk | High risk | Some concerns |
| Umbricht 2017[131] | MedicationADH | NoAdditionalIntervention | 9 | 9 | Low risk | High risk | Low risk | Low risk | High risk |
| Corbett 2017[111] | SkillsTraining | NoAdditionalIntervention | 17 | 13 | Some concerns | Some concerns | High risk | High risk | Some concerns |
| Kuehnel 2014[102] | SkillsTraining | NoAdditionalIntervention | 19 | 23 | Some concerns | Low risk | High risk | High risk | Some concerns |
| Mcvey 2016a[130] | SkillsTraining | NoAdditionalIntervention | 24 | 23 | Some concerns | High risk | High risk | High risk | Some concerns |
| Morgan 2014[144] | SkillsTraining | NoAdditionalIntervention | 12 | 12 | Some concerns | High risk | High risk | High risk | Some concerns |
| Drahota 2009[122] | CBTNonAdaptedFamilyBased | NoAdditionalIntervention | 17 | 23 | Low risk | Some concerns | Low risk | High risk | Some concerns |
| Nct 2013b[97] | CBTNonAdaptedFamilyBased | NoAdditionalIntervention | 14 | 18 | Some concerns | Some concerns | Low risk | Low risk | Some concerns |
| Reaven 2012[147] | CBTNonAdaptedFamilyBased | NoAdditionalIntervention | 20 | 23 | Low risk | Low risk | High risk | Low risk | Some concerns |
| Conaughton 2017[110] | CBTNonAdaptedSelfDirected | NoAdditionalIntervention | 18 | 18 | Low risk | Some concerns | High risk | Low risk | Some concerns |
| Gaigg 2020[137] | CBTNonAdaptedSelfDirected | NoAdditionalIntervention | 9 | 16 | Some concerns | High risk | High risk | High risk | Some concerns |
| Bernaerts 2020[135] | MedicationOxytocin | NoAdditionalIntervention | 22 | 18 | Low risk | Low risk | Low risk | Some concerns | Some concerns |
| Nct 2012b[96] | MedicationOxytocin | NoAdditionalIntervention | 16 | 18 | Low risk | Low risk | Low risk | Low risk | Some concerns |
| Yamasue 2020[87] | MedicationOxytocin | NoAdditionalIntervention | 51 | 52 | Low risk | High risk | Low risk | High risk | Some concerns |
| Eslamzadeh 2018[113] | MedicationSNRI | NoAdditionalIntervention | 22 | 22 | Low risk | Low risk | Low risk | Low risk | Some concerns |
| Hospital 2007[91] | MedicationSNRI | NoAdditionalIntervention | 27 | 30 | Some concerns | Low risk | High risk | High risk | Some concerns |
| Politte 2018[37] | MedicationSNRI | NoAdditionalIntervention | 30 | 32 | Low risk | Low risk | High risk | Low risk | Some concerns |
| Gaigg 2020[137] | Mindfulness | NoAdditionalIntervention | 14 | 16 | Some concerns | High risk | High risk | High risk | Some concerns |
| Pagni 2020[138] | Mindfulness | NoAdditionalIntervention | 15 | 13 | Some concerns | High risk | High risk | High risk | Some concerns |
| Spek 2013[38] | Mindfulness | NoAdditionalIntervention | 20 | 21 | Low risk | Some concerns | Low risk | High risk | Some concerns |
| Gaigg 2020[137] | Mindfulness | CBTNonAdaptedSelfDirected | 14 | 9 | Some concerns | High risk | High risk | High risk | Some concerns |
| Oswald 2018[133] | SkillsTrainingGroup | NoAdditionalIntervention | 25 | 15 | Some concerns | High risk | High risk | High risk | High risk |
| Reitzel 2013[98] | SkillsTrainingGroup | NoAdditionalIntervention | 8 | 7 | High risk | High risk | High risk | High risk | Some concerns |
| Wright 2016[109] | SkillsTrainingGroup | NoAdditionalIntervention | 23 | 27 | High risk | Low risk | Low risk | High risk | Low risk |
| Solomon 2008[139] | ABA | NoAdditionalIntervention | 10 | 9 | Some concerns | High risk | High risk | High risk | Some concerns |
| Cortesi 2012[83] | CBTNonAdaptedIndividual | NoAdditionalIntervention | 33 | 32 | Some concerns | High risk | High risk | High risk | Some concerns |
| Wood 2020[84] | CBTNonAdaptedIndividual | NoAdditionalIntervention | 59 | 18 | Low risk | High risk | High risk | High risk | Some concerns |
| Wood 2020[84] | CBTNonAdaptedIndividual | CBTAdaptedIndividual | 59 | 66 | Low risk | High risk | High risk | High risk | Some concerns |
| Liu 2019[126] | DietarySupplement | NoAdditionalIntervention | 36 | 35 | Low risk | High risk | High risk | Low risk | Some concerns |
| Mehrazad-Saber 2018[125] | DietarySupplement | NoAdditionalIntervention | 21 | 22 | Some concerns | High risk | High risk | High risk | Some concerns |
| Hesselmark 2014[143] | GroupActivity | CBTAdaptedGroup | 28 | 27 | Low risk | Some concerns | High risk | High risk | Some concerns |
| Sung 2011[95] | GroupActivity | CBTAdaptedGroup | 29 | 28 | Some concerns | High risk | High risk | High risk | Some concerns |
| Potter 2019[118] | MedicationSSRI | NoAdditionalIntervention | 26 | 21 | Low risk | High risk | High risk | High risk | Some concerns |
| Reddihough 2019[86] | MedicationSSRI | NoAdditionalIntervention | 53 | 53 | Low risk | Low risk | High risk | High risk | Some concerns |
| Schohl 2014[104] | SkillsTrainingGroupPEERS | NoAdditionalIntervention | 29 | 29 | Some concerns | High risk | High risk | High risk | High risk |
| Yoo 2014[105] | SkillsTrainingGroupPEERS | NoAdditionalIntervention | 23 | 24 | High risk | High risk | High risk | Some concerns | Some concerns |
| White 2013[100] | SkillsTrainingMASSI | NoAdditionalIntervention | 15 | 15 | Low risk | Low risk | Low risk | High risk | Some concerns |
| Du 2015[106] | ABA+MedicationDiuretic | ABA | 29 | 26 | High risk | Low risk | High risk | Some concerns | Some concerns |
| Tachibana 2013[99] | BookReading | NoAdditionalIntervention | 6 | 4 | Low risk | High risk | High risk | Low risk | Some concerns |
| Cook 2019[116] | CBTAdaptedParentMediated | NoAdditionalIntervention | 12 | 14 | Some concerns | High risk | High risk | High risk | Some concerns |
| Russell 2019[134] | CBTAdaptedSelfDirected | NoAdditionalIntervention | 28 | 20 | Low risk | High risk | High risk | Some concerns | Some concerns |
| Storch 2020[121] | CBTFamilyBasedExposureFocussed | NoAdditionalIntervention | 11 | 18 | Some concerns | High risk | Low risk | High risk | Some concerns |
| Scarpa 2011[94] | CBTNonAdaptedGroup | NoAdditionalIntervention | 5 | 6 | Some concerns | Low risk | Low risk | High risk | Some concerns |
| Cortesi 2012[83] | CBTNonAdaptedIndividual+MedicationMelatonin | NoAdditionalIntervention | 35 | 32 | Some concerns | High risk | High risk | High risk | Some concerns |
| Cortesi 2012[83] | CBTNonAdaptedIndividual+MedicationMelatonin | CBTNonAdaptedIndividual | 35 | 33 | Some concerns | High risk | High risk | High risk | Some concerns |
| Murphy 2017[36] | Counselling | SkillsTrainingMASSI | 19 | 17 | Low risk | Some concerns | Low risk | Low risk | Some concerns |
| Isong 2014[101] | Distraction | NoAdditionalIntervention | 15 | 19 | Low risk | Some concerns | High risk | High risk | Some concerns |
| Balci 2020[142] | IndividualCBT | NoAdditionalIntervention | 11 | 13 | Some concerns | High risk | Low risk | High risk | Some concerns |
| Mcdougle 1998[128] | MedicationAtypicalAntiPsychotic | NoAdditionalIntervention | 14 | 16 | Low risk | Low risk | High risk | High risk | Some concerns |
| Danforth 2018[132] | MedicationMDMA | NoAdditionalIntervention | 8 | 4 | Some concerns | Low risk | Low risk | Some concerns | Some concerns |
| Cortesi 2012[83] | MedicationMelatonin | NoAdditionalIntervention | 34 | 32 | Some concerns | High risk | High risk | High risk | Some concerns |
| Cortesi 2012[83] | MedicationMelatonin | CBTNonAdaptedIndividual | 34 | 33 | Some concerns | High risk | High risk | High risk | Some concerns |
| Cortesi 2012[83] | MedicationMelatonin | CBTNonAdaptedIndividual+MedicationMelatonin | 34 | 35 | Some concerns | High risk | High risk | High risk | Some concerns |
| Dean 2017[33] | MedicationNAC | NoAdditionalIntervention | 48 | 50 | Some concerns | Low risk | Low risk | Low risk | Some concerns |
| Nct 2011c[123] | MedicationNaSSA | NoAdditionalIntervention | 20 | 10 | Low risk | Low risk | Low risk | Low risk | Some concerns |
| Campbell 1993[146] | OpioidReceptorAntagonist | NoAdditionalIntervention | 23 | 18 | Some concerns | High risk | High risk | Some concerns | Some concerns |
| Bischof 2018[88] | ParentPsychoeducation | NoAdditionalIntervention | 11 | 13 | Some concerns | Low risk | High risk | High risk | Some concerns |
| Piravej 2009[92] | SensoryIntegrationTherapy+ThaiTraditionalMassage | SensoryIntegrationTherapy | 30 | 30 | Low risk | Low risk | Low risk | High risk | Some concerns |
| Ioannou 2020[119] | SkillsTrainingGroupSENSE | NoAdditionalIntervention | 44 | 33 | Low risk | Low risk | High risk | High risk | Some concerns |
| Capriola-Hall 2020[136] | SkillsTrainingIndividual | NoAdditionalIntervention | 16 | 16 | Some concerns | Low risk | Low risk | High risk | Some concerns |
| Pryor 2018[114] | SkillsTrainingSelfdirected | CBTNonAdaptedSelfDirected | 12 | 12 | Some concerns | High risk | High risk | Low risk | Some concerns |
| Isong 2014[101] | SkillsTrainingVideo | NoAdditionalIntervention | 17 | 19 | Low risk | Some concerns | High risk | High risk | Some concerns |
| Isong 2014[101] | SkillsTrainingVideo | Distraction | 17 | 15 | Low risk | Some concerns | High risk | High risk | Some concerns |
| Isong 2014[101] | SkillsTrainingVideo+Distraction | NoAdditionalIntervention | 18 | 19 | Low risk | Some concerns | High risk | High risk | Some concerns |
| Isong 2014[101] | SkillsTrainingVideo+Distraction | Distraction | 18 | 15 | Low risk | Some concerns | High risk | High risk | Some concerns |
| Isong 2014[101] | SkillsTrainingVideo+Distraction | SkillsTrainingVideo | 18 | 17 | Low risk | Some concerns | High risk | High risk | Some concerns |

### Table 5 Summary of outcomes, included studies and included interventions

| Outcome | Number of studies | Total number of participants | References | Interventions included | Number of trials included in network meta-analysis (NMA) |
| --- | --- | --- | --- | --- | --- |
| Children: Proportion of patients with anxiety | 4 | 78 | Fujii 2013; Mcnally Keehn 2013; Murphy 2017; Bischof 2018 | counselling, family-based adapted CBT, individual adapted CBT, MASSI skills training, parent psychoeducation, and no additional intervention | NMA was not performed for this outcome measure |
| Children: Anxiety scores | 44 | 1966 | Chalfant 2007; Hospital 2007; Piravej 2009; Wood 2009; Scarpa 2011; Sung 2011; Nct 2012; Reaven 2012; Andrews 2013; Fujii 2013; Mcnally Keehn 2013; Nct 2013; Nct 2013; Reitzel 2013; Storch 2013; Tachibana 2013; White 2013; Isong 2014; Kuehnel 2014; Mcconachie 2014; Schohl 2014; Yoo 2014; Du 2015; Storch 2015; Wood 2015; Wright 2016; Conaughton 2017; Corbett 2017; Dean 2017; Luxford 2017; Murphy 2017; Bischof 2018; Eslamzadeh 2018; Politte 2018; Pryor 2018; Weiss 2018; Cook 2019; Maskey 2019; Potter 2019; Reddihough 2019; Wood 2019; Ioannou 2020; Kilburn 2020; Storch 2020 | ABA, ABA plus diuretic, anti-diuretic hormone analogue, book reading, counselling, distraction, family-based adapted CBT, family-based exposure-focussed CBT, family-based non-adapted CBT, group activity, group adapted CBT, group non-adapted CBT, group skills training, individual adapted CBT, individual non-adapted CBT, MASSI skills training, N-acetyl cysteine, oxytocin, parent psychoeducation, parent-mediated adapted CBT, PEERS group skills training, self-directed non-adapted CBT, self-directed skills training, SENSE group skills training, sensory integration therapy, sensory integration therapy plus Thai traditional massage, skills training, SNRI, SSRI, video skills training, video skills training plus distraction, and no additional intervention | 42 |
| Children: Anxiety change scores | 12 | 804 | Chalfant 2007; Drahota 2009; Nct 2011; Cortesi 2012; Storch 2013; Mehrazad-Saber 2018; Weiss 2018; Liu 2019; Parker 2019; Potter 2019; Reddihough 2019; Wood 2019 | anti-diuretic hormone analogue, dietary supplement, family-based adapted CBT, family-based non-adapted CBT, group adapted CBT, individual adapted CBT, individual non-adapted CBT, individual non-adapted CBT plus melatonin, melatonin, NaSSA, SSRI, and no additional intervention | 12 |
| Adults: Anxiety scores | 13 | 526 | Mcdougle 1998; Spek 2013; Langdon 2016; Mcvey 2016; Umbricht 2017; Danforth 2018; Oswald 2018; Russell 2019; Bernaerts 2020; Capriola-Hall 2020; Gaigg 2020; Pagni 2020; Yamasue 2020 | anti-diuretic hormone analogue, atypical antipsychotic, group adapted CBT, group skills training, individual skills training, MDMA, mindfulness, oxytocin, self-directed adapted CBT, self-directed non-adapted CBT, skills training, and no additional intervention | 13 |
| Adults: Anxiety change scores | 2 | 121 | Umbricht 2017; Yamasue 2020 | anti-diuretic hormone analogue, oxytocin, and no additional intervention | 2 |
| Children: Depression scores | 7 | 231 | Solomon 2008; Kuehnel 2014; Schohl 2014; Yoo 2014; Santomauro 2016; Mackay 2017; Balci 2020 | ABA, group adapted CBT, individual CBT, PEERS group skills training, skills training, and no additional intervention | 7 |
| Adults: Depression scores | 10 | 448 | Mcdougle 1998; Spek 2013; Hesselmark 2014; Morgan 2014; Langdon 2016; Russell 2019; Capriola-Hall 2020; Gaigg 2020; Pagni 2020; Yamasue 2020 | atypical antipsychotic, group activity, group adapted CBT, individual skills training, mindfulness, oxytocin, self-directed adapted CBT, self-directed non-adapted CBT, skills training, and no additional intervention | 10 |
| Adults: Depression change scores | 1 | 40 | Bernaerts 2020 | oxytocin, no additional intervention | NMA was not performed for this outcome measure |
| Children: Quality of life | 2 | 87 | NCT 2013e; Hospital 2007 | anti-diuretic hormone analogue, SNRI, and no additional intervention | 2 |
| Adults: Quality of life | 1 | 48 | Russell 2019 | self-directed adapted CBT, no additional intervention | NMA was not performed for this outcome measure |
| Adults: Change in Quality of life | 2 | 95 | Hesselmark 2014; Bernaerts 2020 | group activity, group adapted CBT, oxytocin, and no additional intervention | NMA was not performed for this outcome measure |
| Serious Adverse events | 9 | 328 | Nct 2011c; Nct 2013b; Nct 2013e; Storch 2013a; Storch 2015; Dean 2017; Danforth 2018; Potter 2019; Russell 2019 | Nct 2011c; Nct 2013b; Nct 2013e; Storch 2013a; Storch 2015; Danforth 2018; Russell 2019 | NMA was not performed for this outcome measure |
| Adults: mental health related quality of life | 1 | 48 | Russell 2019 | self-directed adapted CBT, no additional intervention | NMA was not performed for this outcome measure |
| Self-harm | 1 | 41 | Campbell 1993 | opioid receptor antagonist and no additional intervention | NMA was not performed for this outcome measure |
| Non-serious adverse events - number of people | 7 | 183 | Nct 2011c; Nct 2013b; Nct 2013e; Storch 2013a; Storch 2015; Danforth 2018; Russell 2019 | anti-diuretic hormone analogue, family-based adapted CBT, family-based non-adapted CBT, individual adapted CBT, MDMA, NaSSA, self-directed adapted CBT, and no additional intervention | 4 |
| Non-serious adverse events - number of events | 1 | 12 | Danforth 2018 | MDMA, no additional intervention | NMA was not performed for this outcome measure |
| Proportion of people with adverse events | 9 | 337 | Campbell 1993; Nct 2012b; Nct 2013b; Nct 2013e; Storch 2013a; Storch 2015; Danforth 2018; Reddihough 2019; Russell 2019 | anti-diuretic hormone analogue, family-based adapted CBT, family-based non-adapted CBT, individual adapted CBT, MDMA, opioid receptor antagonist, oxytocin, self-directed adapted CBT, SSRI, and no additional intervention | 5 |
| Number of adverse events | 8 | 568 | Campbell 1993; Dean 2017; Danforth 2018; Politte 2018; Parker 2019; Potter 2019; Reddihough 2019; Yamasue 2020 | anti-diuretic hormone analogue, MDMA, N-acetyl cysteine, opioid receptor antagonist, oxytocin, SNRI, SSRI, and no additional intervention | 8 |
| Proportion of people who died | 1 | 20 | Nct 2013e | anti-diuretic hormone analogue | NMA was not performed for this outcome measure |

Note: we have listed study name here. Full references for these studies can be found in Appendix 9.

### Table 6 Fit statistics for fixed-effect, random-effects, and inconsistency model

| Anxiety (scores) (children) | Fixed-effect model | Random-effects model | Inconsistency model |
| --- | --- | --- | --- |
| Dbar | 104.8 | 35.76 | 35.57 |
| DIC | 131.8 | 78.19 | 78.47 |
| pD | 27.02 | 42.42 | 42.9 |
| Anxiety (scores) (children): change | Fixed-effect model | Random-effects model | Inconsistency model |
| Dbar | 28.8 | 5.217 | - |
| DIC | 39.87 | 20.14 | - |
| pD | 11.07 | 14.93 | - |
| Anxiety (scores) (adults) | Fixed-effect model | Random-effects model | Inconsistency model |
| Dbar | 10.85 | 10.45 | - |
| DIC | 21.89 | 23.17 | - |
| pD | 11.04 | 12.72 | - |
| Anxiety (scores) (adults): change | Fixed-effect model | Random-effects model | Inconsistency model |
| Dbar | 0.9702 | - | - |
| DIC | 2.994 | - | - |
| pD | 2.023 | - | - |
| Depression (scores) (children) | Fixed-effect model | Random-effects model | Inconsistency model |
| Dbar | 10.84 | 6.522 | - |
| DIC | 15.86 | 13.36 | - |
| pD | 5.022 | 6.833 | - |
| Depression (scores) (adults) | Fixed-effect model | Random-effects model | Inconsistency model |
| Dbar | 7.241 | 6.788 | - |
| DIC | 16.31 | 17.21 | - |
| pD | 9.069 | 10.42 | - |
| Depression (scores) (adults): change | Fixed-effect model | Random-effects model | Inconsistency model |
| Dbar | 0.5693 | - | - |
| DIC | 1.58 | - | - |
| pD | 1.01 | - | - |
| Quality of life (children) | Fixed-effect model | Random-effects model | Inconsistency model |
| Dbar | 1.145 | - | - |
| DIC | 3.169 | - | - |
| pD | 2.023 | - | - |
| Non-serious adverse events (number of people) | Fixed-effect model | Random-effects model | Inconsistency model |
| Dbar | 14.11 | - | - |
| DIC | 17.93 | - | - |
| pD | 3.817 | - | - |
| Any adverse events (number of people) | Fixed-effect model | Random-effects model | Inconsistency model |
| Dbar | 42.42 | - | - |
| DIC | 52.39 | - | - |
| pD | 9.963 | - | - |
| Any adverse events (number of events) | Fixed-effect model | Random-effects model | Inconsistency model |
| Dbar | 106.7 | 106.7 | - |
| DIC | 121.6 | 121.6 | - |
| pD | 14.92 | 14.92 | - |
| Anxiety (scores) (children) (Mental illhealth) | Fixed-effect model | Random-effects model | Inconsistency model |
| Dbar | 67.71 | 19.62 | - |
| DIC | 82.76 | 41.93 | - |
| pD | 15.05 | 22.31 | - |
| Anxiety (scores) (children): change (Mental illhealth) | Fixed-effect model | Random-effects model | Inconsistency model |
| Dbar | 3.201 | - | - |
| DIC | 9.224 | - | - |
| pD | 6.023 | - | - |
| Anxiety (scores) (adults) (Mental illhealth) | Fixed-effect model | Random-effects model | Inconsistency model |
| Dbar | 3.428 | - | - |
| DIC | 7.437 | - | - |
| pD | 4.009 | - | - |
| Depression (scores) (adults) (Mental illhealth) | Fixed-effect model | Random-effects model | Inconsistency model |
| Dbar | 1.396 | - | - |
| DIC | 4.38 | - | - |
| pD | 2.983 | - | - |

Dbar: posterior mean of deviance; DIC: deviance information criteria; pD: effective number of parameters or leverage.

Empty cells for random-effects model indicate that the presence of only a single trial for all comparisons of any two interventions; empty cells for inconsistency model indicates absence of direct and indirect effect estimates for all comparisons of any two interventions for the outcome.

### Table 7 Effect estimates

Please see <https://doi.org/10.5281/zenodo.4844257>.

### Table 8 Summary of findings (Certainty of evidence)

| Interventions | Relative effect (95% CrI) | Anticipated absolute effect* (95% CrI) | | | Certainty of evidence | Rank |
| --- | --- | --- | --- | --- | --- | --- |
|  |  | NoAdditionalIntervention | Various interventions | Difference |  |  |
| Proportion of patients with anxiety Total studies: 1 Total participants: 24 | | | | | | |
| ParentPsychoeducation | OR 0.15 (0.02 to 0.87) Network estimate | 769 per 1000 | 332 per 1000 (59 to 743) | 231 fewer per 1000 (231 fewer to 26 fewer) | Very low certainty evidence^1,2,3^ | 1 (1 to 1) |
| NoAdditionalIntervention | Reference | | | | | 2 (2 to 2) |
| Anxiety (scores) (children) Total studies: 42 Total participants: 1831 | | | | | | |
| NoAdditionalIntervention | Reference | | | | | - |
| CBTAdaptedGroup |  |  | SMD 1.44 lower (2.47 lower to 0.48 lower) Network estimate | Same as previous column | Very low certainty evidence^1,3,4^ | - |
| CBTAdaptedFamilyBased |  |  | SMD 1.09 lower (1.88 lower to 0.35 lower) Network estimate | Same as previous column | Very low certainty evidence^1,3,4^ | - |
| CBTAdaptedIndividual |  |  | SMD 0.75 lower (1.74 lower to 0.23 higher) Network estimate | Same as previous column | Very low certainty evidence^1,3,4,5^ | - |
| MedicationADH |  |  | SMD 1.04 higher (0.95 lower to 3.02 higher) Network estimate | Same as previous column | Very low certainty evidence^1,3,4,5^ | - |
| SkillsTraining |  |  | SMD 0.53 lower (1.92 lower to 0.85 higher) Network estimate | Same as previous column | Very low certainty evidence^1,3,4,5^ | - |
| CBTNonAdaptedFamilyBased |  |  | SMD 1.35 lower (2.77 lower to 0.02 higher) Network estimate | Same as previous column | Very low certainty evidence^1,3,4,5^ | - |
| CBTNonAdaptedSelfDirected |  |  | SMD 0.44 lower (2.38 lower to 1.47 higher) Network estimate | Same as previous column | Very low certainty evidence^1,3,4,5^ | - |
| MedicationOxytocin |  |  | SMD 1.06 lower (3.02 lower to 0.91 higher) Network estimate | Same as previous column | Very low certainty evidence^1,3,4,5^ | - |
| MedicationSNRI |  |  | SMD 0.05 higher (1.05 lower to 1.16 higher) Network estimate | Same as previous column | Very low certainty evidence^1,3,4,5^ | - |
| SkillsTrainingGroup |  |  | SMD 0.10 lower (1.55 lower to 1.31 higher) Network estimate | Same as previous column | Very low certainty evidence^1,3,4,5^ | - |
| CBTNonAdaptedIndividual |  |  | SMD 0.64 lower (2.39 lower to 1.13 higher) Network estimate | Same as previous column | Very low certainty evidence^1,3,4,5^ | - |
| GroupActivity |  |  | SMD 1.46 lower (3.70 lower to 0.63 higher) Network estimate | Same as previous column | Very low certainty evidence^1,3,4,5^ | - |
| MedicationSSRI |  |  | SMD 0.19 lower (1.52 lower to 1.14 higher) Network estimate | Same as previous column | Very low certainty evidence^1,3,4,5^ | - |
| SkillsTrainingGroupPEERS |  |  | SMD 0.23 higher (1.11 lower to 1.59 higher) Network estimate | Same as previous column | Very low certainty evidence^1,3,4,5^ | - |
| SkillsTrainingMASSI |  |  | SMD 0.32 lower (2.30 lower to 1.63 higher) Network estimate | Same as previous column | Very low certainty evidence^1,3,4,5^ | - |
| BookReading |  |  | SMD 0.84 lower (3.10 lower to 1.49 higher) Network estimate | Same as previous column | Very low certainty evidence^1,3,4,5^ | - |
| CBTAdaptedParentMediated |  |  | SMD 0.77 lower (2.76 lower to 1.20 higher) Network estimate | Same as previous column | Very low certainty evidence^1,3,4,5^ | - |
| CBTFamilyBasedExposureFocussed |  |  | SMD 2.24 lower (4.30 lower to 0.18 lower) Network estimate | Same as previous column | Very low certainty evidence^1,3,4^ | - |
| CBTNonAdaptedGroup |  |  | SMD 2.80 higher (0.19 higher to 5.43 higher) Network estimate | Same as previous column | Low certainty evidence^1,3,4^ | - |
| Counselling |  |  | SMD 0.67 lower (3.44 lower to 2.06 higher) Network estimate | Same as previous column | Very low certainty evidence^1,3,4,5^ | - |
| Distraction |  |  | SMD 0.34 lower (2.31 lower to 1.58 higher) Network estimate | Same as previous column | Very low certainty evidence^1,3,4,5^ | - |
| MedicationNAC |  |  | SMD 0.30 lower (2.16 lower to 1.58 higher) Network estimate | Same as previous column | Very low certainty evidence^1,3,4,5^ | - |
| ParentPsychoeducation |  |  | SMD 0.85 lower (2.89 lower to 1.19 higher) Network estimate | Same as previous column | Very low certainty evidence^1,3,4,5^ | - |
| SkillsTrainingGroupSENSE |  |  | SMD 0.60 lower (2.44 lower to 1.26 higher) Network estimate | Same as previous column | Very low certainty evidence^1,3,4,5^ | - |
| SkillsTrainingSelfdirected |  |  | SMD 0.16 lower (2.91 lower to 2.65 higher) Network estimate | Same as previous column | Very low certainty evidence^1,3,4,5^ | - |
| SkillsTrainingVideo |  |  | SMD 0.17 higher (1.72 lower to 2.09 higher) Network estimate | Same as previous column | Very low certainty evidence^1,3,4,5^ | - |
| SkillsTrainingVideo+Distraction |  |  | SMD 0.11 lower (2.06 lower to 1.80 higher) Network estimate | Same as previous column | Very low certainty evidence^1,3,4,5^ | - |
| Anxiety (scores) (children): change Total studies: 12 Total participants: 804 | | | | | | |
| NoAdditionalIntervention | Reference | | | | | - |
| CBTAdaptedGroup |  |  | SMD 1.80 lower (4.15 lower to 0.37 higher) Network estimate | Same as previous column | Very low certainty evidence^1,3,4,5^ | - |
| CBTAdaptedFamilyBased |  |  | SMD 0.77 lower (3.95 lower to 2.43 higher) Network estimate | Same as previous column | Very low certainty evidence^1,3,4,5^ | - |
| CBTAdaptedIndividual |  |  | SMD 0.95 lower (3.88 lower to 1.99 higher) Network estimate | Same as previous column | Very low certainty evidence^1,3,4,5^ | - |
| MedicationADH |  |  | SMD 1.04 lower (4.24 lower to 2.15 higher) Network estimate | Same as previous column | Very low certainty evidence^1,3,4,5^ | - |
| CBTNonAdaptedFamilyBased |  |  | SMD 1.77 lower (4.95 lower to 1.42 higher) Network estimate | Same as previous column | Very low certainty evidence^1,3,4,5^ | - |
| CBTNonAdaptedIndividual |  |  | SMD 0.34 lower (2.56 lower to 1.91 higher) Network estimate | Same as previous column | Very low certainty evidence^1,3,4,5^ | - |
| DietarySupplement |  |  | SMD 0.01 lower (2.24 lower to 2.21 higher) Network estimate | Same as previous column | Very low certainty evidence^1,3,4,5^ | - |
| MedicationSSRI |  |  | SMD 0.03 lower (2.24 lower to 2.19 higher) Network estimate | Same as previous column | Very low certainty evidence^1,3,4,5^ | - |
| CBTNonAdaptedIndividual+MedicationMelatonin |  |  | SMD 1.70 lower (4.66 lower to 1.28 higher) Network estimate | Same as previous column | Very low certainty evidence^1,3,4,5^ | - |
| MedicationMelatonin |  |  | SMD 0.33 lower (3.26 lower to 2.63 higher) Network estimate | Same as previous column | Very low certainty evidence^1,3,4,5^ | - |
| MedicationNaSSA |  |  | SMD 0.34 lower (3.51 lower to 2.86 higher) Network estimate | Same as previous column | Very low certainty evidence^1,3,4,5^ | - |
| Anxiety (scores) (adults) Total studies: 13 Total participants: 526 | | | | | | |
| NoAdditionalIntervention | Reference | | | | | - |
| CBTAdaptedGroup |  |  | SMD 0.37 higher (1.09 lower to 1.87 higher) Network estimate | Same as previous column | Very low certainty evidence^1,3,5^ | - |
| MedicationADH |  |  | SMD 0.55 higher (1.08 lower to 2.16 higher) Network estimate | Same as previous column | Very low certainty evidence^1,3,5^ | - |
| SkillsTraining |  |  | SMD 0.50 lower (1.98 lower to 0.97 higher) Network estimate | Same as previous column | Very low certainty evidence^1,3,5^ | - |
| CBTNonAdaptedSelfDirected |  |  | SMD 0.05 lower (1.60 lower to 1.42 higher) Network estimate | Same as previous column | Very low certainty evidence^1,3,5^ | - |
| MedicationOxytocin |  |  | SMD 0.16 higher (0.89 lower to 1.18 higher) Network estimate | Same as previous column | Very low certainty evidence^1,3,5^ | - |
| Mindfulness |  |  | SMD 0.41 lower (1.27 lower to 0.48 higher) Network estimate | Same as previous column | Very low certainty evidence^1,3,5^ | - |
| SkillsTrainingGroup |  |  | SMD 0.04 lower (1.55 lower to 1.47 higher) Network estimate | Same as previous column | Very low certainty evidence^1,3,5^ | - |
| CBTAdaptedSelfDirected |  |  | SMD 0.72 lower (2.21 lower to 0.75 higher) Network estimate | Same as previous column | Very low certainty evidence^1,3,5^ | - |
| MedicationAtypicalAntiPsychotic |  |  | SMD 0.61 lower (2.15 lower to 0.92 higher) Network estimate | Same as previous column | Very low certainty evidence^1,3,5^ | - |
| MedicationMDMA |  |  | SMD 0.80 lower (2.65 lower to 1.05 higher) Network estimate | Same as previous column | Very low certainty evidence^1,3,5^ | - |
| SkillsTrainingIndividual |  |  | SMD 0.63 lower (2.15 lower to 0.89 higher) Network estimate | Same as previous column | Very low certainty evidence^1,3,5^ | - |
| Anxiety (scores) (adults): change Total studies: 2 Total participants: 121 | | | | | | |
| NoAdditionalIntervention | Reference | | | | | - |
| MedicationADH |  |  | SMD 0.21 lower (1.12 lower to 0.73 higher) Network estimate | Same as previous column | Very low certainty evidence^1,2,3,5^ | - |
| MedicationOxytocin |  |  | SMD 0.12 higher (0.27 lower to 0.51 higher) Network estimate | Same as previous column | Very low certainty evidence^1,2,3,5^ | - |
| Depression (scores) (children) Total studies: 7 Total participants: 231 | | | | | | |
| NoAdditionalIntervention | Reference | | | | | - |
| CBTAdaptedGroup |  |  | SMD 0.31 higher (2.27 lower to 3.00 higher) Network estimate | Same as previous column | Very low certainty evidence^1,2,3,5^ | - |
| SkillsTraining |  |  | SMD 0.45 lower (4.06 lower to 3.17 higher) Network estimate | Same as previous column | Very low certainty evidence^1,2,3,5^ | - |
| ABA |  |  | SMD 1.01 lower (4.71 lower to 2.70 higher) Network estimate | Same as previous column | Very low certainty evidence^1,2,3,5^ | - |
| SkillsTrainingGroupPEERS |  |  | SMD 0.37 lower (2.95 lower to 2.23 higher) Network estimate | Same as previous column | Very low certainty evidence^1,2,3,5^ | - |
| IndividualCBT |  |  | SMD 0.32 lower (4.01 lower to 3.40 higher) Network estimate | Same as previous column | Very low certainty evidence^1,2,3,5^ | - |
| Depression (scores) (adults) Total studies: 10 Total participants: 448 | | | | | | |
| NoAdditionalIntervention | Reference | | | | | - |
| CBTAdaptedGroup |  |  | SMD 0.04 higher (2.70 lower to 2.83 higher) Network estimate | Same as previous column | Very low certainty evidence^1,3,5^ | - |
| SkillsTraining |  |  | SMD 0.48 lower (3.28 lower to 2.30 higher) Network estimate | Same as previous column | Very low certainty evidence^1,3,5^ | - |
| CBTNonAdaptedSelfDirected |  |  | SMD 0.50 lower (3.13 lower to 2.09 higher) Network estimate | Same as previous column | Very low certainty evidence^1,3,5^ | - |
| MedicationOxytocin |  |  | SMD 0.03 higher (2.72 lower to 2.80 higher) Network estimate | Same as previous column | Very low certainty evidence^1,3,5^ | - |
| Mindfulness |  |  | SMD 0.52 lower (2.12 lower to 1.11 higher) Network estimate | Same as previous column | Very low certainty evidence^1,3,5^ | - |
| GroupActivity |  |  | SMD 0.47 higher (3.42 lower to 4.41 higher) Network estimate | Same as previous column | Very low certainty evidence^1,3,5^ | - |
| CBTAdaptedSelfDirected |  |  | SMD 0.72 lower (3.48 lower to 2.04 higher) Network estimate | Same as previous column | Very low certainty evidence^1,3,5^ | - |
| MedicationAtypicalAntiPsychotic |  |  | SMD 0.53 lower (3.31 lower to 2.24 higher) Network estimate | Same as previous column | Very low certainty evidence^1,3,5^ | - |
| SkillsTrainingIndividual |  |  | SMD 0.76 lower (3.55 lower to 2.02 higher) Network estimate | Same as previous column | Very low certainty evidence^1,3,5^ | - |
| Depression (scores) (adults): change Total studies: 1 Total participants: 40 | | | | | | |
| NoAdditionalIntervention | Reference | | | | |  |
| MedicationOxytocin |  |  | SMD 0.25 lower (0.38 lower to 0.87 higher) Direct estimate | Same as previous column | Very low certainty evidence^1,2,3,5^ |  |
| Quality of life (children) Total studies: 2 Total participants: 87 | | | | | | |
| NoAdditionalIntervention | Reference | | | | | - |
| MedicationADH |  |  | SMD 0.55 higher (0.20 lower to 1.30 higher) Network estimate | Same as previous column | Very low certainty evidence^1,2,3,5^ | - |
| MedicationSNRI |  |  | SMD 0.12 higher (0.41 lower to 0.65 higher) Network estimate | Same as previous column | Very low certainty evidence^1,2,3,5^ | - |
| Quality of life (adults) Total studies: 1 Total participants: 48 | | | | | | |
| NoAdditionalIntervention | Reference | | | | |  |
| CBTAdaptedSelfDirected |  |  | SMD 0.87 more (0.26 more to 1.48 more) Direct estimate | Same as previous column | Very low certainty evidence^1,2,3^ |  |
| Quality of life (adult): change Total studies: 1 Total participants: 40 | | | | | | |
| NoAdditionalIntervention | Reference | | | | |  |
| MedicationOxytocin |  |  | SMD 0.12 more (0.51 less to 0.74 more) Direct estimate | Same as previous column | Very low certainty evidence^1,2,3,5^ |  |
| Serious adverse events (number of people) Total studies: 1 Total participants: 98 | | | | | | |
| NoAdditionalIntervention | Reference | | | | | - |
| MedicationNAC | OR 1.05 (0.03 to 41.06) Direct estimate | 20 per 1000 | 21 per 1000 (1 to 456) | 1 more per 1000 (19 fewer to 436 more) | Very low certainty evidence^1,2,3,5^ | - |
| Mental health-related quality of life (maximal follow-up)  Total studies: 1  Total participants: 48 | | | | | | |
| NoAdditionalIntervention | Reference | | | | | - |
| "CBTAdaptedSelfDirected  (1 RCT; 43 participants)" | MD 4.34  (-2.14 to 10.74)  Network estimate | 28.9 | 33.24  (26.77 to 39.64) | 4.34 more  (2.14 fewer to 10.74 more) | Very low certainty evidence^1,2,3,5^ | - |
| Proportion of patients with self-harm Total studies: 1 Total participants: 41 | | | | | | |
| NoAdditionalIntervention | Reference | | | | | - |
| OpioidReceptorAntagonist | OR 0.48 (0.12 to 1.71) Direct estimate | 611 per 1000 | 428 per 1000 (163 to 729) | 183 fewer per 1000 (389 fewer to 118 more) | Very low certainty evidence^1,2,3,5^ | - |
| Non-serious adverse events (number of people) Total studies: 2 Total participants: 78 | | | | | | |
| NoAdditionalIntervention | Reference | | | | | - |
| MedicationADH | OR 0.21 (0.01 to 1.92) Network estimate | 394 per 1000 | 118 per 1000 (5 to 555) | 276 fewer per 1000 (389 fewer to 161 more) | Very low certainty evidence^1,2,3,5^ | - |
| CBTAdaptedSelfDirected | OR 2.88 (0.30 to 96.74) Network estimate | 394 per 1000 | 652 per 1000 (161 to 984) | 258 more per 1000 (233 fewer to 590 more) | Very low certainty evidence^1,2,3,5^ | - |
| Non-serious adverse events (number of events) Total studies: 1 Total participants: 12 | | | | | | |
| NoAdditionalIntervention | Reference | | | | | 1 (1 to 1) |
| MedicationMDMA | Rate ratio 2.30 (1.21 to 4.86) Network estimate | 2500 per 1000 | 5749 per 1000 (3013 to 12162) | 3249 more per 1000 (513 more to 9662 more) | Very low certainty evidence^1,2,3^ | 2 (2 to 2) |
| Any adverse events (number of people) Total studies: 5 Total participants: 262 | | | | | | |
| NoAdditionalIntervention | Reference | | | | | - |
| MedicationADH | OR 0.20 (0.01 to 1.92) Network estimate | 500 per 1000 | 169 per 1000 (6 to 658) | 331 fewer per 1000 (494 fewer to 158 more) | Very low certainty evidence^1,2,3,5^ | - |
| MedicationOxytocin | OR 1.11 (0.25 to 5.07) Network estimate | 500 per 1000 | 526 per 1000 (201 to 835) | 26 more per 1000 (299 fewer to 335 more) | Very low certainty evidence^1,2,3,5^ | - |
| MedicationSSRI | OR 1.42 (0.66 to 3.11) Network estimate | 500 per 1000 | 587 per 1000 (397 to 757) | 87 more per 1000 (103 fewer to 257 more) | Very low certainty evidence^1,2,3,5^ | - |
| CBTAdaptedSelfDirected | OR 2.93 (0.29 to 93.88) Network estimate | 500 per 1000 | 746 per 1000 (224 to 989) | 246 more per 1000 (276 fewer to 489 more) | Very low certainty evidence^1,2,3,5^ | - |
| OpioidReceptorAntagonist | OR 2.11 (0.60 to 7.90) Network estimate | 500 per 1000 | 678 per 1000 (373 to 888) | 178 more per 1000 (127 fewer to 388 more) | Very low certainty evidence^1,2,3,5^ | - |
| Any adverse events (number of events) Total studies: 8 Total participants: 505 | | | | | | |
| NoAdditionalIntervention | Reference | | | | | - |
| MedicationADH | Rate ratio 0.90 (0.57 to 1.42) Network estimate | 1785 per 1000 | 1608 per 1000 (1021 to 2531) | 177 fewer per 1000 (764 fewer to 747 more) | Very low certainty evidence^1,3,4,5^ | - |
| MedicationOxytocin | Rate ratio 1.58 (0.99 to 2.51) Network estimate | 1785 per 1000 | 2812 per 1000 (1775 to 4482) | 1028 more per 1000 (10 fewer to 2697 more) | Very low certainty evidence^1,3,4,5^ | - |
| MedicationSNRI | Rate ratio 2.50 (1.95 to 3.22) Network estimate | 1785 per 1000 | 4460 per 1000 (3471 to 5738) | 2675 more per 1000 (1687 more to 3954 more) | Very low certainty evidence^1,4,5^ | - |
| MedicationSSRI | Rate ratio 0.94 (0.78 to 1.12) Network estimate | 1785 per 1000 | 1673 per 1000 (1400 to 2001) | 111 fewer per 1000 (385 fewer to 217 more) | Very low certainty evidence^1,3,4,5^ | - |
| MedicationMDMA | Rate ratio 2.29 (1.21 to 4.91) Network estimate | 1785 per 1000 | 4085 per 1000 (2154 to 8760) | 2301 more per 1000 (370 more to 6975 more) | Very low certainty evidence^1,4,5^ | - |
| MedicationNAC | Rate ratio 1.20 (0.71 to 2.02) Network estimate | 1785 per 1000 | 2142 per 1000 (1265 to 3613) | 357 more per 1000 (519 fewer to 1829 more) | Very low certainty evidence^1,3,4,5^ | - |
| OpioidReceptorAntagonist | Rate ratio 1.13 (0.60 to 2.19) Network estimate | 1785 per 1000 | 2023 per 1000 (1075 to 3905) | 238 more per 1000 (710 fewer to 2121 more) | Very low certainty evidence^1,3,4,5^ | - |

^1^Downgraded one level for risk of bias
^2^Downgraded one level for imprecision due to small sample size
^3^Downgraded one level for reporting bias
^4^Downgraded one level for heterogeneity
^5^Downgraded one level for imprecision due to poor overlap of confidence intervals

### Table 9 Anxiety scales used

| Short name | Full name | Minimum score | Maximum score | Do higher scores indicate more anxiety? | Source of information |
| --- | --- | --- | --- | --- | --- |
| SCAC-C | Spencer Children’s Anxiety Scale – Child version | 0 | 114 | Yes | [https://www.scaswebsite.com/index.php?p=1_7](https://eur01.safelinks.protection.outlook.com/?url=https%3A%2F%2Fwww.scaswebsite.com%2Findex.php%3Fp%3D1_7&data=04%7C01%7C%7Cceb041340517443910bb08d8ba37281a%7C1faf88fea9984c5b93c9210a11d9a5c2%7C0%7C0%7C637464093341552855%7CUnknown%7CTWFpbGZsb3d8eyJWIjoiMC4wLjAwMDAiLCJQIjoiV2luMzIiLCJBTiI6Ik1haWwiLCJXVCI6Mn0%3D%7C1000&sdata=9uniLBI9J0sWwbGVPZKAdaZPgBfDXm5PrcWXlfAM9Tk%3D&reserved=0) |
| SCAS-P | Spence Children's Anxiety Scale – Parent version (change scores separated from final scores) | 0 | 114 | Yes | <https://www.scaswebsite.com/index.php?p=1_14> |
| SCAS-P-change | Spence Children's Anxiety Scale – Parent version: change from baseline | -144 | 144 | Yes | <https://www.scaswebsite.com/index.php?p=1_14> |
| SAS | School Anxiety Scale – Teacher Report | 0 | 48 | Yes | <https://www.sciencedirect.com/science/article/pii/S0887618507000643> |
| ADIS-PIR | ADIS-P Anxiety Disorders Interview Schedule – Parent Interference Ratings (renamed) | 0 | 8 | Yes | <https://www.ncbi.nlm.nih.gov/pmc/articles/PMC5490389/pdf/pas_29_7_835.pdf> |
| ADIS-change | Anxiety Disorders Interview Schedule (ADIS) for DSM-IV – change scores (separated from PIR scores) | -8 | 8 | Yes | <https://www.ncbi.nlm.nih.gov/pmc/articles/PMC5490389/pdf/pas_29_7_835.pdf> |
| ADIS-diagnoses | ADIS-C/P Number of Anxiety Diagnoses | 0 | 4 | Yes | <https://www.ncbi.nlm.nih.gov/pmc/articles/PMC5490389/pdf/pas_29_7_835.pdf> |
| CSR | ADIS-C/P Clinician Severity Rating | 0 | 8 | Yes | <https://www.ncbi.nlm.nih.gov/pmc/articles/PMC5490389/pdf/pas_29_7_835.pdf> |
| CGI-Sev | Clinical Global Impression – Severity scale (CGI-S) - anxiety | 1  or 0 | 7  or 6 | Yes | <https://archive.org/details/ecdeuassessmentm1933guyw/page/218/mode/2up?q=cgi> |
| CGI-Imp | Clinical Global Impression – Improvement scale (CGI-I) – anxiety | 1  or 0 | 7  or 6 | Yes | <https://archive.org/details/ecdeuassessmentm1933guyw/page/218/mode/2up?q=cgi> |
| PARS | Pediatric Anxiety Rating Scale  (change/mean scores separated from final scores) | 0 | 25  or 30 | Yes | <https://www.sciencedirect.com/science/article/pii/S0890856709609552?via%3Dihub> |
| PARS-change | Pediatric Anxiety Rating Scale – change in score from baseline | -25 | 25 | Yes | <https://www.sciencedirect.com/science/article/pii/S0890856709609552?via%3Dihub> |
| PARS-mean | Pediatric Anxiety Rating Scale - mean score across items | 0 | 5 | Yes | <https://www.sciencedirect.com/science/article/pii/S0890856709609552?via%3Dihub> |
| MASC-P | Multi-dimensional Anxiety Scale for Children – parent reported | 0 | 117 | Yes | <https://www.ncbi.nlm.nih.gov/pmc/articles/PMC3858516/pdf/nihms502295.pdf> (39 items; score 0-3) |
| MASC-C | Multi-dimensional Anxiety Scale for Children – child reported | 0 | 117 | Yes | <https://www.ncbi.nlm.nih.gov/pmc/articles/PMC3858516/pdf/nihms502295.pdf> (39 items; score 0-3) |
| CBCL-anx_T | Child Behavior Checklist - anxious & depressed scale – T-scores (re-named; separated from mean item score) | 0 | 100 | Yes | <https://www.ncbi.nlm.nih.gov/pmc/articles/PMC4527577/pdf/nihms606150.pdf> (raw score: 13 items; score 0-2) |
| CBCL-anx_item | Child Behavior Checklist – anxious & depressed scale – mean item value (separated from anx T-scores) | 0 | 2 | Yes | <https://www.ncbi.nlm.nih.gov/pmc/articles/PMC4527577/pdf/nihms606150.pdf> |
| CBCL-AP | Child Behavior Checklist – anxiety problems scale (separated from anxious/depressed scale) | 0 | 12 | Yes | <https://www.ncbi.nlm.nih.gov/pmc/articles/PMC4527577/pdf/nihms606150.pdf> (6 items; score 0-2) |
| CBCL-AP_T | Child Behavior Checklist – anxiety problems scale (T-scores) (separated from anxious/depressed scale) | 0 | 100 | Yes | <https://www.ncbi.nlm.nih.gov/pmc/articles/PMC4527577/pdf/nihms606150.pdf> (raw score: 6 items; score 0-2) |
| CBCL-int | Child Behavior Checklist – internalizing scale | 0 | 66 | Yes | <https://www.ncbi.nlm.nih.gov/pmc/articles/PMC4527577/pdf/nihms606150.pdf> (33 items; score 0-2) |
| Liebowitz | Liebowitz Social Anxiety Scale | 0 | 144 | Yes | <https://www.cambridge.org/core/journals/psychological-medicine/article/psychometric-properties-of-the-liebowitz-social-anxiety-scale/6891D37D00A9BEC179E61C8BFF30F08A> |
| CASI | Child and Adolescent Symptom Inventory - anxiety subscale | 0 | 60 | Yes | <https://www.ncbi.nlm.nih.gov/pmc/articles/PMC4038127/> (20 items: scored 0-3) |
| STAI-S | State-Trait Anxiety Inventory – State anxiety subscale | 20 | 80 | Yes | <https://doi.org/10.1002/acr.20561> |
| STAI-T | State-Trait Anxiety Inventory – Trait anxiety subscale | 20 | 80 | Yes | <https://doi.org/10.1002/acr.20561> |
| STAI_x | State-Trait Anxiety Inventory – unclear subscale | 20 | 80 | Yes | <https://doi.org/10.1002/acr.20561> |
| CSHQ_sleep | Children's Sleep Habits Questionnaire (CSHQ) - sleep anxiety subscale (re-named) | 4 | 12 | Yes | <https://depts.washington.edu/dbpeds/Screening%20Tools/CHSQ%20article.pdf> (4 items: scored 1-3) |
| CSHQ_total | Children's Sleep Habits Questionnaire (CSHQ) – total score (sleep subscale separated from total) | 33 | 99 | Yes | <https://depts.washington.edu/dbpeds/Screening%20Tools/CHSQ%20article.pdf> (33 items: scored 1-3) |
| RCMAS | Revised Children’s Manifest Anxiety Scale  (separated from worry factor subscale and RCMAS2) | 0 | 37 | Yes | <http://www.clintools.com/victims/resources/assessment/affect/rcmas.html> (37 items: score 0-1) |
| RCMAS_worry | Revised Children’s Manifest Anxiety Scale – worry factor subscale | 0 | 11 | Yes | <http://www.clintools.com/victims/resources/assessment/affect/rcmas.html> (11 items: score 0-1) |
| RCMAS2 | Revised Children’s Manifest Anxiety Scale – Second Edition (T-scores) | 0 | 100 | Yes | <https://link.springer.com/referenceworkentry/10.1007/978-0-387-79061-9_2435> (49 items; appears to be T-scores) |
| VAS | VAS scale - anxious or nervous (clinician rated) (row 141: cm changed to mm) | 0 | 100 | Yes | <https://www.ncbi.nlm.nih.gov/pmc/articles/PMC2904728/> |
| DBC-anx | Developmental Behavior Checklist – Parent/Carer Version - anxiety subsection (raw score) (separated from percentile rank) | 0 | 18 | Yes | <https://www.corc.uk.net/outcome-experience-measures/developmental-behaviour-checklist/> (9 items: score 0-2) |
| DBC-rank | Developmental Behavior Checklist – Parent/Carer Version - anxiety subsection (percentile rank) | 0 | 100 | Yes | <https://www.corc.uk.net/outcome-experience-measures/developmental-behaviour-checklist/> |
| DBC-item | Developmental Behavior Checklist – anxiety item | 0 | 2 | Yes | <https://www.corc.uk.net/outcome-experience-measures/developmental-behaviour-checklist/> (item 85) |
| SIAS | Social interaction anxiety scale | 0 | 80 | Yes | <https://novopsych.com.au/assessments/social-interaction-anxiety-scale-sias> (20 items; score 0-4) |
| Pre-school | Pre-school anxiety scale–revised | 0 | 112 | Yes | <https://www.scaswebsite.com/index.php?p=1_29> (28 items; score 0-4) |
| CARS | Childhood Autism Rating Scale - Anxiety subscale | 1 | 4 | Yes | <https://www.pearsonclinical.co.uk/Psychology/ChildMentalHealth/ChildAutisticSpectrumDisorders/ChildhoodAutismRatingScale-SecondEdition(CARS2)/ChildhoodAutismRatingScale-SecondEdition(CARS2).aspx> (1 item; score 1-4) |
| SPIN | Social Phobia Inventory | 0 | 68 | Yes | <https://psychology-tools.com/test/spin> |
| RCADS | Revised Child Anxiety and Depression Scale | 0 | 141 | Yes | <https://www.corc.uk.net/outcome-experience-measures/revised-childrens-anxiety-and-depression-scale-and-subscales/> (47 items; score 0-3) |
| CALIS-C | Child Anxiety Life Interference Scale – Child version (children’s opinion of interference in own life) | 0 | 36 | Yes | <https://www.mq.edu.au/__data/assets/pdf_file/0008/174266/CALISScoring_English_copyright_2016.pdf> |
| CALIS-P | Child Anxiety Life Interference Scale – Parent version (parent’s view of interference in the child’s life) (separated parent version from preschool version) | 0 | 36 | Yes | <https://www.mq.edu.au/__data/assets/pdf_file/0008/174266/CALISScoring_English_copyright_2016.pdf> |
| CALIS-PV | Child Anxiety Life Interference Scale – Preschool version (effects of anxiety symptoms on child's life) | 0 | 44 | Yes | <https://dev.mq.edu.au/__data/assets/pdf_file/0005/570137/CALIS_Preschool_2017_copyright_Scoring.pdf> (11 items; score 0-4) |
| CAIS | Child Anxiety Impact Scale (CAIS) – total  (separated social subscale data) | 0 | 81 | Yes | <https://www.ncbi.nlm.nih.gov/pmc/articles/PMC4137893/> (27 items; score 0-3) |
| CAIS_social | Child Anxiety Impact Scale (CAIS) – social subscale | 0 | 33 | Yes | <https://www.ncbi.nlm.nih.gov/pmc/articles/PMC4137893/> (11 items; score 0-3) |
| Conners' | Conners' parent rating scales - anxiety subsection | 0 | 3 | Yes | <https://journals.sagepub.com/doi/pdf/10.1177/153450849902401-409> (8 items; score 0-3; data appear to be mean score across items) |
| Beck | Beck Anxiety Inventory | 0 | 63 | Yes | <https://onlinelibrary.wiley.com/doi/10.1002/acr.20561#:~:text=Scoring%20is%20easily%20accomplished%20by,30%E2%80%9363%2C%20severe%20anxiety> (21 items; score 0-3) |
| Confidence | Confidence in managing anxiety | 1 | 10 | No | <https://www.cambridge.org/core/journals/behavioural-and-cognitive-psychotherapy/article/improving-emotion-regulation-with-cbt-in-young-children-with-high-functioning-autism-spectrum-disorders-a-pilot-study/33CA7970FE16CEDDC1B1F247505E7444> |
| PGI | Parent Global Impressions-2 scale - anxiety subscale (average change) | -6 | 6 | No | <https://www.ncbi.nlm.nih.gov/pmc/articles/PMC5872787/> |
| Hamilton | Hamilton Rating Scale for Anxiety | 0 | 56 | Yes | <https://dcf.psychiatry.ufl.edu/files/2011/05/HAMILTON-ANXIETY.pdf> |
| Venham | Venham anxiety rating scale | 0 | 5 | Yes | <https://jglobaloralhealth.org/appropriateness-of-various-behavior-rating-scales-used-in-pediatric-dentistry-a-review/#:~:text=Venham%20anxiety%20and%20behavioral%20rating,anxiety%20or%20lack%20of%20cooperation>. |
| SCL-90 | Symptom Checklist-90-Revised – Anxiety  (separated from change score) | 0 | 64 | Yes | <http://scireproject.com/wp-content/uploads/Clinician-Summary-v.5.0_SCL-90-R.pdf> (16 items; score 0-4) |
| SCL-90_change | Symptom Checklist-90-Revised – Anxiety – change in score | -64 | 64 | Yes | <http://scireproject.com/wp-content/uploads/Clinician-Summary-v.5.0_SCL-90-R.pdf> |
| SWQ-C | Social Worries Questionnaire – child reported | 0 | 26 | Yes | <https://www.scaswebsite.com/index.php?p=1_57> |
| SWQ-T | Social Worries Questionnaire – teacher-reported | 0 | 16 | Yes | <https://www.scaswebsite.com/index.php?p=1_57> |
| CMFWQ | Children’s moods, fears and worries questionnaire | 1 | 5 | Yes | <https://onlinelibrary.wiley.com/doi/full/10.1002/icd.1966> |
| FearQ | Fear questionnaire - anxiety/depression | 0 | 40 | Yes | <https://reader.elsevier.com/reader/sd/pii/000579677990041X?token=43B1756C9535DD8E3EB236CEDD4D4B08835E2BC1983507141B0D2B6CC465C7788EDC3DC60697C177409998361C421D21> |
| FSSC-R | Fear Survey Schedule for Children – Revised | 80 | 240 | Yes | <https://www.sciencedirect.com/science/article/pii/000579677990041X> |
| GAD7 | Generalised Anxiety Disorder-7 | 0 | 21 | Yes | <https://www.corc.uk.net/outcome-experience-measures/generalised-anxiety-disorder-assessment/#:~:text=GAD%2D7%20total%20score%20for,ranges%20from%200%20to%2021.&text=Scores%20of%205%2C%2010%2C%20and,score%20is%2010%20or%20greater> |
| SAAM | State Adult Attachment Measure (SAAM) - attachment anxiety subscale (change scores) | -42 | 42 | Yes | <https://www.psytoolkit.org/survey-library/attachment-saam.html> (raw score range: 7-49) |
| ASR | Adult Self Report (ASR) Anxiety Problems Subscale (T-scores) (same as Participant ASEBA anxiety problems scale) | 0 | 100 | Yes | <https://nda.nih.gov/data_structure.html?short_name=abcd_asrs01> (raw scores 0-12) |

### Table 10 Depression scales used

| Short name | Full name | Minimum score | Maximum score | Do higher scores indicate more depression? | Source of information |
| --- | --- | --- | --- | --- | --- |
| BDI | Beck Depression Inventory | 0 | 63 | Yes | <https://www.tandfonline.com/doi/abs/10.1207/s15327752jpa6703_13> |
| CDI | Children’s Depression Inventory | 0 | 54 | Yes | <https://www.hindawi.com/journals/drt/2011/134179/> |
| CDI_T | Children’s Depression Inventory – T-scores | 0 | 100 | Yes | <https://www.hindawi.com/journals/drt/2011/134179/> |
| HAM-D | Hamilton Rating Scale – Depression | 0 | 50 | Yes | <https://www.mdcalc.com/hamilton-depression-rating-scale-ham-d> |
| GRID-HAMD | GRID-Hamilton Depression Rating Scale | 0 | 52 | Yes | <https://louisville.edu/depression/outreach-education/HAMD17.pdf> |
| PHQ9 | Patient Health Questionnaire 9 | 0 | 27 | Yes | <https://www.med.umich.edu/1info/FHP/practiceguides/depress/phq-9.pdf> |
| DASS | Depression Anxiety Stress Scale (DASS) – depression subscale | 0 | 42 | Yes | <https://www.karger.com/Article/Fulltext/485182#ref9> (7 items; score 0-3; X 2) |
| RADS-2 | Reynolds Adolescent Depression Scale – Second Edition | 30 | 120 | Yes | <https://thekeep.eiu.edu/cgi/viewcontent.cgi?referer=https://www.google.com/&httpsredir=1&article=2757&context=theses> |
| VAS | Clinician-rated visual analog scale | 0 | 100 | Yes | <https://www.kcl.ac.uk/research/m3vas> |
| BASC | Behavior Assessment System for Children– Parent Rating Scales (depression subscale; T-scores) | 0 | 100 | Yes | <https://link.springer.com/referenceworkentry/10.1007%2F978-1-4419-1698-3_847> (Full scale: 138 items; score 0-3. Unclear for depression subscale. Appears data are T-scores) |
| SCL | Symptom Checklist-90-Revised – Depression  (separated from change score) | 0 | 40 | Yes | <https://onlinelibrary.wiley.com/doi/full/10.1111/j.1360-0443.2004.00570.x?sid=nlm%3Apubmed> (10 items; score 0-4) |
| SCL_change | Symptom Checklist-90-Revised – Depression – change in score | -40 | 40 | Yes | <http://scireproject.com/wp-content/uploads/Clinician-Summary-v.5.0_SCL-90-R.pdf> |
| CES-D | Centre for Epidemiologic Studies Depression Scale | 0 | 60 | Yes | <https://www.ncbi.nlm.nih.gov/pmc/articles/PMC3585724/> (20 items; score 0-3) |
| POMS | Profile of Mood States questionnaire – depression subscale (change scores) | -60 | 60 | Yes | <https://www.brianmac.co.uk/poms.htm> (raw scores 0-60) |
| ASR | Adult Self Report Depressive Problems Subscale (T-scores) | 0 | 100 | Yes | <https://nda.nih.gov/data_structure.html?short_name=abcd_asrs01> (raw scores 0-28) |
| HADS | Hospital Anxiety and Depression Scale | 0 | 21 | Yes | <https://academic.oup.com/occmed/article/64/5/393/1436876> |

### Table 11 Assessment of reporting bias

| Included comparisons present in excluded studies | Number of trials |
| --- | --- |
| ABA versus NoAdditionalIntervention | 29 |
| ABA+MedicationDiuretic versus ABA | 0 |
| BookReading versus NoAdditionalIntervention | 5 |
| CBTAdaptedFamilyBased versus CBTAdaptedGroup | 0 |
| CBTAdaptedFamilyBased versus NoAdditionalIntervention | 2 |
| CBTAdaptedGroup versus NoAdditionalIntervention | 4 |
| CBTAdaptedIndividual versus NoAdditionalIntervention | 11 |
| CBTAdaptedParentMediated versus NoAdditionalIntervention | 1 |
| CBTAdaptedSelfDirected versus NoAdditionalIntervention | 0 |
| CBTFamilyBasedExposureFocussed versus NoAdditionalIntervention | 1 |
| CBTNonAdaptedFamilyBased versus NoAdditionalIntervention | 0 |
| CBTNonAdaptedGroup versus NoAdditionalIntervention | 8 |
| CBTNonAdaptedIndividual versus CBTAdaptedIndividual | 2 |
| CBTNonAdaptedIndividual versus NoAdditionalIntervention | 9 |
| CBTNonAdaptedIndividual+MedicationMelatonin versus CBTNonAdaptedIndividual | 0 |
| CBTNonAdaptedIndividual+MedicationMelatonin versus NoAdditionalIntervention | 0 |
| CBTNonAdaptedSelfDirected versus NoAdditionalIntervention | 2 |
| Counselling versus SkillsTrainingMASSI | 0 |
| DietarySupplement versus NoAdditionalIntervention | 118 |
| Distraction versus NoAdditionalIntervention | 0 |
| GroupActivity versus CBTAdaptedGroup | 0 |
| IndividualCBT versus NoAdditionalIntervention | 0 |
| MedicationADH versus NoAdditionalIntervention | 5 |
| MedicationAtypicalAntiPsychotic versus NoAdditionalIntervention | 49 |
| MedicationMDMA versus NoAdditionalIntervention | 1 |
| MedicationMelatonin versus CBTNonAdaptedIndividual | 0 |
| MedicationMelatonin versus CBTNonAdaptedIndividual+MedicationMelatonin | 0 |
| MedicationMelatonin versus NoAdditionalIntervention | 8 |
| MedicationNAC versus NoAdditionalIntervention | 8 |
| MedicationNaSSA versus NoAdditionalIntervention | 0 |
| MedicationOxytocin versus NoAdditionalIntervention | 78 |
| MedicationSNRI versus NoAdditionalIntervention | 12 |
| MedicationSSRI versus NoAdditionalIntervention | 23 |
| Mindfulness versus CBTNonAdaptedSelfDirected | 0 |
| Mindfulness versus NoAdditionalIntervention | 7 |
| OpioidReceptorAntagonist versus NoAdditionalIntervention | 2 |
| ParentPsychoeducation versus NoAdditionalIntervention | 51 |
| SensoryIntegrationTherapy+ThaiTraditionalMassage versus SensoryIntegrationTherapy | 0 |
| SkillsTraining versus NoAdditionalIntervention | 107 |
| SkillsTrainingGroup versus NoAdditionalIntervention | 22 |
| SkillsTrainingGroupPEERS versus NoAdditionalIntervention | 11 |
| SkillsTrainingGroupSENSE versus NoAdditionalIntervention | 1 |
| SkillsTrainingIndividual versus NoAdditionalIntervention | 7 |
| SkillsTrainingMASSI versus NoAdditionalIntervention | 1 |
| SkillsTrainingSelfdirected versus CBTNonAdaptedSelfDirected | 0 |
| SkillsTrainingVideo versus Distraction | 0 |
| SkillsTrainingVideo versus NoAdditionalIntervention | 23 |
| SkillsTrainingVideo+Distraction versus Distraction | 0 |
| SkillsTrainingVideo+Distraction versus NoAdditionalIntervention | 0 |
| SkillsTrainingVideo+Distraction versus SkillsTrainingVideo | 0 |

## Figures

### Figure 1 Reference flow


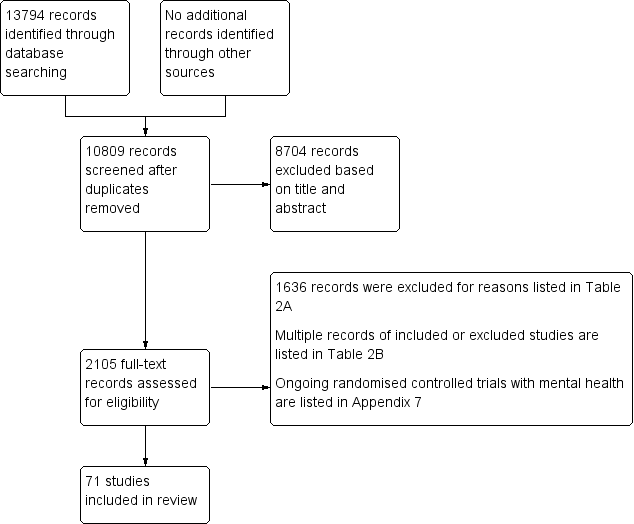


### Figure 2 Network plots


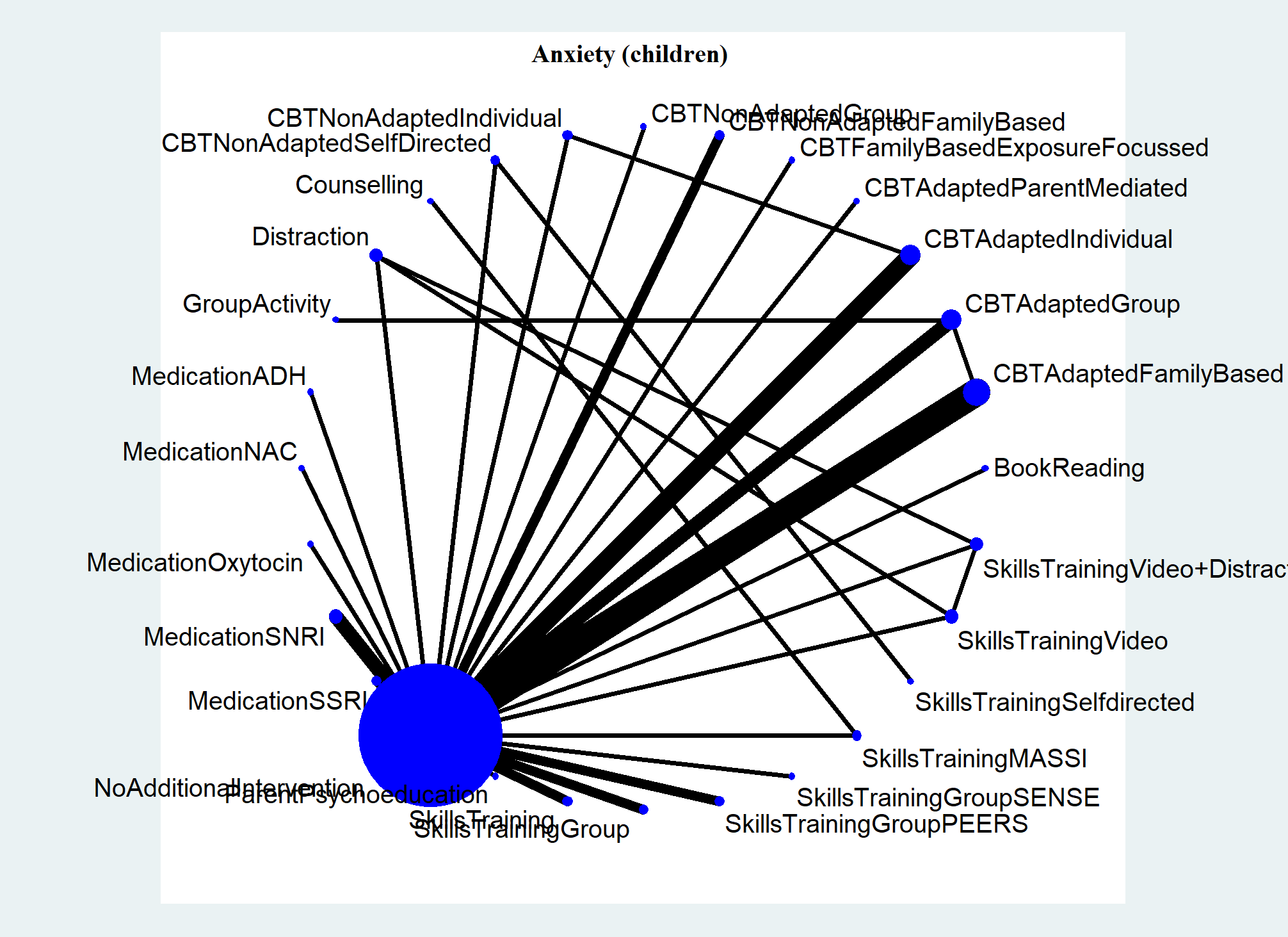


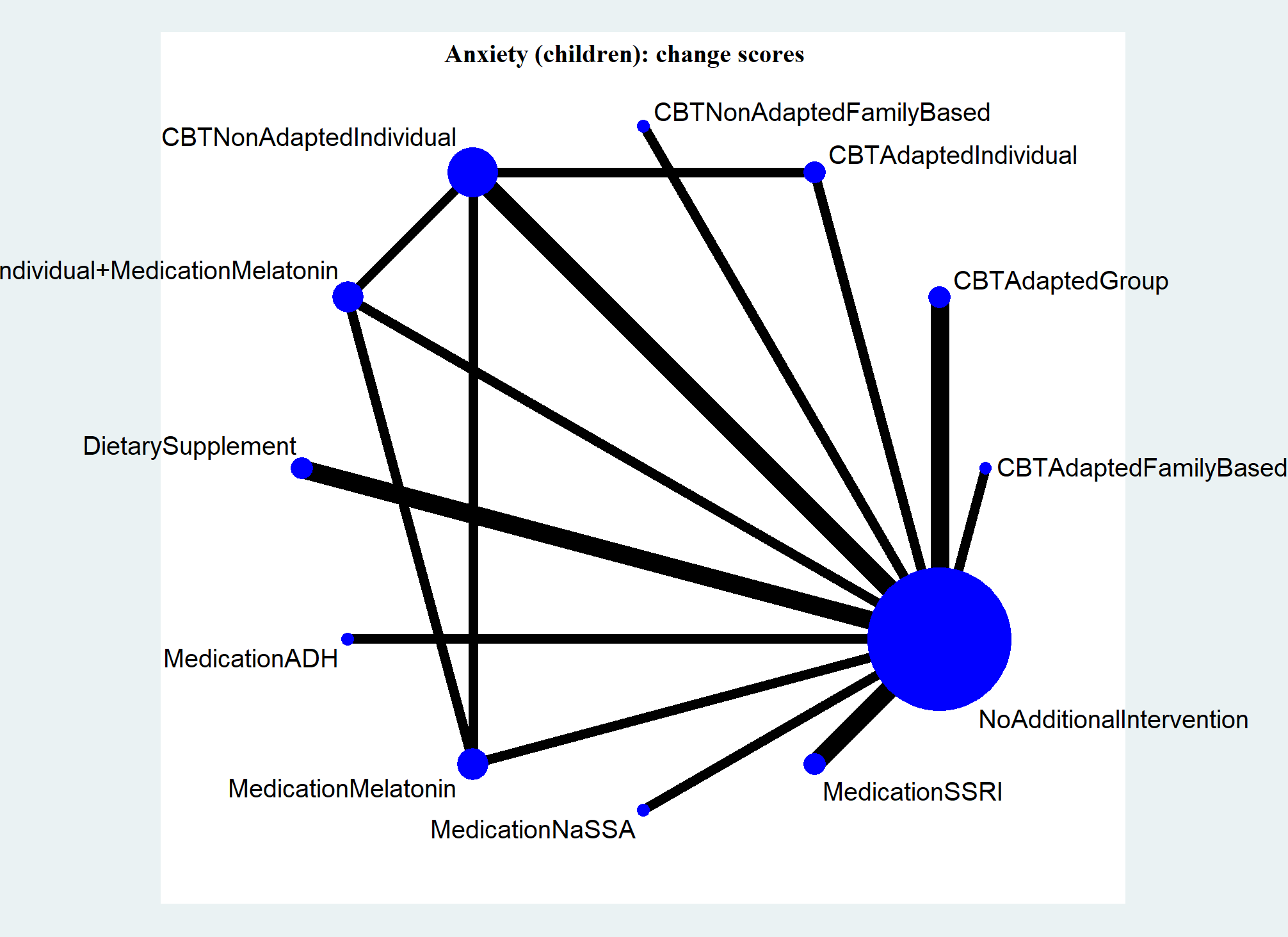


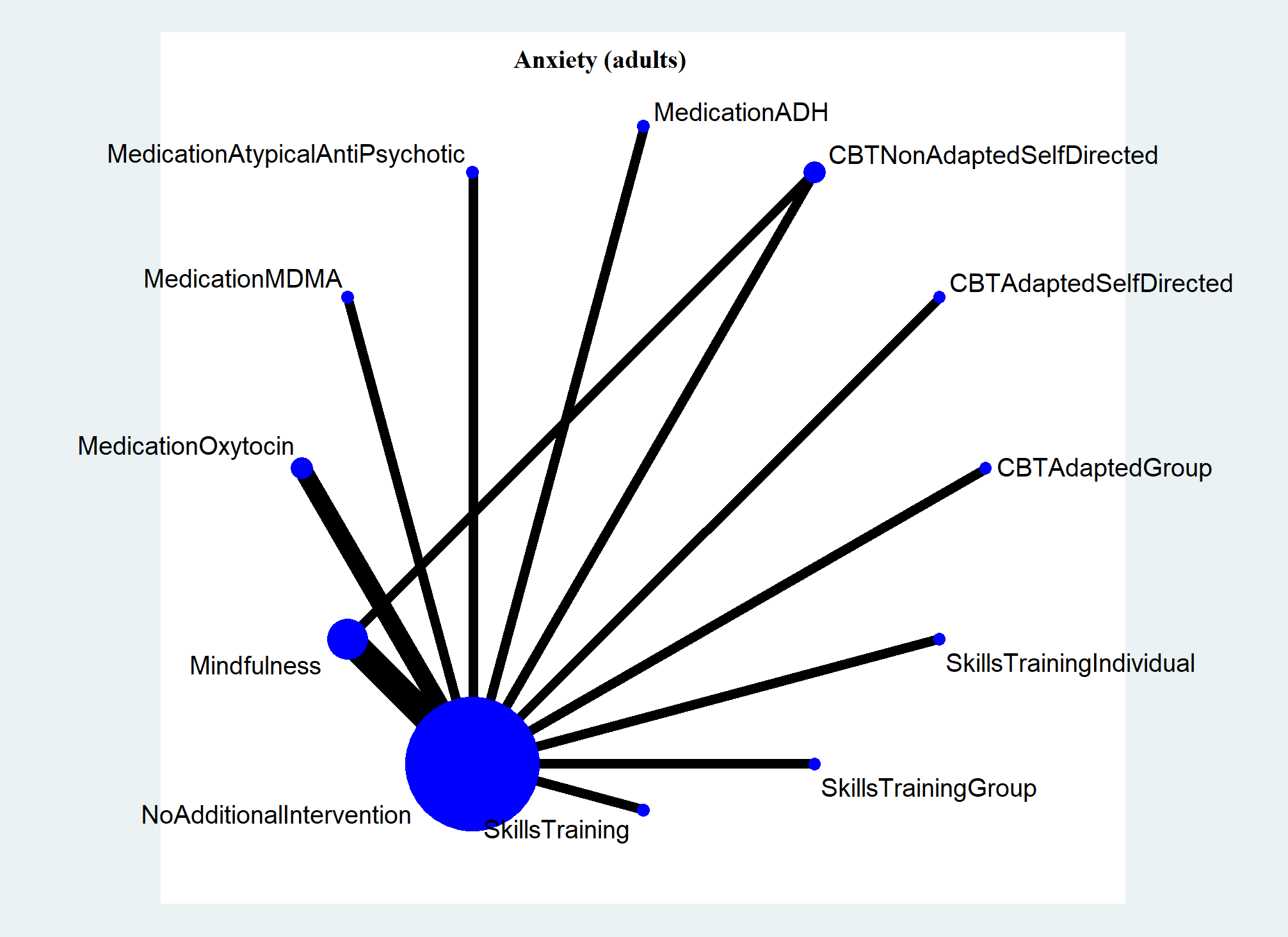


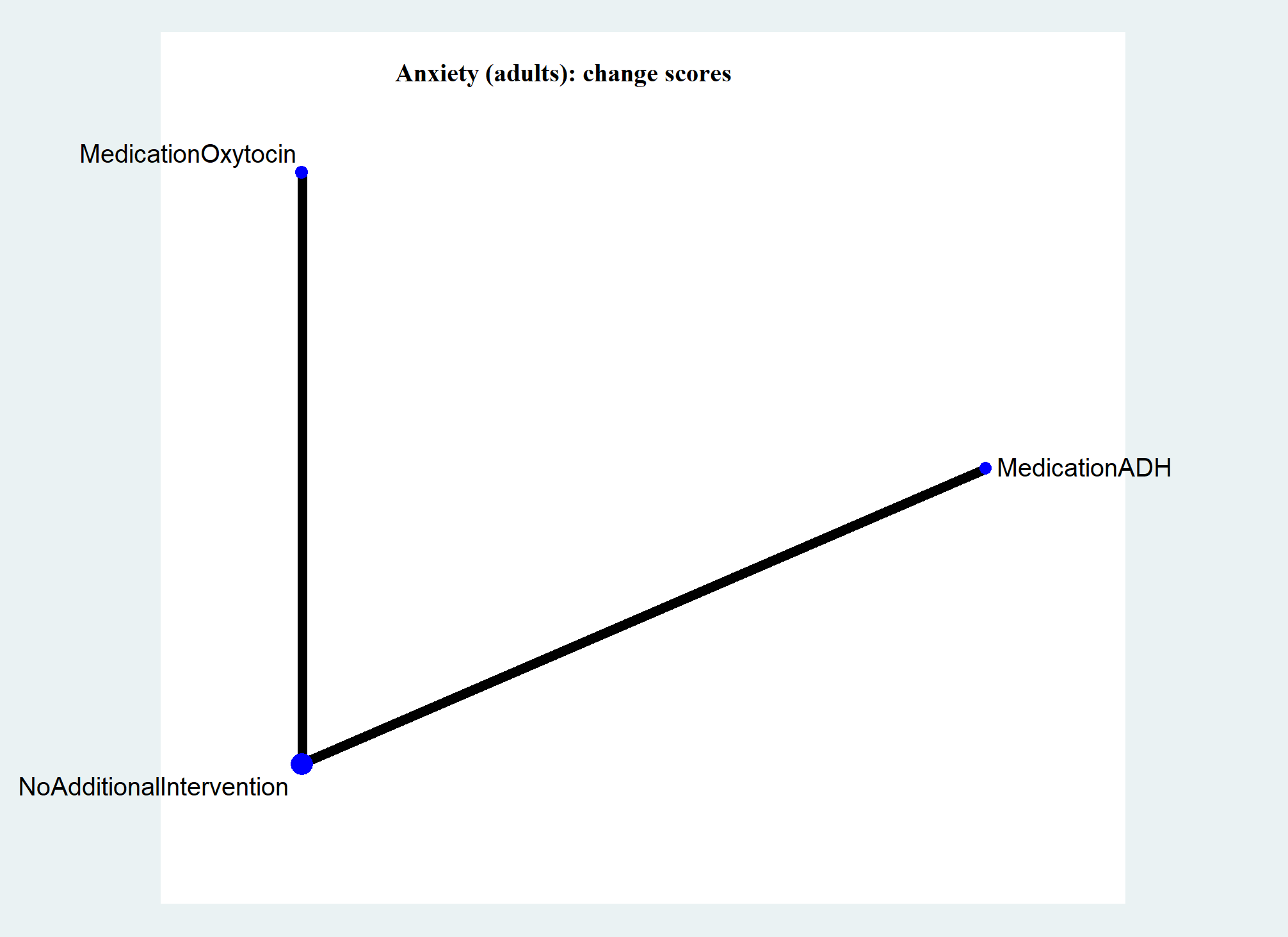


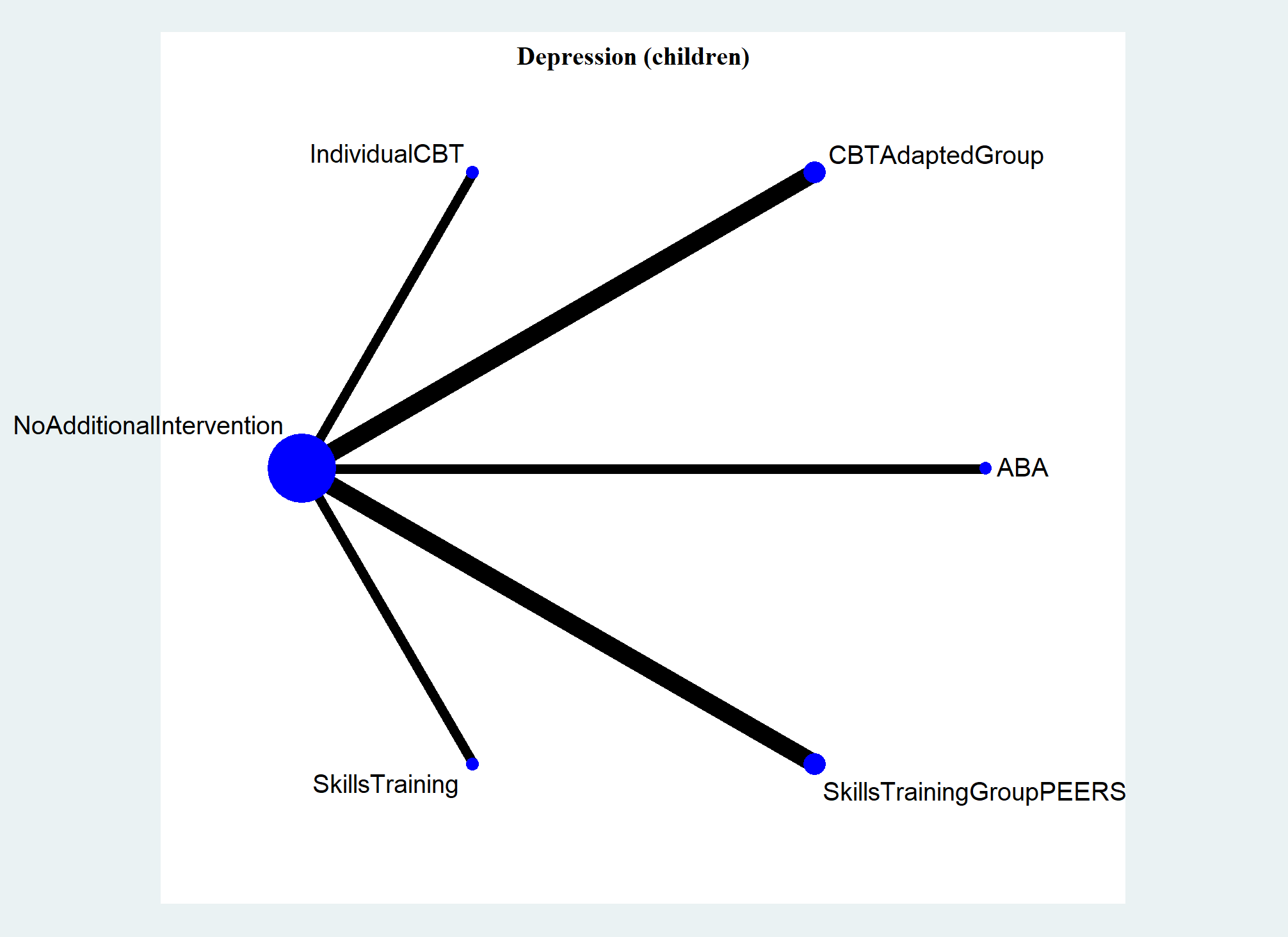


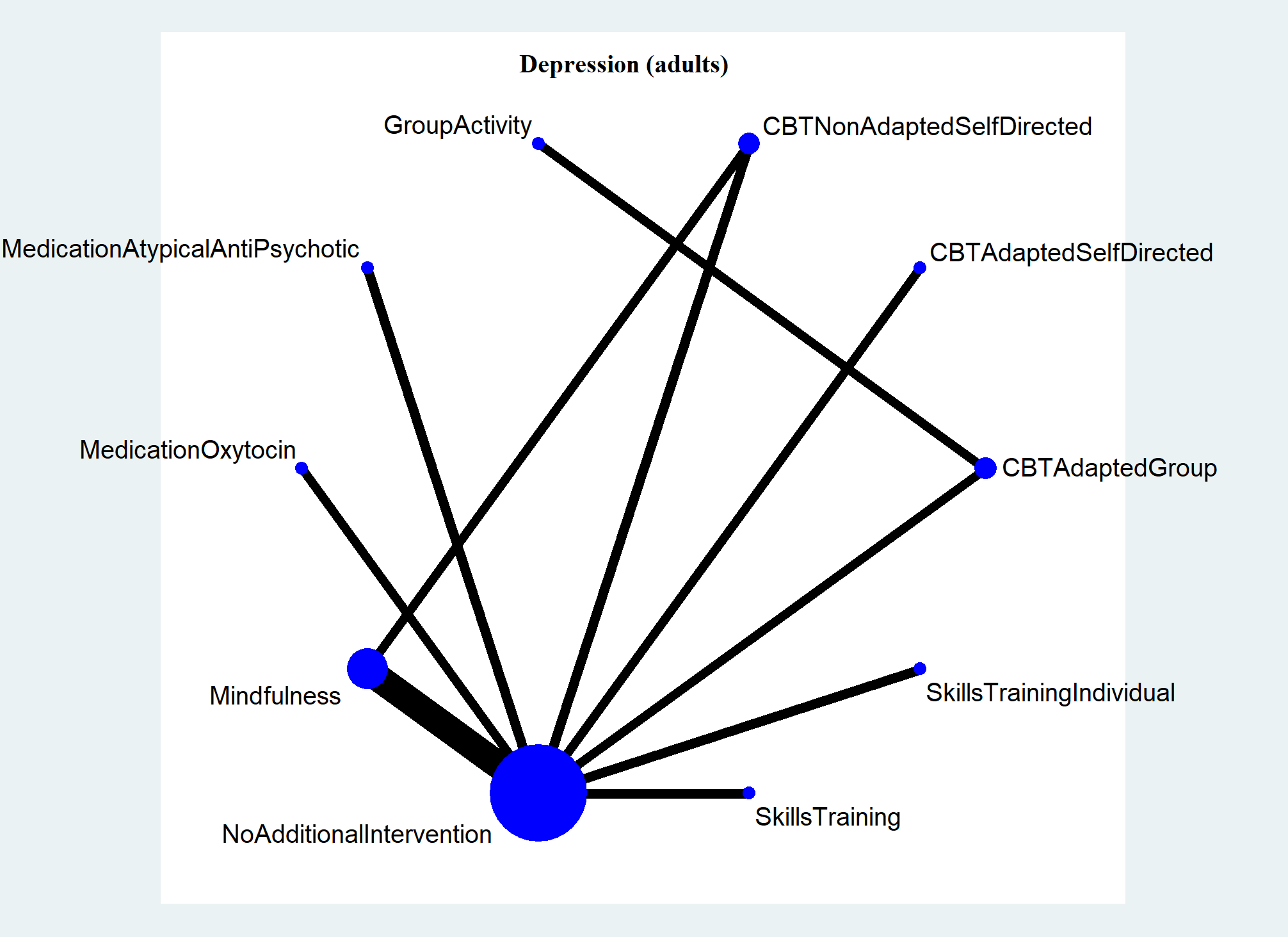


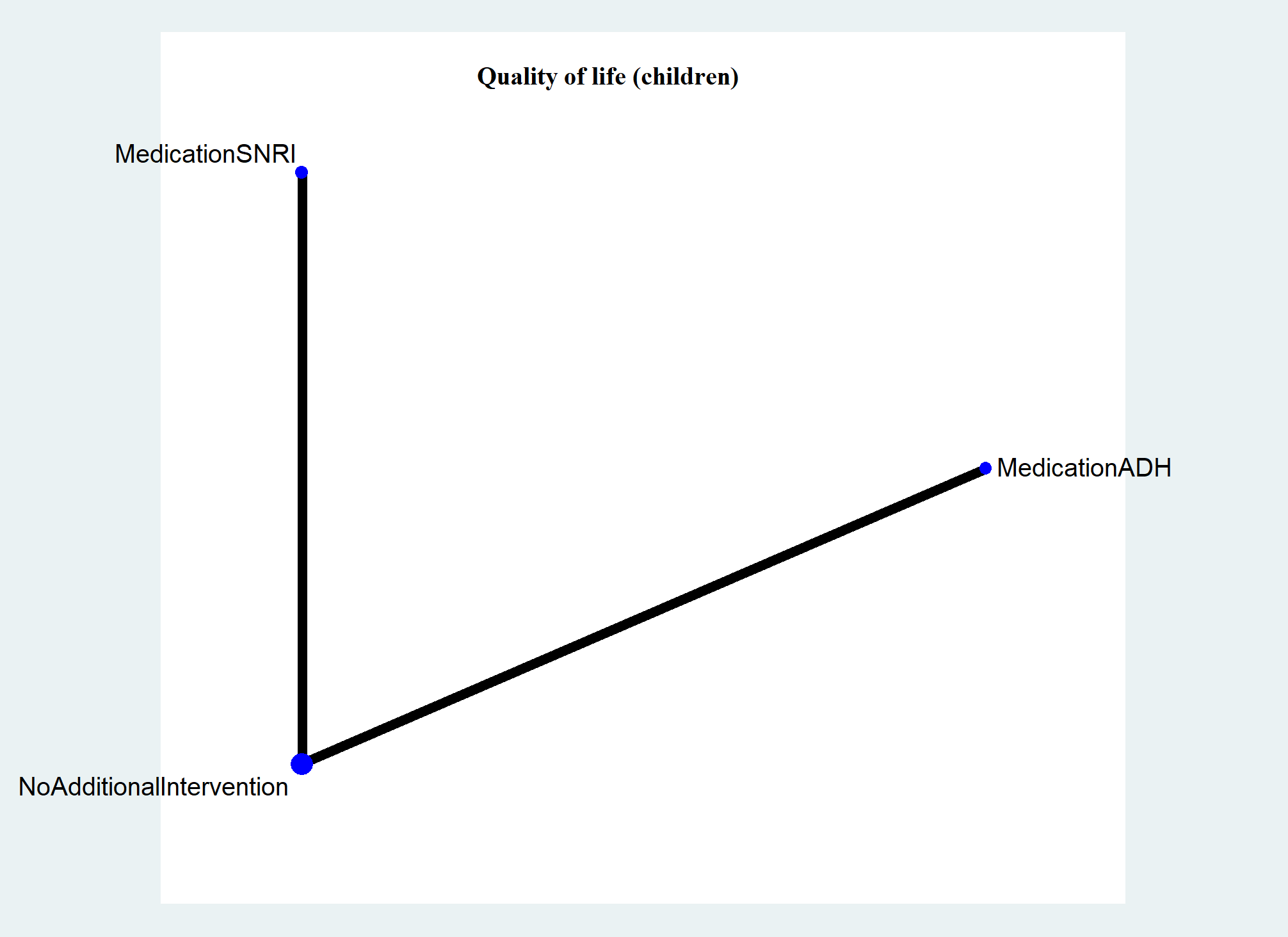


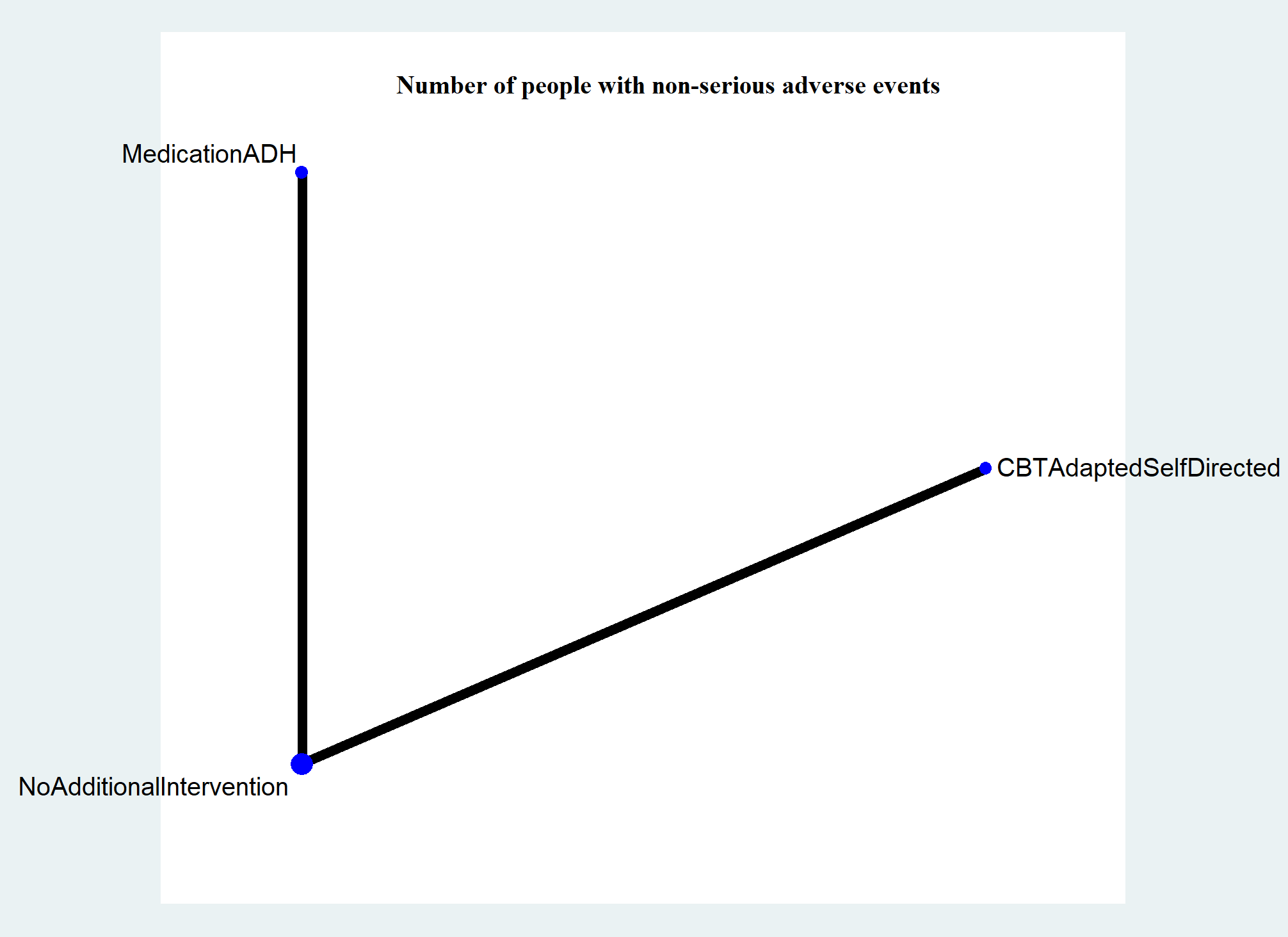


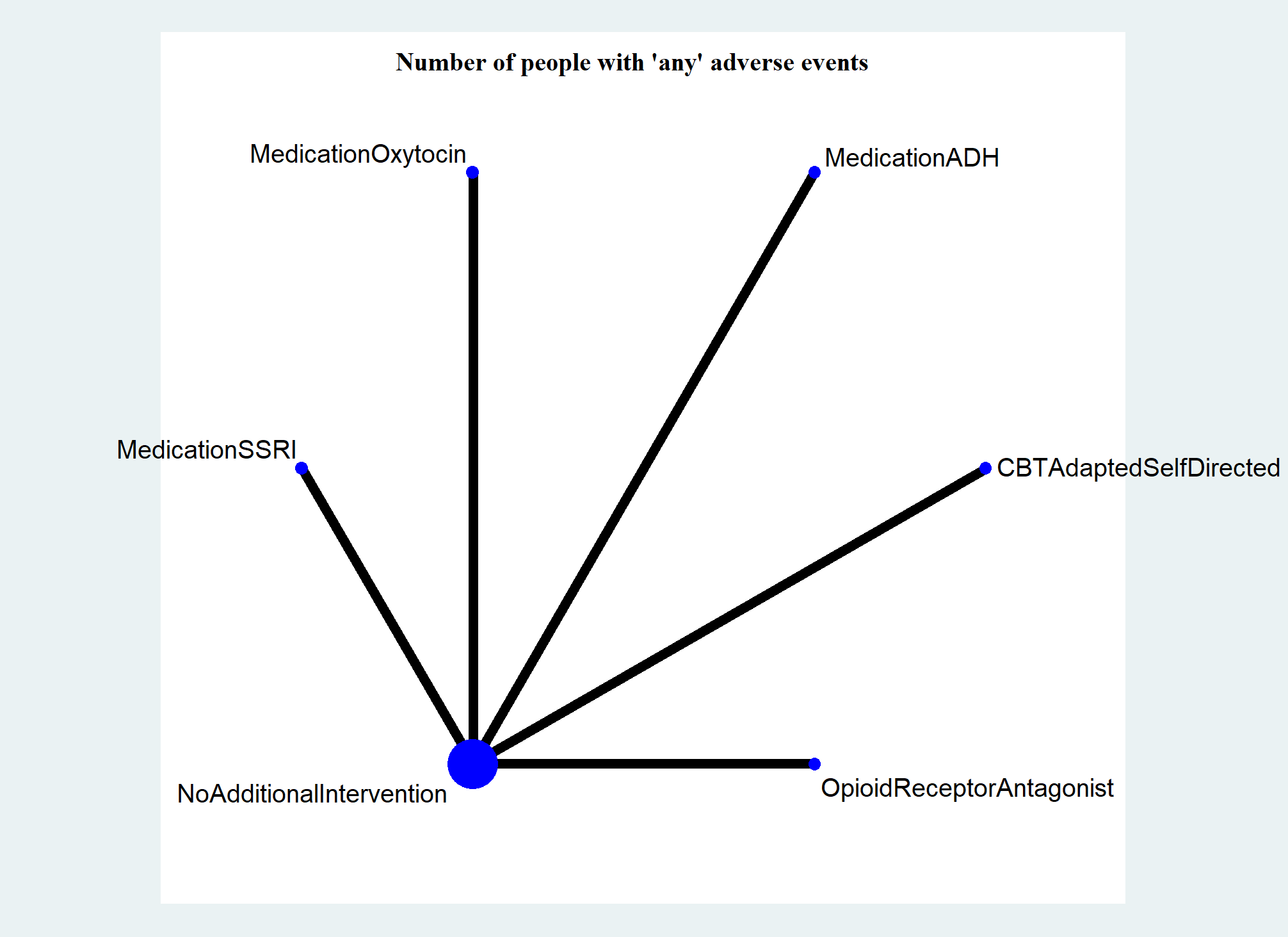


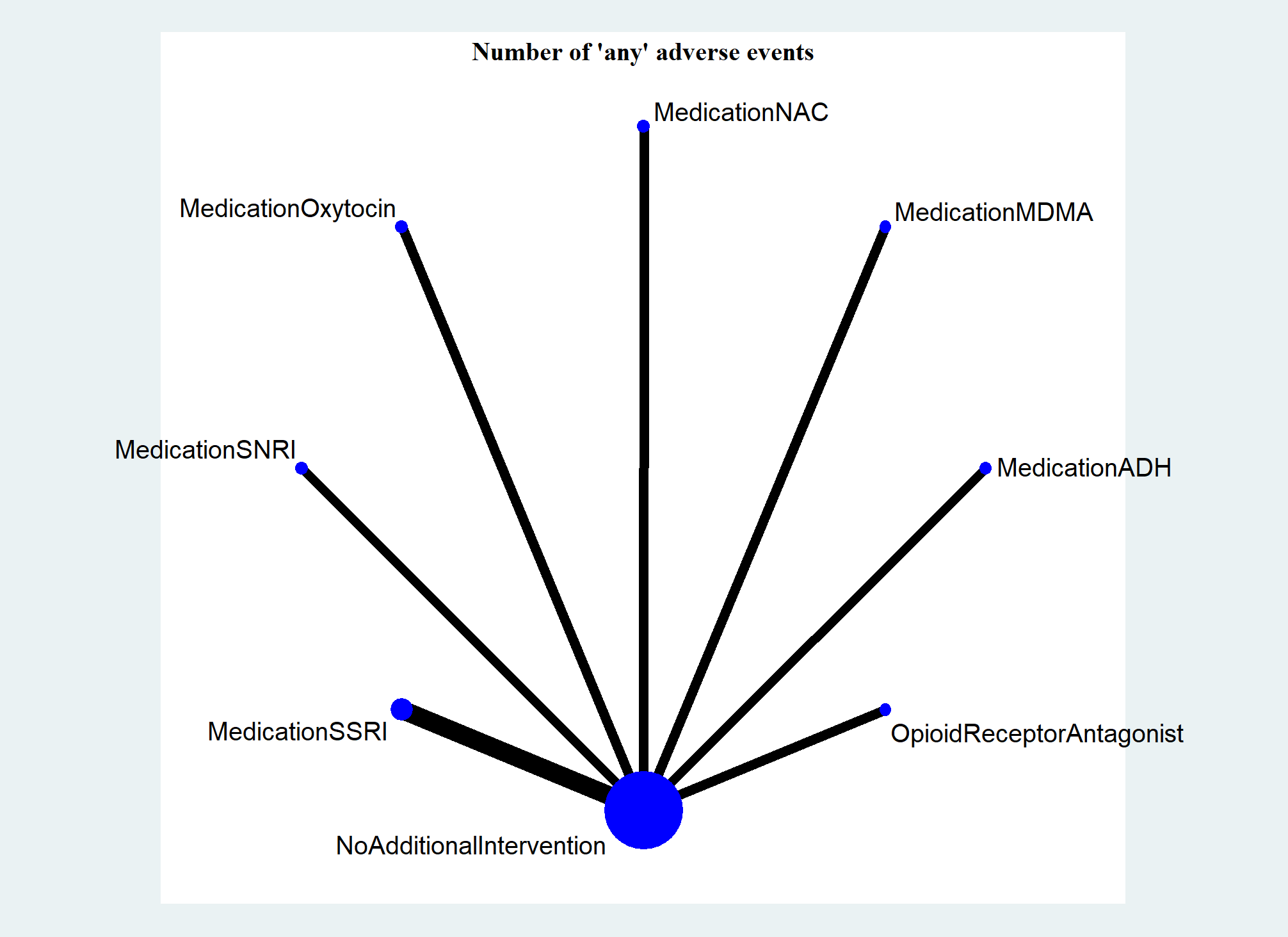


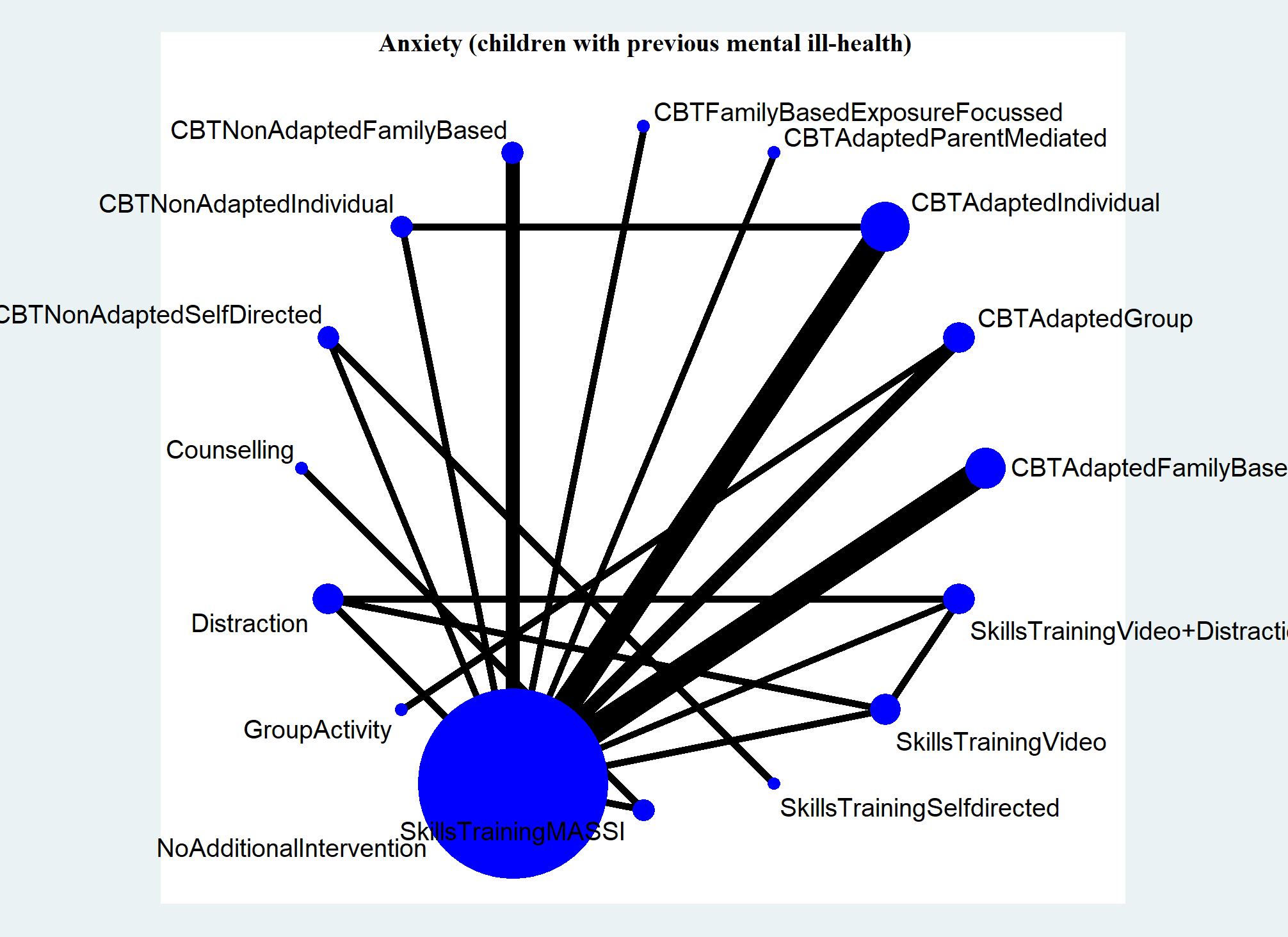


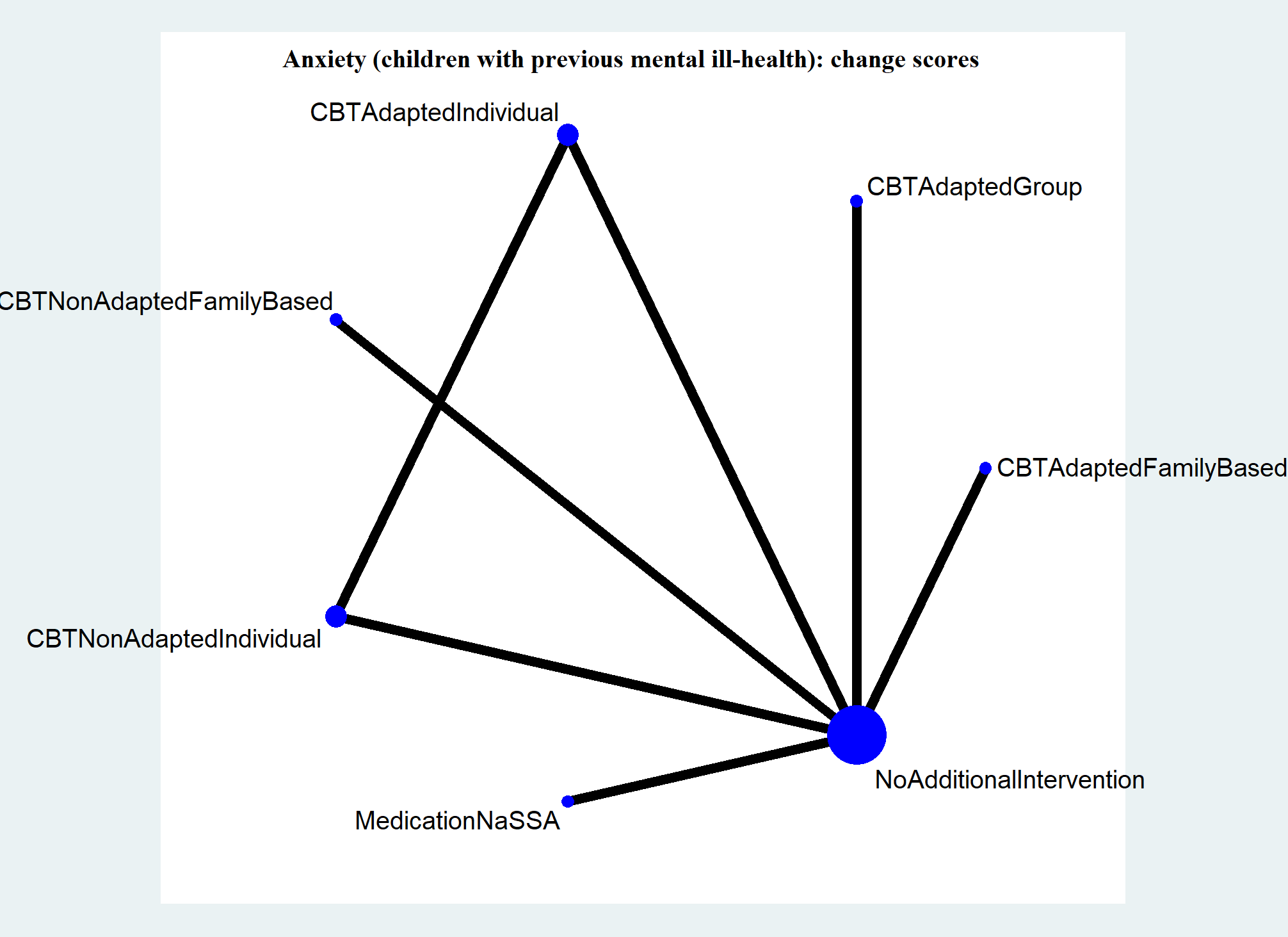


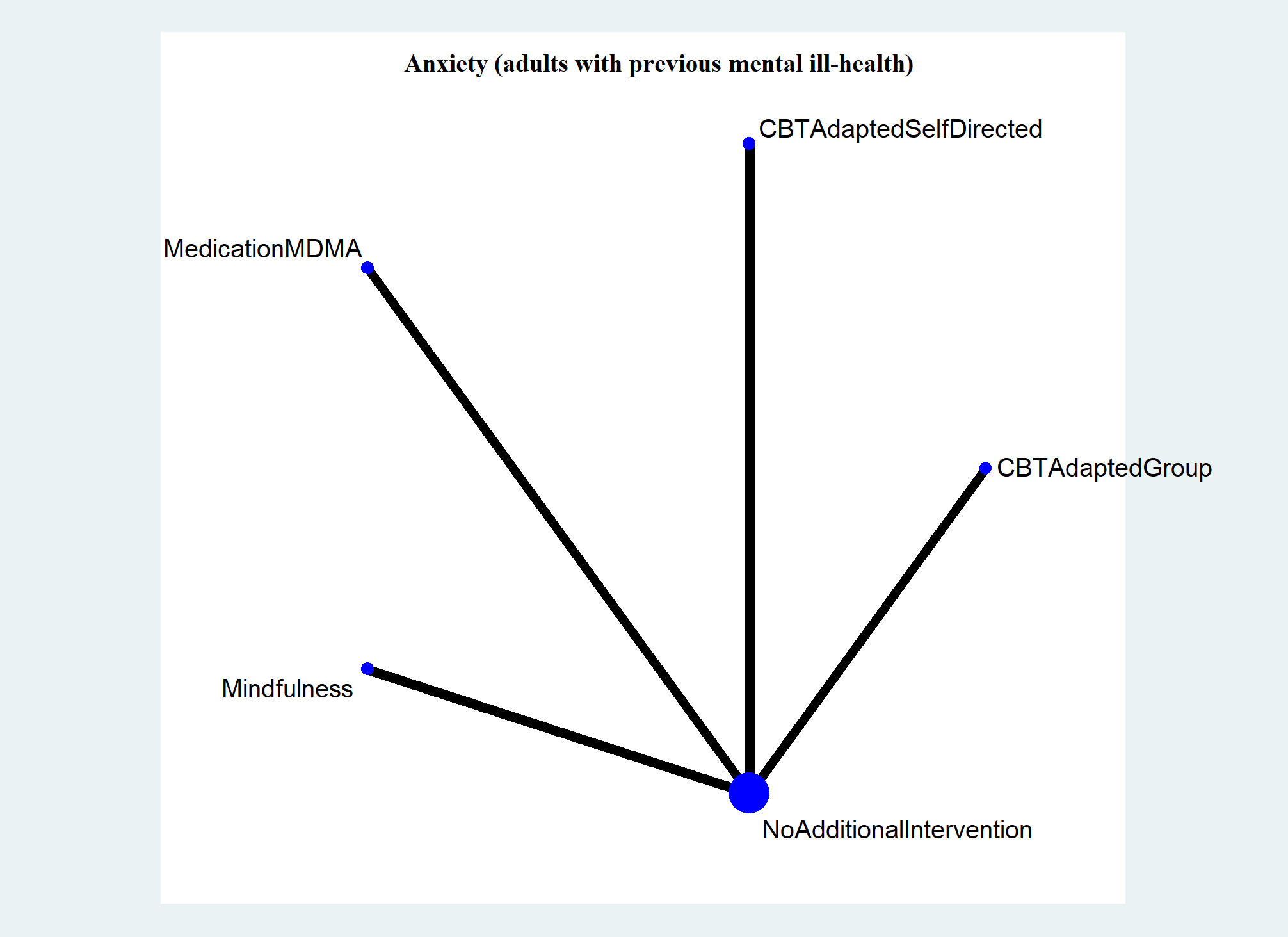


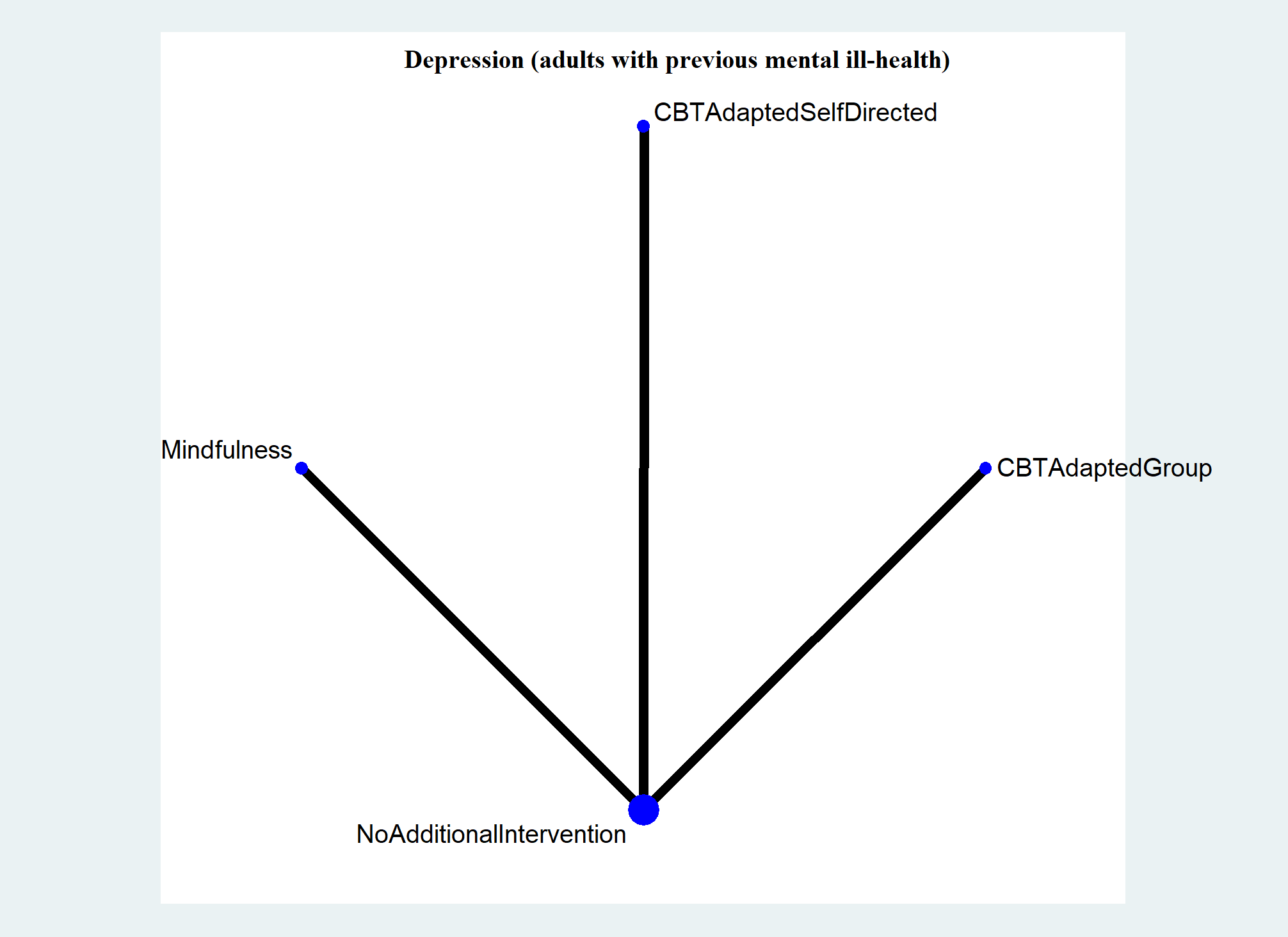


The network plots showing the outcomes for which network meta-analysis was performed. The size of the node (circle) provides a measure of the number of trials in which the particular intervention was included as one of the intervention groups. The thickness of the line provides a measure of the number of direct comparisons between two nodes (Interventions).

### Figure 3 Inconsistency factor plot


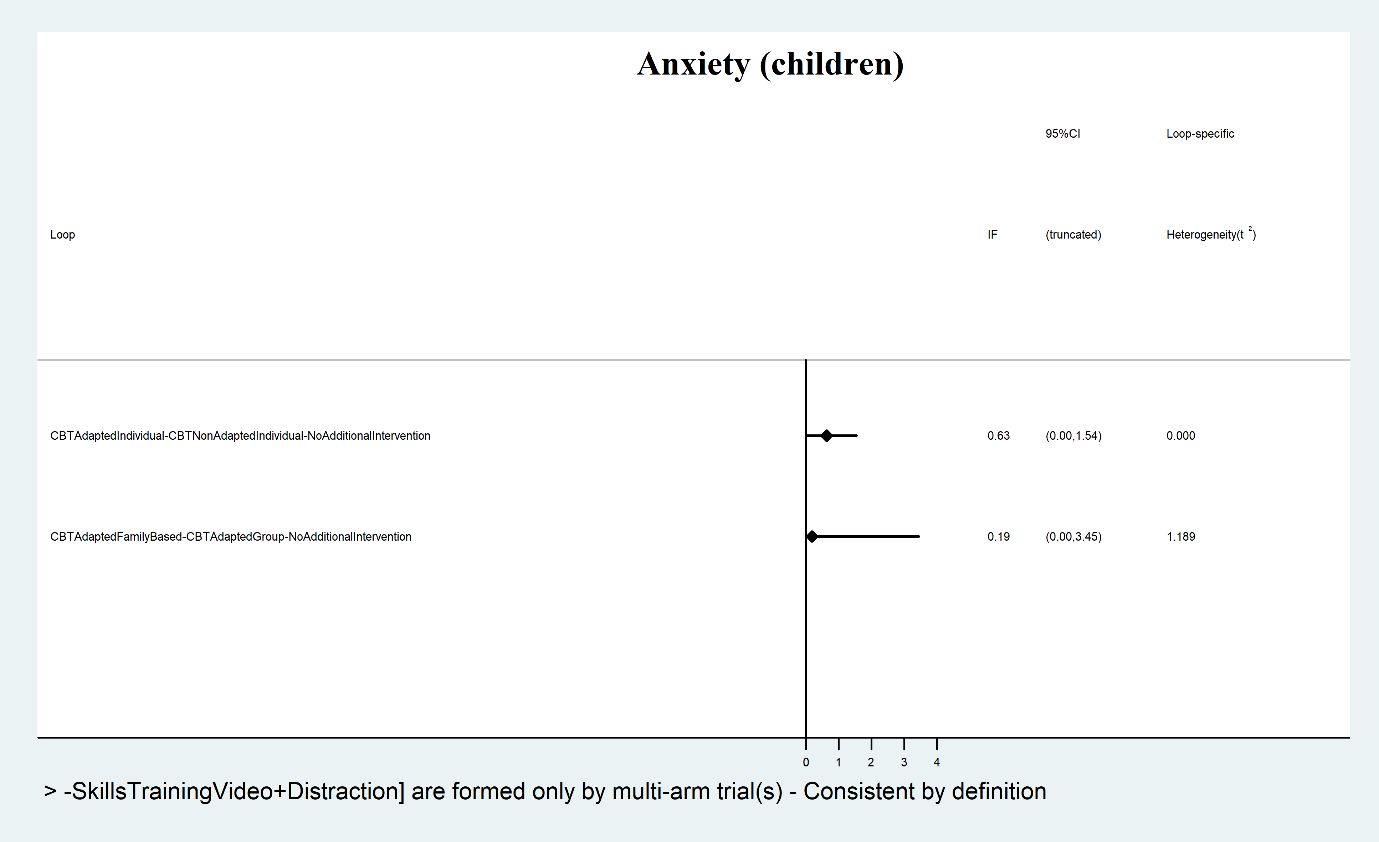


Inconsistency factor plot which shows that there is no evidence of inconsistency in any of the loops involving direct and indirect comparisons.

### Figure 4 Forest plots

## Appendices

### Appendix 1 Search strategy

Date of search: 17 October 2020

Database (number of references retrieved)

#### Medline (3354)

1. exp child development disorders, pervasive/

2. ((Pervasive adj2 Development) or PDD or PDDs or ASD or ASDs or autis* or asperger* or kanner* or (childhood adj1 schizophreni*) or ((communicat* or speech) adj3 disorder*) or (language adj3 delay*)).tw.

3. 1 or 2

4. mental disorders/ or exp adjustment disorders/ or exp anxiety disorders/ or exp mood disorders/ or neurotic disorders/ or exp Self-Injurious Behavior/ or exp Self Mutilation/ or exp Suicide/

5. (anxi* or depress* or melancholi* or neuros* or neurotic or psychoneuro* or stress* or distress* or emotion* or self-harm* or "self harm" or selfharm or DSH or self-injur* or "self injury" or selfinjury or "self injurious" or selfinjurious or "self injuring" or selfinjuring or self-cutting or "self cutting" or self-directed or "self directed" or selfdirected or self-inflicted or "self inflicted" or selfinflicated or self-destruct* or "self destructive" or auto-destruct* or self-mutilation or "self mutilation" or selfmutilation or auto-mutilat* or "auto mutilation" or automutilation or suicide or suicidal* or suicides or self-killing or "self killing" or selfkilling).tw.

6. affective symptom*.tw.

7. 4 or 5 or 6

8. 3 and 7

9. randomized controlled trial.pt.

10. controlled clinical trial.pt.

11. randomized.ab.

12. placebo.ab.

13. drug therapy.fs.

14. randomly.ab.

15. trial.ab.

16. groups.ab.

17. 9 or 10 or 11 or 12 or 13 or 14 or 15 or 16

18. exp animals/ not humans.sh.

19. 17 not 18

20. 8 and 19

#### Embase (2004)

1. exp autism/

2. ((Pervasive adj2 Development) or PDD or PDDs or ASD or ASDs or autis* or asperger* or kanner* or (childhood adj1 schizophreni*) or ((communicat* or speech) adj3 disorder*) or (language adj3 delay*)).tw.

3. 1 or 2

4. mental disease/ or adjustment disorder/ or exp anxiety disorder/ or exp neurosis/

5. exp "psychological and psychiatric procedures, techniques and concepts"/

6. exp mood disorder/

7. exp stress/

8. exp emotion/

9. emotional disorder/

10. anxiety/

11. exp automutilation/ or exp suicide/

12. (anxi* or depress* or melancholi* or neuros* or neurotic or psychoneuro* or stress* or distress* or emotion* or self-harm* or "self harm" or selfharm or DSH or self-injur* or "self injury" or selfinjury or "self injurious" or selfinjurious or "self injuring" or selfinjuring or self-cutting or "self cutting" or self-directed or "self directed" or selfdirected or self-inflicted or "self inflicted" or selfinflicated or self-destruct* or "self destructive" or auto-destruct* or self-mutilation or "self mutilation" or selfmutilation or auto-mutilat* or "auto mutilation" or automutilation or suicide or suicidal* or suicides or self-killing or "self killing" or selfkilling).tw.

13. affective symptom*.tw.

14. 4 or 5 or 6 or 7 or 8 or 9 or 10 or 11 or 12 or 13

15. 3 and 14

16. exp crossover-procedure/ or exp double-blind procedure/ or exp randomized controlled trial/ or single-blind procedure/

17. (((((random* or factorial* or crossover* or cross over* or cross-over* or placebo* or double*) adj blind*) or single*) adj blind*) or assign* or allocat* or volunteer*).af.

18. 16 or 17

19. 15 and 18

#### PsycINFO (2091)

1. exp pervasive developmental disorders/

2. ((Pervasive adj2 Development) or PDD or PDDs or ASD or ASDs or autis* or asperger* or kanner* or (childhood adj1 schizophreni*) or ((communicat* or speech) adj3 disorder*) or (language adj3 delay*)).tw.

3. 1 or 2

4. exp stress/

5. exp emotional states/

6. exp Self-Destructive Behavior/ or exp Self-Injurious Behavior/ or exp Suicide/ or exp Suicidal Ideation/

7. (anxi* or depress* or melancholi* or neuros* or neurotic or psychoneuro* or stress* or distress* or emotion* or self-harm* or "self harm" or selfharm or DSH or self-injur* or "self injury" or selfinjury or "self injurious" or selfinjurious or "self injuring" or selfinjuring or self-cutting or "self cutting" or self-directed or "self directed" or selfdirected or self-inflicted or "self inflicted" or selfinflicated or self-destruct* or "self destructive" or auto-destruct* or self-mutilation or "self mutilation" or selfmutilation or auto-mutilat* or "auto mutilation" or automutilation or suicide or suicidal* or suicides or self-killing or "self killing" or selfkilling).tw.

8. 4 or 5 or 6 or 7

9. double-blind.tw.

10. random: assigned.tw.

11. control.tw.

12. 9 or 10 or 11

13. 3 and 8 and 12

#### CINAHL Plus (1015)

RCT filter: Health Info Libr J. 2019 Mar;36(1):73-90. doi: 10.1111/hir.12251. Epub 2019 Feb 8. Development of a search filter to identify reports of controlled clinical trials within CINAHL Plus. Glanville J1, Dooley G2, Wisniewski S3, Foxlee R4, Noel-Storr A4.

S1 (MH "Child Development Disorders, Pervasive+")

S2 TI ( ((Pervasive N2 Development) or PDD or PDDs or ASD or ASDs or autis* or asperger* or kanner* or (childhood N1 schizophreni*) or ((communicat* or speech) N3 disorder*) or (language N3 delay*)) ) OR AB ( ((Pervasive N2 Development) or PDD or PDDs or ASD or ASDs or autis* or asperger* or kanner* or (childhood N1 schizophreni*) or ((communicat* or speech) N3 disorder*) or (language N3 delay*)) )

S3 S1 OR S2

S4 (MH "Mental Health") or (MH "Injuries, Self-Inflicted") or (MH "Suicide+")

S5 (MH "Stress, Psychological+")

S6 (MH "Affective Symptoms+")

S7 TI ( (anxi* or depress* or melancholi* or neuros* or neurotic or psychoneuro* or stress* or distress* or emotion* or self-harm* or "self harm" or selfharm or DSH or self-injur* or "self injury" or selfinjury or "self injurious" or selfinjurious or "self injuring" or selfinjuring or self-cutting or "self cutting" or self-directed or "self directed" or selfdirected or self-inflicted or "self inflicted" or selfinflicated or self-destruct* or "self destructive" or auto-destruct* or self-mutilation or "self mutilation" or selfmutilation or auto-mutilat* or "auto mutilation" or automutilation or suicide or suicidal* or suicides or self-killing or "self killing" or selfkilling) ) OR AB ( (anxi* or depress* or melancholi* or neuros* or neurotic or psychoneuro* or stress* or distress* or emotion* or self-harm* or "self harm" or selfharm or DSH or self-injur* or "self injury" or selfinjury or "self injurious" or selfinjurious or "self injuring" or selfinjuring or self-cutting or "self cutting" or self-directed or "self directed" or selfdirected or self-inflicted or "self inflicted" or selfinflicated or self-destruct* or "self destructive" or auto-destruct* or self-mutilation or "self mutilation" or selfmutilation or auto-mutilat* or "auto mutilation" or automutilation or suicide or suicidal* or suicides or self-killing or "self killing" or selfkilling) )

S8 TI affective symptom* OR AB affective symptom*

S9 S4 OR S5 OR S6 OR S7 OR S8

S10 S3 AND S9

S11 MH randomized controlled trials

S12 MH double-blind studies

S13 MH single-blind studies

S14 MH random assignment

S15 MH pretest-posttest design

S16 MH cluster sample

S17 TI (randomised OR randomized)

S18 AB (random*)

S19 TI (trial)

S20 MH (sample size) AND AB (assigned ORallocated OR control)

S21 MH (placebos)

S22 PT (randomized controlled trial)

S23 AB (control W5 group)

S24 MH (crossover design) OR MH(comparative studies)

S25 AB (cluster W3 RCT)

S26 MH animals+

S27 MH (animal studies)

S28 TI (animal model*)

S29 S26 OR S27 OR S28

S30 MH (human)

S31 S29 NOT S30

S32 S11 OR S12 OR S13 OR S14 OR S15 OR S16 OR S17 OR S18 OR S19 OR S20 OR S21 OR S22 OR S23 OR S24 OR S25

S33 S32 NOT S31

S34 S10 AND S33

#### Cochrane (1949)

#1 MeSH descriptor: [Child Development Disorders, Pervasive] explode all trees

#2 (Pervasive adj2 Development) or PDD or PDDs or ASD or ASDs or autis* or asperger* or kanner* or (childhood near/1 schizophreni*) or ((communicat* or speech) near/3 disorder*) or (language near/3 delay*)

#3 #1 or #2

#4 MeSH descriptor: [Mental Disorders] this term only

#5 MeSH descriptor: [Adjustment Disorders] explode all trees

#6 MeSH descriptor: [Anxiety Disorders] explode all trees

#7 MeSH descriptor: [Mood Disorders] explode all trees

#8 MeSH descriptor: [Neurotic Disorders] this term only

#9 MeSH descriptor: [Self-Injurious Behavior] explode all trees

#10 MeSH descriptor: [Self Mutilation] explode all trees

#11 MeSH descriptor: [Suicide] explode all trees

#12 (anxi* or depress* or melancholi* or neuros* or neurotic or psychoneuro* or stress* or distress* or emotion* OR self-harm* OR "self harm" OR selfharm OR DSH OR self-injur* OR "self injury" OR selfinjury OR "self injurious" OR selfinjurious OR "self injuring" OR selfinjuring OR self-cutting OR "self cutting" OR self-directed OR "self directed" OR selfdirected OR self-inflicted OR "self inflicted" OR selfinflicated OR self-destruct* OR "self destructive" OR auto-destruct* OR self-mutilation OR "self mutilation" OR selfmutilation OR auto-mutilat* OR "auto mutilation" OR automutilation OR suicide OR suicidal* OR suicides OR self-killing OR "self killing" OR selfkilling)

#13 affective symptom*

#14 #4 or #5 or #6 or #7 or #8 or #9 or #10 or #11 or #12 or #13

#15 #3 and #14

#### Science Citation Index (1354)

#1 TS=((Pervasive near/2 Development) or PDD or PDDs or ASD or ASDs or autis* or asperger* or kanner* or (childhood near/1 schizophreni*) or ((communicat* or speech) near/3 disorder*) or (language near/3 delay*))

#2 TS=(anxi* or depress* or melancholi* or neuros* or neurotic or psychoneuro* or stress* or distress* or emotion* or self-harm* or "self harm" or selfharm or DSH or self-injur* or "self injury" or selfinjury or "self injurious" or selfinjurious or "self injuring" or selfinjuring or self-cutting or "self cutting" or self-directed or "self directed" or selfdirected or self-inflicted or "self inflicted" or selfinflicated or self-destruct* or "self destructive" or auto-destruct* or self-mutilation or "self mutilation" or selfmutilation or auto-mutilat* or "auto mutilation" or automutilation or suicide or suicidal* or suicides or self-killing or "self killing" or selfkilling)

#3 TS=(affective symptom*)

#4 #3 OR #2

#5 TS=(random* OR rct* OR crossover OR masked OR blind* OR placebo*)

#6 #5 AND #4 AND #1

#### ClinicalTrials.gov (289)

Interventional Studies | Autism | Phase 2, 3, 4

#### WHO ICTRP (1738)

Condition: autism

#### Total = 13794

### Appendix 2 Abbreviations

| Interventions: short name | Interventions: full name |
| --- | --- |
| ABA | applied behavioural analysis |
| ABA+MedicationDiuretic | ABA plus diuretic |
| BookReading | book reading |
| CBT | cognitive behavioural therapy |
| CBTAdaptedFamilyBased | family-based adapted CBT |
| CBTAdaptedGroup | group adapted CBT |
| CBTAdaptedIndividual | individual adapted CBT |
| CBTAdaptedParentMediated | parent-mediated adapted CBT |
| CBTAdaptedSelfDirected | self-directed adapted CBT |
| CBTFamilyBasedExposureFocussed | family-based exposure-focussed CBT |
| CBTNonAdaptedFamilyBased | family-based non-adapted CBT |
| CBTNonAdaptedGroup | group non-adapted CBT |
| CBTNonAdaptedIndividual | individual non-adapted CBT |
| CBTNonAdaptedIndividual+MedicationMelatonin | individual non-adapted CBT plus melatonin |
| CBTNonAdaptedSelfDirected | self-directed non-adapted CBT |
| Counselling | counselling |
| DietarySupplement | dietary supplement |
| Distraction | distraction |
| GroupActivity | group activity |
| IndividualCBT | individual CBT |
| MDMA | 3,4-Methyl​enedioxy​methamphetamine (ecstasy) |
| MedicationADH | anti-diuretic hormone analogue |
| MedicationAtypicalAntiPsychotic | atypical antipsychotic |
| MedicationMDMA | MDMA |
| MedicationMelatonin | melatonin |
| MedicationNAC | N-acetyl cysteine |
| MedicationNaSSA | NaSSA |
| MedicationOxytocin | oxytocin |
| MedicationSNRI | SNRI |
| MedicationSSRI | SSRI |
| Mindfulness | mindfulness |
| NoAdditionalIntervention | no additional intervention |
| OpioidReceptorAntagonist | opioid receptor antagonist |
| ParentPsychoeducation | parent psychoeducation |
| SensoryIntegrationTherapy | sensory integration therapy |
| SensoryIntegrationTherapy+ThaiTraditionalMassage | sensory integration therapy plus Thai traditional massage |
| SkillsTraining | skills training |
| SkillsTrainingGroup | group skills training |
| SkillsTrainingGroupPEERS | PEERS group skills training |
| SkillsTrainingGroupSENSE | SENSE group skills training |
| SkillsTrainingIndividual | individual skills training |
| SkillsTrainingMASSI | MASSI skills training |
| SkillsTrainingSelfdirected | self-directed skills training |
| SkillsTrainingVideo | video skills training |
| SkillsTrainingVideo+Distraction | video skills training plus distraction |
| SNRI | serotonin and norepinephrine reuptake inhibitors |
| SSRI | selective serotonin reuptake inhibitor |

| Outcomes: short name | Outcomes: full name |
| --- | --- |
| AE_Num | Any adverse events (number of events) |
| AE_Prop | Any adverse events (number of people) |
| Anxiety_Adults | Anxiety (scores) (adults) |
| Anxiety_Adults_MH | Anxiety (scores) (adults) (Mental illhealth) |
| Anxiety_Change_Adults | Anxiety (scores) (adults): change |
| Anxiety_Change_Adults_MH | Anxiety (scores) (adults): change (Mental illhealth) |
| Anxiety_Change_Children | Anxiety (scores) (children): change |
| Anxiety_Change_Children_MH | Anxiety (scores) (children): change (Mental illhealth) |
| Anxiety_Children | Anxiety (scores) (children) |
| Anxiety_Children_MH | Anxiety (scores) (children) (Mental illhealth) |
| Anxiety_Depression_Prop | Proportion of patients with anxiety or depression |
| Anxiety_Prop | Proportion of patients with anxiety |
| Depression_Adults | Depression (scores) (adults) |
| Depression_Adults_MH | Depression (scores) (adults) (Mental illhealth) |
| Depression_Change_Adults | Depression (scores) (adults): change |
| Depression_Change_Adults_MH | Depression (scores) (adults): change (Mental illhealth) |
| Depression_Change_Children | Depression (scores) (children): change |
| Depression_Change_Children_MH | Depression (scores) (children): change (Mental illhealth) |
| Depression_Children | Depression (scores) (children) |
| Depression_Children_MH | Depression (scores) (children) (Mental illhealth) |
| Depression_Prop | Proportion of patients with depression |
| Employed_Prop | Proportion of people employed or having meaningful life activities |
| Mortality_Prop | Proportion of people who died |
| MQoL | Mental health-related quality of life (maximal follow-up) |
| NSAE_Num | Non-serious adverse events (number of events) |
| NSAE_Prop | Non-serious adverse events (number of people) |
| QoL_Adults | Quality of life (adults) |
| QoL_Change_Adults | Quality of life (adult): change |
| QoL_Children | Quality of life (children) |
| SAE_Num | Serious adverse events (number of events) |
| SAE_Prop | Serious adverse events (number of people) |
| Self-harm_Prop | Proportion of patients with self-harm |
| Suicidal_Prop | Proportion of patients with suicidal thoughts or attempted suicide |

| Other abbreviations: short name | Other abbreviations: full name |
| --- | --- |
| CrI | credible intervals |
| MD | mean difference |
| OR | odds ratio |
| SMD | standardised mean difference |

### Appendix 3 Data

Please see <https://doi.org/10.5281/zenodo.4844289>.

### Appendix 4 Data analysis (unprocessed)

Please see: <https://doi.org/10.5281/zenodo.4845814>.

### Appendix 5 Sensitivity analysis

Main analysis indicates the analysis without any imputation. The other analyses indicate the sensitivity analyses.

#### Any adverse events (number of people)

##### SSRI versus no additional intervention:

main analysis: no evidence of difference between groups

worst-best analysis: lower in SSRI than no additional intervention

best-worst analysis: higher in SSRI than no additional intervention

##### Self-directed adapted CBT versus no additional intervention:

- main analysis: no evidence of difference between groups
- worst-best analysis: lower in self-directed adapted CBT than no additional intervention
- best-worst analysis: higher in self-directed adapted CBT than no additional intervention

##### SSRI versus anti-diuretic hormone:

- main analysis: no evidence of difference between groups
- worst-best analysis: no evidence of difference between groups
- best-worst analysis: higher in SSRI than anti-diuretic hormone

##### Self-directed adapted CBT versus anti-diuretic hormone:

- main analysis: no evidence of difference between groups
- worst-best analysis: no evidence of difference between groups
- best-worst analysis: higher in self-directed adapted CBT than anti-diuretic hormone

##### Self-directed adapted CBT versus oxytocin:

- main analysis: no evidence of difference between groups
- worst-best analysis: lower in self-directed adapted CBT than oxytocin
- best-worst analysis: higher in self-directed adapted CBT than oxytocin

##### Opioid receptor antagonist versus SSRI:

- main analysis: no evidence of difference between groups
- worst-best analysis: higher in opioid receptor antagonist than SSRI
- best-worst analysis: no evidence of difference between groups

##### Opioid receptor antagonist versus self-directed adapted CBT:

- main analysis: no evidence of difference between groups
- worst-best analysis: higher in opioid receptor antagonist than self-directed adapted CBT
- best-worst analysis: no evidence of difference between groups

### Appendix 6 Results of metaregression

Abbreviations:

Anx = anxiety

Chi~e = change scores in children

Dep = depression

es = effect size

In the above analysis, the differences in core features in autism could not predict the differences in anxiety or depression scores.

### Appendix 7 List of ongoing randomised controlled trials with mental health outcomes

| Study name | Reference |
| --- | --- |
| Actrn 2019a | Actrn (2019). "Transition program for adolescents and young adults with autism in Australia." http://www.who.int/trialsearch/Trial2.aspx?TrialID=ACTRN12619001267145. |
| Actrn 2019b | Actrn (2019). "Use and Effectiveness of the Smiling Mind Smartphone Application on Wellbeing of Children and Adults with Autism Spectrum Disorder and Their Caregivers." http://www.who.int/trialsearch/Trial2.aspx?TrialID=ACTRN12619000584134. |
| Actrn 2020 | ACTRN12620000890932 (2020). A multisite clinical trial of repetitive transcranial magnetic stimulation (rTMS) for social communication in autism spectrum disorder (ASD). |
| Afsharnejad 2019 | Afsharnejad, B., M. Falkmer, M. H. Black, T. Alach, F. Lenhard, A. Fridell, C. Coco, K. Milne, N. T. M. Chen, S. Bolte and S. Girdler (2019). "KONTAKT© for Australian adolescents on the autism spectrum: protocol of a randomized control trial." Trials [Electronic Resource] 20(1): 687. |
| Anagnostou 2019 | Anagnostou, E., H. B. K. R. Hospital, M. University, C. University of Western Ontario, Q. University, T. St. Michael's Hospital, U. Toronto and E. M. D. Anagnostou (2019). "Arbaclofen vs. Placebo in the Treatment of Children and Adolescents With ASD (ARBA)." https://clinicaltrials.gov/ct2/show/NCT03887676 |
| Chi 2017a | Chi, C. I. (2017). "Clinical application and social promtion of parental skills training for children with autism." Http://wwwwhoint/trialsearch/trial2aspx? Trialid=chictr-ioc-17013638. |
| Chi 2017b | Chi, C. I. (2017). "Effect of transcutaneous vagus nerve stimulation at auricular concha on patients with autism spectrum disorder: a random-controlled trail study." Http://wwwwhoint/trialsearch/trial2aspx? Trialid=chictr-inr-17012642. |
| Drks 2015 | Drks (2015). "Oxytocin-induced enhancement of Social Skills Training in Adolescents with ASD." http://www.who.int/trialsearch/Trial2.aspx?TrialID=DRKS00008952 |
| Euctr 2011 | Euctr ES. Agomelatine efficacy of the drug to improve sleep problems in autistic people. Http://wwwwhoint/trialsearch/trial2aspx? Trialid=euctr2011-003313-42-es 2011. |
| Euctr 2013 | Euctr DE. group-therapy, autism and oxytocin - an investigation with the question Does oxytocin (OT) enhance therapy effects in autism?“. Http://wwwwhoint/trialsearch/trial2aspx? Trialid=euctr2010-024202-34-de 2013. |
| Euctr-000769-35-Be 2018 | Euctr-000769-35-Be (2018). "The use of Oxytocin for Autism Spectrum Disorders: Investigating the effect on behavior and at the level of the brain." |
| Euctr-003313-42-Es 2011 | Euctr-003313-42-Es (2011). "Agomelatine efficacy of the drug to improve sleep problems in autistic people." https://www.clinicaltrialsregister.eu/ctr-search/trial/2011-003313-42/ES |
| Euctr-004378-32-Es 2018 | Euctr-004378-32-Es. A Study to Evaluate Efficacy, and Safety of Balovaptan in Adults with Autism Spectrum Disorder with a 2 Year Open-Label Extension. 2018. |
| Hollander 2019 | Hollander E, Defense U, Pharmaceuticals G, Center MM. Cannabidivarin (CBDV) vs. Placebo in Children With Autism Spectrum Disorder (ASD). 2019. - NCT03202303 |
| Hospital 2014 | Hospital MG, Technology M. A Randomized, Controlled Trial of Intranasal Oxytocin as an Adjunct to Behavioral Therapy for Autism Spectrum Disorder. 2014. - NCT01914939 |
| Hospital 2020 | Hospital, P. C. s. (2020). Effect of a Wide Spectrum Nutritional Supplement on Mitochondrial Function in Children With Autism Spectrum Disorder, https://ClinicalTrials.gov/show/NCT03835117. |
| Huntjens 2020 | Huntjens, A., L. van den Bosch, B. Sizoo, A. Kerkhof, M. J. H. Huibers and M. van der Gaag (2020). "The effect of dialectical behaviour therapy in autism spectrum patients with suicidality and/ or self-destructive behaviour (DIASS): study protocol for a multicentre randomised controlled trial." BMC Psychiatry 20(1): 127. |
| Isrctn 2017a | Isrctn. Therapeutic group for women on the autism spectrum. 2017. ISRCTN13900653 |
| Isrctn 2017b | Isrctn. The Secret Agent Society: Operation Regulation intervention - transdiagnostic trial. 2017. ISRCTN14052478 |
| Isrctn 2017c | Isrctn. ADIE to prevent development of anxiety disorders in autism. 2017. ISRCTN14848787 |
| ISRCTN 2019 | ISRCTN11634810 (2019). A primary school research study to establish whether Social Stories™ can improve social and emotional health in children with autism spectrum disorder. |
| ISRCTN 2020 | ISRCTN70594445 (2020). A study of the use of safety plans to reduce self-harm and suicide for autistic adults. |
| Jprn 2018 | Jprn U. The efficacy of group CBT for children with autism spectrum disorder to reduce anxiety symptoms. Http://wwwwhoint/trialsearch/trial2aspx? Trialid=jprn-umin000033218 2018. |
| JPRN-UMIN 2020 | JPRN-UMIN000040092 (2020). A Preliminary Randomized Controlled Trial Examining the Effects of Rumination Focused Cognitive Behavioral Therapy on Autism Spectrum Disorder and their Brain Basis. |
| Kuroda 2013 | Kuroda M, Kawakubo Y, Kuwabara H, Kano Y, Kamio Y. [Characteristics and adaptive problems of adults with subthreshold ASD in cognitive-behavioral intervention research for emotion regulation]. Seishin Shinkeigaku Zasshi - Psychiatria et Neurologia Japonica 2013;115:623-629. |
| Nct 2014 | Nct (2014). "Examining the Efficacy of the Anxiety Meter for Recognition and Management of Anxiety Signs in Autism Spectrum Disorder." Https://clinicaltrialsgov/show/nct02160691. |
| Nct 2015 | Nct (2015). "Sensory Adapted Dental Environments to Enhance Oral Care for Children." Https://clinicaltrialsgov/show/nct02430051. |
| Nct 2016a | Nct (2016). "CBT for Anxiety Disorder in Children With Co-morbid ASD." Https://clinicaltrialsgov/show/nct02908321. |
| Nct 2016b | Nct (2016). "CBT for Anxiety in Children With Autism." |
| Nct 2016c | Nct (2016). "Treatment of Anxiety in Late Adolescents With Autism." |
| Nct 2016d | Nct (2016). "Trial of Propranolol in Children and Youth With Autism Spectrum Disorder and Predictors of Response." |
| Nct 2017a | Nct (2017). "Intranasal Vasopressin Treatment in Children With Autism." |
| Nct 2017b | Nct (2017). "Specifying and Treating Anxiety in Autism Research." Https://clinicaltrialsgov/show/nct03279471. |
| Nct 2018a | Nct (2018). "The Effect of Aroma on Anxiety Among Children With Autism." |
| Nct 2018b | Nct (2018). "Efficacy and Safety of Bumetanide Oral Liquid Formulation in Children and Adolescents Aged From 7 to Less Than 18 Years Old With Autism Spectrum Disorder." |
| Nct 2018c | Nct (2018). "Evaluating the Efficacy and Effectiveness of SCOPE - RCT." |
| Nct 2018d | Nct (2018). "Internet-delivered Cognitive Behaviour Therapy for Sleep Problems in ASD." |
| Nct 2018e | Nct (2018). "Modifying (Phase I) and Evaluating (Phase II) Virtual Reality Job Interview Training for Youth in Transition." |
| Nct 2018f | Nct (2018). "A Study to Investigate the Efficacy, Safety, and Tolerability of JNJ-42165279 in Adolescent and Adult Participants With Autism Spectrum Disorder." |
| Nct 2019a | Nct (2019). "The Effect Of Virtual Reality Glasses On The Behavior Of Children With Autism Spectrum Disorder." |
| Nct 2019b | Nct (2019). "The Effects of Exercise in Anxiety in Children With ASD." |
| Nl 2019 | Nl (2019). "Tandem study: Theraplay in children with autism spectrum disorder and their parents." |
| Preckel 2016 | Preckel, K., P. Kanske, T. Singer, F. M. Paulus and S. Krach (2016). "Clinical trial of modulatory effects of oxytocin treatment on higher-order social cognition in autism spectrum disorder: a randomized, placebo-controlled, double-blind and crossover trial." BMC Psychiatry 16(1): 329. |
| Russell 2017 | Russell, A., K. Cooper, S. Barton, I. Ensum, D. Gaunt, J. Horwood, B. Ingham, D. Kessler, C. Metcalfe, J. Parr and et al. (2017). "Protocol for a feasibility study and randomised pilot trial of a low-intensity psychological intervention for depression in adults with autism: the Autism Depression Trial (ADEPT)." BMJ open 7(12): e019545. |
| Servier 2018 | Servier, I., a. Adir and Servier (2018). "Efficacy and Safety of Bumetanide Oral Liquid Formulation in Children Aged From 2 to Less Than 7 Years Old With Autism Spectrum Disorder." |
| Sinai 2014 | Sinai I, Foundation AS. A Pilot Treatment Study of Insulin-Like Growth Factor-1 (IGF-1) in Autism Spectrum Disorder. 2014. |
| University 2018a | University S, Health E, Development H. Intranasal Vasopressin Treatment in Children With Autism. 2018. |
| University 2018b | University Y. Translating Neuroprediction Into Precision Medicine Via Brain Priming. 2018. |
| University of California 2017 | University of California D. Specifying and Treating Anxiety in Autism Research. 2017. |

### Appendix 8 References for excluded studies

| Study name | Reference |
| --- | --- |
| Aaronson 2018 | Aaronson, B., A. M. Estes, S. J. Rogers, G. Dawson and R. Bernier (2018). "5.15 The Impact of Comprehensive Behavioral Intervention on Mu Rhythm Attenuation in ASD." Journal of the American Academy of Child and Adolescent Psychiatry 57(10 suppl): s232. |
| Actrn 2008A | Actrn (2008). "Music Therapy in Social and Nonsocial Behaviours of Autism Spectrum Disorder Children." |
| Actrn 2008B | Actrn (2008). "Fluoxetine for the treatment of repetitive behaviours in children and adolescents with autism: A randomised double-blind placebo-controlled trial." |
| Actrn 2009A | Actrn (2009). "Can repetitive transcranial magnetic stimulation (rTMS) alter motor function in autism and Asperger's disorder?". |
| Actrn 2009B | Actrn (2009). "A course of oxytocin nasal spray (OT) to treat social problems in youth with autism spectrum disorders." |
| Actrn 2009C | Actrn (2009). "The effect of Oxytocin (OT) on social cognition and behaviour in youth with Autism Spectrum Disorders (ASD)." |
| Actrn 2010A | Actrn (2010). "Effects of a music therapy protocol on communication of Brazilian children with autistic disorder: a randomized controlled study." |
| Actrn 2010B | Actrn (2010). "Efficacy Of N-Acetyl Cysteine In Autism: A Double-Blind, Placebo-Controlled Randomised Trial." |
| Actrn 2010C | Actrn (2010). "Assessing the efficacy and social validity of a manualised, family-based group Cognitive Behavioural Therapy for adolescents with high-functioning autism and comorbid anxiety disorder.". |
| Actrn 2011A | Actrn (2011). "Can transcranial direct current stimulation (tDCS) improve mirror system activity in autism spectrum disorders?". |
| Actrn 2011B | Actrn (2011). "Effects of Oxytocin on Social Behavior and Repetitive Behavior in Children with Autism." |
| Actrn 2011C | Actrn (2011). "Healthy Eating Learning Program (HELP) Study for Children with Autism Spectrum Disorder." |
| Actrn 2011D | Actrn (2011). "Music, Arousal and Self-Injurious Behaviour: A 3-Stage Mediating Model For Children With Low Functioning Autism (Study 2)." |
| Actrn 2011E | Actrn (2011). "Music, Arousal and Self-Injurious Behaviour: A 3-Stage Mediating Model For Children With Low Functioning Autism - Study 3." |
| Actrn 2012A | Actrn (2012). "Autism early intervention outcomes study." |
| Actrn 2012B | Actrn (2012). "A Randomised Controlled Trial Evaluating a Brief Parenting Program with Children with Autism Spectrum Disorders." |
| Actrn 2013A | Actrn (2013). "A randomized controlled trial of fish-oil supplementation for children with autism spectrum disorder." |
| Actrn 2013B | Actrn (2013). "Evaluation of a randomised control trial of a cognitive behavioural intervention for symptoms of depression in adolescents with autism spectrum disorders." |
| Actrn 2014A | Actrn (2014). "A Within-Subject Randomized Controlled Trial on the Effects of Phenytoin on Social Cognition and Behaviour in Males aged 16 Years and Older with Autism Spectrum Disorders." |
| Actrn 2014B | Actrn (2014). "Levelling the Playing Field: Promoting manageable risk taking in children with disabilities, starting with the school playground." |
| Actrn 2014C | Actrn (2014). "Parent-mediated cognitive behavioural therapy for young children with high-functioning autism spectrum disorder and anxiety: a randomized control trial." |
| Actrn 2015A | Actrn (2015). "Omega-3, vitamin D and Autism in children." |
| Actrn 2015B | Actrn (2015). "A pairwise randomised control trial of a peer-to-peer play-based intervention for children with Autism Spectrum Disorder to improve social play skills and pragmatic language." |
| Actrn 2015C | Actrn (2015). "A Within-Subject Single Dose Trial on the Effects of Bremelanotide on Social Cognition and Behaviour." |
| Actrn 2015D | Actrn (2015). "Does hydrotherapy influence behaviours in children with autism spectrum disorder (ASD)?". |
| Actrn 2016A | Actrn (2016). "A clinical trial of repetitive transcranial magnetic stimulation (rTMS) for improving social relating in adolescents and young adults with autism spectrum disorder (ASD)." |
| Actrn 2016B | Actrn (2016). "A clinical trial to evaluate the safety and efficacy of high-definition transcranial direct current stimulation (HD-tDCS) to improve cognitive flexibility and emotional self-regulation in young people with autism spectrum disorder." |
| Actrn 2016C | Actrn (2016). "Developmental and sequenced 1 to 1 educational intervention in children with autism spectrum disorder (ASD): a randomized controlled trial." |
| Actrn 2016D | Actrn (2016). "The effects of chiropractic care on sensory integration in children with autism." |
| Actrn 2016E | Actrn (2016). "Improving independence in driving for people with Autism Spectrum Disorder." |
| Actrn 2016F | Actrn (2016). "Lactobacillus plantarum PS128 on behavior activity of children with autism." |
| Actrn 2016G | Actrn (2016). "The Secret Agent Society: Whole of classroom program , investigating the efficacy of a social skill and emotion regulation focused program for children with autism spectrum disorders, unspecified social and emotional difficulties and typically developing children within a classroom context.". |
| Actrn 2017A | Actrn (2017). "A Course of Oxytocin to Improve Social Communication in Young Children with Autism." |
| Actrn 2017B | Actrn (2017). "Decreasing Defensive Responses: Investigating the Safe &amp; Sound Protocol (SSP) to improve emotional regulation, communication, and social engagement with children with Autism.". |
| Actrn 2017C | Actrn (2017). "Evaluating the effectiveness of mind-body skills training based on yoga techniques in children on the Autism Spectrum." |
| ACTRN 2017D | ACTRN12617000538347 (2017). A school-based intervention for supporting the social, communication and friendship skills of children with autism and their classmates. |
| Actrn 2017E | Actrn (2017). "Building Bridges Triple P: A new parenting program for parents of teens with a developmental disorder aimed at offering parents strategies to deal with emotional and behavioural problems in their children." |
| Actrn 2017F | Actrn (2017). "KONTAKT a Social Skills Group Training Intervention for Teenagers with Autism Spectrum Disorders in Australia." |
| Actrn 2018A | Actrn (2018). "An evaluation of the Sleeping Sound program on children's sleep difficulties in children with Autism Spectrum Disorder and Intellectual Disability." |
| Actrn 2018B | Actrn (2018). "Oxytocin in Preschoolers with Autism receiving Social Learning Therapy." |
| Actrn 2018C | Actrn (2018). "Evaluation of the Early Start Denver Model (ESDM) in an Inclusive Preschool Setting." |
| Actrn 2018D | Actrn (2018). "Outcome of Early Start Denver Model Direct Therapy in addition to Parent Training for Young Children with Autism." |
| Actrn 2019A | Actrn (2019). "The Developmental and Social Benefits of a Classical/Contemporary AllPlay Dance Program for Children with Autism Spectrum Disorder." http://www.who.int/trialsearch/Trial2.aspx?TrialID=ACTRN12619000671167. |
| Actrn 2019B | Actrn (2019). "Prebiotic supplement use, the gut microbiome and behaviour change in children with autism spectrum disorder (ASD)." http://www.who.int/trialsearch/Trial2.aspx?TrialID=ACTRN12619000615189. |
| Actrn 2019C | Actrn (2019). "Social Groups for Australian Children on the Autism Spectrum." http://www.who.int/trialsearch/Trial2.aspx?TrialID=ACTRN12619000994189. |
| ACTRN 2019D | ACTRN12619000584134 (2019). Use and Effectiveness of the Smiling Mind Smartphone Application on Wellbeing of Children and Adults with Autism Spectrum Disorder and Their Caregivers. |
| ACTRN 2019E | ACTRN12619000615189 (2019). Prebiotic supplement use, the gut microbiome and behaviour change in children with autism spectrum disorder (ASD). |
| ACTRN 2019F | ACTRN12619000671167 (2019). The Developmental and Social Benefits of a Classical/Contemporary AllPlay Dance Program for Children with Autism Spectrum Disorder. |
| ACTRN 2019G | ACTRN12619000994189 (2019). Social Groups for Australian Children on the Autism Spectrum. |
| ACTRN 2019H | ACTRN12619001267145 (2019). Transition program for adolescents and young adults with autism in Australia. |
| ACTRN 2020 | ACTRN12620000139976 (2020). Simulation training to manage challenging behaviours in children with autism. |
| Adams 2009 | Adams, J. B., M. Baral, E. Geis, J. Mitchell, J. Ingram, A. Hensley, I. Zappia, S. Newmark, E. Gehn, R. A. Rubin, K. Mitchell, J. Bradstreet and J. El-Dahr (2009). "Safety and efficacy of oral DMSA therapy for children with autism spectrum disorders: Part A - Medical results." BMC Clinical Pharmacology 9(16). |
| Adams 2011 | Adams, J. B., T. Audhya, S. McDonough-Means, R. A. Rubin, D. Quig, E. Geis, E. Gehn, M. Loresto, J. Mitchell, S. Atwood, S. Barnhouse and W. Lee (2011). "Effect of a vitamin/mineral supplement on children and adults with autism." BMC Pediatrics 11: 111. |
| Adams 2012 | Adams, C., E. Lockton, J. Freed, J. Gaile, G. Earl, K. McBean, M. Nash, J. Green, A. Vail and J. Law (2012). "The Social Communication Intervention Project: a randomized controlled trial of the effectiveness of speech and language therapy for school-age children who have pragmatic and social communication problems with or without autism spectrum disorder." International journal of language and communication disorders / Royal College of Speech and Language Therapists 47(3): 233-244. |
| Adams 2018 | Adams, J. B., T. Audhya, E. Geis, E. Gehn, V. Fimbres, E. L. Pollard, J. Mitchell, J. Ingram, R. Hellmers, D. Laake, J. S. Matthews, K. F. Li, J. C. Naviaux, R. K. Naviaux, R. L. Adams, D. M. Coleman and D. W. Quig (2018). "Comprehensive Nutritional and Dietary Intervention for Autism Spectrum Disorder-A Randomized, Controlled 12-Month Trial." Nutrients 10(3). |
| Adibsereshki 2015 | Adibsereshki, N., A. Nesayan, R. Asadi Gandomani and M. Karimlou (2015). "The effectiveness of theory of mind training on the social skills of children with high functioning autism spectrum disorders." Iranian Journal of Child Neurology 9(3): 40-49. |
| Afsharnejad 2019 | Afsharnejad, B., M. Falkmer, M. H. Black, T. Alach, F. Lenhard, A. Fridell, C. Coco, K. Milne, N. T. M. Chen, S. Bolte and et al. (2019). "KONTAKTÂ© for Australian adolescents on the autism spectrum: protocol of a randomized control trial." Trials 20(1). |
| Aghababaei 2020 | Sara Aghababaei, Maryam Taghavi The effectiveness of mindfulness-based cognitive therapy training on the psychological well-being of mothers of children with autism and childhood symptoms. Shenakht Journal of Psychology & Psychiatry 2020;6(6): 88-100. |
| Ajram 2015A | Ajram, L. (2015). "Pharmacological modulation of excitatory/ inhibitory balance in autism spectrum disorder." European Neuropsychopharmacology 2): s127. |
| Ajram 2015B | Ajram, L., J. Horder, M. A. Mendez, L. Brennan, A. Galanopoulos, J. Zinkstok, D. Lythgoe, S. Williams, D. Murphy and G. McAlonan (2015). "Pharmacological modulation of excitatory/inhibitory balance in autism spectrum disorder." European Neuropsychopharmacology 25(SUPPL): s61. |
| Akabogu 2019 | Akabogu, J., A. Nnamani, M. S. Otu, A. C. Uloh-Bethels, E. Ukoha, O. M. Iyekekpolor, J. C. Omile, M. N. Obiezu, A. E. Dike and C. V. Ike (2019). "Effects of a language education intervention on social participation among emerging adults with autism." Journal of International Medical Research 48(1). |
| Akhondzadeh 2008 | Akhondzadeh, S., H. Tajdar, M. R. Mohammadi, M. Mohammadi, G. H. Nouroozinejad, O. L. Shabstari and H. A. Ghelichnia (2008). "A double-blind placebo controlled trial of piracetam added to risperidone in patients with autistic disorder." Child Psychiatry and Human Development 39(3): 237-245. |
| Alaerts 2019 | Alaerts, K. and S. Bernaerts (2019). "P.509 Continual oxytocin treatment induces long-lasting adaptations within amygdala circuitry in autism: a randomized placebo-controlled trial." European Neuropsychopharmacology 29 (Supplement 6): S358-S359. |
| Alaerts 2020A | Alaerts, K., S. Bernaerts, J. Prinsen, C. Dillen, J. Steyaert and N. Wenderoth (2020). "Oxytocin induces long-lasting adaptations within amygdala circuitry in autism: a treatment-mechanism study with randomized placebo-controlled design." Neuropsychopharmacology 45(7): 1141-1149. |
| Alaerts 2020B | Alaerts, K., S. Bernaerts, B. Vanaudenaerde, N. Daniels and N. Wenderoth (2019). "Amygdala-Hippocampal Connectivity Is Associated With Endogenous Levels of Oxytocin and Can Be Altered by Exogenously Administered Oxytocin in Adults With Autism." Biological Psychiatry: Cognitive Neuroscience and Neuroimaging 4(7): 655-663. |
| Albaum 2019 | Albaum, C., F. Roudbarani, P. Tablon Modica and J. A. Weiss (2019). "Therapist behaviours predicting therapeutic alliance in cognitive behaviour therapy for children with autism." Journal of Intellectual Disability Research 63 (7): 664. |
| Albaum 2020 | Albaum, C., P. Tablon, F. Roudbarani and J. A. Weiss (2020). "Predictors and outcomes associated with therapeutic alliance in cognitive behaviour therapy for children with autism." Autism 24(1): 211-220. |
| Aldred 2004 | Aldred, C., J. Green and C. Adams (2004). "A new social communication intervention for children with autism: Pilot randomised controlled treatment study suggesting effectiveness." Journal of Child Psychology and Psychiatry and Allied Disciplines 45(8): 1420-1430. |
| Alliance 2006 | Alliance, C. H. and B. M. Squibb (2006). "An Open-Label Trial of Aripiprazole in Autism Spectrum Disorders.": https://clinicaltrials.gov/ct2/show/NCT00308074 |
| Alolaby 2020 | Alolaby, R. R., P. Jiraanont, B. Durbin-Johnson, M. Jasoliya, H. T. Tang, R. Hagerman and F. Tassone (2020). "Molecular Biomarkers Predictive of Sertraline Treatment Response in Young Children With Autism Spectrum Disorder." Frontiers in Genetics 11 (no pagination). |
| Alquraini 2019 | Alquraini, T., A. Al-Odaib, H. Al-Dhalaan, H. Merza and G. Mahoney (2019). "Relationship-based Intervention with Young Children with Autism in Saudi Arabia: impediments and Consequences of Parenting Stress and Depression." International journal of disability, development & education 66(3): 233‐248. |
| Althaus 2015 | Althaus, M., Y. Groen, A. Wijers, H. Noltes, O. Tucha and P. Hoekstra (2015). "Oxytocin enhances orienting to social information in a selective group of high-functioning male adults with autism spectrum disorder." Neuropsychologia 79(part a): 53-69. |
| Althaus 2016 | Althaus, M., Y. Groen, A. W. A, H. Noltes, O. Tucha, F. C. Sweep, F. Calcagnoli and P. J. Hoekstra (2016). "Do blood plasma levels of oxytocin moderate the effect of nasally administered oxytocin on social orienting in high-functioning male adults with autism spectrum disorder?" Psychopharmacology 233(14): 2737?2751. |
| Alvares 2019 | Alvares, G. A., N. T. M. Chen, L. Notebaert, J. Granich, C. Mitchell and A. J. O. Whitehouse (2019). "Brief social attention bias modification for children with autism spectrum disorder." Autism research : Official Journal of the International Society for Autism Research 12(3): 527-535. |
| Aman 2005 | Aman, M. G., L. Arnold, C. J. McDougle, B. Vitiello, L. Scahill, M. Davies, J. T. McCracken, E. Tierney, P. L. Nash, D. J. Posey, S. Chuang, A. Martin, B. Shah, N. M. Gonzalez, N. B. Swiezy, L. Ritz, K. Koenig, J. McGough, J. K. Ghuman and R. L. Lindsay (2005). "Acute and long-term safety and tolerability of risperidone in children with autism." Journal of Child and Adolescent Psychopharmacology 15(6): 869-884. |
| Aman 2009A | Aman, M. G., C. J. McDougle, L. Scahill, B. Handen, L. E. Arnold, C. Johnson, K. A. Stigler, K. Bearss, E. Butter, N. B. Swiezy, D. D. Sukhodolsky, Y. Ramadan, S. L. Pozdol, R. Nikolov, L. Lecavalier, A. E. Kohn, K. Koenig, J. A. Hollway, P. Korzekwa and A. Gavaletz (2009). "Medication and parent training in children with pervasive developmental disorders and serious behavior problems: results from a randomized clinical trial." Journal of the American Academy of Child & Adolescent Psychiatry 48(12): 1143-1154. |
| Aman 2009B | Aman, M. G., C. J. McDougle, L. Scahill, B. Handen, L. E. Arnold, C. Johnson, K. A. Stigler, K. Bearss, E. Butter, N. B. Swiezy, D. D. Sukhodolsky, Y. Ramadan, S. L. Pozdol, R. Nikolov, L. Lecavalier, A. E. Kohn, K. Koenig, J. A. Hollway, P. Korzekwa, A. Gavaletz, J. A. Mulick, K. L. Hall, J. Dziura, L. Ritz, S. Trollinger, S. Yu, B. Vitiello and A. Wagner (2009). "Medication and Parent Training in Children With Pervasive Developmental Disorders and Serious Behavior Problems: Results From a Randomized Clinical Trial." Journal of the American Academy of Child and Adolescent Psychiatry 48(12): 1143-1154. |
| Aman 2010 | Aman, M. G., W. Kasper, G. Manos, S. Mathew, R. Marcus, R. Owen and R. Mankoski (2010). "Line-item analysis of the aberrant behavior checklist: Results from two studies of aripiprazole in the treatment of irritability associated with autistic disorder." Journal of Child and Adolescent Psychopharmacology 20(5): 415-422. |
| Aman 2015 | Aman, M., M. Rettiganti, H. N. Nagaraja, J. A. Hollway, J. McCracken, C. J. McDougle, E. Tierney, L. Scahill, L. E. Arnold, J. Hellings, D. J. Posey, N. B. Swiezy, J. Ghuman, M. Grados, B. Shah and B. Vitiello (2015). "Tolerability, safety, and benefits of risperidone in children and adolescents with autism: 21-month follow-up after 8-week placebo-controlled trial." Journal of Child and Adolescent Psychopharmacology 25(6): 482-493. |
| Aman 2017 | Aman, M. G., R. L. Findling, A. Y. Hardan, R. L. Hendren, R. D. Melmed, O. Kehinde-Nelson, H. A. Hsu, J. M. Trugman, R. H. Palmer, S. M. Graham, A. T. Gage, J. L. Perhach and E. Katz (2017). "Safety and efficacy of memantine in children with autism: Randomized, placebo-controlled study and open-label extension." Journal of Child and Adolescent Psychopharmacology 27(5): 403-412. |
| Aman 2018 | Aman, M. G., J. A. Hollway, J. Veenstra-Vanderweele, B. L. Handen, K. B. Sanders, J. Chan, E. Macklin, L. E. Arnold, T. Wong, C. Newsom, R. Hastie Adams, S. Marler, N. Peleg and E. A. Anagnostou (2018). "Effects of Metformin on Spatial and Verbal Memory in Children with ASD and Overweight Associated with Atypical Antipsychotic Use." Journal of Child and Adolescent Psychopharmacology 28(4): 266-273. |
| Amatachaya 2015 | Amatachaya, A., M. P. Jensen, N. Patjanasoontorn, N. Auvichayapat, C. Suphakunpinyo, S. Janjarasjitt, N. Ngernyam, B. Aree-Uea and P. Auvichayapat (2015). "The short-term effects of transcranial direct current stimulation on electroencephalography in children with autism: A randomized crossover controlled trial." Behavioural Neurology 2015(928631). |
| Ameis 2017A | Ameis, S., L. Yoganathan, C. Hawco, A. Voineskos, R. Lyon, J. Daskalakis, D. Blumberger, P. Croarkin and P. Szatmari (2017). "Findings from baseline neuroimaging data in a double-blind randomized controlled trial of RTMS for executive function deficits for ASD." Neuropsychopharmacology 43(suppl 1): s499. |
| Ameis 2017B | Ameis, S. H., Z. J. Daskalakis, D. M. Blumberger, P. Desarkar, I. Drmic, D. J. Mabbott, M. C. Lai, P. E. Croarkin and P. Szatmari (2017). "Repetitive Transcranial Magnetic Stimulation for the Treatment of Executive Function Deficits in Autism Spectrum Disorder: Clinical Trial Approach." Journal of Child and Adolescent Psychopharmacology 27(5): 413-421. |
| Ameis 2018 | Ameis, S. H. (2018). "Feasibility and Tolerability of Repetitive Transcranial Magnetic Stimulation (RTMS) Treatment for Executive-Function Deficits in ASD: Results From a Randomized, Controlled Trial." Journal of the American Academy of Child and Adolescent Psychiatry 57(10 suppl): s318. |
| Ameis 2020 | Ameis, S. H., D. M. Blumberger, P. E. Croarkin, D. J. Mabbott, M. C. Lai, P. Desarkar, P. Szatmari and Z. J. Daskalakis (2020). "Treatment of Executive Function Deficits in autism spectrum disorder with repetitive transcranial magnetic stimulation: A double-blind, sham-controlled, pilot trial." Brain Stimulation 13(3): 539-547. |
| Amminger 2007A | Amminger, G. P., G. E. Berger, M. R. Schafer, C. Klier, M. H. Friedrich and M. Feucht (2007). "Omega-3 Fatty Acids Supplementation in Children with Autism: A Double-blind Randomized, Placebo-controlled Pilot Study." Biological Psychiatry 61(4): 551-553. |
| Amminger 2007B | Amminger, G. P., G. E. Berger, M. R. Schäfer, C. Klier, M. H. Friedrich and M. Feucht (2007). "Omega-3 fatty acids supplementation in children with autism: a double-blind randomized, placebo-controlled pilot study." Biological psychiatry 61(4): 551‐553. |
| Anagnostou 2006A | Anagnostou, E., K. Esposito, L. Soorya, W. Chaplin, S. Wasserman and E. Hollander (2006). "Divalproex versus placebo for the prevention of irritability associated with fluoxetine treatment in autism spectrum disorder [11]." Journal of Clinical Psychopharmacology 26(4): 444-446. |
| Anagnostou 2006B | Anagnostou, E., K. Esposito, L. Soorya, W. Chaplin, S. Wasserman and E. Hollander (2006). "Divalproex versus placebo for the prevention of irritability associated with fluoxetine treatment in autism spectrum disorder." Journal of Clinical Psychopharmacology 26(4): 444?446. |
| Anagnostou 2006C | Anagnostou, E., I. Sinai and E. M. D. Anagnostou (2006). "Intranasal Oxytocin in the Treatment of Autism." https://clinicaltrials.gov/ct2/show/NCT00490802 |
| Anagnostou 2010A | Anagnostou, E., H. B. K. R. Hospital, T. Children and E. M. D. Anagnostou (2010). "Omega-3 Fatty Acids For Treatment Of Young Children With Autism (OMG)." https://clinicaltrials.gov/ct2/show/NCT01248728 |
| Anagnostou 2010B | Anagnostou, E., H. B. K. R. Hospital, T. Children, U. Chicago and E. M. D. Anagnostou (2010). "Intranasal Oxytocin for the Treatment of Children and Adolescents With ASD (OXY)." https://clinicaltrials.gov/ct2/show/NCT01256060 |
| Anagnostou 2011 | Anagnostou, E., I. Sinai, R. U. M. Center, N. Hospital and E. M. D. Anagnostou (2011). "A Multi-site Double-blind Placebo-controlled Trial of Memantine Versus Placebo in Children With Autism (MEM)." https://clinicaltrials.gov/ct2/show/NCT01372449 |
| Anagnostou 2012 | Anagnostou, E., L. Soorya, W. Chaplin, J. Bartz, D. Halpern, S. Wasserman, A. T. Wang, L. Pepa, N. Tanel, A. Kushki and et al. (2012). "Intranasal oxytocin versus placebo in the treatment of adults with autism spectrum disorders: a randomized controlled trial." Molecular Autism 3(1). |
| Anagnostou 2013A | Anagnostou, E., H. B. K. R. Hospital, M. University, T. Children, C. University of Western Ontario, T. St. Michael's Hospital, U. Toronto and E. M. D. Anagnostou (2013). "A Pilot Study of Riluzole Versus Placebo in the Treatment of Children and Adolescents With ASD." https://clinicaltrials.gov/ct2/show/NCT01661855 |
| Anagnostou 2013B | Anagnostou, E., U. Defense and E. M. D. Anagnostou (2013). "Intranasal Oxytocin for the Treatment of Children and Adolescents With Autism Spectrum Disorders (ASD)." https://clinicaltrials.gov/ct2/show/NCT01908205 |
| Anagnostou 2013C | Anagnostou, E., H. B. K. R. Hospital and E. M. D. Anagnostou (2013). "Dose Finding Study of Pioglitazone in Children With Autism Spectrum Disorders (ASD) (PIO)." https://clinicaltrials.gov/ct2/show/NCT01205282 |
| Anagnostou 2013D | Anagnostou, E., M. G. Hospital, V. University, U. Pittsburgh, N. Hospital, O. S. University and E. M. D. Anagnostou (2013). "Treatment of Overweight Induced by Antipsychotic Medication in Young People With Autism Spectrum Disorders (ASD)." https://clinicaltrials.gov/ct2/show/NCT01825798 |
| Anagnostou 2014 | Anagnostou, E., H. B. K. R. Hospital, M. University, T. St. Michael's Hospital and E. M. D. Anagnostou (2014). "INtranasal Oxytocin for the Treatment of Autism Spectrum Disorders." https://clinicaltrials.gov/ct2/show/NCT01788072 |
| Anagnostou 2016A | Anagnostou, E., M. G. Aman, B. L. Handen, K. B. Sanders, A. Shui, J. A. Hollway, J. Brian, L. E. Arnold, L. Capano, J. A. Hellings, E. Butter, D. Mankad, R. Tumuluru, J. Kettel, C. R. Newsom, S. Hadjiyannakis, N. Peleg, D. Odrobina, S. McAuliffe-Bellin, P. Zakroysky, S. Marler, A. Wagner, T. Wong, E. A. Macklin and J. Veenstra-Vander Weele (2016). "Metformin for treatment of overweight induced by atypical antipsychotic medication in young people with autism spectrum disorder: A randomized clinical trial." JAMA Psychiatry 73(9): 928-937. |
| Anagnostou 2016B | Anagnostou, E., H. B. K. R. Hospital, M. University, C. University of Western Ontario, T. St. Michael's Hospital, U. Toronto and E. M. D. Anagnostou (2016). "Tideglusib vs. Placebo in the Treatment of Adolescents With Autism Spectrum Disorders." |
| Anagnostou 2018 | Anagnostou, E., T. A. Bennett, K. Thorpe and R. Nicolson (2018). "5.16 A Phase 2 Randomized, Placebo-Controlled Trial of Tideglusib, an Orally Administered GSK-3 Beta Inhibitor, in the Treatment of Adolescents With ASD." Journal of the American Academy of Child and Adolescent Psychiatry 57(10 suppl): s232. |
| Anderson 1989 | Anderson, L. T., M. Campbell, P. Adams, A. M. Small, R. Perry and J. Shell (1989). "The effects of haloperidol on discrimination learning and behavioral symptoms in autistic children." Journal of Autism and Developmental Disorders 19(2): 227-239. |
| Anderson 2009 | Anderson, K. S. (2009). "Social skills training for children with autism utilizing peers as behavioral models." Dissertation Abstracts International: Section B: The Sciences and Engineering 70(5-b): 3159. |
| Anninos 2016 | Anninos, P., A. Chatzimichael, A. Adamopoulos, A. Kotini and N. Tsagas (2016). "A combined study of MEG and pico-Tesla TMS on children with autism disorder." Journal of Integrative Neuroscience 15(4): 497?513. |
| Anonymous 1980 | Anonymous (1980). "A multi centered double blind trial of pimozide (Orap), haloperidol and placebo for abnormal behavior in children using crossover design." Rinsho hyoka /clinical evaluation 8(3): 629?673. |
| Anonymous 2002 | Anonymous (2002). "Children with autism may benefit from risperidone." Pharmaceutical Journal 269(7210): 184. |
| Anonymous 2005A | Anonymous (2005). "Randomized, controlled, crossover trial of methylphenidate in pervasive developmental disorders with hyperactivity." Archives of General Psychiatry 62(11): 1266?1274. |
| Anonymous 2005B | Anonymous (2005). "Risperidone treatment of autistic disorder: longer-term benefits and blinded discontinuation after 6 months." American Journal of Psychiatry 162(7): 1361?1369. |
| Anonymous 2009 | Anonymous (2009). "Citalopram ineffective for reducing repetitive behavior in autism spectrum disorders." Journal of the National Medical Association 101(9): 976. |
| Anonymous 2011 | Anonymous (2011). "Efficacy of agomelatine on sleep disturbance in Autism Spectrum Disorder (ASD)." EU clinical trials register [wwwclinicaltrialsregistereu]. |
| Anonymous 2016A | Anonymous (2016). "Fostering Social Cognition through an Imitation- and Synchronization-Based Dance/Movement Intervention in Adults with Autism Spectrum Disorder: a Controlled Proof-of-Concept Study." Psychotherapy and psychosomatics 85 (1) (pp 27-35), 2016 Date of publication: 01 jan 2016". |
| Anonymous 2016B | Anonymous (2016). "Investigating mobile emotional learning for children with autistic spectrum disorders." International journal of developmental disabilities (pp 1-10), 2016 Date of publication: 06 aug 2016". |
| Anonymous 2017 | Anonymous (2017). "Evaluating the Effectiveness of a School-Based Cognitive Behavioural Therapy Intervention for Anxiety in Adolescents Diagnosed with Autism Spectrum Disorder." Journal of Autism and Developmental Disorders 47(12): 3896?3908. |
| Anonymous1 | (2002). "Children with autism may benefit from risperidone." Pharmaceutical journal 269(7210): 184‐. |
| Anonymous10 | (2020). "Improved performance on the empathy quotient in adult autism spectrum disorder after tDCS." Clinical neurophysiology 131(4): e80‐e81. |
| Anonymous2 | (2019). "The effect of music therapy on autistic symptoms and quality of life in children with autism spectrum disorder." Anadolu psikiyatri dergisi 20(4): 436‐441. |
| Anonymous3 | (2019). "Effectiveness of play-based empathy training on social skills in students with autistic spectrum Disorders." Archives of psychiatry and psychotherapy 21(3): 71‐76. |
| Anonymous4 | (2019). "Erratum to: the feasibility of low-intensity psychological therapy for depression co-occurring with autism in adults: the Autism Depression Trial (ADEPT) â€“ a pilot randomised controlled trial (Autism, (2019), 10.1177/1362361319889272)." Autism. |
| Anonymous5 | (2019). "Group-Based Social Skills Training with Play for Children on the Autism Spectrum." Journal of autism and developmental disorders 49(6): 2231‐2242. |
| Anonymous6 | (2019). "Music therapy as a therapeutic tool in improving the social skills of autistic children." Egyptian journal of neurology, psychiatry and neurosurgery 55(1). |
| Anonymous7 | (2019). "Outcomes of children receiving Group-Early Start Denver Model in an inclusive versus autism-specific setting: A pilot randomized controlled trial." Autism: The International Journal of Research & Practice 23(5): 1165-1175. |
| Anonymous8 | (2020). "Effects of mini-basketball training program on executive functions and core symptoms among preschool children with autism spectrum disorders." Brain sciences 10(5). |
| Anonymous9 | (2020). Erratum...Russell A, Gaunt DM, Cooper K, et al. The feasibility of low-intensity psychological therapy for depression co-occurring with autism in adults: The Autism Depression Trial (ADEPT) – a pilot Randomised controlled trial. Autism: The International Journal of Research & Practice (AUTISM), 2020; 24(6): 1360-1372. Thousand Oaks, California, Sage Publications Inc. 24: 1583-1583. |
| Antonini 2017 | Antonini, M., S. Serret, F. Maria, J. Bourgeois, E. Fontas, F. Askenazy, M. Benoit and M. Fouchet (2017). "Evaluation of the effectiveness of the serious game JeStiMulE for the improvement of social cognition in adults with autistic spectrum disorders." European Neuropsychopharmacology 27(suppl 4): s1105-s1106. |
| Aoki 2014 | Aoki, Y., N. Yahata, T. Watanabe, Y. Takano, Y. Kawakubo, H. Kuwabara, N. Iwashiro, T. Natsubori, H. Inoue, M. Suga, H. Takao, H. Sasaki, W. Gonoi, A. Kunimatsu, K. Kasai and H. Yamasue (2014). "Oxytocin improves behavioural and neural deficits in inferring others' social emotions in autism." Brain 137(pt 11): 3073-3086. |
| Arabi 2019 | Arabi, M., A. S. Kakhki, M. Sohrabi, S. S. Kouhbanani and M. J. Nooghabi (2019). "Is visuomotor training an effective intervention for children with autism spectrum disorders?" Neuropsychiatric Disease and Treatment 15: 3089-3102. |
| Arkansas 2012 | Arkansas, U. and A. Institute (2012). "A Folinic Acid Intervention for Autism Spectrum Disorders." https://clinicaltrials.gov/ct2/show/NCT01602016 |
| Arnold 2003 | Arnold, L., B. Vitiello, C. McDougle, L. Scahill, B. Shah, N. M. Gonzalez, S. Chuang, M. Davies, J. Holloway, M. G. Aman, P. Cronin, K. Koenig, A. E. Kohn, D. J. McMahon and E. Tierney (2003). "Parent-Defined Target Symptoms Respond to Risperidone in RUPP Autism Study: Customer Approach to Clinical Trials." Journal of the American Academy of Child & Adolescent Psychiatry 42(12): 1443-1450. |
| Arnold 2006 | Arnold, L. E., M. G. Aman, A. M. Cook, A. N. Witwer, K. L. Hall, S. Thompson and Y. Ramadan (2006). "Atomoxetine for hyperactivity in autism spectrum disorders: Placebo-controlled crossover pilot trial." Journal of the American Academy of Child and Adolescent Psychiatry 45(10): 1196-1205. |
| Arnold 2010 | Arnold, L., C. Farmer, H. C. Kraemer, M. Davies, A. Witwer, S. Chuang, R. DiSilvestro, C. J. McDougle, J. McCracken, B. Vitiello, M. G. Aman, L. Scahill, D. J. Posey and N. B. Swiezy (2010). "Moderators, mediators, and other predictors of risperidone response in children with autistic disorder and irritability." Journal of Child and Adolescent Psychopharmacology 20(2): 83-93. |
| Arnold 2012 | Arnold, L. E., M. G. Aman, J. Hollway, E. Hurt, B. Bates, X. Li, C. Farmer, R. Anand, S. Thompson, Y. Ramadan and C. Williams (2012). "Placebo-controlled pilot trial of mecamylamine for treatment of autism spectrum disorders." Journal of Child and Adolescent Psychopharmacology 22(3): 198-205. |
| Arnold 2018 | Arnold, L. E. (2018). "Placebo-Controlled Pilot Data for Three Complementary/Alternative Treatments in Autism." Journal of the American Academy of Child and Adolescent Psychiatry 57(10 suppl): s117. |
| Arnold 2019 | Arnold, L. E., R. A. Luna, K. Williams, J. Chan, R. A. Parker, Q. Wu, J. A. Hollway, A. Jeffs, F. Lu, D. L. Coury and et al. (2019). "Probiotics for Gastrointestinal Symptoms and Quality of Life in Autism: a Placebo-Controlled Pilot Trial." Journal of child and adolescent psychopharmacology 29(9): 659‐669. |
| As 2015 | As, O., O. U. Hospital and U. Oslo (2015). "Effects of Intranasal Administration of a Single Dose of Oxytocin Using a Novel Device in Adults With Autism Spectrum Disorder." https://clinicaltrials.gov/ct2/show/NCT02414503 |
| Asadabadi 2013 | Asadabadi, M., M. R. Mohammadi, A. Ghanizadeh, A. Modabbernia, M. Ashrafi, E. Hassanzadeh, S. Forghani and S. Akhondzadeh (2013). "Celecoxib as adjunctive treatment to risperidone in children with autistic disorder: a randomized, double-blind, placebo-controlled trial." Psychopharmacology 225(1): 51-59. |
| Auyeung 2015 | Auyeung, B., M. V. Lombardo, M. Heinrichs, B. Chakrabarti, A. Sule, J. B. Deakin, R. A. I. Bethlehem, L. Dickens, N. Mooney, J. A. N. Sipple, P. Thiemann and S. Baron-Cohen (2015). "Oxytocin increases eye contact during a real-time, naturalistic social interaction in males with and without autism." Translational Psychiatry 5(2 e507). |
| Awad 1996 | Awad, G. A. (1996). "The use of selective serotonin reuptake inhibitors in young children with pervasive developmental disorders: some clinical observations." Canadian Journal of Psychiatry - Revue Canadienne de Psychiatrie 41(6): 361-366. |
| Ayatollahi 2020 | Ayatollahi, A., S. Bagheri, A. Ashraf-Ganjouei, K. Moradi, M. R. Mohammadi and S. Akhondzadeh (2020). "Does Pregnenolone Adjunct to Risperidone Ameliorate Irritable Behavior in Adolescents With Autism Spectrum Disorder: A Randomized, Double-Blind, Placebo-Controlled Clinical Trial?" Clinical Neuropharmacology 15: 15. |
| Bader 2006 | Bader, R. (2006). "Using social stories to increase emotion recognition and labeling in school-age children with autism." Dissertation Abstracts International: Section B: The Sciences and Engineering 67(3-b): 1692. |
| Bagaiolo 2017 | Bagaiolo, L. F., J. D. J. Mari, D. Bordini, T. C. Ribeiro, M. C. C. Martone, S. C. Caetano, D. Brunoni, H. Brentani and C. S. Paula (2017). "Procedures and compliance of a video modeling applied behavior analysis intervention for Brazilian parents of children with autism spectrum disorders." Autism 21(5): 603-610. |
| Baghdadli 2010 | Baghdadli, A., J. Brisot-Dubois, M. C. Picot and C. Michelon (2010). "Comparison of the effect of two prosocial interventions about the evolution of recognition of facial expression abilities and social cognition of children with an Asperger syndrome or high functioning autism." Neuropsychiatrie de l'Enfance et de l'Adolescence 58(8): 456-462. |
| Ballester 2015 | Ballester, P., M. J. Martinez, A. Javaloyes, L. Hernandez and A. M. Peiro (2015). "Agomelatine effectiveness in sleep disturbances in autism spectrum disorder." Clinical therapeutics 37(8 suppl 1): e132?e133. |
| Ballester 2019 | Ballester, P., M. J. Martinez, M. D. M. Inda, A. Javaloyes, A. L. Richdale, J. Muriel, C. Belda, N. Toral, D. Morales, E. Fernandez and A. M. Peiro (2019). "Evaluation of agomelatine for the treatment of sleep problems in adults with autism spectrum disorder and co-morbid intellectual disability." Journal of Psychopharmacology 33(11): 1395-1406. |
| Bangabandhu 2019 | Bangabandhu Sheikh Mujib Medical University, D., Bangladesh (2019). Vitamin B6 and Magnesium- A Clinical Trial on ASD Patients, https://ClinicalTrials.gov/show/NCT03963479. |
| Barthelemy 1989 | Barthelemy, C., N. Bruneau, J. Jouve, J. Martineau, J. Muh and G. Lelord (1989). "Urinary dopamine metabolites as indicators of the responsiveness to fenfluramine treatment in children with autistic behavior." Journal of Autism and Developmental Disorders 19(2): 241-254. |
| Bauminger-Zviely 2020 | Bauminger-Zviely, N., D. Eytan, S. Hoshmand and O. Rajwan Ben-Shlomo (2020). "Preschool Peer Social Intervention (PPSI) to Enhance Social Play, Interaction, and Conversation: Study Outcomes." Journal of Autism and Developmental Disorders 50(3): 844-863. |
| Bearss 2015 | Bearss, K., C. Johnson, T. Smith, L. Lecavalier, N. Swiezy, M. Aman, D. B. McAdam, E. Butter, C. Stillitano, N. Minshawi, D. G. Sukhodolsky, D. W. Mruzek, K. Turner, T. Neal, V. Hallett, J. A. Mulick, B. Green, B. Handen, Y. H. Deng, J. Dziura and L. Scahill (2015). "Effect of Parent Training vs Parent Education on Behavioral Problems in Children With Autism Spectrum Disorder A Randomized Clinical Trial." Jama-JAMA 313(15): 1524-1533. |
| Beaumont 2008A | Beaumont, R. and K. Sofronoff (2008). "A multi-component social skills intervention for children with Asperger syndrome: the Junior Detective Training Program." Journal of Child Psychology and Psychiatry and Allied Disciplines 49(7): 743-753. |
| Beaumont 2008B | Beaumont, R. and K. Sofronoff (2008). "A multi-component social skills intervention for children with Asperger syndrome: The Junior Detective Training Program : Errata." Journal of Child Psychology and Psychiatry 49(8): 895. |
| Beaumont 2018A | Beaumont, R. B. (2018). "RANDOMIZED CONTROLLED TRIAL OF A SERIOUS GAMING INTERVENTION FOR CHILDREN ON THE AUTISM SPECTRUM: EFFECTS ON EMOTION REGULATION AND SOCIAL SKILLS." Journal of the American Academy of Child and Adolescent Psychiatry 57(10): S35-S36. |
| Beaumont 2018B | Beaumont, R. B. (2018). "24.2 Randomized Controlled Trial of a Serious Gaming Intervention for Children on the Autism Spectrum: Effects on Emotion Regulation and Social Skills." Journal of the American Academy of Child and Adolescent Psychiatry 57(10 suppl): s35-s36. |
| Becker 2017 | Becker, J. L., E. C. Rogers and B. Burrows (2017). "Animal-assisted social skills training for children with autism spectrum disorders." Anthrozoos 30(2): 307-326. |
| Beckloff 1998 | Beckloff, D. R. (1998). "Filial therapy with children with spectrum pervasive development disorders." Dissertation Abstracts International: Section B: The Sciences and Engineering 58(11-b): 6224. |
| Begeer 2011 | Begeer, S., C. Gevers, P. Clifford, M. Verhoeve, K. Kat, E. Hoddenbach and F. Boer (2011). "Theory of mind training in children with autism: A randomized controlled trial." Journal of Autism and Developmental Disorders 41(8): 997-1006. |
| Begeer 2015 | Begeer, S., P. Howlin, E. Hoddenbach, C. Clauser, R. Lindauer, P. Clifford, C. Gevers, F. Boer and H. M. Koot (2015). "Effects and Moderators of a Short Theory of Mind Intervention for Children with Autism Spectrum Disorder: A Randomized Controlled Trial." Autism research : Official Journal of the International Society for Autism Research 8(6): 738-748. |
| Behmanesh 2019 | Behmanesh, H., H. S. Moghaddam, M. R. Mohammadi and S. Akhondzadeh (2019). "Risperidone Combination Therapy with Propentofylline for Treatment of Irritability in Autism Spectrum Disorders: A Randomized, Double-Blind, Placebo-Controlled Clinical Trial." Clinical Neuropharmacology 42(6): 189-196. |
| Bekhet 2017A | Bekhet, A. K. (2017). "Online Positive Thinking Training Intervention for Caregivers of Individuals with ASD: Necessity, Acceptability and Feasibility." Issues in Mental Health Nursing 38(5): 443-448. |
| Bekhet 2017B | Bekhet, A. K. (2017). "Positive Thinking Training Intervention for Caregivers of Persons with Autism: Establishing Fidelity." Archives of Psychiatric Nursing 31(3): 306-310. |
| Belsito 2001 | Belsito, K. M., P. A. Law, K. S. Kirk, R. J. Landa and A. W. Zimmerman (2001). "Lamotrigine therapy for autistic disorder: a randomized, double-blind, placebo-controlled trial." Journal of Autism and Developmental Disorders 31(2): 175-181. |
| Bent 2014 | Bent, S., R. L. Hendren, T. Zandi, K. Law, J. E. Choi, F. Widjaja, L. Kalb, J. Nestle and P. Law (2014). "Internet-based, randomized, controlled trial of omega-3 fatty acids for hyperactivity in autism." Journal of the American Academy of Child and Adolescent Psychiatry 53(6): 658-666. |
| Benton 2011 | Benton, T. D. (2011). "Aripiprazole to treat irritability associated with autism: a placebo-controlled, fixed-dose trial." Current psychiatry reports 13(2): 77?79. |
| Bernaerts 2017 | Bernaerts, S., C. Dillen, J. Steyaert and K. Alaerts (2017). "The effects of four weeks of intranasal oxytocin on social responsiveness and repetitive and restricted behaviors in autism spectrum disorders: A randomized controlled trial." Biological Psychiatry 81(10 suppl 1): s349-s350. |
| Bernard-Opitz 2004 | Bernard-Opitz, V., S. Ing and T. Y. Kong (2004). "Comparison of behavioural and natural play interventions for young children with autism." Autism 8(3): 319-333. |
| Bertoglio 2010 | Bertoglio, K., S. James, L. Deprey, N. Brule and R. L. Hendren (2010). "Pilot study of the effect of methyl B12 treatment on behavioral and biomarker measures in children with autism." The Journal of Alternative and Complementary Medicine 16(5): 555-560. |
| Bettison 1996 | Bettison, S. (1996). "The long-term effects of auditory training on children with autism." Journal of Autism and Developmental Disorders 26(3): 361-374. |
| Bettison 1997 | Bettison, S. (1997). "The long-term effects of auditory training on children with autism." Journal of Autism and Developmental Disorders 27(3): 347?348. |
| Beversdorf 2011 | Beversdorf, D. Q., S. Saklayen, K. F. Higgins, K. E. Bodner, S. M. Kanne and S. E. Christ (2011). "Effect of propranolol on word fluency in autism." Cognitive and Behavioral Neurology 24(1): 11-17. |
| Beversdorf 2014 | Beversdorf, D., R. Zamzow, B. Ferguson, T. Martin, M. Lewis and J. Stichter (2014). "Predictors of response to propranolol for social functioning in autism spectrum disorder." Neurology 82(10 suppl 1). https://n.neurology.org/content/82/10_Supplement/S18.006.short |
| Beversdorf 2015 | Beversdorf, D., B. Ferguson, J. Stichter, E. Porges and R. Zamzow (2016). "Effects of propranolol on verbal problem solving and conversational reciprocity in autism spectrum disorder: A double-blind, single-dose psychopharmacological challenge study." Neuropsychopharmacology 41(suppl 1): s218. |
| Bharathi 2019 | Bharathi, G., A. Venugopal and B. Vellingiri (2019). "Music therapy as a therapeutic tool in improving the social skills of autistic children." Egyptian Journal of Neurology, Psychiatry and Neurosurgery 55(1). |
| Bieleninik 2017 | Bieleninik, L., M. Geretsegger, K. Mossler, J. Assmus, G. Thompson, G. Gattino, C. Elefant, T. Gottfried, R. Igliozzi, F. Muratori, F. Suvini, J. Kim, M. J. Crawford, H. Odell-Miller, A. Oldfield, O. Casey, J. Finnemann, J. Carpente, A. L. Park, E. Grossi and C. Gold (2017). "Effects of improvisational music therapy vs enhanced standard care on symptom severity among children with autism spectrum disorder: The TIME-A randomized clinical trial." Jama - Jama 318(6): 525-535. |
| Black 2012 | Black, D. (2012). "A gluten-free casein-free diet can be beneficial for persons with FASD." Journal of Population Therapeutics and Clinical Pharmacology 19(-3): e408. |
| Blackman 2020 | Blackman, A. L., C. Jimenez-Gomez and S. Shvarts (2020). "Comparison of the efficacy of online versus in-vivo behavior analytic training for parents of children with autism spectrum disorder." Behavior Analysis: Research and Practice 20(1): 13-23. |
| Bolognani 2019 | Bolognani, F., M. Del Valle Rubido, L. Squassante, C. Wandel, M. Derks, L. Murtagh, J. Sevigny, O. Khwaja, D. Umbricht and P. Fontoura (2019). "A phase 2 clinical trial of a vasopressin V1a receptor antagonist shows improved adaptive behaviors in men with autism spectrum disorder." Science Translational Medicine 11(491). |
| Bolte 2016 | Bolte, S. (2016). "Social skills group training: Kontakt for children and adolescents with highfunctioning autism spectrum disorder: A pragmatic multicenter and randomized controlled trial." Journal of the American Academy of Child and Adolescent Psychiatry 55(10 suppl 1): s101-s102. |
| Bonnot 2015A | Bonnot, O., E. Carasco, T. Rabeyron, V. Bisson and F. X. Vrait (2015). "Simple blind randomized controlled trial of music therapy versus music listening in patients with autism spectrum disorders regarding efficiency in behaviour and communication disorders. Preliminary results." European Child and Adolescent Psychiatry 24(1 SUPPL): s115. |
| Bonnot 2015B | Bonnot, O., D. Cohen, F. Muratori, A. Narzisi, S. Viaux, M. Chetouani, T. Rabeyron, V. Bisson, C. Cuevas and B. Azurmendi (2015). "Psychotherapy in autism spectrum disorders-taking account of psychopathology." European Child and Adolescent Psychiatry 24(1 SUPPL): s113-s114. |
| Bordini 2020 | Bordini, D., C. S. Paula, G. R. Cunha, S. C. Caetano, L. F. Bagaiolo, T. C. Ribeiro, M. C. C. Martone, J. Portolese, A. C. Moya, D. Brunoni, C. Bosa, H. Brentani, H. Cogo-Moreira and J. de Jesus Mari (2020). "A randomised clinical pilot trial to test the effectiveness of parent training with video modelling to improve functioning and symptoms in children with autism spectrum disorders and intellectual disability." Journal of Intellectual Disability Research 64(8): 629-643. |
| Borgi 2016 | Borgi, M., D. Loliva, S. Cerino, F. Chiarotti, A. Venerosi, M. Bramini, E. Nonnis, M. Marcelli, C. Vinti, C. De Santis and et al. (2016). "Effectiveness of a Standardized Equine-Assisted Therapy Program for Children with Autism Spectrum Disorder." Journal of Autism and Developmental Disorders 46(1): 1?9. |
| Borowiak 2020 | Borowiak, K. and K. von Kriegstein (2020). "Intranasal oxytocin modulates brain responses to voice-identity recognition in typically developing individuals, but not in ASD." Translational Psychiatry 10(1). |
| Bouvard 1995 | Bouvard, M. P., M. Leboyer, J. M. Launay, C. Recasens, M. H. Plumet, D. Wallerperotte, F. Tabuteau, D. Bondoux, M. Dugas, P. Lensing and J. Panksepp (1995). "LOW-DOSE NALTREXONE EFFECTS ON PLASMA CHEMISTRIES AND CLINICAL SYMPTOMS IN AUTISM - A DOUBLE-BLIND, PLACEBO-CONTROLLED STUDY." Psychiatry Research 58(3): 191-201. |
| Bowrin 2020 | Bowrin, P. and U. Iqbal (2020). "Strengthening Behavior and Social Functioning Among Persons with Autism Spectrum Conditions Using Artificial Intelligence and Behavioral Activation: Protocol for the Well-Being and Health for Loved onEs with ASD (WHOLE) Psychosocial Pilot Randomized Controlled Trial." Studies in health technology and informatics 270: 1399-1400. |
| Boyd 2018 | Boyd, B. A., L. R. Watson, S. S. Reszka, J. Sideris, M. Alessandri, G. T. Baranek, E. R. Crais, A. Donaldson, A. Gutierrez, L. Johnson and K. Belardi (2018). "Efficacy of the ASAP Intervention for Preschoolers with ASD: A Cluster Randomized Controlled Trial." Journal of Autism and Developmental Disorders 48(9): 3144-3162. |
| Bradshaw 2018 | Bradshaw, J., K. Bearss, C. McCracken, T. Smith, C. Johnson, L. Lecavalier, N. Swiezy and L. Scahill (2018). "Parent Education for Young Children With Autism and Disruptive Behavior: Response to Active Control Treatment." Journal of Clinical Child and Adolescent Psychology 47(sup1): s445-s455. |
| Bradshaw 2019 | Bradshaw, J., F. Shic, A. N. Holden, E. J. Horowitz, A. C. Barrett, T. C. German and T. W. Vernon (2019). "The Use of Eye Tracking as a Biomarker of Treatment Outcome in a Pilot Randomized Clinical Trial for Young Children with Autism." Autism Research. |
| Brainsway 2013 | Brainsway and H. M. Organization (2013). "Deep rTMS (Repetitive Transcranial Magnetic Stimulation)for Treatment of Autism Symptoms in Children.": https://clinicaltrials.gov/ct2/show/NCT01388179 |
| Brian 2017 | Brian, J. A., I. M. Smith, L. Zwaigenbaum and S. E. Bryson (2017). "Cross-site randomized control trial of the Social ABCs caregiver-mediated intervention for toddlers with autism spectrum disorder." Autism Research 10(10): 1700-1711. |
| Brito 2020 | Brito, A. R., G. D. P. T. Vairo, A. P. B. H. Dias, B. Olej, O. J. M. Nascimento and M. M. Vasconcelos (2020). "Effect of prednisolone on language function in children with autistic spectrum disorder: a randomized clinical trial." Jornal de Pediatria. |
| Brookma-Frazee 2019 | Brookman-Frazee, L., S. Roesch, C. Chlebowski, M. Baker-Ericzen and W. Ganger (2019). "Effectiveness of training therapists to deliver an individualized mental health intervention for children with asd in publicly funded mental health services: A cluster randomized clinical trial." JAMA Psychiatry 76(6): 574-583. |
| Brookma-Frazee 2020 | Brookman-Frazee, L., C. Chlebowski, M. Villodas, S. Roesch and K. Martinez (2020). "Training Community Therapists to Deliver a Mental Health Intervention for Autism Spectrum Disorder: Changes in Caregiver Outcomes and Mediating Role on Child Outcomes." Journal of the American Academy of Child and Adolescent Psychiatry. 31. |
| Brookman-Frazee 2018 | Brookman-Frazee, L. and A. C. Stahmer (2018). "Effectiveness of a multi-level implementation strategy for ASD interventions: study protocol for two linked cluster randomized trials." Implementation science : IS 13(1): 66. |
| Brunero 2009 | Brunero, M., P. Bailo and R. Boldorini (2009). "Clinical study of the effectiveness of the diet without gluten and casein and anti-inflammatory intestinal changing bowel and psychiatric symptoms in patients with infantile autism. Study and preliminary results." Digestive and Liver Disease 41s(s3): s225. |
| Buchsbaum 2001 | Buchsbaum, M. S., E. Hollander, M. M. Haznedar, C. Tang, J. Spiegel-Cohen, T. C. Wei, A. Solimando, B. R. Buchsbaum, D. Robins, C. Bienstock, C. Cartwright and S. Mosovich (2001). "Effect of fluoxetine on regional cerebral metabolism in autistic spectrum disorders: a pilot study." International Journal of Neuropsychopharmacology 4(2): 119-125. |
| Buitelaar 1992 | Buitelaar, J. K., H. Van Engeland, K. De Kogel, H. De Vries, J. Van Hooff and J. Van Ree (1992). "The adrenocorticotrophic hormone (4-9) analog ORG 2766 benefits autistic children: Report on a second controlled clinical trial." Journal of the American Academy of Child and Adolescent Psychiatry 31(6): 1149-1156. |
| Buitelaar 1996 | Buitelaar, J. K., S. Willemsen-Swinkels and H. Van Engeland (1996). "Treatment of autism and self-injury with naltrexone." Xth world congress of psychiatry; 1996 aug 23-28; madrid, spain. |
| Byford 2015 | Byford, S., M. Cary, B. Barrett, C. R. Aldred, T. Charman, P. Howlin, K. Hudry, K. Leadbitter, A. Le Couteur, H. McConachie, A. Pickles, V. Slonims, K. J. Temple, J. Green, K. Bourne, L. Blazey, C. Holt, D. Kapadia, W. MacDonald, L. White, T. Houghton, C. Taylor, A. Cutress, S. Leach, S. Barron, R. Colmer, S. Randles, K. Beggs and J. Collino (2015). "Cost-effectiveness analysis of a communication-focused therapy for pre-school children with autism: Results from a randomised controlled trial." BMC Psychiatry 15(1 316). |
| Caihong 2016 | Caihong, S., Z. Mingyang, X. Wei, W. Lijie and Z. Dong (2016). "Efficacy of Folic Acid Supplementation in Autistic Children Participating in Structured Teaching: An Open-Label Trial." Nutrients 8(6): 337. |
| Campbell 1978 | Campbell, M., L. T. Anderson, M. Meier, I. L. Cohen, A. M. Small, C. Samit and E. J. Sachar (1978). "A comparison of haloperidol and behavior therapy and their interaction in autistic children." Journal of the American Academy of Child Psychiatry 17(4): 640-655. |
| Campbell 1982 | Campbell, M., L. T. Anderson and I. L. Cohen (1982). "Haloperidol in autistic children: Effects on learning, behavior, and abnormal involuntary movements." Psychopharmacology Bulletin 18(1): 110-111. |
| Campbell 1990 | Campbell, M., L. T. Anderson, A. M. Small, J. J. Locascio, N. S. Lynch and M. C. Choroco (1990). "Naltexone in autistic children: A double-blind and placebo-controlled study." Psychopharmacology Bulletin 26(1): 130-135. |
| Campillo 2014 | Campillo, C., G. Herrera, C. Remírez de Ganuza, J. L. Cuesta, R. Abellán, A. Campos, I. Navarro, J. Sevilla, C. Pardo and F. Amati (2014). "Using Tic-Tac software to reduce anxiety-related behaviour in adults with autism and learning difficulties during waiting periods: A pilot study." Autism: The International Journal of Research and Practice 18(3): 264-271. |
| Capano 2018 | Capano, L., A. Dupuis, J. Brian, D. Mankad, L. Genore, R. Hastie Adams, S. Smile, T. Lui, D. Odrobina, J. A. Foster and E. Anagnostou (2018). "A pilot dose finding study of pioglitazone in autistic children." Molecular Autism 9(1 59). |
| Carey 2016 | Carey, G., S. Parker, P. Constable and C. Sprick (2016). "Can a virtual tour reduce anxiety in young optometry patients with autism spectrum disorder?" Journal of Intellectual Disability Research 60(7-8): 647. |
| Cariveau 2019 | Cariveau, T., M. A. Shillingsburg, A. Alamoudi, T. Thompson, B. Bartlett, S. Gillespie and L. Scahill (2019). "Brief Report: Feasibility and Preliminary Efficacy of a Behavioral Intervention for Minimally Verbal Girls with Autism Spectrum Disorder." Journal of Autism and Developmental Disorders 49(5): 2203-2209. |
| Carminati 2016 | Carminati, G. G., F. Gerber, B. Darbellay, M. M. Kosel, N. Deriaz, J. Chabert, M. Fathi, G. Bertschy, F. Ferrero and F. Carminati (2016). "Using venlafaxine to treat behavioral disorders in patients with autism spectrum disorder." Progress in Neuro-Psychopharmacology and Biological Psychiatry 65: 85-95. |
| Carolina 2011 | Carolina, M. (2011). "Biomarkers in Autism of Aripiprazole and Risperidone Treatment (BAART)." https://clinicaltrials.gov/ct2/show/NCT01333072 |
| Carr 2016 | Carr, T., W. Shih, K. Lawton, C. Lord, B. King and C. Kasari (2016). "The relationship between treatment attendance, adherence, and outcome in a caregiver-mediated intervention for low-resourced families of young children with autism spectrum disorder." Autism 20(6): 643-652. |
| Carrick 2018 | Carrick, F. R., G. Pagnacco, A. Hankir, M. Abdulrahman, R. Zaman, E. R. Kalambaheti, D. A. Barton, P. E. Link and E. Oggero (2018). "The Treatment of Autism Spectrum Disorder With Auditory Neurofeedback: A Randomized Placebo Controlled Trial Using the Mente Autism Device." Frontiers in neurology [electronic resource] 9: 537. |
| Carter 2011 | Carter, A. S., D. S. Messinger, W. L. Stone, S. Celimli, A. S. Nahmias and P. Yoder (2011). "A randomized controlled trial of Hanen's 'More Than Words' in toddlers with early autism symptoms." Journal of child psychology and psychiatry, and allied disciplines" 52(7): 741-752. |
| Carter Alena 2005 | Carter Alena, M. (2005). "Stress reduction for parents of children with autism: a comparison of stress inoculation training and social support." https://www.cochranelibrary.com/central/doi/10.1002/central/CN-01706778/full |
| Castorina 2011 | Castorina, L. L. and L. M. Negri (2011). "The inclusion of siblings in social skills training groups for boys with Asperger syndrome." Journal of Autism and Developmental Disorders 41(1): 73-81. |
| Cell 2017 | Cell, V. and G. Technology (2017). "Autologous Bone Marrow Stem Cell Therapy Combined With Psychological Therapy and Rehabilitation for Autism." https://clinicaltrials.gov/ct2/show/NCT03225651 |
| Center 2002 | Center, M. M., N. Disorders and Stroke (2002). "Divalproex Sodium vs. Placebo in Childhood/Adolescent Autism." https://clinicaltrials.gov/ct2/show/NCT00211757 |
| Center 2005 | Center, M. M. (2005). "Divalproex Sodium ER in Adult Autism." https://clinicaltrials.gov/ct2/show/NCT00211796 |
| Center 2009 | Center, B. M., Hrsa/Maternal and C. H. Bureau (2009). "Supporting the Well Being of Families of Young Children With Autism Spectrum Disorders." https://clinicaltrials.gov/ct2/show/NCT01021384 |
| Center 2010 | Center, S. M. and T. Pediatrics (2010). "Treatment With Acetyl-Choline Esterase Inhibitors in Children With Autism Spectrum Disorders." https://clinicaltrials.gov/ct2/show/NCT01098383 |
| Center 2011 | Center, M. M. and F. Laboratories (2011). "Milnacipran in Autism and the Functional Locus Coeruleus and Noradrenergic Model of Autism." https://clinicaltrials.gov/ct2/show/NCT01337700 |
| Center 2012 | Center, E. M. and U. University (2012). "Efficacy of RAD001/Everolimus in Autism and NeuroPsychological Deficits in Children With Tuberous Sclerosis Complex." https://clinicaltrials.gov/ct2/show/NCT01730209 |
| Center 2014 | Center, B. I. D. M. and B. University (2014). "Testing a Novel Speech Intervention in Minimally Verbal Children With Autism Spectrum Disorder (ASD)." https://clinicaltrials.gov/ct2/show/NCT03015272 |
| Center 2016 | Center, R. U. M., U. Chicago, U. Chicago, N. University and E. L. University (2016). "Oxytocin and Social Cognitive Skills Groups." https://clinicaltrials.gov/ct2/show/NCT02918864 |
| Center 2017 | Center, S. Z. M. (2017). "Cannabinoids for Behavioral Problems in Children With ASD." https://clinicaltrials.gov/ct2/show/NCT02956226 |
| Center 2020A | Center, R. M. (2020). Intranasal Dexmedetomidine vs Oral Triclofos Sodium for EEG in Children With Autism, https://ClinicalTrials.gov/show/NCT04270708. |
| Center 2020B | Center, S. M. (2020). Treatment of Children With Autistic Spectrum Disorder With Autologous Umbilical Cord Blood, a Pilot Study, https://ClinicalTrials.gov/show/NCT04243382. |
| Center for Autisme 2006 | Center for Autisme, D., N. Settlement, U. K. The Robert Luff Foundation, D. Eric Birger Christensen Fond and N. Norsk Proteinintolerance (2006). "ScanBrit Dietary Intervention in Autism." https://clinicaltrials.gov/ct2/show/NCT00614198 |
| Central Hospital 2015 | Central Hospital, N. F. (2015). "Folinic Acid in Children With Autism Spectrum Disorders." https://clinicaltrials.gov/ct2/show/NCT02551380 |
| Cermak 2015 | Cermak, S., L. Stein Duker, M. Williams, M. Dawson, C. Lane and J. Polido (2015). "Sensory Adapted Dental Environments to Enhance Oral Care for Children with Autism Spectrum Disorders: A Randomized Controlled Pilot Study." Journal of Autism and Developmental Disorders 45(9): 2876-2888. |
| Chaitanya Hospital 2014 | Chaitanya Hospital, P. (2014). "A Clinical Trial to Study the Safety and Efficacy of Bone Marrow Derived Autologous Cells for the Treatment of Autism." https://clinicaltrials.gov/ct2/show/NCT01836562 |
| Chan 2013 | Chan, A. S., S. L. Sze, N. Y. Siu, E. M. Lau and M. C. Cheung (2013). "A Chinese Mind-Body Exercise Improves Self-Control of Children with Autism: A Randomized Controlled Trial." PloS One 8(7 e68184). |
| Chan 2014 | Chan, A. S., S. L. Sze and Y. M. Y. Han (2014). "An intranasal herbal medicine improves executive functions and activates the underlying neural network in children with autism." Research in Autism Spectrum Disorders 8(6): 681-691. |
| Chen 2020 | Chen, M. T., Y. P. Chang, M. E. Marraccini, M. C. Cho and N. W. Guo (2020). "Comprehensive attention training system (CATS): A computerized executive-functioning training for school-aged children with autism spectrum disorder." International Journal of Developmental Disabilities. |
| Cheng 2018A | Cheng, Y., S. Luo, H. Lin and C. Yang (2018). "Investigating mobile emotional learning for children with autistic spectrum disorders." International journal of developmental disabilities 64(1): 25‐34. |
| Cheng 2018B | Cheng, Y., S. Y. Luo, H. C. Lin and C. S. Yang (2018). "Investigating mobile emotional learning for children with autistic spectrum disorders." International Journal of Developmental Disabilities 64(1): 25-34. |
| Chester 2019 | Chester, M., A. L. Richdale and J. McGillivray (2019). "Group-Based Social Skills Training with Play for Children on the Autism Spectrum." Journal of Autism and Developmental Disorders 30: 30. |
| Chez 2016 | Chez, M., C. Lepage, C. Parise, A. Dang-Chu and A. Hankins (2016). "A randomized, blinded, placebo-controlled, crossover study to assess the efficacy of stem cells from autologous umbilical cord blood to improve language and behavior in children with autism." Cytotherapy 18(6 SUPPL): s112. |
| Chez 2018 | Chez, M., C. Lepage, C. Parise, A. Dang-Chu, A. Hankins and M. Carroll (2018). "Safety and Observations from a Placebo-Controlled, Crossover Study to Assess Use of Autologous Umbilical Cord Blood Stem Cells to Improve Symptoms in Children with Autism." Stem cells translational medicine 7(4): 333?341. |
| Chi 2012 | Chi, C. T. (2012). "Mind-Body Exercise for Children with Autism Spectrum Disorders." Http://wwwwhoint/trialsearch/trial2aspx? Trialid=chictr-trc-12002561. |
| Chi 2017 | Chi, C. I. (2017). "Improving well-being of children with Autistic Spectrum Disorder (ASD) and their families with mindfulness training in Hong Kong." http://apps.who.int/trialsearch/Trial2.aspx?TrialID=ChiCTR-IPR-17011233 |
| Chictr 2018A | ChiCtr (2018). "A clinical research of transcranial direct current stimulation in the treatment for children with autism spectrum disorders." |
| Chictr 2018B | ChiCtr (2018). "Clinical study of Fecal microbiota transplantation for treatment of autism." |
| Chictr 2018C | ChiCtr (2018). "Core feature target intervention in the treatment of children with autism spectrum disorders." http://www.chictr.org.cn/showproj.aspx?proj=31693 |
| Chictr 2018D | ChiCtr (2018). "Effects of sensory motor training and yoga on motor coordination and balance for children with ASD and Developmental Disabilities." |
| Chictr 2018E | ChiCtr (2018). "Mindfulness based intervention for Chinese children with autism and their parents: a randomised controlled trial." |
| Chictr 2018F | ChiCtr (2018). "Prospective Study of Vitamin A and Vitamin D Treatment in Children with Autism Spectrum Disorders." |
| Chictr 2018G | ChiCtr (2018). "A Random, Double-blind, Placebo Controlled trial for Oxytocin Nasal Spray in the Treatment for ASD Social Dysfunction." |
| Chictr 2018H | ChiCtr (2018). "A randomised controlled trial for investigating the effectiveness of parent training on promoting physical activity in children with autism spectrum disorder." |
| Chictr 2018I | ChiCtr (2018). "Clinical study for massage to improve the symptoms of children with autism spectrum disorders." http://www.chictr.org.cn/showproj.aspx?proj=33299 |
| Chictr 2018J | ChiCtr (2018). "Development of gesture ability in children with autism spectrum disorders and its efficacy in predicting curative effect." http://www.chictr.org.cn/showproj.aspx?proj=32786 |
| Chictr 2018K | ChiCtr (2018). "The effect of improving gut microbiota for treating children with Autism Spectrum Disorder(ASD)." http://www.chictr.org.cn/showproj.aspx?proj=22025 |
| Chictr 2018L | ChiCtr (2018). "Effect of intestinal microecological reconstruction on autistic children’s symptoms and preliminary mechanism exploration." |
| Chictr 2018M | ChiCtr (2018). "Research for Intervention in Autism Spectrum Disorders Children by repetitive Transcranial Magnetic Stimulation with Magnetic Resonance Imaging Navigation and Evaluation of its Clinical Intervention Effect by DTI and IHMT Sequences of Magnetic Resonance Imaging." http://www.chictr.org.cn/showproj.aspx?proj=31078 |
| ChiCTR 2019A | ChiCTR1900022646 (2019). Effects of probiotics on social behavior, serum biochemical parameters, and intestinal/oral flora in children with autism spectrum disorders and Prader-Willi Syndrome. |
| ChiCTR 2019B | ChiCTR1900023363 (2019). Effect of aerobic exercise on gut microbiota in children with autism spectrum disorders. |
| ChiCTR 2019C | ChiCTR1900023774 (2019). The effects of chronic intranasal oxytocin in the treatment of young children with autism. |
| ChiCTR 2019D | ChiCTR1900024973 (2019). A series of studies for adaptive sports activities promoting the development of brain function in children with autism. |
| ChiCTR 2019E | ChiCTR1900027145 (2019). The improvement ffects of transcranial direct current stimulation (tDCS) on the symptoms in children with autism spectrum disorder (ASD). |
| ChiCTR 2019F | ChiCTR1900028552 (2019). Study for clinical and community application of caregivers skills training for autistic children. |
| ChiCTR 2020A | ChiCTR2000028896 (2020). Effects and Mechanisms of Parent-Child Creative Art Therapy on Preschool Children with Autism Spectrum Disorder and Their Mothers. |
| ChiCTR 2020B | ChiCTR2000028985 (2020). Clinical study for intervention effect of the probiotics in autistic children. |
| ChiCTR 2020C | ChiCTR2000029256 (2020). Study for the effect of Intervention Training of Lexiang Gymnastics for the Problem Behavior of Autistic Children. |
| ChiCTR 2020D | ChiCTR2000029357 (2020). Positive Regulation of the Social and Emotional Disorders and Brain-Gut Axis Mechanisms for Children with Autism Spectrum Disorders by Jin's 3 needle Technique. |
| ChiCTR 2020E | ChiCTR2000032605 (2020). Clinical Randomized Controlled Trial for Repeated Transcranial Magnetic Stimulation on Sleep Disorder in Children with Autism Spectrum Disorder. |
| ChiCTR 2020F | ChiCTR2000032853 (2020). Effect of mindfulness training combined with Baduanjin exercise on school-age children with autism spectrum disorder and their parents. |
| ChiCTR 2020G | ChiCTR2000033042 (2020). The effect and mechanism of dolphin house digital bionic training system on children with autism spectrum disorder: a randomized controlled trial. |
| ChiCTR 2020H | ChiCTR2000033586 (2020). A randomized controlled study on the mechanism of frontoparietal disconnection and its regulation in autism spectrum disorders. |
| ChiCTR 2020I | ChiCTR2000033736 (2020). Fecal microbiota transplantation in the treatment of young Chinese children with autism spectrum disorders: a multicenter double-blind randomized controlled trial. |
| ChiCTR 2020J | ChiCTR2000034439 (2020). Research on New Methods of Diagnosis and Treatment of Autism Spectrum Disorder. |
| ChiCTR 2020K | ChiCTR2000035006 (2020). Preliminary clinical study on probiotics Bacteroides fragilis BF839 in Children with Autism Spectrum Disorder (ASD). |
| ChiCTR 2020L | ChiCTR2000035176 (2020). Intervention effect of World Health Organization caregiver parenting skills training on autism spectrum disorders. |
| ChiCTR 2020M | ChiCTR2000035624 (2020). Fecal microbiota transplantation for children with ASD: a multicenter, randomized, double-blind controlled study. |
| Chictr-Ccc 2013 | ChiCtr-Ccc (2013). "The associate of polymorphisms of vitamin D metabolism-related genes with autism, and the treatment of autism with vitamin D." http://www.chictr.org.cn/showproj.aspx?proj=5074 |
| Chictr-Iir 2016 | ChiCtr-Iir (2016). "Fecal Microbiota Preparation Treatment for Autism Spectrum Disorder: A Prospective, Open-label, Randomized, Controlled Trial." |
| Chictr-Inr 2017 | ChiCtr-Inr (2017). "A clinical research of transcranial direct current stimulation in the treatment for children with autism spectrum disorders." |
| Chictr-Ior 2016 | ChiCtr-Ior (2016). "The brain functional promotion of auricular concha electro-acupuncture on children with autism spectrum disorder." |
| Chictr-Ior 2017A | ChiCtr-Ior (2017). "A comparative study of three types of rehabilitation interventions for autism spectrum disorders." |
| Chictr-Ior 2017B | ChiCtr-Ior (2017). "The effect of an game-based exercise training program on promoting physical fitness and mental health in children with autism spectrum disorder." |
| Chictr-Ipr 2016 | ChiCtr-Ipr (2016). "A randomized double-blind placebo-controlled trial of the efficiency and mechanism of bumetanide on children with Autism Spectrum Disorder." |
| Chictr-Ipr 2017A | ChiCtr-Ipr (2017). "the comprehensive intervention study of autistic children based on the theory of multiple intelligences." |
| Chictr-Ipr 2017B | ChiCtr-Ipr (2017). "Effects of acupuncture on the brain neurochemical substrate metabolism status in non-verbal autistic children." |
| Chictr-Ipr 2017C | ChiCtr-Ipr (2017). "Improving well-being of children with Autistic Spectrum Disorder (ASD) and their families with mindfulness training in Hong Kong." |
| Chictr-Onrc 2012 | ChiCtr-Onrc (2012). "Rudimental research into effects of combination therapy of rehabilitation training and oral bumetanide on children with autism." |
| Chictr-Oon 2014 | ChiCtr-Oon (2014). "The therapeutic effect and mechanism of ketogenic diet for children with autism." |
| Chictr-Oon 2017 | ChiCtr-Oon (2017). "Exercise training program for preschool children with autism spectrum disorder." |
| Chictr-Tnrc 2011 | ChiCtr-Tnrc (2011). "Studies on effect of transcutaneous electrical acupoint stimulation on children with autism and primary mechanisms." |
| Chictr-Trc 2009 | ChiCtr-Trc (2009). "The research of the effect of multisensory interactive training system ZM8.1." |
| Chictr-Trc 2012 | ChiCtr-Trc (2012). "Mind-Body Exercise for Children with Autism Spectrum Disorders." |
| Chictr-Trc 2014 | ChiCtr-Trc (2014). "Combination of intranasal dexmedetomidine and oral midazolam in autism children undergoing computer tomographic: a prospective randomized controlled trial." |
| Children 1999 | Children, T. (1999). "Fluvoxamine and Sertraline in Childhood Autism - Does SSRI Therapy Improve Behaviour and/or Mood?". https://clinicaltrials.gov/ct2/show/NCT00655174 |
| Children's Hospital Medical Center 2013A | Children's Hospital Medical Center, C. (2013). "Pilot Study of Riluzole for Drug-Refractory Irritability in Autism Spectrum Disorders." https://clinicaltrials.gov/ct2/show/NCT02081027 |
| Children's Hospital Medical Center 2013B | Children's Hospital Medical Center, C. and A. Speaks (2013). "Study of Acamprosate in Autism." https://clinicaltrials.gov/ct2/show/NCT01813318 |
| Chlebowski 2020 | Chlebowski, C., E. Hurwich-Reiss, B. Wright and L. Brookman-Frazee (2020). "Using stakeholder perspectives to guide systematic adaptation of an autism mental health intervention for Latinx families: A qualitative study." Journal of community psychology 48(4): 1194-1214. |
| Choque Olsson 2016 | Choque Olsson, N., D. Rautio, J. Asztalos, U. Stoetzer and S. Bolte (2016). "Social skills group training in high-functioning autism: A qualitative responder study." Autism 20(8): 995-1010. |
| Choque Olsson 2017 | Choque Olsson, N., O. Flygare, C. Coco, A. Gorling, A. Rade, Q. Chen, K. Lindstedt, S. Berggren, E. Serlachius, U. Jonsson, K. Tammimies, L. Kjellin and S. Bolte (2017). "Social Skills Training for Children and Adolescents With Autism Spectrum Disorder: A Randomized Controlled Trial." Journal of the American Academy of Child and Adolescent Psychiatry 56(7): 585-592. |
| Chu 2012 | Chu, C. H. and C. Y. Pan (2012). "The effect of peer- and sibling-assisted aquatic program on interaction behaviors and aquatic skills of children with autism spectrum disorders and their peers/siblings." Research in Autism Spectrum Disorders 6(3): 1211-1223. |
| Chugani 2004 | Chugani, D. C. (2004). "Early Pharmacotherapy Aimed at Neuroplasticity in Autism : Safety and Efficacy." https://clinicaltrials.gov/ct2/show/NCT00166621 |
| Chugani 2009 | Chugani, D. C., N. Disorders and Stroke (2009). "Buspirone in the Treatment of 2-6 Year Old Children With Autistic Disorder." |
| Chugani 2016 | Chugani, D. C., H. T. Chugani, M. Wiznitzer, S. Parikh, P. A. Evans, R. L. Hansen, R. Nass, J. J. Janisse, P. Dixon-Thomas, M. Behen, R. Rothermel, J. S. Parker, A. Kumar, O. Muzik, D. J. Edwards, D. Hirtz and N. Autism Center of Excellence (2016). "Efficacy of Low-Dose Buspirone for Restricted and Repetitive Behavior in Young Children with Autism Spectrum Disorder: A Randomized Trial." Journal of Pediatrics 170: 45-53.e41-44. |
| Cibrian 2020 | Cibrian, F. L., M. Madrigal, M. Avelais and M. Tentori (2020). "Supporting coordination of children with ASD using neurological music therapy: A pilot randomized control trial comparing an elastic touch-display with tambourines." Research in Developmental Disabilities 106 (no pagination). |
| Cidav 2017 | Cidav, Z., J. Munson, A. Estes, G. Dawson, S. Rogers and D. Mandell (2017). "Cost Offset Associated With Early Start Denver Model for Children With Autism." Journal of the American Academy of Child and Adolescent Psychiatry 56(9): 777-783. |
| Clifford 2013 | Clifford, T. and P. Minnes (2013). "Logging on: evaluating an online support group for parents of children with autism spectrum disorders." Journal of Autism and Developmental Disorders 43(7): 1662-1675. |
| Clinical 2014A | Clinical, U. and T. S. Institute (2014). "Single Dose Intranasal Oxytocin and Cognitive Effects in Autism." https://clinicaltrials.gov/ct2/show/NCT02493426 |
| Clinical 2014B | Clinical, U., T. S. Institute and C. Philadelphia (2014). "Intranasal Oxytocin and Learning in Autism." https://clinicaltrials.gov/ct2/show/NCT02090829 |
| Coggins 1988 | Coggins, T. E., C. Morisset, L. Krasney, R. Frederickson, V. A. Holm and V. A. Raisys (1988). "Brief Report: Does fenfluramine treatment enhance the cognitive and communicative functioning of autistic children." Journal of Autism and Developmental Disorders 18(3): 425-434. |
| Collard 1969 | Collard, J. (1969). "[Sulpiride, an unusual neuroleptic anti-autism and thymoanaleptic drug]." Semaine des Hopitaux 45(48): 3028-3033. |
| Conner 2013 | Conner, C. M., B. B. Maddox and S. W. White (2013). "Parents' state and trait anxiety: relationships with anxiety severity and treatment response in adolescents with autism spectrum disorders." Journal of Autism and Developmental Disorders 43(8): 1811-1818. |
| Cook 2019 | Cook, J. M., C. L. Donovan and M. S. Garnett (2019). "Parent-mediated cognitive behavioural therapy for young children with high-functioning autism spectrum disorder and anxiety: A randomized control trial." Early Child Development and Care 189(1): 119-134. |
| Corbett 2008 | Corbett, B. A., K. Shickman and E. Ferrer (2008). "Brief report: The effects of Tomatis sound therapy on language in children with autism." Journal of Autism and Developmental Disorders 38(3): 562-566. |
| Corbett 2011 | Corbett, B., J. Gunther, D. Comins, J. Price, N. Ryan, D. Simon, C. Schupp and T. Rios (2011). "Brief Report: Theatre as Therapy for Children with Autism Spectrum Disorder." Journal of Autism and Developmental Disorders 41(4): 505-511. |
| Corbett 2016A | Corbett, B. A., K. L. Bales, D. Swain, K. Sanders, T. A. R. Weinstein and L. J. Muglia (2016). "Comparing oxytocin and cortisol regulation in a double-blind, placebo-controlled, hydrocortisone challenge pilot study in children with autism and typical development." Journal of Neurodevelopmental Disorders 8(1 32). |
| Corbett 2016B | Corbett, B. A., A. P. Key, L. Qualls, S. Fecteau, C. Newsom, C. Coke and P. Yoder (2016). "Improvement in Social Competence Using a Randomized Trial of a Theatre Intervention for Children with Autism Spectrum Disorder." Journal of Autism and Developmental Disorders 46(2): 658-672. |
| Corbett 2019 | Corbett, B. A., S. Ioannou, A. P. Key, C. Coke, R. Muscatello, S. Vandekar and I. Muse (2019). "Treatment Effects in Social Cognition and Behavior following a Theater-based Intervention for Youth with Autism." Developmental neuropsychology 44(7): 481-494. |
| Coronado Biosciences 2014 | Coronado Biosciences, I. (2014). "Efficacy and Safety of Trichuris Suis Ova (TSO) as Compared to Placebo in Autism Spectrum Disorder." https://clinicaltrials.gov/ct2/show/NCT02140112 |
| Corporation 2002A | Corporation, R. (2002). "Synthetic Human Secretin in Children With Autism." https://clinicaltrials.gov/ct2/show/NCT00036244 |
| Corporation 2002B | Corporation, R. (2002). "Synthetic Human Secretin in Children With Autism and Gastrointestinal Dysfunction." https://clinicaltrials.gov/ct2/show/NCT00036231 |
| Corti 2018 | Corti, C., F. Pergolizzi, L. Vanzin, G. Cargasacchi, L. Villa, M. Pozzi and M. Molteni (2018). "Acceptance and commitment therapy-oriented parent-training for parents of children with autism." Journal of Child and Family Studies: no pagination specified. |
| Costescu 2017 | Costescu, C. A., B. Vanderborght and D. O. David (2017). "Robot-enhanced CBT for dysfunctional emotions in social situations for children with ASD." Journal of Evidence-Based Psychotherapies 17(2): 119-132. |
| Council 2009A | Council, T. and B. Pharmaceutical (2009). "Sapropterin as a Treatment for Autistic Disorder." https://clinicaltrials.gov/ct2/show/NCT00850070 |
| Council 2009B | Council, T. and B. Pharmaceutical (2009). "Open-Label Extension Study of Kuvan for Autism." https://clinicaltrials.gov/ct2/show/NCT00943579 |
| Crawford 2017 | Crawford, M. J., C. Gold, H. Odell-Miller, L. Thana, S. Faber, J. Assmus, L. Bieleninik, M. Geretsegger, C. Grant, A. Maratos, S. Sandford, A. Claringbold, H. McConachie, M. Maskey, K. A. Mossler, P. Ramchandani and A. Hassiotis (2017). "International multicentre randomised controlled trial of improvisational music therapy for children with autism spectrum disorder: TIME-A study." Health Technology Assessment 21(59): 1-66. |
| Crowell 2020 | Crowell, C., B. Sayis, J. P. Benitez and N. Pares (2020). "Mixed reality, full-body interactive experience to encourage social initiation for autism: Comparison with a control nondigital intervention." Cyberpsychology, Behavior, and Social Networking 23(1): 5-9. |
| CTRI 2015 | CTRI/2015/12/006418 (2015). Feasibility and Effectiveness of home based sensory interventions in children with autism and sensory processing abnormalities as compared to standard care : An Open label Randomized study. |
| Ctri 2016 | Ctri (2016). "Effectiveness of Homoeopathic medicines in Autism." http://www.ctri.nic.in/Clinicaltrials/pmaindet2.php?trialid=3840 |
| Ctri 2017A | Ctri (2017). "Mantha sanni ( Autism Spectrum Disorder) in Children." http://www.ctri.nic.in/Clinicaltrials/pmaindet2.php?trialid=17736 |
| Ctri 2017B | Ctri (2017). "A study to compare two intervention therapy programs in children with autism, one involving weekly visits and other involving 6 weekly visits." |
| Ctri 2017C | Ctri (2017). "Yoga intervention for autism spectrum disorder children." http://www.ctri.nic.in/Clinicaltrials/pmaindet2.php?trialid=11520 |
| CTRI 2017D | CTRI/2017/05/008454 (2017). An open label pilot study of abhay ghrita in the management of autistic disorder. |
| Ctri 2017E | Ctri (2017). "A study on brief parent delivered intervention for children with Autism." http://apps.who.int/trialsearch/Trial2.aspx?TrialID=CTRI/2017/05/008672 |
| Ctri 2018A | Ctri (2018). "Effect of Virtual Reality Distraction on Pain and Anxiety During Dental Treatment in Autistic Children." http://www.ctri.nic.in/Clinicaltrials/pmaindet2.php?trialid=25005 |
| Ctri 2018B | Ctri (2018). "Vedic Chants Intervention program (VCIP) for management of children with Autism." http://www.ctri.nic.in/Clinicaltrials/pmaindet2.php?trialid=28457 |
| Ctri 2018C | Ctri (2018). "Effect of Abhaya Ghrita and Panchabhautika Taila Nasya in treating Autism Spectrum Disorders in children." http://www.ctri.nic.in/Clinicaltrials/pmaindet2.php?trialid=20999 |
| Ctri 2018D | Ctri (2018). "Ayurveda for autism." |
| Ctri 2019A | Ctri (2019). "comparing two different methods of aids to help toothbrushing among autistic children." http://www.ctri.nic.in/Clinicaltrials/pmaindet2.php?trialid=30576 |
| Ctri 2019B | Ctri (2019). "Efficacy of Transcranial Magnetic Stimulation in children with Autism Spectrum Disorder." |
| CTRI 2019C | CTRI/2019/04/018568 (2019). Effect of L-carnosine versus early intensive behavioural intervention (EIBI) in children with Autism. |
| CTRI 2019D | CTRI/2019/07/020102 (2019). Effect of supplementations in children with Autism Spectrum Disorders. |
| CTRI 2019E | CTRI/2019/07/020399 (2019). Ayurveda for Autism. |
| CTRI 2019F | CTRI/2019/09/021079 (2019). Effect of vitamin D in autism spectrum disorder: A clinical trial. |
| CTRI 2020A | CTRI/2020/01/022745 (2020). IMPROVEMENT OF ORAL HYGIENE BY VIDEO MODELLING IN AUTISTIC CHILDREN. |
| CTRI 2020B | CTRI/2020/02/023460 (2020). Vision therapy for Autism. |
| CTRI 2020C | CTRI/2020/06/025871 (2020). A study to observe additional effect of ayurvedic treatment on Autism Spectrum Disorder in Children. |
| CTRI 2020D | CTRI/2020/07/026285 (2020). Siddha Management for Maantha Sanni (Autism Spectrum Disorder) in Children. |
| CTRI 2020E | CTRI/2020/08/027099 (2020). Effect of Family Mediated Intervention (FMI) vs Early Intensive Behavioral Intervention (EIBI) in children with Autism spectrum disorder. |
| Curemark 2009 | Curemark (2009). "A Trial of CM-AT in Children With Autism." https://clinicaltrials.gov/ct2/show/NCT00881452 |
| Curemark 2010 | Curemark (2010). "A Trial of CM-AT in Children With Autism- Open Label Extension Study." https://clinicaltrials.gov/ct2/show/NCT00912691 |
| Curemark 2015A | Curemark (2015). "A Trial of CM-AT in Children With Autism With All Levels of FCT (The Blum Study)." https://clinicaltrials.gov/ct2/show/NCT02410902 |
| Curemark 2015B | Curemark (2015). "An Open Label Study of CM-AT for the Treatment of Children With Autism." https://clinicaltrials.gov/ct2/show/NCT02649959 |
| Da Paz 2016 | Da Paz, N. S., J. Tiemensma and J. L. Wallander (2016). "Written disclosure as treatment for parents of children with autism spectrum disorders: A RCT using the cortisol awakening response." Psychoneuroendocrinology 71(suppl 1): 69. |
| Da Paz 2017A | Da Paz, N. S., J. Tiemensma and J. L. Wallander (2017). "A randomized controlled trial evaluating the effect of written disclosure on subjective stress and cortisol activity among parents of children with autism." Psychosomatic Medicine 79(4): a95-a. |
| Da Paz 2017B | Da Paz, N. S. (2017). "Randomized controlled trial to evaluate written disclosure as treatment for parents of children with autism spectrum disorders." Dissertation Abstracts International: Section B: The Sciences and Engineering 77(10-be): no pagination specified. |
| Da Paz 2018 | Da Paz, N. S., J. L. Wallander and J. Tiemensma (2018). "Effects of written disclosure on psychophysiological stress among parents of children with autism: A randomized controlled pilot study." Research in Autism Spectrum Disorders 53: 7-17. |
| Dadds 2014 | Dadds, M., E. MacDonald, A. Cauchi, K. Williams, F. Levy and J. Brennan (2014). "Nasal Oxytocin for Social Deficits in Childhood Autism: A Randomized Controlled Trial." Journal of Autism and Developmental Disorders 44(3): 521-531. |
| Daly 2012 | Daly, E. M., Q. Deeley, C. Ecker, M. Craig, B. Hallahan, C. Murphy, P. Johnston, D. Spain, N. Gillan, M. Brammer and et al. (2012). "Serotonin and the neural processing of facial emotions in adults with autism: an fMRI study using acute tryptophan depletion." Archives of General Psychiatry 69(10): 1003?1013. |
| Danfors 2005 | Danfors, T., A. L. Von Knorring, P. Hartvig, B. Langstrom, R. Moulder, B. Stromberg, R. Torstenson, U. Wester, Y. Watanabe and O. Eeg-Olofsson (2005). "Tetrahydrobiopterin in the treatment of children with autistic disorder: A double-blind placebo-controlled crossover study." Journal of Clinical Psychopharmacology 25(5): 485-489. |
| Davlantis 2015 | Davlantis, K. S. (2015). "Learning opportunities provided by parents to young children with ASD: Developing and testing a measurement system." Dissertation Abstracts International: Section B: The Sciences and Engineering 75(7-be): no pagination specified. |
| Dawalt 2017 | DaWalt, L. S., J. S. Greenberg and M. R. Mailick (2017). "Transitioning Together: a Multi-family Group Psychoeducation Program for Adolescents with ASD and Their Parents." Journal of Autism and Developmental Disorders: 1?13. |
| Dawalt 2018 | DaWalt, L. S., J. S. Greenberg and M. R. Mailick (2018). "Transitioning Together: A Multi-family Group Psychoeducation Program for Adolescents with ASD and Their Parents." Journal of Autism and Developmental Disorders 48(1): 251-263. |
| Dawson 2010 | Dawson, G., S. Rogers, J. Munson, M. Smith, J. Winter, J. Greenson, A. Donaldson and J. Varley (2010). "Randomized, controlled trial of an intervention for toddlers with autism: The early start Denver model." Pediatrics 125(1): e17-e23. |
| de Jong 2019 | de Jong, R. K., H. Snoek, W. G. Staal and H. Klip (2019). "The effect of patients' feedback on treatment outcome in a child and adolescent psychiatric sample: a randomized controlled trial." European Child and Adolescent Psychiatry 28(6): 819-834. |
| Dean 2020 | Dean, M., J. Williams, F. Orlich and C. Kasari (2020). "Adolescents with autism spectrum disorder and social skills groups at school: A randomized trial comparing intervention environment and peer composition." School Psychology Review 49(1): 60-73. |
| Deckers 2016 | Deckers, A., P. Muris, J. Roelofs and A. Arntz (2016). "A Group-Administered social Skills Training for 8- to 12- Year-Old, high-Functioning Children With Autism Spectrum Disorders: An Evaluation of its Effectiveness in a Naturalistic Outpatient Treatment Setting." Journal of Autism and Developmental Disorders 46(11): 3493-3504. |
| Decocq 1996 | Decocq, G., N. Kandelaft and M. Compagnon (1996). "[Effects of naltrexone on automutilation behavior in autistic psychosis]." Presse Medicale 25(7): 305. |
| Dekker 2014 | Dekker, V., M. H. Nauta, E. J. Mulder, M. E. Timmerman and A. de Bildt (2014). "A randomized controlled study of a social skills training for preadolescent children with autism spectrum disorders: Generalization of skills by training parents and teachers?" BMC Psychiatry 14(1 189). |
| Dekker 2020 | Dekker, V., M. H. Nauta, M. E. Timmerman, E. J. Mulder, P. J. Hoekstra and A. de Bildt (2020). "Application of Latent Class Analysis to Identify Subgroups of Children with Autism Spectrum Disorders who Benefit from Social Skills Training." Journal of Autism & Developmental Disorders 05: 05. |
| Del Valle Rubido 2015 | Del Valle Rubido, M., D. Umbricht, F. Shic, J. T. McCracken, L. Scahill, O. Khwaja, L. Squassante, L. Boak, F. Bolognani, P. Fontoura, C. Wall, R. Jou, R. Loomis, M. Lyons, A. Gavaletz, J. Cowen, T. Apelian, S. Jeste, C. Ferretti, B. Taylor, G. Berlin, R. Noone, L. Antar and E. Hollander (2015). "Results from a phase I proof-of-mechanism study with a vasopressin 1A receptor antagonist in autism spectrum disorder." European Neuropsychopharmacology 2): s646-s647. |
| D'elia 2014 | D'Elia, L., G. Valeri, F. Sonnino, I. Fontana, A. Mammone and S. Vicari (2014). "A longitudinal study of the TEACCH program in different settings: The potential benefits of low intensity intervention in preschool children with autism spectrum disorder." Journal of Autism and Developmental Disorders 44(3): 615-626. |
| Demb 1996 | Demb, H. B. (1996). "Risperidone in young children with pervasive developmental disorders and other developmental disabilities." Journal of Child & Adolescent Psychopharmacology 6(1): 79-80. |
| Demurie 2010 | Demurie, E., H. Roeyers, E. Sonuga-Barke and D. Baeyens (2010). "Temporal reward discounting in ADHD and ASD." European Child and Adolescent Psychiatry 19(SUPPL): s85-s86. |
| Derguy 2018 | Derguy, C., M. Poumeyreau, S. Pingault and K. M'Bailara (2018). "[A therapeutic education program for parents of children with ASD: Preliminary results about the effectiveness of the ETAP program]." Encephale 44(5): 421-428. |
| Derosier 2011 | Derosier, M. E., D. C. Swick, N. O. Davis, J. S. McMillen and R. Matthews (2011). "The efficacy of a social skills group intervention for improving social behaviors in children with high functioning autism spectrum disorders." Journal of Autism and Developmental Disorders 41(8): 1033-1043. |
| Desarkar 2017 | Desarkar, P., T. K. Rajji, S. H. Ameis, M. C. Lai, D. Blumberger and Z. J. Daskalakis (2017). "Assessing and stabilizing aberrant neuroplasticity in autism spectrum disorder using transcranial magnetic stimulation: Preliminary findings from a proof-of-principle study." Brain Stimulation 10(-2): 389. |
| Desarkar 2020 | Desarkar, P., T. K. Rajji, S. H. Ameis, D. M. Blumberger, M. C. Lai, Y. Lunsky and Z. J. Daskalakis (2020). "Assessing and Stabilizing Aberrant Neuroplasticity in Autism Spectrum Disorder Using Transcranial Magnetic Stimulation: Results From a Proof-Of-Principle Study." Biological Psychiatry 87 (9 Supplement): S252-S253. |
| Development 2003 | Development, F. and N. Health (2003). "A Controlled Study of Olanzapine in Children With Autism." https://clinicaltrials.gov/ct2/show/NCT00057408 |
| Development 2006A | Development, O. P., I. Commercialization and O. A. Pharmaceutical (2006). "Study of Aripiprazole in the Treatment of Children and Adolescents With Autistic Disorder (AD)." https://clinicaltrials.gov/ct2/show/NCT00332241 |
| Development 2006B | Development, O. P., I. Commercialization and O. A. Pharmaceutical (2006). "Study of Aripiprazole in the Treatment of Serious Behavioral Problems in Children and Adolescents With Autistic Disorder (AD)." https://clinicaltrials.gov/ct2/show/NCT00365859 |
| Development 2019 | Development, O. P., I. Commercialization and H. L. A/S (2019). Brexpiprazole in Treatment of Children and Adolescents With Irritability Associated With Autism Spectrum Disorder, https://ClinicalTrials.gov/show/NCT04174365. |
| Dickson 2020 | Dickson, K. S., C. Chlebowski, R. Haine-Schlagel, B. Ganger and L. Brookman-Frazee (2020). "Impact of Therapist Training on Parent Attendance in Mental Health Services for Children with ASD." Journal of clinical child and adolescent psychology : the official journal for the Society of Clinical Child and Adolescent Psychology, American Psychological Association, Division 53: 1-12. |
| Dille 2010 | Dille, L. (2010). "A comparison of two curricular models of instruction to increase teacher repertoires for instructing students with autism." Dissertation Abstracts International Section A: Humanities and Social Sciences 70(7-a): 2465. |
| Dinasty 2018 | Dinasty, S. F. H. F. S. R. M. C. (2018). "Allogenic Cord Blood Transfusion in Patients With Autism." https://clinicaltrials.gov/ct2/show/NCT03786744 |
| Divan 2019 | Divan, G., V. Vajaratkar, P. Cardozo, S. Huzurbazar, M. Verma, E. Howarth, R. Emsley, C. Taylor, V. Patel and J. Green (2019). "The Feasibility and Effectiveness of PASS Plus, A Lay Health Worker Delivered Comprehensive Intervention for Autism Spectrum Disorders: Pilot RCT in a Rural Low and Middle Income Country Setting." Autism Research 12(2): 328-339. |
| Doble 2017 | Doble, B., P. E. Langdon, L. Shepstone, G. H. Murphy, D. Fowler, D. Heavens, A. Malovic, A. Russell, A. Rose, L. Mullineaux and E. C. F. Wilson (2017). "Economic Evaluation Alongside a Randomized Controlled Crossover Trial of Modified Group Cognitive-Behavioral Therapy for Anxiety Compared to Treatment-as-Usual in Adults With Asperger Syndrome." MDM Policy and Practice 2(2): 2381468317729353. |
| Doernberg 2020 | Doernberg, E. A., S. W. Russ and A. Dimitropoulos (2020). "Believing in make-believe: Efficacy of a pretend play intervention for school-aged children with high-functioning autism spectrum disorder." Journal of Autism and Developmental Disorders: No Pagination Specified. |
| Domes 2013 | Domes, G., M. Heinrichs, E. Kumbier, A. Grossmann, K. Hauenstein and S. C. Herpertz (2013). "Effects of Intranasal Oxytocin on the Neural Basis of Face Processing in Autism Spectrum Disorder." Biological Psychiatry 74(3): 164-171. |
| Domes 2014 | Domes, G., E. Kumbier, M. Heinrichs and S. C. Herpertz (2014). "Oxytocin promotes facial emotion recognition and amygdala reactivity in adults with asperger syndrome." Neuropsychopharmacology 39(3): 698-706. |
| Drahota 2011 | Drahota, A., J. Wood, K. Sze and M. Dyke (2011). "Effects of cognitive behavioral therapy on daily living skills in children with high-functioning autism and concurrent anxiety disorders." Journal of Autism and Developmental Disorders 41(3): 257-265. |
| Drks 2012 | Drks (2012). "Evaluation of the Freiburg Social Skills Training Program with Focus on Theory of Mind for Children and Adolescents with High-Functioning Autism Spectrum Disorders (TOMTASS)." http://apps.who.int/trialsearch/Trial2.aspx?TrialID=DRKS00004431 |
| DRKS 2015A | DRKS00008984 (2015). A placebo-controlled, double blind, randomised trial with crossover-design investigating the effect of oxytocin nasal spray on neuronal processes of empathy. |
| DRKS 2015B | DRKS00009337 (2015). Zirkus Empathico – Promoting socioemotional competencies in 5- to 10-year-old children with autism spectrum conditions using a computer-based training program. |
| DRKS 2015C | DRKS00008952 (2015). Oxytocin-induced enhancement of Social Skills Training in Adolescents with ASD. |
| DRKS 2016A | DRKS00010053 (2016). Pacebo-controlled, double-blind, randomised phase II study with crossover-design investigating the modulatory effects of intranasal Oxytocin on social cognition in patients with Autism-Spectrum-Disorder. |
| DRKS 2016B | DRKS00011142 (2016). Encoding and decoding of signals for prominence in prosody in subjects with high-functioning autism. |
| DRKS 2018 | DRKS00014732 (2018). Phase-IIa randomized, double-blind, sham-controlled, parallel group trial on anodal tDCS over the left and right tempo-parietal junction in Autism Spectrum Disorder. |
| DRKS 2019A | DRKS00016330 (2019). Multicentre, randomized controlled trial of the Frankfurt Early Intervention Program for tod-dlers and preschool children with Autism Spectrum Disorder – A-FFIP. |
| DRKS 2019B | DRKS00017505 (2019). Modulation of cortical activity with noninvasive neurostimulation in children and adolescents with autism spectrum disorder – A randomized, double-blind and sham-controlled study-. |
| Drks 2019C | Drks (2019). "Emotions on the Road: enhancing Emotion Recognition in Young Children with Autism Spectrum Disorder (ASD) in Hong Kong using The Transporters." http://www.who.int/trialsearch/Trial2.aspx?TrialID=DRKS00016506. |
| Drks 2019D | Drks (2019). "Modulation of cortical activity with noninvasive neurostimulation in children and adolescents with autism spectrum disorder â€“ A randomized, double-blind and sham-controlled study." http://www.who.int/trialsearch/Trial2.aspx?TrialID=DRKS00017505. |
| Drks 2019E | Drks (2019). "Multicentre, randomized controlled trial of the Frankfurt Early Intervention Program for tod-dlers and preschool children with Autism Spectrum Disorder â€“ A-FFIP." http://www.who.int/trialsearch/Trial2.aspx?TrialID=DRKS00016330. |
| DRKS 2020 | DRKS00017817 (2020). Three-arm, cluster-randomized controlled study to assess the effects of FASTER and SCOTT trainings for adults with high-functioning autism spectrum disorder (ASD). |
| Dubicka 2008 | Dubicka, B. (2008). "Adolescents with SSRI-resistant depression: CBT plus antidepressant switch more effective than medication switch alone." Evidence Based Mental Health 11(4): 110-. |
| Dykens 2014 | Dykens, E. M., M. H. Fisher, J. L. Taylor, W. Lambert and N. Miodrag (2014). "Reducing distress in mothers of children with autism and other disabilities: a randomized trial." Pediatrics 134(2): e454-463. |
| Eack 2018 | Eack, S. M., S. S. Hogarty, D. P. Greenwald, M. Y. Litschge, S. A. Porton, C. A. Mazefsky and N. J. Minshew (2018). "Cognitive enhancement therapy for adult autism spectrum disorder: Results of an 18-month randomized clinical trial." Autism Research 11(3): 519-530. |
| Edelson 1999 | Edelson, S. M., M. G. Edelson, D. C. Kerr and T. Grandin (1999). "Behavioral and physiological effects of deep pressure on children with autism: a pilot study evaluating the efficacy of Grandin's Hug Machine." American Journal of Occupational Therapy 53(2): 145-152. |
| Einfeld 2018 | Einfeld, S. L., R. Beaumont, T. Clark, K. S. Clarke, D. Costley, K. M. Gray, S. K. Horstead, M. Redoblado Hodge, J. Roberts, K. Sofronoff, J. R. Taffe and P. Howlin (2018). "School-based social skills training for young people with autism spectrum disorders." Journal of Intellectual and Developmental Disability 43(1): 29-39. |
| Enticott 2014 | Enticott, P. G., B. M. Fitzgibbon, H. A. Kennedy, S. L. Arnold, D. Elliot, A. Peachey, A. Zangen and P. B. Fitzgerald (2014). "A double-blind, randomized trial of deep repetitive transcranial magnetic stimulation (rTMS) for autism spectrum disorder." Brain Stimulation 7(2): 206-211. |
| Enticott 2015 | Enticott, P. G., B. M. Fitzgibbon, M. Kirkovski, A. Green, D. Elliot, A. Zangen and P. B. Fitzgerald (2015). "Deep repetitive transcranial magnetic stimulation (rTMS) to bilateral dorsomedial prefrontal cortex in autism spectrum disorder." Brain stimulation 8(2): 335. |
| Erickson 2011 | Erickson, C. A., K. A. Stigler, L. K. Wink, J. E. Mullett, A. Kohn, D. J. Posey and C. J. McDougle (2011). "A prospective open-label study of aripiprazole in fragile X syndrome." Psychopharmacology 216(1): 85?90. |
| Erickson 2014 | Erickson, C. A., L. K. Wink, M. C. Early, E. Stiegelmeyer, L. Mathieu-Frasier, V. Patrick and C. J. McDougle (2014). "Brief report: Pilot single-blind placebo lead-in study of acamprosate in youth with autistic disorder." Journal of Autism and Developmental Disorders 44(4): 981-987. |
| Ernst 1993 | Ernst, M., L. Devi, R. R. Silva, N. M. Gonzalez, A. M. Small, R. P. Malone and M. Campbell (1993). "Plasma beta-endorphin levels, naltrexone, and haloperidol in autistic children." Psychopharmacology Bulletin 29(2): 221-227. |
| Esse Wilson 2018 | Esse Wilson, J., M. C. Trumbo, J. Wilson and C. D. Tesche (2018). "Transcranial direct current stimulation (tDCS) over right temporoparietal junction (rTPJ) for social cognition and social skills in adults with autism spectrum disorder (ASD)." Journal of Neural Transmission 125(12): 1857-1866. |
| Estes 2014 | Estes, A., L. Vismara, C. Mercado, A. Fitzpatrick, L. Elder, J. Greenson, C. Lord, J. Munson, J. Winter, G. Young, G. Dawson and S. Rogers (2014). "The impact of parent-delivered intervention on parents of very young children with autism." Journal of Autism and Developmental Disorders 44(2): 353-365. |
| EU/EEA 2017 | EU/EEA, E. O. (2017). "Study of Aripiprazole in the Treatment of Serious Behavioral Problems in Children and Adolescents With Autistic Disorder (AD)." http://www.who.int/trialsearch/Trial2.aspx?TrialID=EUCTR2016-005111-40-Outside-EU/EEA. |
| Euctr 2006 | Euctr, N. L. (2006). "A Randomized, Double-Blind, Placebo-Controlled Maintenance of Effect Study of Olanzapine in the Treatment of Disruptive Behavioral Symptoms in Children and Adolescents with Pervasive Developmental Disorders - HGMR." http://www.who.int/trialsearch/Trial2.aspx?TrialID=EUCTR2006-005346-37-NL. |
| Euctr 2007 | Euctr FR. Emotional and Social deficits in Asperger syndrome - Asperger and Oxytocin. Http://wwwwhoint/trialsearch/trial2aspx? Trialid=euctr2006-006126-25-fr 2007. |
| EUCTR 2013 | EUCTR2012-005742-38-GB (2013). Early phase triple blind placebo controlled RCT of simvastatin treatment for autism in young children with Neurofibromatosis Type 1. |
| Euctr 2018A | Euctr BE. The use of Oxytocin for Autism Spectrum Disorders: investigating the effect on behavior and at the level of the brain. Http://wwwwhoint/trialsearch/trial2aspx? Trialid=euctr2018-000769-35-be 2018. |
| EUCTR 2018B | EUCTR2017-004419-38-FR (2018). Efficacy and safety of bumetanide oral liquid formulation in children and adolescents aged from 7 to less than 18 years old with Autism Spectrum Disorder. |
| Euctr 2018C | Euctr, P. T. (2018). "Efficacy and safety of bumetanide oral liquid formulation in children aged from 2 to less than 7 years old with Autism Spectrum Disorder." http://www.who.int/trialsearch/Trial2.aspx?TrialID=EUCTR2017-004420-30-PT. |
| EUCTR 2018D | EUCTR2017-004419-38-GB (2018). Efficacy and safety of bumetanide oral liquid formulation in children and adolescents aged from 7 to less than 18 years old with Autism Spectrum Disorder. |
| Euctr 2018E | Euctr ES. A Study to Evaluate Efficacy, and Safety of Balovaptan in Adults with Autism Spectrum Disorder with a 2 Year Open-Label Extension. Http://wwwwhoint/trialsearch/trial2aspx? Trialid=euctr2017-004378-32-es 2018. |
| Euctr 2019 | Euctr, D. E. (2018). "Efficacy and safety of bumetanide oral liquid formulation in children and adolescents aged from 7 to less than 18 years old with Autism Spectrum Disorder." http://www.who.int/trialsearch/Trial2.aspx?TrialID=EUCTR2017-004419-38-DE. |
| Euctr-000106-11-Fr 2016 | Euctr-000106-11-Fr (2016). "Evaluation of the efficiency of treatment by bumetanide on autistic children with a known etiology: Multicenter and double-blind study with randomized parallel group, against placebo.". https://www.clinicaltrialsregister.eu/ctr-search/trial/2016-000106-11/FR |
| Euctr-000586-45-Be 2014 | Euctr-000586-45-Be (2014). "The use of Oxytocin for Autism Spectrum Disorders: Investigating the effect on behavior and at the level of the brain." https://www.clinicaltrialsregister.eu/ctr-search/trial/2014-000586-45/BE |
| Euctr-000955-25-Fr 2015 | Euctr-000955-25-Fr (2015). "Evaluation of the efficiency of B9 vitamin on the reduction of autistic spectrum symptoms:a pilot study EFFET." https://www.clinicaltrialsregister.eu/ctr-search/trial/2015-000955-25/FR |
| Euctr-001220-31-Outside-Eu/Eea 2015 | Euctr-001220-31-Outside-Eu/Eea (2015). "A Study of the Effectiveness and Safety of Two Doses of Risperidone in the Treatment of Children and Adolescents With Autistic Disorder." https://www.clinicaltrialsregister.eu/ctr-search/trial/2015-001220-31/3rd |
| Euctr-001230-17-Fr 2015 | Euctr-001230-17-Fr (2015). "La melatonine restaure-t-elle l’architecture du sommeil chez les enfants avec autisme ? Etude de phase II." https://www.clinicaltrialsregister.eu/ctr-search/trial/2013-001230-17/FR |
| Euctr-001320-31-Outside-Eu/Eea 2015 | Euctr-001320-31-Outside-Eu/Eea (2015). "A Study to Evaluate the Efficacy and Safety of Risperidone (R064766) in Children and Adolescents with Irritability Associated with Autistic Disorder." https://www.clinicaltrialsregister.eu/ctr-search/trial/2015-001320-31/3rd |
| Euctr-001560-35-Nl 2016 | Euctr-001560-35-Nl (2016). "Bumetanide for Autism Treatment Study." https://www.clinicaltrialsregister.eu/ctr-search/trial/2014-001560-35/NL |
| Euctr-001689-97-Fr 2009 | Euctr-001689-97-Fr (2009). "Etude de la relation dose-effet de la melatonine dans l'autisme infantile. - MELADOSE." https://www.clinicaltrialsregister.eu/ctr-search/trial/2008-001689-97/FR |
| Euctr-003259-39-Es 2014 | Euctr-003259-39-Es (2014). "Study in children and adolescents with autism." https://www.clinicaltrialsregister.eu/ctr-search/trial/2013-003259-39/ES |
| Euctr-003712-36-Fr 2008 | Euctr-003712-36-Fr (2008). "Etude de la reponse clinique et neurofonctionnelle a la fluoxetine dans l'autisme infantile - FAIR." https://www.clinicaltrialsregister.eu/ctr-search/trial/2008-003712-36/FR |
| Euctr-003750-89-De 2012 | Euctr-003750-89-De (2012). "Empathy, Autism and Oxytocin – an investigation by means of functional magnetic resonance imaging and moleculargenetic analyses." https://www.clinicaltrialsregister.eu/ctr-search/trial/2012-003750-89/DE |
| Euctr-004419-38-De 2018 | Euctr-004419-38-De. Efficacy and safety of bumetanide oral liquid formulation in children and adolescents aged from 7 to less than 18 years old with Autism Spectrum Disorder. 2018. |
| Euctr-004420-30-Es 2018 | Euctr-004420-30-Es. Efficacy and safety of bumetanide oral liquid formulation in children aged from 2 to less than 7 years old with Autism Spectrum Disorder. 2018. |
| Euctr-006126-25-Fr 2007 | Euctr-006126-25-Fr. Emotional and Social deficits in Asperger syndrome - Asperger and Oxytocin. 2007. |
| Euctr-006444-21-Es 2010 | Euctr-006444-21-Es. EFFECT OF 8-WEEK FATTY ACIDS OMEGA-3 TREATMENT ON OXIDATIVE METABOLISM IN PATIENTS WITH AUTISM SPECTRUM DISORDER: A RANDOMISED DOUBLE-BLIND CROSSOVER PLACEBO-CONTROLLED TRIAL. - Omega-3 tr. 2010. |
| Euctr-009475-35-Nl 2009 | Euctr-009475-35-Nl. Lack of Empathy as a Symptom in various Psychiatric Disorders - Psychopathology and the Lack of Empathy. 2009. |
| Euctr-010393-38-Fr 2009 | Euctr-010393-38-Fr. Etude de l’efficacite d’un traitement par bumetanide dans une population d’enfants autistes. 2009. |
| Euctr-012102-39-It 2009 | Euctr-012102-39-It. ?Clinical studies on the effectiveness of the gluten-free diet and casein and therapy anti-inflammatory bowel change in psychiatric symptoms intestinal and in patients with childhood autismo? - nd. 2009. |
| Euctr-018740-13-Nl 2010 | Euctr-018740-13-Nl. Short- and long-term effects of oxytocin on empathy and social behaviour in autistic and antisocial male adults. - Oxytocin effects in autistic and antisocial male adults. 2010. |
| Euctr-022511-18-De 2010 | Euctr-022511-18-De. Behavioral effects and neural correlates of oxytocin on social attention [Verhaltenseffekte und neuronales Korrelat von Oxytocin im Kontext sozialer Aufmerksamkeit]. 2010. |
| Euctr-024202-34-De 2013 | Euctr-024202-34-De. group-therapy, autism and oxytocin - an investigation with the question Does oxytocin (OT) enhance therapy effects in autism?“. 2013. |
| Eugene Arnold 2019 | Eugene Arnold, L., R. A. Luna, K. Williams, J. Chan, R. A. Parker, Q. Wu, J. A. Hollway, A. Jeffs, F. Lu, D. L. Coury, C. Hayes and T. Savidge (2019). "Probiotics for Gastrointestinal Symptoms and Quality of Life in Autism: A Placebo-Controlled Pilot Trial." Journal of Child and Adolescent Psychopharmacology 29(9): 659-669. |
| Factor 2019 | Factor, R. S., D. M. Swain, L. Antezana, A. Muskett, A. J. Gatto, S. R. Radtke and A. Scarpa (2019). "Teaching emotion regulation to children with autism spectrum disorder: Outcomes of the Stress and Anger Management Program (STAMP)." Bulletin of the Menninger Clinic 83(3): 235-258. |
| Falissard 2019 | Falissard, B., C. A. Severo, E. Lambert, V. Crutel, S. Kyaga, S. Serret, D. Ravel and E. Lemonnier (2019). "P.809 Correlation between childhood autism rating scale 2 and clinical global impression improvement." European Neuropsychopharmacology 29 (Supplement 6): S538-S539. |
| Fang 2018 | Fang L, Jiang X, Huang Y et al. Efficacy of Vitamin D combined with omega-3 fatty acid in treatment of children with autism spectrum disorder. Pharmaceutical Care and Research 2018; 18: 347-350 and 363. |
| Feinberg 2014 | Feinberg E, Augustyn M, Fitzgerald E et al. Improving maternal mental health after a child's diagnosis of autism spectrum disorder: results from a randomized clinical trial. JAMA Pediatrics 2014; 168: 40-46. |
| Feldman 1999 | Feldman HM, Kolmen BK, Gonzaga AM. Naltrexone and communication skills in young children with autism. Journal of the American Academy of Child and Adolescent Psychiatry 1999; 38: 587-593. |
| Felzer-Kim 2020 | Felzer-Kim, I. T. and J. L. Hauck (2020). "How Much Instructional Time Is Necessary? Mid-intervention Results of Fundamental Movement Skills Training Within ABA Early Intervention Centers." Frontiers in Integrative Neuroscience 14 (no pagination). |
| Field 1997 | Field T, Lasko D, Mundy P et al. Brief report: Autistic children's attentiveness and responsivity improve after touch therapy. Journal of Autism and Developmental Disorders 1997; 27: 333-338. |
| Findling 2014 | Findling RL, Mankoski R, Timko K et al. A randomized controlled trial investigating the safety and efficacy of aripiprazole in the long-term maintenance treatment of pediatric patients with irritability associated with autistic disorder. Journal of Clinical Psychiatry 2014; 75: 22-30. |
| Fisher 2005 | Fisher N, Happe F. A training study of theory of mind and executive function in children with autistic spectrum disorders. Journal of Autism and Developmental Disorders 2005; 35: 757-771. |
| Fisher 2020 | Fisher, W. W., K. C. Luczynski, A. P. Blowers, M. E. Vosters, M. D. Pisman, A. R. Craig, S. A. Hood, M. A. Machado, A. D. Lesser and C. C. Piazza (2020). "A randomized clinical trial of a virtual-training program for teaching applied-behavior- analysis skills to parents of children with autism spectrum disorder." Journal of applied behavior analysis. 28. |
| Floreo 2018 | Floreo, I. (2018). VR for Joint Attention Support in ASD, https://ClinicalTrials.gov/show/NCT04016701. |
| Florida 2009 | Florida U, Health E, Development H et al. Cognitive Behavioral Therapy (CBT) for Anxiety Disorders in Autism: Adapting Treatment for Adolescents. 2009. |
| Florida 2010 | Florida U. Cognitive-Behavioral Treatment for Anxiety Disorders in Children With Autism Spectrum Disorders. 2010. |
| Florida 2014 | Florida U, University T, University of California LA. Psychotherapy for Anxiety in Children With Autism Spectrum Disorder. 2014. |
| Foundation 2004 | Foundation N. An Autism Study Using Nambudripad's Food Allergy Elimination Treatments. 2004. |
| Fox 2018 | Fox SA. An early start denver model-based group intervention for parents of very young children diagnosed with or at risk for autism spectrum disorder. Dissertation Abstracts International: Section B: The Sciences and Engineering 2018; 79: no pagination specified. |
| Frank 2020 | Frank, H. E., E. R. Kagan, E. A. Storch, J. J. Wood, C. M. Kerns, A. B. Lewin, B. J. Small and P. C. Kendall (2020). "Accommodation of Anxiety in Youth with Autism Spectrum Disorder: Results from the TAASD Study." Journal of Clinical Child & Adolescent Psychology: 1-11. |
| Frankel 2010A | Frankel F, Myatt R, Sugar C et al. A randomized controlled study of parent-assisted children's friendship training with children having autism spectrum disorders. Journal of Autism and Developmental Disorders 2010; 40: 827-842. |
| Frankel 2010B | Frankel F, Myatt R, Sugar C et al. Erratum: a randomized controlled study of parent-assisted children's friendship training with children having autism spectrum disorders (Journal of Autism and Developmental Disorders DOI: 10.1007/s10803-009-0932-z). Journal of Autism and Developmental Disorders 2010; 40: 843. |
| Frazier 2017 | Frazier TW, Krishna J, Klingemier E et al. A randomized, crossover trial of a novel sound-to-sleep mattress technology in children with autism and sleep difficulties. Journal of Clinical Sleep Medicine 2017; 13: 95-104. |
| Freitag 2013 | Freitag CM, Cholemkery H, Elsuni L et al. The group-based social skills training SOSTA-FRA in children and adolescents with high functioning autism spectrum disorder--study protocol of the randomised, multi-centre controlled SOSTA--net trial. Trials 2013; 14: 6. |
| Freitag 2015 | Freitag C. Autism specific group therapy: Randomized controlled trial. European Child and Adolescent Psychiatry 2015; 24: s46. |
| Freitag 2016 | Freitag CM, Jensen K, Elsuni L et al. Group-based cognitive behavioural psychotherapy for children and adolescents with ASD: the randomized, multicentre, controlled SOSTA-net trial. Journal of child psychology and psychiatry, and allied disciplines" 2016; 57: 596-605. |
| Fridenson-Hayo 2017A | Fridenson-Hayo S, Berggren S, Lassalle A et al. 'Emotiplay': a serious game for learning about emotions in children with autism: results of a cross-cultural evaluation. European Child and Adolescent Psychiatry 2017; 26: 979-992. |
| Fridenson-Hayo 2017B | Fridenson-Hayo S, Berggren S, Lassalle A et al. 'Emotiplay': a serious game for learning about emotions in children with autism: results of a cross-cultural evaluation. European Child and Adolescent Psychiatry 2017; 1?14. |
| Frolli 2020 | Frolli, A., M. C. Ricci, F. A. Tortorelli, A. Cavallaro, L. Valenzano, A. Rega, F. F. Operto and G. Corrivetti (2020). "Emotional Education in Early Onset Schizophrenia and Asperger's Syndrome." Behavioral sciences 10(9): 29. |
| Frye 2018 | Frye RE, Slattery J, Delhey L et al. Folinic acid improves verbal communication in children with autism and language impairment: a randomized double-blind placebo-controlled trial. Molecular Psychiatry 2018; 23: 247-256. |
| Fujii 2013 | Fujii C, Renno P, McLeod Bryce D et al. Intensive cognitive behavioral therapy for anxiety disorders in school-aged children with autism: a preliminary comparison with treatment-as-usual. School mental health 2013; 5: 25?37. |
| Fung 2014 | Fung SC, Leung ASM. Pilot Study Investigating the Role of Therapy Dogs in Facilitating Social Interaction among Children with Autism. Journal of Contemporary Psychotherapy 2014; 44: 253-262. |
| Furukawa 2018 | Furukawa K, Okuno H, Mohri I et al. Effectiveness of child-directed interaction training for young Japanese children with autism spectrum disorders. Child and Family Behavior Therapy 2018; 40: 166-186. |
| Gabis 2019 | Gabis, L. V., R. Ben-Hur, S. Shefer, A. Jokel and D. B. Shalom (2019). "Improvement of Language in Children with Autism with Combined Donepezil and Choline Treatment." Journal of Molecular Neuroscience 69(2): 224-234. |
| Gabriels 2015 | Gabriels RL, Pan Z, Dechant B et al. Randomized Controlled Trial of Therapeutic Horseback Riding in Children and Adolescents With Autism Spectrum Disorder. Journal of the American Academy of Child and Adolescent Psychiatry 2015; 54: 541-549. |
| Gabriels 2018 | Gabriels RL, Pan ZX, Guerin NA et al. Long-Term Effect of Therapeutic Horseback Riding in Youth With Autism Spectrum Disorder: A Randomized Trial. Frontiers in Veterinary Science 2018; 5. |
| Gantman 2012 | Gantman A, Kapp SK, Orenski K, Laugeson EA. Social skills training for young adults with high-functioning autism spectrum disorders: A randomized controlled pilot study. Journal of Autism and Developmental Disorders 2012; 42: 1094-1103. |
| Gao 2020 | Gao, D., T. Yu, C. L. Li, F. Y. Jia and H. H. Li (2020). "[Effect of parental training based on Early Start Denver Model combined with intensive training on children with autism spectrum disorder and its impact on parenting stress]." Zhongguo Dangdai Erke Zazhi 22(2): 158-163. |
| Garcia-Villamisar 2011 | Garcia-Villamisar D, Dattilo J. Social and clinical effects of a leisure program on adults with autism spectrum disorder. Research in Autism Spectrum Disorders 2011; 5: 246-253. |
| Garcia-Villamisar 2017 | Garcia-Villamisar D, Dattilo J, Muela C. Effects of B-Active2 on Balance, Gait, Stress, and Well-Being of Adults With Autism Spectrum Disorders and Intellectual Disability: A Controlled Trial. Adapted Physical Activity Quarterly 2017; 34: 125-140. |
| Gengoux 2019 | Gengoux, G. W., D. A. Abrams, R. Schuck, M. E. Millan, R. Libove, C. M. Ardel, J. M. Phillips, M. Fox, T. W. Frazier and A. Y. Hardan (2019). "A pivotal response treatment package for children with autism spectrum disorder: An RCT." Pediatrics 144(3). |
| Geretsegger 2012 | Geretsegger M, Holck U, Gold C. Randomised controlled Trial of Improvisational Music therapy's Effectiveness for children with Autism spectrum disorders (TIME-A): Study protocol. BMC Pediatrics 2012; 2. |
| Geretsegger 2016 | Geretsegger M, Holck U, Bieleninik L, Gold C. Feasibility of a Trial on Improvisational Music Therapy for Children with Autism Spectrum Disorder. Journal of Music Therapy 2016; 53: 93-120. |
| Germone 2019 | Germone, M. M., R. L. Gabriels, N. A. Guérin, Z. Pan, T. Banks and M. E. O'Haire (2019). "Animal-assisted activity improves social behaviors in psychiatrically hospitalized youth with autism." Autism 23(7): 1740‐1751. |
| Gev 2017 | Gev T, Rosenan R, Golan O. Unique effects of The transporters animated series and of parental support on emotion recognition skills of children with ASD: Results of a randomized controlled trial. Autism research : Official Journal of the International Society for Autism Research 2017; 10: 993-1003. |
| Ghaleiha 2013 | Ghaleiha A, Mohammadi E, Mohammadi MR et al. Riluzole as an adjunctive therapy to risperidone for the treatment of irritability in children with autistic disorder: A double-blind, placebo-controlled, randomized trial. Pediatric Drugs 2013; 15: 505-514. |
| Ghaleiha 2014 | Ghaleiha A, Ghyasvand M, Mohammadi MR et al. Galantamine efficacy and tolerability as an augmentative therapy in autistic children: A randomized, double-blind, placebo-controlled trial. Journal of Psychopharmacology 2014; 28: 677-685. |
| Ghalichi 2016 | Ghalichi F, Ghaemmaghami J, Malek A, Ostadrahimi A. Effect of gluten free diet on gastrointestinal and behavioral indices for children with autism spectrum disorders: a randomized clinical trial. World Journal of Pediatrics 2016; 12: 436-442. |
| Ghanizadeh 2013 | Ghanizadeh A, Moghimi-Sarani E. A randomized double blind placebo controlled clinical trial of N-Acetylcysteine added to risperidone for treating autistic disorders. BMC Psychiatry 2013; 13. |
| Ghaziuddin 1991 | Ghaziuddin M, Tsai L, Ghaziuddin N. Fluoxetine in autism with depression. Journal of the American Academy of Child and Adolescent Psychiatry 1991; 30: 508-509. |
| Ghodsi 2019 | Ghodsi R, Kheirouri S, Nosrati R. Carnosine supplementation does not affect serum concentrations of advanced glycation and precursors of lipoxidation end products in autism: a randomized controlled clinical trial. Annals of Clinical Biochemistry 2019; 56: 148-154. |
| Giarelli 2005 | Giarelli E, Souders M, Pinto-Martin J et al. Intervention pilot for parents of children with autistic spectrum disorder. Pediatric Nursing 2005; 31: 389-399. |
| Ginn 2017 | Ginn NC, Clionsky LN, Eyberg SM et al. Child-Directed Interaction Training for Young Children With Autism Spectrum Disorders: Parent and Child Outcomes. Journal of Clinical Child and Adolescent Psychology 2017; 46: 101-109. |
| Glod 2013 | Glod M. Teaching emotion recognition skills to young children with autism: a randomised controlled trial of an emotion training programme. Child: Care, Health and Development" 2013; 39: 613-. |
| Goeb 2009A | Goeb JL, Ravary M, Lallie C et al. Packing therapy is efficient in serious behavioral problems in children and adolescents with autism. Neuropsychiatrie de l'Enfance et de l'Adolescence 2009; 57: 529-534. |
| Goeb 2009B | Goeb, J. L., M. Ravary, C. Lallie, G. Kechid, R. Jardri, F. Bonelli, A. Y. Lenfant, J. M. Baleyte, C. Mille and P. Delion (2009). "Packing therapy is efficient in serious behavioral problems in children and adolescents with autism." Neuropsychiatrie de l'enfance ET de l'adolescence 57(6): 529‐534. |
| Golan 2010 | Golan O, Ashwin E, Granader Y et al. Enhancing emotion recognition in children with autism spectrum conditions: an intervention using animated vehicles with real emotional faces. Journal of Autism and Developmental Disorders 2010; 40: 269?279. |
| Gonzalez-Domenech 2020 | González-Domenech, P. J., F. Díaz Atienza, C. García Pablos, M. L. Fernández Soto, J. M. Martínez-Ortega and L. Gutiérrez-Rojas (2020). "Influence of a Combined Gluten-Free and Casein-Free Diet on Behavior Disorders in Children and Adolescents Diagnosed with Autism Spectrum Disorder: a 12-Month Follow-Up Clinical Trial." Journal of autism and developmental disorders 50(3): 935‐948. |
| Goodday 2014 | Goodday A, Corkum P, Smith IM. Parental acceptance of treatments for insomnia in children with attention-deficit/hyperactivity disorder, autistic spectrum disorder, and their typically developing peers. Children's Health Care 2014; 43: 54-71. |
| Goodman 2018 | Anonymous (2018). "A neurovisceral approach to autism: targeting self-regulation and core symptoms using neurofeedback and biofeedback." Neuroregulation 5(1): 9?29. |
| Goods 2013 | Goods KS, Ishijima E, Chang YC, Kasari C. Preschool based JASPER intervention in minimally verbal children with Autism: Pilot RCT. Journal of Autism and Developmental Disorders 2013; 43: 1050-1056. |
| Gordon 1992 | Gordon CT, Rapoport JL, Hamburger SD et al. Differential response of seven subjects with autistic disorder to clomipramine and desipramine. American Journal of Psychiatry 1992; 149: 363-366. |
| Gordon 1993 | Gordon CT, State RC, Nelson JE et al. A double-blind comparison of clomipramine, desipramine, and placebo in the treatment of autistic disorder. Archives of General Psychiatry 1993; 50: 441-447. |
| Gordon 2011 | Gordon K, Pasco G, McElduff F et al. A communication-based intervention for nonverbal children with autism: What changes? who benefits? Journal of Consulting and Clinical Psychology 2011; 79: 447-457. |
| Gordon 2015 | Gordon K, Murin M, Baykaner O et al. A randomised controlled trial of PEGASUS, a psychoeducational programme for young people with high-functioning autism spectrum disorder. Journal of child psychology and psychiatry, and allied disciplines" 2015; 56: 468-476. |
| Gordon 2016 | Gordon I, Jack A, Pretzsch CM et al. Intranasal Oxytocin Enhances Connectivity in the Neural Circuitry Supporting Social Motivation and Social Perception in Children with Autism. Scientific Reports 2016; 6: 35054. |
| Grahame 2014 | Grahame V, Dixon L, McConachie H et al. Managing repetitive behaviours in young children with autism spectrum disorder (ASD): New parent group intervention. Developmental Medicine and Child Neurology 2014; 4): 53-54. |
| Grahame 2015 | Grahame V, Brett D, Dixon L et al. Managing Repetitive Behaviours in Young Children with Autism Spectrum Disorder (ASD): Pilot Randomised Controlled Trial of a New Parent Group Intervention. Journal of Autism and Developmental Disorders 2015; 45: 3168-3182. |
| Grandgeorge 2012 | Grandgeorge M, Tordjman S, Lazartigues A et al. Does pet arrival trigger prosocial behaviors in individuals with autism? PloS One 2012; 7. |
| Gringras 2014 | Gringras P, Green D, Wright B et al. Weighted blankets and sleep in autistic children - A randomized controlled trial. Pediatrics 2014; 134: 298-306. |
| Gringras 2017 | Gringras P, Nir T, Breddy J et al. Efficacy and Safety of Pediatric Prolonged-Release Melatonin for Insomnia in Children With Autism Spectrum Disorder. Journal of the American Academy of Child and Adolescent Psychiatry 2017; 56: 948?957.e944. |
| Grob 2014 | Grob CS. Placebo-controlled, Randomized, Blinded, Dose Finding Phase 2 Pilot Safety Study of MDMA-assisted Therapy for Social Anxiety in Autistic Adults. Http://clinicaltrialsgov/show/nct02008396 2014. |
| Guastella 2010 | Guastella AJ, Einfeld SL, Gray KM et al. Intranasal oxytocin improves emotion recognition for youth with autism spectrum disorders. Biological Psychiatry 2010; 67: 692-694. |
| Guastella 2012 | Guastella AJ. A randomized controlled trial of oxytocin nasal spray to treat youth diagnosed with autism spectrum disorders. Biological Psychiatry 2012; 71: 234s. |
| Guastella 2015 | Guastella AJ, Gray KM, Rinehart NJ et al. The effects of a course of intranasal oxytocin on social behaviors in youth diagnosed with autism spectrum disorders: A randomized controlled trial. Journal of Child Psychology and Psychiatry 2015; 56: 444-452. |
| Gulsrud 2007 | Gulsrud AC, Kasari C, Freeman S, Paparella T. Children with autism's response to novel stimuli while participating in interventions targeting joint attention or symbolic play skills. Autism 2007; 11: 535-546. |
| Gulsrud 2014 | Gulsrud AC, Hellemann GS, Freeman SF, Kasari C. Two to ten years: developmental trajectories of joint attention in children with ASD who received targeted social communication interventions. Autism Research 2014; 7: 207?215. |
| Haakonsen Smith 2018 | Haakonsen Smith C, Turbitt E, Muschelli J et al. Feasibility of Coping Effectiveness Training for Caregivers of Children with Autism Spectrum Disorder: a Genetic Counseling Intervention. Journal of Genetic Counseling 2018; 27: 252-262. |
| Hadjikhani 2018 | Hadjikhani N, Asberg Johnels J, Lassalle A et al. Bumetanide for autism: more eye contact, less amygdala activation. Scientific Reports 2018; 8: 3602. |
| Hadwin 1996 | Hadwin J, Baron-Cohen S, Howlin P, Hill K. Can we teach children with autism to understand emotions, belief or pretence? Development and Psychopathology 1996; 8: 345?365. |
| Hagerman 2018 | Hagerman R, Potter L, Biag H et al. A controlled trial of sertraline in children 2 to 6 with ASD without fragile X syndrome. Journal of Intellectual Disability Research 2018; 62: 667-668. |
| Hagner 2012 | Hagner D, Kurtz A, Cloutier H et al. Outcomes of a family-centered transition process for students with autism spectrum disorders. Focus on Autism and Other Developmental Disabilities 2012; 27: 42-50. |
| Hajiabolhasani-Nargani 2016 | Hajiabolhasani-Nargani Z, Najafi M, Mehrabi T. Effect of mobile parenting skills education on anxiety of the mothers with autistic children. Iranian Journal of Nursing and Midwifery Research 2016; 21: 572-576. |
| Hajizadeh-Zaker 2018 | Hajizadeh-Zaker R, Ghajar A, Mesgarpour B et al. l-Carnosine As an Adjunctive Therapy to Risperidone in Children with Autistic Disorder: A Randomized, Double-Blind, Placebo-Controlled Trial. Journal of Child and Adolescent Psychopharmacology 2018; 28: 74-81. |
| Halas 2016 | Halas FP, Llc YP. Safety of L1-79 in Autism. 2016. - NCT02947048 |
| Hallett 2020 | Hallett, V., J. Mueller, L. Breese, M. Hollett, B. Beresford, A. Irvine, A. Pickles, V. Slonims, S. Scott, T. Charman and E. Simonoff (2020). "Introducing 'Predictive Parenting': A Feasibility Study of a New Group Parenting Intervention Targeting Emotional and Behavioral Difficulties in Children with Autism Spectrum Disorder." Journal of Autism & Developmental Disorders 15: 15. |
| Handen 2009 | Handen BL, Melmed RD, Hansen RL et al. A double-blind, placebo-controlled trial of oral human immunoglobulin for gastrointestinal dysfunction in children with autistic disorder. Journal of Autism and Developmental Disorders 2009; 39: 796-805. |
| Handen 2011 | Handen BL, Johnson CR, McAuliffe-Bellin S et al. Safety and efficacy of donepezil in children and adolescents with autism: Neuropsychological measures. Journal of Child and Adolescent Psychopharmacology 2011; 21: 43-50. |
| Handen 2017 | Handen BL, Anagnostou E, Aman MG et al. A Randomized, Placebo-Controlled Trial of Metformin for the Treatment of Overweight Induced by Antipsychotic Medication in Young People With Autism Spectrum Disorder: Open-Label Extension. Journal of the American Academy of Child and Adolescent Psychiatry 2017; 56: 849-856.e846. |
| Hannant 2019 | Hannant, P., S. Cassidy, D. Renshaw and A. Joyce (2019). "A double-blind, placebo-controlled, randomised-designed GABA tea study in children diagnosed with autism spectrum conditions: a feasibility study clinical trial registration: ISRCTN 72571312." Nutritional Neuroscience: 1-17. |
| Hardan 2012 | Hardan AY, Fung LK, Libove RA et al. A Randomized Controlled Pilot Trial of Oral N-Acetylcysteine in Children with Autism. Biological Psychiatry 2012; 71: 956-961. |
| Hardan 2015 | Hardan AY, Gengoux GW, Berquist KL et al. A randomized controlled trial of Pivotal Response Treatment Group for parents of children with autism. Journal of child psychology and psychiatry, and allied disciplines" 2015; 56: 884-892. |
| Hardan 2016 | Hardan A. Psychopharmacological treatment of anxiety symptoms in autism spectrum disorder. Journal of the American Academy of Child and Adolescent Psychiatry 2016; 55: s325-s326. |
| Hardan 2019 | Hardan, A. Y., R. L. Hendren, M. G. Aman, A. Robb, R. D. Melmed, K. A. Andersen, R. Luchini, R. Rahman, S. Ali, X. D. Jia, M. Mallick, J. E. Lateiner, R. H. Palmer and S. M. Graham (2019). "Efficacy and safety of memantine in children with autism spectrum disorder: Results from three phase 2 multicenter studies." Autism 23(8): 2096-2111. |
| Harfterkamp 2013 | Harfterkamp M, Van Der Meer J. A Randomized double-blind study of atomoxetine vs. placebo followed by an open label extension period of treatment with atomoxetine for ADHD symptoms in children with ASD. European Child and Adolescent Psychiatry 2013; 22: s216-s217. |
| Harfterkamp 2014 | Harfterkamp M, Buitelaar JK, Minderaa RB et al. Atomoxetine in Autism Spectrum Disorder: No Effects on Social Functioning; Some Beneficial Effects on Stereotyped Behaviors, Inappropriate Speech, and Fear of Change. Journal of Child and Adolescent Psychopharmacology 2014; 24: 481-485. |
| Hawkins 2019 | Hawkins, J. R., N. Weatherby, B. Wrye and K. Ujcich Ward (2019). "Bergamot Aromatherapy for Medical Office-Induced Anxiety Among Children With an Autism Spectrum Disorder: A Randomized, Controlled, Blinded Clinical Trial." Holistic Nursing Practice 33(5): 285-294. |
| Health 1997 | Health N. Treatment of Autism in Children and Adolescents. 1997. - NCT00005014 |
| Health 1999 | Health E, Development H, Deafness N, Disorders OC. Secretin for the Treatment of Autism. 1999. - NCT00065962 |
| Health 2001 | Health E, Development H, Health N. Improving Attention Skills of Children With Autism. 2001. |
| Health 2006A | Health N, Center N. Minocycline to Treat Childhood Regressive Autism. 2006. |
| Health 2006B | Health N, Center N. Mercury Chelation to Treat Autism. 2006. - NCT00376194 |
| Health 2010 | Health N, Center N. A Study of Divalproex Sodium in Children With ASD and Epileptiform EEG. 2010. - NCT01170325 |
| Health 2012A | Health S. Nuedexta for the Treatment of Adults With Autism. 2012. - NCT01630811 |
| Health 2012B | Health S. Autologous Cord Blood Stem Cells for Autism. 2012. |
| Health 2013 | Health N, Center N. A Trial of the Drug Donepezil for Sleep Enhancement and Behavioral Change in Children With Autism. 2013. - NCT01887132 |
| Hegarty 2017 | Hegarty JP, Ferguson BJ, Zamzow RM et al. Beta-adrenergic antagonism modulates functional connectivity in the default mode network of individuals with and without autism spectrum disorder. Brain Imaging and Behavior 2017; 11: 1278-1289. |
| Hellings 2001 | Hellings, J. A., J. R. Zarcone, K. Crandall, D. Wallace and S. R. Schroeder (2001). "Weight gain in a controlled study of risperidone in children, adolescents and adults with mental retardation and autism." Journal of Child & Adolescent Psychopharmacology 11(3): 229-238. |
| Hellings 2005 | Hellings, J. A., J. R. Zarcone, M. G. Valdovinos, R. Reese, E. Gaughan and S. R. Schroeder (2005). "Risperidone-induced prolactin elevation in a prospective study of children, adolescents, and adults with mental retardation and pervasive developmental disorders." Journal of Child and Adolescent Psychopharmacology 15(6): 885-892. |
| Hemdi 2017 | Hemdi A, Daley D. The Effectiveness of a Psychoeducation Intervention delivered via WhatsApp for mothers of children with Autism Spectrum Disorder (ASD) in the Kingdom of Saudi Arabia: A randomized controlled trial. Child: Care, Health and Development" 2017; 43: 933-941. |
| Hendouei 2020 | Hendouei, F., H. S. Moghaddam, M. R. Mohammadi, N. Taslimi, F. Rezaei and S. Akhondzadeh (2020). "Resveratrol as adjunctive therapy in treatment of irritability in children with autism: A double-blind and placebo-controlled randomized trial." Journal of Clinical Pharmacy and Therapeutics 45(2): 324-334. |
| Hendren 2016 | Hendren RL, James S, Widjaja F et al. Randomized, placebo-controlled trial of methyl B12 for children with autism. Journal of Child and Adolescent Psychopharmacology 2016; 26: 774-783. |
| Hepburn 2016 | Hepburn SL, Blakeley-Smith A, Wolff B, Reaven JA. Telehealth delivery of cognitive-behavioral intervention to youth with autism spectrum disorder and anxiety: A pilot study. Autism 2016; 20: 207-218. |
| Herscu 2020 | Herscu, P., B. L. Handen, L. E. Arnold, M. F. Snape, J. D. Bregman, L. Ginsberg, R. Hendren, A. Kolevzon, R. Melmed, M. Mintz, N. Minshew, L. Sikich, A. Attalla, B. King, T. Owley, A. Childress, H. Chugani, J. Frazier, C. Cartwright and T. Murphy (2020). "The SOFIA Study: Negative Multi-center Study of Low Dose Fluoxetine on Repetitive Behaviors in Children and Adolescents with Autistic Disorder." Journal of Autism and Developmental Disorders 50(9): 3233-3244. |
| Higashida 2019 | Higashida H, Munesue T, Kosaka H et al. Social Interaction Improved by Oxytocin in the Subclass of Autism with Comorbid Intellectual Disabilities. Diseases 2019; 7: 22. |
| Ho 2020 | Ho, M. H. and L. Y. Lin (2020). "Efficacy of parent-training programs for preschool children with autism spectrum disorder: A randomized controlled trial." Research in Autism Spectrum Disorders 71 (no pagination). |
| Ho Yan 2018 | Ho Yan L. Evaluation of a home-based, transdisciplinary intervention for autism spectrum disorder in Hong Kong. Dissertation Abstracts International: Section B: The Sciences and Engineering 2018; 79: no pagination specified. |
| Hochhauser 2018 | Hochhauser, M., P. L. Weiss and E. Gal (2018). "Enhancing conflict negotiation strategies of adolescents with autism spectrum disorder using video modeling." Assistive technology : the official journal of RESNA 30(3): 107-118. |
| Hodgetts 2011 | Hodgetts S, Magill-Evans J, Misiaszek JE. Weighted vests, stereotyped behaviors and arousal in children with autism. Journal of Autism and Developmental Disorders 2011; 41: 805-814. |
| Hollander 2003 | Hollander E, Novotny S, Hanratty M et al. Oxytocin infusion reduces repetitive behaviors in adults with autistic and Asperger's disorders. Neuropsychopharmacology : official publication of the American College of Neuropsychopharmacology 2003; 28: 193-198. |
| Hollander 2005 | Hollander E, Phillips A, Chaplin W et al. A placebo controlled crossover trial of liquid fluoxetine on repetitive behaviors in childhood and adolescent autism. Neuropsychopharmacology 2005; 30: 582-589. |
| Hollander 2006A | Hollander, E., E. Swanson, E. Anagnostou, A. Phillips, W. Chaplin and S. Wasserman (2006). Liquid fluoxetine versus placebo for repetitive behaviors in childhood autism. Progress in neurotherapeutics and neuropsychopharmacology. New York, NY, Cambridge University Press; US: 105-113. |
| Hollander 2006B | Hollander E, Soorya L, Wasserman S et al. Divalproex sodium vs. placebo in the treatment of repetitive behaviours in autism spectrum disorder. International Journal of Neuropsychopharmacology 2006; 9: 209-213. |
| Hollander 2006C | Hollander E, Wasserman S, Swanson EN et al. A double-blind placebo-controlled pilot study of olanzapine in childhood/adolescent pervasive developmental disorder. Journal of Child and Adolescent Psychopharmacology 2006; 16: 541-548. |
| Hollander 2007 | Hollander E, Bartz J, Chaplin W et al. Oxytocin increases retention of social cognition in autism. Biological Psychiatry 2007; 61: 498-503. |
| Hollander 2010 | Hollander E, Chaplin W, Soorya L et al. Divalproex sodium vs placebo for the treatment of irritability in children and adolescents with autism spectrum disorders. Neuropsychopharmacology 2010; 35: 990-998. |
| Hollander 2012 | Hollander E, Soorya L, Chaplin W et al. A double-blind placebo-controlled trial of fluoxetine for repetitive behaviors and global severity in adult autism spectrum disorders. American Journal of Psychiatry 2012; 169: 292-299. |
| Hollander 2013A | Hollander E, Ferretti CJ, Taylor BP et al. Trichuris suis ova (TSO) as an immuneinflammatory treatment for repetitive behaviors in ASD. Neuropsychopharmacology 2013; 2): s391-s392. |
| Hollander 2013B | Hollander E. Translational experimental therapeutics of inflammation and fever in autism spectrum disorder: Hot tubs, locus coeruleus modulation and helminth therapy. Neuropsychopharmacology 2013; 2): s92-s93. |
| Hollander 2014A | Hollander E, Ferretti CJ, Taylor BP et al. Trichuris Suis Ova (TSO) as an immuneinflammatory treatment for repetitive behaviors in autism spectrum disorders (ASD). European Neuropsychopharmacology 2014; 2): s723. |
| Hollander 2014B | Hollander E, Del Valle Rubido M, Khwaja O et al. Affective speech recognition clinical biomarker effects of a novel vasopressin 1a receptor antagonist vs placebo in adult autism. Biological Psychiatry 2014; 75: 324s-325s. |
| Hollander 2014C | Hollander E, Del Valle Rubido M, Khwaja O et al. Clinical and biomarker effects of a novel vasopressin 1a receptor antagonist (RG7713) vs. Placebo in high functioning adult autism. Neuropsychopharmacology 2014; 39: s374-s375. |
| Hollander 2014D | Hollander E. V1A antagonist (RG7713) proof of mechanism study in high functioning autism spectrum disorder: Clinical, biomarker and social learning effects. Neuropsychopharmacology 2014; 39: s63. |
| Hollander 2018 | Hollander E, Uzunova G, Taylor BP et al. Randomized crossover feasibility trial of helminthic Trichuris suis ova versus placebo for repetitive behaviors in adult autism spectrum disorder. World Journal of Biological Psychiatry 2018. |
| Hollander 2019 | Hollander, E., U. S. D. o. Defense, G. P. Ltd. and M. M. Center (2019). Cannabidivarin (CBDV) vs. Placebo in Children With Autism Spectrum Disorder (ASD), https://ClinicalTrials.gov/show/NCT03202303. |
| Hollander 2020 | Hollander, E., G. Uzunova, B. P. Taylor, R. Noone, E. Racine, E. Doernberg, K. Freeman and C. J. Ferretti (2020). "Randomized crossover feasibility trial of helminthic Trichuris suis ova versus placebo for repetitive behaviors in adult autism spectrum disorder." World Journal of Biological Psychiatry 21(4): 291-299. |
| Hollway 2018 | Hollway JA, Mendoza-Burcham M, Andridge R et al. Atomoxetine, Parent Training, and Their Effects on Sleep in Youth with Autism Spectrum Disorder and Attention-Deficit/Hyperactivity Disorder. Journal of Child and Adolescent Psychopharmacology 2018; 28: 130-135. |
| Hollway 2019 | Hollway, J. A., L. E. Arnold, X. Pan, T. Wong, C. Li, C. E. Williams and R. R. Rice (2019). "12.1 Essential Oils for Improving Quality of Life and Anxiety in Asd." Journal of the American Academy of Child and Adolescent Psychiatry 58 (10 Supplement): S316. |
| Holopainen 2018 | Holopainen A, de Veld DMJ, Hoddenbach E, Begeer S. Does Theory of Mind Training Enhance Empathy in Autism? Journal of Autism and Developmental Disorders 2018. |
| Holtzer 2015 | Holtzer J. Efficacy of dir: Floortime in children with Autism Spectrum Disorders. Dissertation Abstracts International: Section B: The Sciences and Engineering 2015; 76: no pagination specified. |
| Honomichl 2002 | Honomichl RD, Goodlin-Jones BL, Burnham MM et al. Secretin and sleep in children with autism. Child Psychiatry and Human Development 2002; 33: 107-123. |
| Hopkins 2011 | Hopkins I, Gower M, Perez T et al. Avatar Assistant: Improving Social Skills in Students with an ASD Through a Computer-Based Intervention. Journal of Autism and Developmental Disorders 2011; 41: 1543-1555. |
| Hospital 2006 | Hospital UU, Child C, Health AM, Hospital OU. Joint Attention Intervention and Young Children With Autism. 2006. |
| Hospital 2009 | Hospital MG. Omega-3 Fatty Acids Monotherapy in Children and Adolescents With Autism Spectrum Disorders. 2009. - NCT01248130 |
| Hospital 2010 | Hospital MG, Speaks A. Mirtazapine Treatment of Anxiety in Children and Adolescents With Pervasive Developmental Disorders. 2010. |
| Hospital 2011 | Hospital MG. Double-blind Trial of Buspirone for the Treatment of Anxiety in Youth With Autism Spectrum Disorders. 2011. - NCT01395953 |
| Hospital 2012 | Hospital N, work T. Telephone Care Management to Address Sleep Problems in Young Children With Autism. 2012. - NCT01558180 |
| Hospital 2013 | Hospital RU, Centre Hospitalier Guillaume Régnier R. Melatonin Dose-effect Relation in Childhood Autism. 2013. - NCT01780883 |
| Hospital 2014A | Hospital MG, Hospital M. Behavioral and Neural Response to Memantine in Adolescents With Autism Spectrum Disorder. 2014. - NCT01972074 |
| Hospital 2014B | Hospital S, Pfizer. Dose Response Effects of Quillivant XR in Children With ADHD and Autism: A Pilot Study. 2014. - NCT02255565 |
| Hospital 2014C | Hospital B, Louisville U. Treatment of Children With Autism Spectrum Disorders and Epileptiform EEG With Divalproex Sodium. 2014. - NCT02094651 |
| Hospital 2015A | Hospital, M. G. and M. Hospital (2015). Behavioral and Neural Response to Memantine in Adolescents With Autism Spectrum Disorder, https://ClinicalTrials.gov/show/NCT01972074. |
| Hospital 2015B | Hospital U. Oxytocin in Adolescents With Autism Spectrum Disorders. 2015. - NCT02007447 |
| Hospital 2017A | Hospital MG. A Study of Oxytocin for the Treatment of Social Impairment in Individuals With High Functioning Autism Spectrum Disorder. 2017. - NCT02985749 |
| Hospital 2017B | Hospital MG. Probiotics and Oxytocin Nasal Spray on Social Behaviors of Autism Spectrum Disorder (ASD) Children. 2017. |
| Hospital 2018 | Hospital MG, Technology M. Improving Driving in Young People With Autism Spectrum Disorders. 2018. - NCT03538431 |
| Hospital 2020A | Hospital, P. C. s., S. U. o. N. Y.-D. M. Center, U. S. D. o. Defense, U. o. Arizona and N. Y. S. I. f. B. Research (2020). Early Treatment of Language Impairment in Young Children With Autism Spectrum Disorder With Leucovorin Calcium, https://ClinicalTrials.gov/show/NCT04060017. |
| Hospital 2020B | Hospital, P. C. s., A. Speaks, S. U. o. N. Y.-D. M. Center, U. o. Arizona and N. Y. S. I. f. B. Research (2020). Treatment of Social and Language Deficits With Leucovorin for Young Children With Autism, https://ClinicalTrials.gov/show/NCT04060030. |
| Howard 2005 | Howard JS, Sparkman CR, Cohen HG et al. A comparison of intensive behavior analytic and eclectic treatments for young children with autism. Research in Developmental Disabilities 2005; 26: 359-383. |
| Howlin 2007 | Howlin P, Gordon RK, Pasco G et al. The effectiveness of Picture Exchange Communication System (PECS) training for teachers of children with autism: A pragmatic, group randomised controlled trial. Journal of Child Psychology and Psychiatry and Allied Disciplines 2007; 48: 473-481. |
| Howlin 2018 | Howlin P, Begeer S, Hudry K. Interventions for children with autism: Identifying what works for whom. Journal of Intellectual Disability Research 2018; 62: 668. |
| Hugo W. Moser Research Institute at Kennedy Krieger 2012 | Hugo W. Moser Research Institute at Kennedy Krieger I, University of California SF. Omega-3 Fatty Acids for Hyperactivity Treatment in Autism Spectrum Disorder. 2012. - NCT01694667 |
| Iadarola 2017 | Iadarola S, Levato L, Harrison B et al. Teaching Parents Behavioral Strategies for Autism Spectrum Disorder (ASD): effects on Stress, Strain, and Competence. Journal of Autism and Developmental Disorders 2017; 1?10. |
| Iadarola 2018A | Iadarola S, Shih W, Dean M et al. Implementing a Manualized, Classroom Transition Intervention for Students With ASD in Underresourced Schools. Behavior Modification 2018; 42: 126-147. |
| Iadarola 2018B | Iadarola S, Levato L, Harrison B et al. Teaching Parents Behavioral Strategies for Autism Spectrum Disorder (ASD): Effects on Stress, Strain, and Competence. Journal of Autism and Developmental Disorders 2018; 48: 1031-1040. |
| Ibanez 2018A | Ibañez, L. V., K. Kobak, A. Swanson, L. Wallace, Z. Warren and W. L. Stone (2018). "Enhancing interactions during daily routines: a randomized controlled trial of a web-based tutorial for parents of young children with ASD." Autism research 11(4): 667‐678. |
| Ibanez 2018B | Ibanez LV, Kobak K, Swanson A et al. Enhancing interactions during daily routines: A randomized controlled trial of a web-based tutorial for parents of young children with ASD. Autism research : Official Journal of the International Society for Autism Research 2018; 11: 667-678. |
| Ichikawa 2013 | Ichikawa K, Takahashi Y, Ando M et al. TEACCH-based group social skills training for children with high-functioning autism: a pilot randomized controlled trial. Biopsychosocial Medicine 2013; 7: 14. |
| Ichikawa 2018 | Ichikawa H, Hiratani M, Yasuhara A et al. An open-label extension long-term study of the safety and efficacy of aripiprazole for irritability in children and adolescents with autistic disorder in Japan. Psychiatry and Clinical Neurosciences 2018; 72: 84?94. |
| Inc 2016 | Inc IM. ASD Parent Trainer: Online Coaching for Parents of Children With Autism. 2016. - NCT02469870 |
| Ingersoll 2012A | Ingersoll B. Brief report: effect of a focused imitation intervention on social functioning in children with autism. Journal of Autism and Developmental Disorders 2012; 42: 1768-1773. |
| Ingersoll 2012B | Ingersoll B. Effect of a focused imitation intervention on social functioning in children with autism. Journal of Autism and Developmental Disorders 2012; 42: 1768-1773. |
| Ingersoll 2015 | Ingersoll B, Berger NI. Parent Engagement With a Telehealth-Based Parent-Mediated Intervention Program for Children With Autism Spectrum Disorders: Predictors of Program Use and Parent Outcomes. Journal of Medical Internet Research 2015; 17: e227. |
| Ingersoll 2016 | Ingersoll B, Wainer A, Berger N et al. Comparison of a Self-Directed and Therapist-Assisted Telehealth Parent-Mediated Intervention for Children with ASD: A Pilot RCT. Journal of Autism and Developmental Disorders 2016; 46: 2275-2284. |
| Institute 2018 | Institute OBR. Aripiprazole Oral Solution in the Treatment of Children and Adolescents With Autistic Disorder. 2018. - NCT03487770 |
| IRCT 2017 | IRCT20090117001556N102 (2017). Prednisolone in autism spectrum disorders. |
| IRCT 2018A | IRCT20180518039703N1 (2018). An Investigation effect of Responsive Intervention Method on Improving Speech and Communication in Children Autism. |
| Irct 2018B | Irct20180721040549N (2018). "Evaluation of the Effect of Education based on Leventhal's Self-Regulation Model on The Psychological Distress of Parentâ€™s of Children with Autism in Qazvin, 1397." http://www.who.int/trialsearch/Trial2.aspx?TrialID=IRCT20180721040549N1. |
| Irct 2019A | Irct20101130005280N (2019). "Comparison between efficacy of reality therapy and mindfulness â€“based cognitive therapy mother's attitude towards child's with autism spectrum disorders." http://www.who.int/trialsearch/Trial2.aspx?TrialID=IRCT20101130005280N30. |
| IRCT 2019B | IRCT20150317021497N3 (2019). Effectiveness of happiness on parent-child relationships and hope. |
| IRCT 2019C | IRCT20180617040124N1 (2019). The Effect of Pivotal Response Treatment on children with Autism Spectrum Disorder. |
| IRCT 2019D | IRCT20181126041764N1 (2019). Effectiveness of self-differentiation psycho education in mothers of children with Autism Spectrum Disorder. |
| Irct 2019E | Irct20181221042068N (2019). "Investigate the Effectiveness of Ingersol Parental Education Treatment on the Symptoms of Autistim Disorder, functional emotional developmental level, and Parental Stress in Children with Autism Spectrum Disorder." http://www.who.int/trialsearch/Trial2.aspx?TrialID=IRCT20181221042068N1. |
| IRCT 2019F | IRCT20181227042147N1 (2019). The therapeutic effects of camel milk and chicory inulin in autistic children. |
| IRCT 2019G | IRCT20190420043334N1 (2019). The effectiveness of ??applied behavioral analysis and sensory enrichment on improving executive functions. |
| IRCT 2019H | IRCT20190505043477N1 (2019). "Narrative discourse intervention in children with autism". |
| IRCT 2019I | IRCT20190624043999N1 (2019). The Effect Of Foot Reflexology On The Habits Of Sleep Children With Autism. |
| IRCT 2019J | IRCT20190703044082N1 (2019). Emotional face identification in children with autism. |
| IRCT 2019K | IRCT20190727044346N1 (2019). Effectiveness of acceptance and commitment therapy for parents of children. |
| IRCT 2019L | IRCT20191009045039N1 (2019). The Impact of Xbox Kinect Games on the Static and Dynamic Balance of Autism Children. |
| IRCT 2019M | IRCT20101130005280N30 (2019). Comparison between efficacy of reality therapy and mindfulness –based cognitive therapy mother's attitude towards child's with autism spectrum disorders. |
| IRCT 2019N | IRCT20181221042068N1 (2019). Investigate the Effectiveness of Ingersol Parental Education Treatment on the Symptoms of Autistim Disorder, functional emotional developmental level, and Parental Stress in Children with Autism Spectrum Disorder. |
| Irct 2019O | Irct20181227042147N (2019). "The therapeutic effects of camel milk and chicory inulin in autistic children." http://www.who.int/trialsearch/Trial2.aspx?TrialID=IRCT20181227042147N1. |
| Irct 2019P | Irct20190624043999N (2019). "The Effect Of Foot Reflexology On The Habits Of Sleep Children With Autism." http://www.who.int/trialsearch/Trial2.aspx?TrialID=IRCT20190624043999N1. |
| Irct 2019Q | Irct20190703044082N (2019). "Emotional face identification in children with autism." http://www.who.int/trialsearch/Trial2.aspx?TrialID=IRCT20190703044082N1. |
| IRCT 2020A | IRCT20090117001556N124 (2020). Cilostazol in the treatment of Autism. |
| IRCT 2020B | IRCT20120215009014N330 (2020). Effect of coenzyme Q10 versus placebo on improving symptoms of autism spectrum disorder. |
| IRCT 2020C | IRCT20180503039517N5 (2020). Effects of vitamin D and/or aquatic exercise on IL-1ß and IL-1RA serum levels and behavior of children with autism spectrum disorder. |
| IRCT 2020D | IRCT20180707040370N8 (2020). Exploring the effect of extended-reality training contents on basic recognition deficits in children with Autism Spectrum Disorder. |
| IRCT 2020E | IRCT20190204042612N1 (2020). The effect of stories on children's sleep disorders. |
| IRCT 2020F | IRCT20190703044082N2 (2020). Comparison of the effect of traditional and computerized emotional facial education training on children with autism. |
| IRCT 2020G | IRCT20190714044199N1 (2019). Efficacy of N-Acetyl cysteine in patients with autism spectrum disorder. |
| IRCT 2020H | IRCT20190915044774N1 (2020). The effect of Curcumin on autism spectrum disorder. |
| IRCT 2020I | IRCT20191025045234N1 (2020). Effectiveness of Trans-Cranial Direct Current Stimulation (TDCS) in children with autism. |
| IRCT 2020J | IRCT20191113045429N1 (2020). Virtual Reality and Autism. |
| IRCT 2020K | IRCT20200317046801N2 (2020). The effect of ondansetron on autism. |
| IRCT 2020L | IRCT20200614047773N1 (2020). The Effect of Happiness Educational on Expressed emotions and Quality of Life of Mothers of Children with Autism spectrum disorders. |
| IRCT 2020M | IRCT20200712048084N1 (2020). Effect of Vitamin C in treatment of Autism Spectrum Disorders. |
| Irct1138901151556n 2010 | Irct1138901151556N. memantine in the treatment of autism. 2010. |
| Irct138711091556n 2009 | Irct138711091556N. Celecoxib and Autism. 2009. |
| Irct138711161556n 2009 | Irct138711161556N. Pentoxifylline in the treatment of autism. 2009. |
| Irct138808202698n 2010 | Irct138808202698N. Efficacy of intercessory prayer on severity of symptoms of children with autism and parental stress. 2010. |
| Irct138901141556n 2010 | Irct138901141556N. Tpoiramate in the treatment of autism. 2010. |
| Irct138904204264n 2010 | Irct138904204264N. Physical education effects in autistic children. 2010. |
| Irct20090117001556n 2017 | Irct20090117001556N. Resveratrol in treatment of autism. 2017. |
| Irct20090117001556n 2018A | Irct20090117001556N. Sulforaphane as adjunctive treatment of irritability in children with Autism spectrum disorder. 2018. |
| Irct20090117001556n 2018B | Irct20090117001556N. Efficacy of pregnenolon in treatment of irritability inteeanagers with autism. 2018. |
| Irct20090117001556n 2018C | Irct20090117001556N. Folinic acid in the treatment of autism spectrum disorder. 2018. |
| Irct20090117001556n 2018D | Irct20090117001556N. Effect of propentofylline in children with autism. 2018. |
| Irct201012031556n 2010 | Irct201012031556N. Ginkgo biloba in the treatment of autistic disorder. 2010. |
| Irct201101105280n 2011 | Irct201101105280N. Cyproheptadin plus Risperidon in treatment of children with Autistic Disorder: a double blind, placebo controlled study. 2011. |
| Irct201106101556n 2011 | Irct201106101556N. Amantadine in the treatment of autism. 2011. |
| Irct201106103930n 2011 | Irct201106103930N. N-Acetylcysteine augmentation with Rispridone in treatment of Autism in children. 2011. |
| Irct201107281556n 2011 | Irct201107281556N. Riluzole in the treatment of autism. 2011. |
| Irct201108155280n 2013 | Irct201108155280N. A comparative study on the effectiveness of Risperidone versus Risperidone plus naltrexone in treatment of autistic spectrum disorder in children with 6-12 years old. 2013. |
| Irct201109037462n 2011 | Irct201109037462N. Efficacy of using ball playing in movement skills and coping behaviors in children with autism. 2011. |
| Irct201110233930n 2011 | Irct201110233930N. Aripiprazole versus risperidone for treatment of autism. 2011. |
| Irct201110281556n 2011 | Irct201110281556N. N-acetyl cysteine in the treatment of autism. 2011. |
| Irct201202281556n 2012 | Irct201202281556N. pioglitazone in the treatment of autism. 2012. |
| Irct201204037202n 2012 | Irct201204037202N. Comparing efficacy and side effects of Memantine and Risperidone in treating autistic patients. 2012. |
| Irct201204081556n 2012 | Irct201204081556N. Galantamine in the treatment of autism. 2012. |
| Irct201204246834n 2012 | Irct201204246834N. The effect of cognitive group therapy on empowerment of parents with autistic child in autism institute of Tabriz city, 2012. 2012. |
| Irct201205259854n 2012 | Irct201205259854N. Pivotal Response Treatment (PRT) in Autism. 2012. |
| Irct2012091010806n 2013 | Irct2012091010806N. Music therapy in autism. 2013. |
| Irct2012111011421n 2013 | Irct2012111011421N. Effect of omega-3 supplementation on Autistic patients. 2013. |
| Irct201212079854n 2012 | Irct201212079854N. Sensory Integration in Autistic Children. 2012. |
| Irct201302201556n 2013 | Irct201302201556N. Minocycline in the treatment of autism. 2013. |
| Irct201307303930n 2014 | Irct201307303930N. A randomized double blind placebo controlled clinical trial of buspirone for treating autism spectrum disorders. 2014. |
| Irct20130904014562n 2018 | Irct20130904014562N. Responsiveness to the hepatitis B vaccine in Autistic child. 2018. |
| Irct20131013014994n 2018 | Irct20131013014994N. effect of vitamin D on Autism Spectrum Disorders. 2018. |
| Irct2013110915339n 2016 | Irct2013110915339N. Pidrolax With or Without A Gluten-Free Diet on Gastrointestinal Symptoms and Behavioral Of Autistic Children. 2016. |
| Irct201402043930n 2014 | Irct201402043930N. short-term co-administration of acid folicfor treating children and adolescents with autism. 2014. |
| Irct20140212016564n 2018 | Irct20140212016564N. The Effect of logotherapy on Perceived stress, Parent-Child Relationship and Meaningfulness of Mothers of Children with Autism Disorder. 2018. |
| Irct201404212017n 2014 | Irct201404212017N. The effect of gluten free diet on gastrointestinal and behavioral indices in children with ASD. 2014. |
| Irct201404222394n 2014 | Irct201404222394N. Efficacy of vitamin D supplementation in children with attention deficit hyperactivity disorder. Http://wwwwhoint/trialsearch/trial2aspx? Trialid=irct201404222394n10 2014. |
| Irct201405273930n 2014 | Irct201405273930N. Vitamin D for treating autism. 2014. |
| Irct2014102519665n 2014 | Irct2014102519665N. The effectiveness of using visual pedagogy in dental check up and preventive dentistry for children with autism compare with autistic children without education. 2014. |
| Irct2014102919732n 2015 | Irct2014102919732N. Group Intervention for Children with Autism. 2015. |
| Irct20150519022323n 2017 | Irct20150519022323N. The effect of Perceptual motor Exercises along with music and Vitamin D3 Supplementation in children with autism spectrum disorder. 2017. |
| Irct2015080223454n 2015 | Irct2015080223454N. Effect of mobile Autism parenting skills education. 2015. |
| Irct201512081556n 2015 | Irct201512081556N. L Carnosine in the treatment of Autism. 2015. |
| Irct2015122625699n 2016 | Irct2015122625699N. The effect of Omega-3 on Autism. 2016. |
| Irct201512315280n 2016 | Irct201512315280N. Efficacy Stem Cell in Treatment Autism. 2016. |
| Irct201602041556n 2016 | Irct201602041556N. Simvastatin in the treatment of Autism. 2016. |
| Irct2016022826802n 2016 | Irct2016022826802N. Assessment the efficacy of atomoxetin(stramox) in autism spectrum disorders. 2016. |
| Irct2016061711689n 2016A | Irct2016061711689N. Effects Of Carnosine On Autism. 2016. |
| Irct2016061711689n 2016B | Irct2016061711689N. Effects of carnosine supplementation on autism disorder. 2016. |
| Irct2016061828511n 2016 | Irct2016061828511N. super brain yoga on children with autism disorder. 2016. |
| Irct2016071728966n 2016 | Irct2016071728966N. Hippotherapy is an innovative approach for enhancing the physical factor of people who suffer from Autism Spectrum Disorder. 2016. |
| Irct2016080829268n 2017 | Irct2016080829268N. Evaluation of the efficacy of memantine in children with attention deficit hyperactivity disorder. Http://wwwwhoint/trialsearch/trial2aspx? Trialid=irct2016080829268n1 2017. |
[truncated: 311,079 more chars]
